# Ageing and degeneration analysis using ageing-related dynamic attention on lateral cephalometric radiographs

**DOI:** 10.1038/s41746-022-00681-y

**Published:** 2022-09-27

**Authors:** Zhiyong Zhang, Ningtao Liu, Zhang Guo, Licheng Jiao, Aaron Fenster, Wenfan Jin, Yuxiang Zhang, Jie Chen, Chunxia Yan, Shuiping Gou

**Affiliations:** 1 Key Laboratory of Shaanxi Province for Craniofacial Precision Medicine Research, College of Stomatology, Xi’an Jiaotong University, Xi’an, 710004 Shaanxi China; 2 College of Forensic Medicine, Xi’an Jiaotong University Health Science Center, Xi’an, 710061 Shaanxi China; 3 Department of Orthodontics, the Affiliated Stomatological Hospital of Xi’an Jiaotong University, Xi’an, 710004 Shaanxi China; 4 Key Laboratory of Intelligent Perception and Image Understanding of Ministry of Education, School of Artificial Intelligence, Xidian University, Xi’an, 710071 Shaanxi China; 5 Robarts Research Institute, Western University, London, N6A 3K7 ON Canada; 6 Academy of Advanced Interdisciplinary Research, Xidian University, Xi’an, 710071 Shaanxi China; 7 Department of Radiology, the Affiliated Stomatological Hospital of Xi’an Jiaotong University, Xi’an, 710004 Shaanxi China

**Keywords:** Geriatrics, Forensic dentistry, Digital radiography in dentistry

## Abstract

With the increase of the ageing in the world’s population, the ageing and degeneration studies of physiological characteristics in human skin, bones, and muscles become important topics. Research on the ageing of bones, especially the skull, are paid much attention in recent years. In this study, a novel deep learning method representing the ageing-related dynamic attention (ARDA) is proposed. The proposed method can quantitatively display the ageing salience of the bones and their change patterns with age on lateral cephalometric radiographs images (LCR) images containing the craniofacial and cervical spine. An age estimation-based deep learning model based on 14142 LCR images from 4 to 40 years old individuals is trained to extract ageing-related features, and based on these features the ageing salience maps are generated by the Grad-CAM method. All ageing salience maps with the same age are merged as an ARDA map corresponding to that age. Ageing salience maps show that ARDA is mainly concentrated in three regions in LCR images: the teeth, craniofacial, and cervical spine regions. Furthermore, the dynamic distribution of ARDA at different ages and instances in LCR images is quantitatively analyzed. The experimental results on 3014 cases show that ARDA can accurately reflect the development and degeneration patterns in LCR images.

## Introduction

In the past few decades, the world’s population has been ageing dramatically as countries have a rising life expectancy resulting from improved healthcare. This trend emerged first in developed countries but is now also common in developing countries. The aged population is currently at its highest level in human history, and according to the United Nations’world population ageing report, the number of people over the age of 60 years in the world will climb to 1.4 billion by 2030.

Population ageing does raise some formidable new challenges in the cost of social security, health care, and the well-being of the elderly^1^. The increase in the total number and the elderly population proportion results in the decline in the proportion of the social labor force and rising pension costs^2^. In addition, population ageing is also a signal of the advent of a tidal wave of chronic and non-communicable diseases such as cardiovascular disease, cancer, diabetes, and chronic respiratory diseases, which draws the attention of researchers and the government to understand and cope with the ageing and degeneration of humans from different perspectives.

Many methods have been applied to the research of human ageing, such as genomics, where the expression levels of RNAs were studied as a function of ageing to characterize age-associated changes in skeletal muscle gene expression of healthy individuals^3^, proteomics, where a proteomic ageing clock comprised of proteins was proposed to predict the human age in the studies of human proteomics ageing^4^, and clinical investigations, which focused on how dietary and pharmacological interventions promote a healthy lifespan by influencing energy intake and circadian rhythms^5^.

Compared with genomics, proteomics, and clinical investigation approaches, radiomics methods have advantages in cost, dataset scale, and convenience. There have been some radiomics studies for human ageing, such as analyzing the correlation between cervical spine alignment and ageing by manually measuring geometric features^6^, and evaluating the 3D mandibular dental changes using the registration of digital models^7^.

The above-mentioned studies have attempted to understand or characterize the ageing of humans from different perspectives or to explore the factors that cause and delay ageing.

Within these studies, it is easy to find that the ageing process of the human body is highly correlated to age changes. At different ages, there are obvious age-related changes in various tissues, while ageing is also a direct manifestation of the increase of age. In most age estimation tasks, the basis of age estimation comes from the characteristics of ageing^8–10^, which inspires us to extract ageing features by an age estimation task.

With the rapid growth of data scale and computing power, deep learning methods have been widely used in healthcare. Gialluisi et al. used a deep neural network to predict mortality and hospitalization risk with multiple circulating biomarkers^11^. Lima et al. used deep neural networks with electrocardiograms to predict the age of patients and explored the correlation between the difference between predicted and actual age and death^12^. In many medical image analysis tasks, the convolutional neural networks (CNNs) have achieved promising performances in recent years, including classification^13^, detection^14^, segmentation^15,16^, registration^17^, and general feature characterization^18^. Deep learning methods are also widely used for age estimation tasks because of the strong representational capabilities and performance far exceeds that of handcrafted feature extraction methods. Deep learning methods have been applied to age estimation in various applications and imaging modalities. For example, specially designed CNNs were used in the task of estimating age with shoeprint images that commonly used in forensics^19^ and masked 3D keen MRI images^20^. X-ray images of hand-wrist bones and teeth were also widely used in age estimation tasks with deep learning models^8,21^.

The lateral cephalometric radiograph (LCR) image, which is one of the most commonly used dental X-ray radiographs in dental clinics, are selected as our research objects. The LCR image can provide more clinical clues about ageing features since it contains more regions such as craniofacial bones and the cervical spine than intraoral periapical radiographs (IPR) images and dental panoramic radiographs (DPR) images. As a result, an age estimation deep learning model trained on a large scale LCR image dataset was used as a promising method for characterizing ageing-related features in LCR images.

Although it is still difficult to track the complete development and ageing process of an individual due to the difficulty of sampling and continuous tracking of a single individual, a large number of study samples distributed across different ages can be used to characterize the process of human development and ageing. In addition, features based on a large number of samples are more objective and general than those obtained from a single individual. Therefore, a fully automated radiomics feature learning method on LCR images is proposed to discover the radiomics ageing representation and relationship between development/ageing and age based on a large number of samples distributed across consecutive ages.

In this study, ageing salience, the numerical form of ageing-related attention, is used to characterize the degree of correlation between regions in an image and ageing in an orderly manner, which can be used to intuitively visualize the drastic degree of ageing changing in LCR images. The general ageing attention obtained from the feature extractor is named ageing-related dynamic attention (ARDA), and the value of ARDA is the average ageing salience as it represents developmental and ageing patterns common to a large number of subjects. The proposed method can show the distribution of average ageing salience and ageing region and their dynamic changes in the LCR images.

In this study, an automatic human development and ageing analysis methodology using deep learning on the LCR images is proposed. Based on this methodology, the quantitative dynamic distribution of ARDA on LCR images is demonstrated. Three ARDA concentrated regions including the teeth, craniofacial, and cervical spine regions are found by the distribution of ARDA. The proposed method not only validates the findings of previous studies of human ageing, but also demonstrates the change process of ageing regions that are not discovered by traditional ageing research methods. The performance of age estimation for adult subjects is significantly improved by the proposed ARDA-constrained model, and the ARDA-guided age estimation model provides a new perspective and solution for research of clinical degenerative diseases and forensic practice.

## Results

### The LCR image

LCR images are the most commonly-used dental X-ray radiographs in dental clinics. All the LCR images in this study were acquired with a Cranex D digital X-ray unit (Soredex, Tuusula, Finland) for diagnosis and therapeutic purposes. The exposure parameters for the LCR images were 73 kV and 7 mA, with an exposure time of 11.7 s. All LCR images are standardized and archived in the Digital Imaging and Communications in Medicine (DICOM) format.

**Fig. 1:**
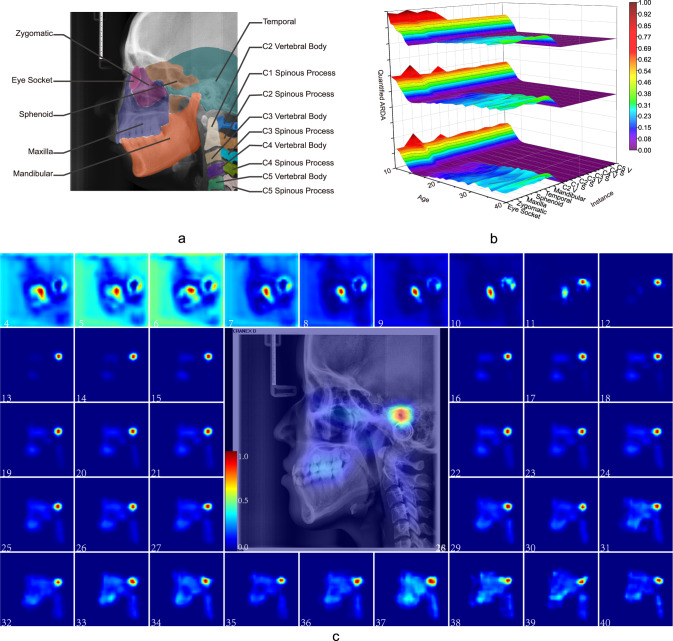
The ARDA of LCR images. The ARDA is generated from LCR images and a pre-trained deep learning ageing feature extractor. **a** Instances in LCR images. The parietal and frontal instances are not labeled because the ARDA is barely distributed on them. **b** Illustration of the quantitative distribution of ARDA on instances in LCR images. The three dimensions are age, instance and quantified ARDA. The contours and position of each instance are shown in **a**. The quantified ARDA of each instance is the mean value of the average ageing salience of the ageing-significant regions it contains. From the bottom to the top are the cases in which the threshold of ageing-significant region is set to the median, 75-th percentile, and 90-th percentile of corresponding ARDA, respectively. In the dimension of instances, Cx V and Cx S represent the x-th vertebral body and the x-th spinous processes of the cervical spine, respectively. **c** The ARDA map of LCR images. To visualize the distribution of ARDA and ageing regions, we mapped the average ageing salience to a color ranging from blue to red. The closer the color of the pixel in the ARDA map is to red, the larger the average ageing salience of the pixel. We display the LCR image of a randomly selected 28-year-old subject overlaid with its corresponding ageing salience map to show the relative position between the ARDA map and the LCR image. Source data are provided as a Source Data file.

The LCR images contained craniofacial bones, teeth, and C1-C5 of the cervical spine, which can provide more information about the development and ageing than IPR and DPR images. As shown in Fig. 1a, we labeled several instances in LCR images according to the anatomical structure to quantitatively analyze ageing salience.

### Ageing representation using ARDA in LCR images

The quantified ARDA of each instance in LCR images is shown in Fig. 1b. Only regions with average ageing salience larger than the threshold were considered to be the ageing-significant regions. Setting the ageing-significant region threshold can filter out the ageing irrelevant regions and avoid the influence of instance size in the quantitative analysis of ARDA. The thresholds of ageing-significant region for each LCR image were set to the median, 75-th and 90-th percentile of its corresponding average ageing salience. For the ARDA at each age, the mean value of average ageing salience in the ageing-significant region contained in each instance was calculated as the quantified ARDA of the instance.

According to the quantified ARDA at the instance scale of ARDA, all the instances in the LCR images are highly correlated to ageing before the age of 9 years. When the threshold is increased, the changing patterns of ARDA in the age dimension remained unchanged, while the sphenoid and temporal bones show more obvious ageing correlations in the instance dimension as the distribution of ARDA is more concentrated in these two instances. From the perspective of the dynamic changes with age, the ARDA is significant but attenuates rapidly during the rapid development period. After that, the average ageing salience increases and tends to change slowly.

As shown in Fig. 1b, c, ARDA is distributed widely in all regions of LCR images as evident during the growth and development period, especially from 4 to 9 years old. After that, although ageing salience and ageing regions grow steadily, they are concentrated in several partial regions in the LCR image. This shows that the development process of the human body is widely reflected in various regions contained in LCR images, while ageing is mainly concentrated in several local regions. In detail, the ageing salience and the ageing region in the ARDA maps before the age of 10 years are greater than that after the age of 10 years, but both decrease until the age of 12 years. The blank regions without any tissue in the LCR images shrink with the development of the tissue, resulting in these regions also showing high ageing salience.

After the age of 12 years, the ARDA concentrates in the zygomatic, maxilla, and mandibular bones steadily. The common feature of these instances is that ARDA is evenly and extensively distributed in them. They still show high ageing salience in the quantitative average salience analysis, even if there is not extremely salience spot in them. After the age of 20 years, the eye socket and sphenoid bone also show ageing salience occasionally. At later ages, both the area and the salience of the ageing-significant region increase slowly.

From an instance point of view, before the age of 9 years, the parietal, frontal, and maxilla bones show a strong ageing correlation, which is consistent with the development stage of the skull and maxilla. At the ages of 10 to 13, the zygomatic, maxilla, and mandibular bones show high ageing salience, suggesting that there are rapid developments and changes in the craniofacial bones. Moreover, the regions with obvious average ageing salience do not appear accidentally, but usually expand or strengthen from an existing one, which suggests that ageing is a slow and progressive process relative to developmental processes, and it extends usually from one tissue to the surrounding region. It is worth noting that there is a particularly conspicuous region above the external auditory canal, where the ARDA is more visible than other regions in the LCR images.

### Three ARDA concentrated regions: teeth, craniofacial and cervical spine

**Fig. 2:**
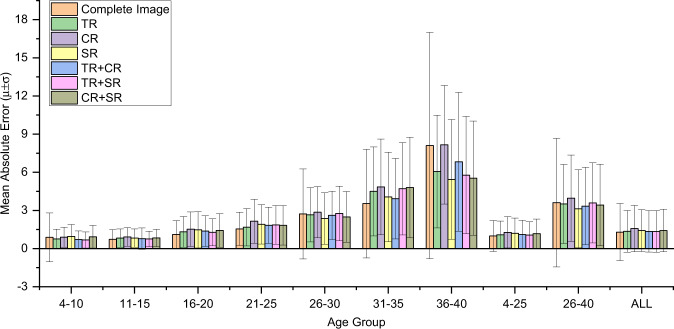
The performance comparison of age estimation in each age group. The performances were obtained by using complete LCR images, the LCR images in which one ARDA concentrated region is selected, and the LCR images in which one ARDA concentrated region is discarded as the input of the baseline model (TR teeth region, CR craniofacial region, SR cervical spine region, *μ* mean of absolute error, and *σ* standard deviation of the absolute error). The heights of the bars indicate the values of the *μ*. The upper and lower endpoints of error bar in each bar indicate the values of *μ* + *σ* and *μ* − *σ*, respectively. Overall, the accuracy of age estimation with complete LCR images is better than the accuracy of any of the three regions being selected or discarded. Source data are provided as a Source Data file.

As shown in Fig. 1c, the ageing regions in the ARDA map remain consistent after the age of 12 years, which can be roughly divided into three ARDA concentrated regions: the teeth, craniofacial, and cervical spine as demonstrated in Fig. 6a. One or two single regions of the three regions were used to train the baseline network for age estimation. The age estimation performances in Tables 1, 2, and Fig. 2, show that similar performance can be achieved as long as one of the three regions of the teeth, craniofacial, and cervical spine regions are included when LCR images are used for age estimation in an uncontrolled environment. The similar performance can relieve the requirement of age estimation for complete human tissues in LCR images and provide a new perspective and the clinical method, especially in the practice of forensic medicine. This confirms that ARDA can represent well the distribution of real ageing and development information on LCR images and shows that the developmental and ageing information in the LCR images is redundant. See the Supplementary Table 1 for the pearson correlation coefficient and *p*-value between real ages and the predicted ages.

**Table 1:** Tabulated measurements results of the mean of absolute error (MAE) ± standard deviation of the absolute error (SD) (years).

Age	4–10	11–15	16–20	21–25	26–30	31–35	36–40	4–25	26–40	ALL
BSL	0.88 ± 1.92	**0.73** **±** **0.76**	**1.11** **±** **1.09**	**1.55** **±** **1.31**	2.72 ± 3.54	3.54 ± 4.27	8.10 ± 8.89	1.00 ± 1.22	3.61 ± 5.06	1.30 ± 2.24
AC	1.06 ± 0.85	0.95 ± 0.89	1.35 ± 1.05	1.80 ± 1.52	**2.35** **±** **1.78**	**3.52** **±** **2.65**	**7.00** **±** **5.44**	1.21 ± 1.10	**3.08** **±** **2.90**	1.42 ± 1.54
RTS	0.80 ± 0.68	0.75 ± 0.62	1.14 ± 1.02	1.60 ± 1.26	2.44 ± 1.90	4.71 ± 3.63	7.20 ± 4.30	**1.00** **±** **0.93**	3.55 ± 3.22	**1.28** **±** **1.59**
TR	0.76 ± 0.76	0.83 ± 0.72	1.32 ± 1.20	1.67 ± 1.46	2.66 ± 2.13	4.50 ± 3.50	6.06 ± 4.44	1.09 ± 1.08	3.51 ± 3.10	1.35 ± 1.63
CR	0.89 ± 0.78	0.91 ± 0.73	1.53 ± 1.36	2.15 ± 1.73	2.86 ± 2.01	4.85 ± 3.77	8.16 ± 4.67	1.28 ± 1.23	3.96 ± 3.40	1.57 ± 1.83
SR	0.96 ± 0.93	0.83 ± 0.72	1.48 ± 1.42	1.91 ± 1.55	2.36 ± 2.03	4.06 ± 3.52	5.43 ± 4.71	1.20 ± 1.20	3.14 ± 3.06	1.41 ± 1.64
TR+CR	0.71 ± 0.68	0.79 ± 0.83	1.37 ± 1.21	1.83 ± 1.42	2.61 ± 1.90	3.93 ± 3.17	6.82 ± 5.45	1.10 ± 1.12	3.34 ± 3.05	1.35 ± 1.63
TR+SR	**0.68** **±** **0.64**	0.76 ± 0.62	1.28 ± 1.06	1.86 ± 1.51	2.76 ± 2.14	4.70 ± 3.63	5.78 ± 4.62	1.07 ± 1.04	3.59 ± 3.15	1.34 ± 1.64
CR+SR	0.93 ± 0.89	0.84 ± 0.68	1.43 ± 1.32	1.83 ± 1.55	2.49 ± 1.99	4.80 ± 3.96	5.54 ± 4.49	1.17 ± 1.15	3.43 ± 3.21	1.42 ± 1.68

The constraint of ARDA can improve the stability of age estimation dramatically, especially in the subject of older populations, while it reduces the precision in the subjects of younger populations. The retest mechanism can maintain the benefits of ARDA constraint for age estimation and offset its adverse effect. The impact on age estimation is limited for any single ARDA concentrated region being selected or discarded. The teeth and the craniofacial regions have the most and the least information on development/ageing, respectively. The best performance in each age group is bolded.

*BSL* baseline model, *AC* ARDA-constrained, *RTS* retest, *TR* teeth region, *CR* craniofacial region, *SR* cervical spine region.

**Table 2:** Tabulated measurements results of the cumulative score-5 (CS-5) and mean cumulative score-5 (MCS-5) (%).

Metric	Age	4–10	11–15	16–20	21–25	26–30	31–35	36–40	4–25	26–40	All
CS-5	BSL	98.01	99.74	99.15	**98.90**	92.00	**86.21**	58.70	99.18	86.31	97.68
	AC	**100.00**	99.74	**99.85**	95.81	**94.59**	80.72	50.00	99.14	**86.94**	**97.78**
	RTS	**100.00**	99.91	99.13	98.02	90.95	77.65	**64.86**	99.40	84.64	**97.78**
	TR	99.49	**100.00**	99.13	98.02	90.95	66.28	54.05	99.37	80.48	97.28
	CR	**100.00**	**100.00**	97.54	94.49	90.00	66.28	21.62	98.43	76.28	95.99
	SR	99.23	99.91	97.97	96.04	93.33	70.93	63.89	98.66	84.34	97.08
	TR+CR	**100.00**	99.65	98.84	97.57	90.95	72.29	43.75	99.14	81.85	97.21
	TR+SR	**100.00**	**100.00**	99.42	97.80	90.95	69.41	43.24	**99.48**	80.12	97.35
	CR+SR	99.49	**100.00**	98.55	96.70	93.33	66.28	59.46	98.99	82.58	97.18
MCS-5	BSL	88.02	**88.92**	**81.89**	**74.76**	62.37	**54.21**	30.43	**84.57**	**56.28**	**81.25**
	AC	83.07	84.65	77.70	70.64	62.24	46.59	20.31	80.26	54.40	77.37
	RTS	87.02	88.13	81.42	70.70	57.86	48.82	31.98	83.30	52.66	79.92
	TR	88.04	86.89	78.37	72.72	58.02	36.82	25.68	82.48	48.95	78.77
	CR	85.37	85.56	75.10	65.05	54.37	34.88	14.41	79.37	44.89	75.56
	SR	84.52	86.89	76.21	68.14	**62.38**	42.44	**36.57**	80.62	54.42	77.74
	TR+CR	89.45	87.82	77.44	69.50	57.32	43.78	22.40	82.27	50.64	78.73
	TR+SR	**89.86**	87.84	79.15	69.90	56.19	35.69	29.28	82.87	47.94	79.02
	CR+SR	84.94	86.84	76.79	70.12	59.76	37.98	32.88	81.15	51.15	77.83

The best CS-5 and MCS-5 in each age group are bolded.

*BSL* baseline model, *AC* ARDA-constrained, *RTS* retest, *TR* teeth region, *CR* craniofacial region, *SR* cervical spine region.

In the comparison of overall age estimation performance, the teeth region contains more development/ageing information than the other two regions, while the craniofacial region contains the least. Specifically, the performance of age estimation using the teeth region is the best in the 4–25 age group, while the cervical spine region performs best for the older subjects in the 26–40 age group. In the 26–40 age group, the accuracy and stability of the age estimation using the cervical spine region suggest the unique importance of this region for ageing research of mature adult subjects.

### The dynamic distribution of ARDA in ARDA concentrated regions

**Fig. 3:**
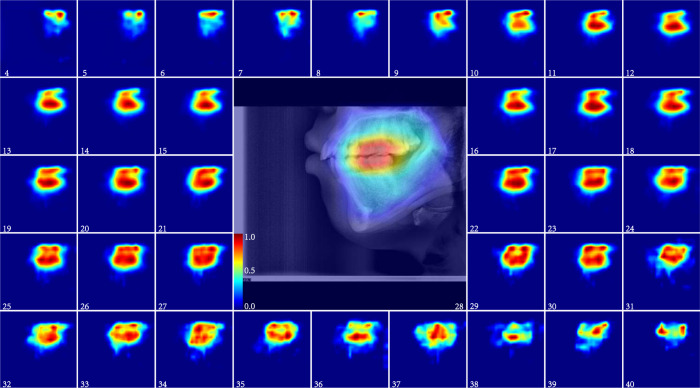
The ARDA map of the teeth region from the ages of 4 to 40 years. The central panel displays the teeth region of a randomly selected 28-year-old subject overlaid with its corresponding ageing salience map to show the relative position between the ARDA map and the teeth region of the LCR image. Source data are provided as a Source Data file.

**Fig. 4:**
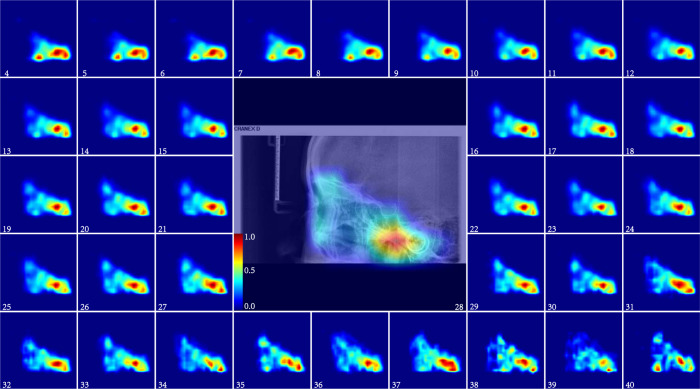
The ARDA map of the craniofacial region from 4 to 40 years old. The central panel displays the craniofacial region of a randomly selected 28-year-old subject overlaid with its corresponding ageing salience map to show the relative position between the ARDA map and the craniofacial region of the LCR image. Source data are provided as a Source Data file.

**Fig. 5:**
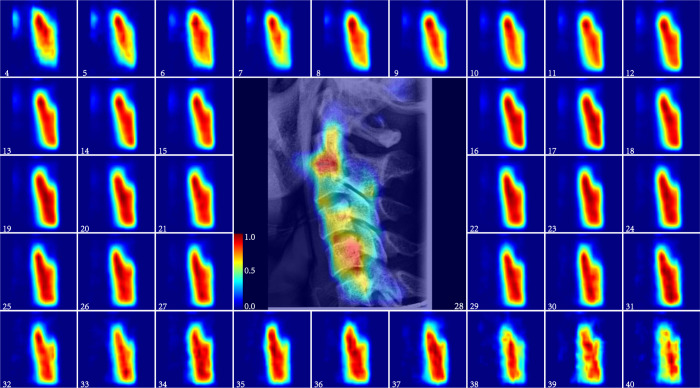
The ARDA map of the cervical spine region from the ages of 4 to 40 years old. The central panel displays the cervical spine region of a randomly selected 28-year-old subject overlaid with its corresponding ageing salience map to show the relative position between the ARDA map and the cervical spine region of the LCR image. Source data are provided as a Source Data file.

**Fig. 6:**
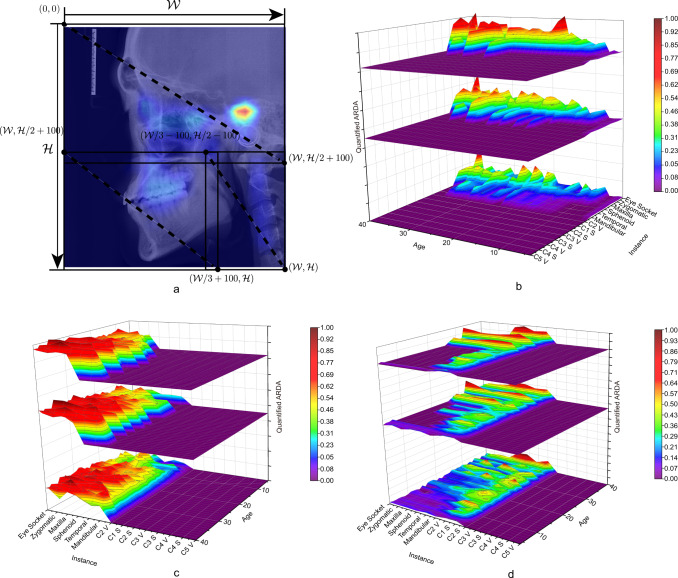
Illustration of quantified ARDA in ARDA concentrated regions. **a** The position of the teeth, craniofacial and cervical spine regions in the LCR images. The coordinates of the upper left and lower right corners of each region are specified. The coordinate system is established with the upper left corner of the LCR images as the origin, the positive direction of the *x* axis to the right, and the positive direction of the *y* axis to the down. **b**, **c**, **d** are the quantified ARDA of each instance of the teeth, craniofacial, and cervical spine regions, respectively. In **b**, **c**, **d** the three surface plots from bottom to top are the cases in which the ageing-significant region threshold is set to the median, 75-th, and 90-th percentile of average ageing salience, respectively. Source data are provided as a Source Data file.

More detailed ARDA maps of the three ARDA concentrated regions: the teeth, craniofacial, and cervical spine are shown in Figs. 3, 4, and 5, respectively, and the quantitative analysis at the instance scale of these regions is illustrated in Fig. 6b, c, d, respectively. As demonstrated in both the ARDA maps and quantitative analysis, the common feature of the ARDAs in the three regions is that their distribution range and average ageing salience continued to increase with age from 4 to 40 years, unlike the pattern of its distribution in the LCR images. However, similar to the distribution of ARDA in LCR images, the most obvious ageing regions (i.e., the red or dark red regions in the ARDA map) in these regions change steadily with age.

In the teeth region, as shown in Fig. 6b, the mandibular and maxilla are the main instances showing ageing salience, which can be reflected in the ARDA map in Fig. 3 where the upper teeth and lower teeth are the main regions of the ARDA distributions. In addition, the ARDA in the lower teeth region is much more obvious than the upper teeth at age ranges from 4 to 40 years except for the ages before 10 years and after 37 years.

In the craniofacial region, the difference in the quantified ARDA of each instance is not obvious, while the quantified ARDA of the eye socket and maxilla bone is slightly greater than that of other instances. The ARDA map of the craniofacial region in Fig. 4 shows that the growth of the average ageing salience is flat, and there are two most obvious ageing regions in the temporal bone. In addition, the two regions that always show ARDA are the frontal bone and the eye socket.

In the cervical spine region, as shown in Fig. 6d, the average ageing salience of C2 is obviously higher than that of other instances before the age of 25 years. After that, ARDA starts to be evenly distributed across instances. In the age range of 4 to 40 years, the quantified ARDA of the vertebral bodies is higher than that of the spinous processes, which indicates that the ARDA is widely distributed in the vertebral bodies.

Furthermore, the effects of increasing the threshold of the ageing-significant region on the quantitative ARDA in the teeth region and craniofacial region mainly appear in the sphenoid, temporal, and mandibular bones in the instance dimension, while the effect on the pattern of change in the age dimension is not significant. In the case of the cervical spine region, the pattern of the quantified ARDA changes moderately with age, while the increase in the threshold causes fluctuations.

In summary, the distribution of ARDA is basically the same as the ageing region proposed by existing studies^22–26^, and there are several regions with obvious average ageing salience are worthy of being verified by subsequent studies such as the upper and lower posterior teeth, maxillary tuberosity in Fig. 3, the frontal bone and eye socket of in Fig. 4, and the spinous processes of C2–C5 in Fig. 5.

## Discussion

In this study, we propose a human development and ageing characterizing method using a deep learning model as feature extractor. The proposed method reveals the distribution of ARDA in LCR images and dynamic changes in time (ages from 4 to 40 years old) and space (instances in LCR image). Our results demonstrate that the ARDA changes dynamically with age and show the changing patterns of ARDA. The age estimation experiments show that ARDA and age information are distributed mainly in three independent regions. As well, the performance and stability of age estimation were improved by the proposed ARDA.

The proposed method can accurately describe the ageing salience and dynamics of the human craniofacial and cervical spine with advantages over previous studies in terms of objectivity, dynamics, and efficiency.

The age estimation method with ARDA constraint was designed in this study. The constraint of ARDA can significantly improve the stability of the model for age estimation, which reduces the overall standard deviation (SD) of the mean absolute error (MAE) from 2.24 to 1.54 years (31.25% reduction). The SD of the absolute error in the age estimation for the 26–40 age group decreased from 5.06 to 2.90 years (42.69% reduction), which shows that the proposed ARDA can improve the stability for the older subjects’age estimation. In addition, the age estimation accuracy for subjects of 26–40 years is also improved by ARDA, in which the MAE decreased from 3.61 to 3.08 years (14.68% reduction). The age estimation error of younger subjects increased slightly by the ADRA constraint, compared to the baseline model. Therefore, the proposed retest method combines the advantages of age estimation with ARDA constraint and age estimation without ARDA constraint and obtains the smallest MAE under the premise of improving stability. In practice, the retest method described in Section should be a feasible solution. The performance of age estimation experiments with incomplete images demonstrates the redundancy of age information in the completed LCR images, which can provide new perspectives and workflows for age estimation and forensic practice.

Craniofacial development is a complex and irreversible process, involving age-related changes in bone and soft tissue. The developmental process that can be quantified allows researchers to have a clearer picture of the development of craniofacial bones and teeth. Previous related ageing and developmental studies attempted to characterize ageing changes in tissues from the perspective of geometric measurements, which include measuring changes in head circumference^27^ and skull volume^28^, and measuring sutural growth displacement of the maxilla^29^, etc. The ageing-significant regions revealed by the proposed ARDA map corresponding developmental period are basically consistent with the developing regions of craniofacial and teeth proven in previous related ageing studies.

**Fig. 7:**
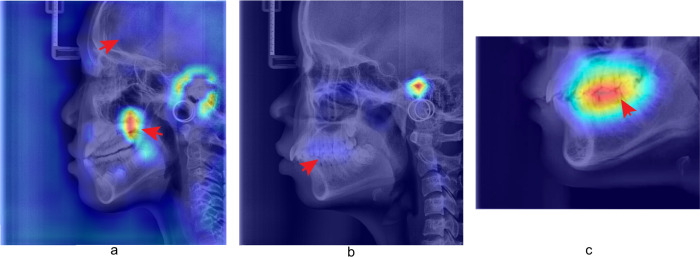
Examples of regions in ARDA map showing ageing salience that can be cross validated with existing research. **a** The LCR image and corresponding ageing salience map of a 7-year-old subject, which represents the ARDA distribution on LCR image in the period from 4 to 7 years of age. The arrows from top to bottom point to the cranial region, and posterior margin of the maxilla, respectively. **b** The LCR image and corresponding ageing salience map of an 18-year-old subject, which represents the ARDA distribution on LCR image in the period from 8 to 18 years of age. The arrow points to the upper and lower dentition. **c** Tooth region of LCR image and corresponding ageing salience map of a 18-year-old sample, which represents the ARDA distribution on the teeth region of LCR image in the period from 8 to 18 years of age. The arrow points to the upper and lower dentitions.

At the age of 0 to 7 years, the skull is in the most intense period of growth. Generally, the skull volume of a child can exceed 90% of that of an adult by the age of 7 years, while the growth after the age of 10 years is minimal^27,28,30^. As shown in Fig. 7a, the cranial region in the ARDA map between the age of 4 to 7 years shows significant ageing salience, which disappears in the ARDA map after the age of 10 years. The change in ageing salience is highly consistent with the findings of the previous studies.

The growth of maxillary length is mainly through bone deposition with the palatal suture and the maxillary tuberosity and peaks at the age of 11 years^29,31^. The growth center is at the posterior margin of the maxilla^32^ indicated by the arrow in Fig. 7a, which shows strong salience, while the salience in this region disappears consistently after the age of 11 years.

The temporary teeth dentition fully erupts at the age of 2.5 years, and the permanent tooth germs develop before the age of 6 years, after which, the temporary teeth are replaced during the period from 6 to 14 years of age. Therefore, the ageing salience of the upper and lower teeth region at this period is associated with the eruption and replacement of teeth. The wear of teeth appears after the teeth fully erupt, so the ageing salience in this region after the age of 15 years is related to tooth wear. As shown in Fig. 7b, the ageing salience in the upper and lower dentitions regions gradually increases but is not the most significant after the age of 15 years, while in Fig. 7c the ageing salience is significant. The difference in ageing salience between the same region on complete and incomplete LCR images is caused by that the ARDA represents ageing salience in a relative or ordinal manner. The ageing salience of a given region is not only related to the absolute degree of its ageing change but also depends on its relative rate of change in the image.

In summary, the average ageing salience shown in the ARDA maps and the quantified ARDA can also be well explained by findings from studies related to craniomaxillofacial.

In addition, the ageing-significant regions of the teeth and maxillary sinus ageing shown in the ARDA map during ageing period are also consistent with the findings of related studies on tooth wear^22,23^ and maxillary sinus ageing^24–26^. Researchers have conducted in-depth research on the ageing changes of the eye socket^33,34^, in which the eye sockets are proven to increase with age. In the cervical spine region, research shows that structural changes of the cervical spine begin in middle age, but sometimes earlier^35^. Intervertebral disc degeneration begins at adolescence, and as it progresses, it can also lead to morphological alterations of the vertebral bodies, while the cervical lordosis increased with age^36^.

Although few related studies that can validate our method in ageing period, our method is widely validated by the existing research during the growth and development period. Therefore, we can reasonably speculate that our proposed ARDA is still reliable during ageing and that the unidentified significant regions of ageing/development revealed by our method are also reasonably valuable for follow-up studies.

Current research tends to estimate the age based on ageing changes in the bones. But there are few studies on ageing itself and its salience as well as regional dynamic changes with age. The feasibility of our method in representing the development and ageing of the craniofacial and cervical spine provides a new perspective and ageing metrics for ageing research.

Our method can capture the distribution of developmental/ageing quantitative salience on LCR images of healthy individuals aged 4 to 40 years. The analysis in time (age) and space (instance) dimensions shows that the ageing salience exhibits regularity, which is general, objective, and credible based on a large-scale subject set. Therefore, the regularity of ARDA distribution in different ages and different instances proposed in this study can be initially used as a clinical quantitative auxiliary index to detect whether there is abnormal ageing/development in a subject.

**Fig. 8:**
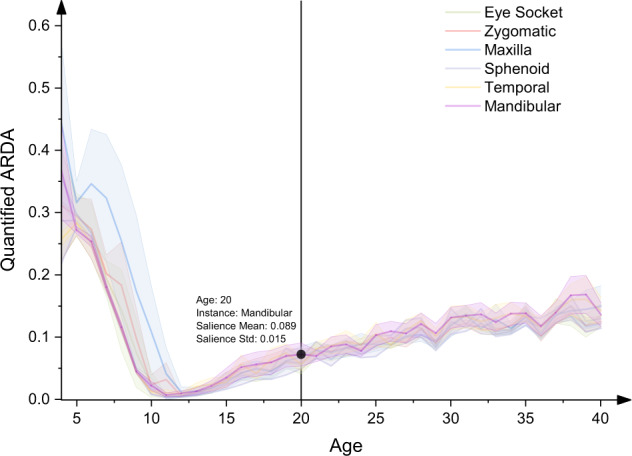
The distribution of ARDA across instances and ages. The threshold of the ageing-significant region is set to the 75-th percentile. For each instance, the points on the line and the interval indicate the mean of the ageing salience and the interval of mean ± standard deviation of the ageing salience, respectively. A point in the instance of mandibular bone is selected as the example. This point shows that the mean and standard deviation of the ageing salience of 3018 subjects are 0.089 and 0.015 for mandibular bone at the age of 20 years, respectively.

As shown in Fig. 8, which demonstrates the distribution of our proposed ARDA across instances and ages for all 3014 subjects in the testing set. The distribution of quantified ARDA is narrower for each instance, especially when the age is greater than 10 years, which suggests that the ARDA has the potential as a standard indication of ageing and development. Assuming that the distribution of the ARDA follows a normal distribution, the highlighted point in Fig. 8 indicates that for a healthy subject of 20 years old, the probability that the quantified ARDA of mandibular bone generated by our method is in the range of 0.059 to 0.119 is 95.45%. The distribution range of ARDA width decreases with the increase of the threshold of the ageing-significant region. In this sense, we suggest that the ARDA has potential as a standard indication of ageing and development.

It should be noted that the feasibility of ARDA as a quantitative ageing/developmental indicator still needs further validation, such as the inclusion of gender factors, a wider range of ages, and a larger data dataset, which will be the focus of our future studies. The index can only be used as a computer-aided diagnosis but is not a viable substitute for fully automated diagnosis by physicians due to the size and age range limitations of the current dataset and the lack of cross-validation in subsequent ageing and developmental studies.

In addition to validating the findings of existing studies, our model also finds a few ageing-significant regions that have not been validated by previous studies. For example, the circumferential (before the age of 10 years) and punctate (after the age of 10 years) regions above the external auditory canal, are also shown correspondingly in the ARDA map of the craniofacial region. However, tissues in LCR images are overlapping, and ARDA on 2D scale cannot accurately represent the ageing salience of tissues in 3D space. Feature work on age-related changes in this area will be carried out on 3D images to eliminate the limitation of tissue overlap on 2D X-ray images.

Physiological changes in the human can be roughly divided into two stages, development and ageing. The developmental and ageing forms of face can reflect the differences between these two stages^37^. The age range from 4 to 40 years chosen in this study could basically cover the development and ageing periods. Children under the age of 4 years are rarely examined with lateral radiographs, and X-ray examination cannot be performed on research subjects for research purposes due to medical ethical reasons. Thus, we chose the age of 4 years as the lower limit of our study age range. In the related research on age estimation using traditional manual feature extraction methods and deep learning^8,38^, the performance of age estimation decreases with the age of the subjects during the ageing period, which is also a challenge for related studies^39^. In the study of age estimation using orthopantomogram images and 3D facial images related to LCR images, the estimated age performance is significantly worse after the age of 40 years^8,40^. In addition, the number of orthodontic patients in northwestern China decreased rapidly from the age of about 15 years as shown in Table 3, which also results in poor performance of age estimation and theinability to obtain accurate and generalized ageing characteristics. Therefore, the age of 40 years was set as the upper age limit for this study.

**Table 3:** The age distribution of the LCR images.

	Dataset			Gender		
Age	Total	Train	Val	Test	Male	Female
4–10	2599	1822	393	384	1264	1335
11–15	7652	5354	1148	1150	3072	4580
16–20	4591	3211	690	690	1713	2878
21–15	3044	2137	454	453	815	2229
26–30	1452	1020	210	222	297	1155
31–35	577	408	86	83	102	475
36–40	259	190	37	32	39	220
All	20,174	14,142	3018	3014	7302	12,872

Limited by the data we collected, our study covers a partial ageing period of people aged from 4 to 40 years. However, based on the verifiability of the ageing and its changing patterns in the LCR images represented by the proposed ARDA, especially the consistency with the findings of existing studies during the development period, future work will extend the ARDA to the whole life span of humans and more anatomical features in application scenarios where the scale of the training data allows.

Another limitation of our proposed ARDA is that it characterizes ageing salience in a relative manner, i.e., highlighting the most ageing-significant regions in LCR images and cannot be used to establish an accurate mapping relationship with physical quantities such as area, rate of tissue change, etc. Correlating ARDA with physical quantities of ageing changes and extending it as an index for aided diagnosis of ageing abnormalities is also a feasible direction for future work.

## Methods

In this study, an ageing and degeneration analysis method using ARDA on LCR images is proposed. The overview of the proposed method is shown in Fig. 10. The method mainly consists of four modules: (1) a pre-trained deep learning model for ageing feature extraction, (2) ARDA generation, (3) acquisition of three ARDA concentrated regions, and (4) quantitative analysis of ageing and degeneration distribution. Among them, the ageing features extracted by the pre-trained age estimation model are used to generate ageing salience maps and obtain the ARDA map. Three ARDA concentrated regions of the LCR image are divided based on the ARDA distributions. Finally, the ARDA of both the LRC image and the ARDA concentrated regions are quantitatively analyzed at the pixel and the instance scales.

### Ethics statement

This study is approved by the Affiliated Stomatological Hospital of Xi’an Jiaotong University Health Science Center (Approval number: xjkqll[2022]NO.30). The study was non-interventional and retrospective, all participants in the study signed the written informed consent, and the LCR images used in this data were anonymized. A sampled and desensitized example dataset was shared in source code repository.

### Study population

We obtained LCR images from the Stomatological Hospital of Xi’an Jiaotong University Health Science Center, China. The bit depth of the images in the dataset is 16 bpp, and the size of most images is 2144 × 2304.

**Fig. 9:**
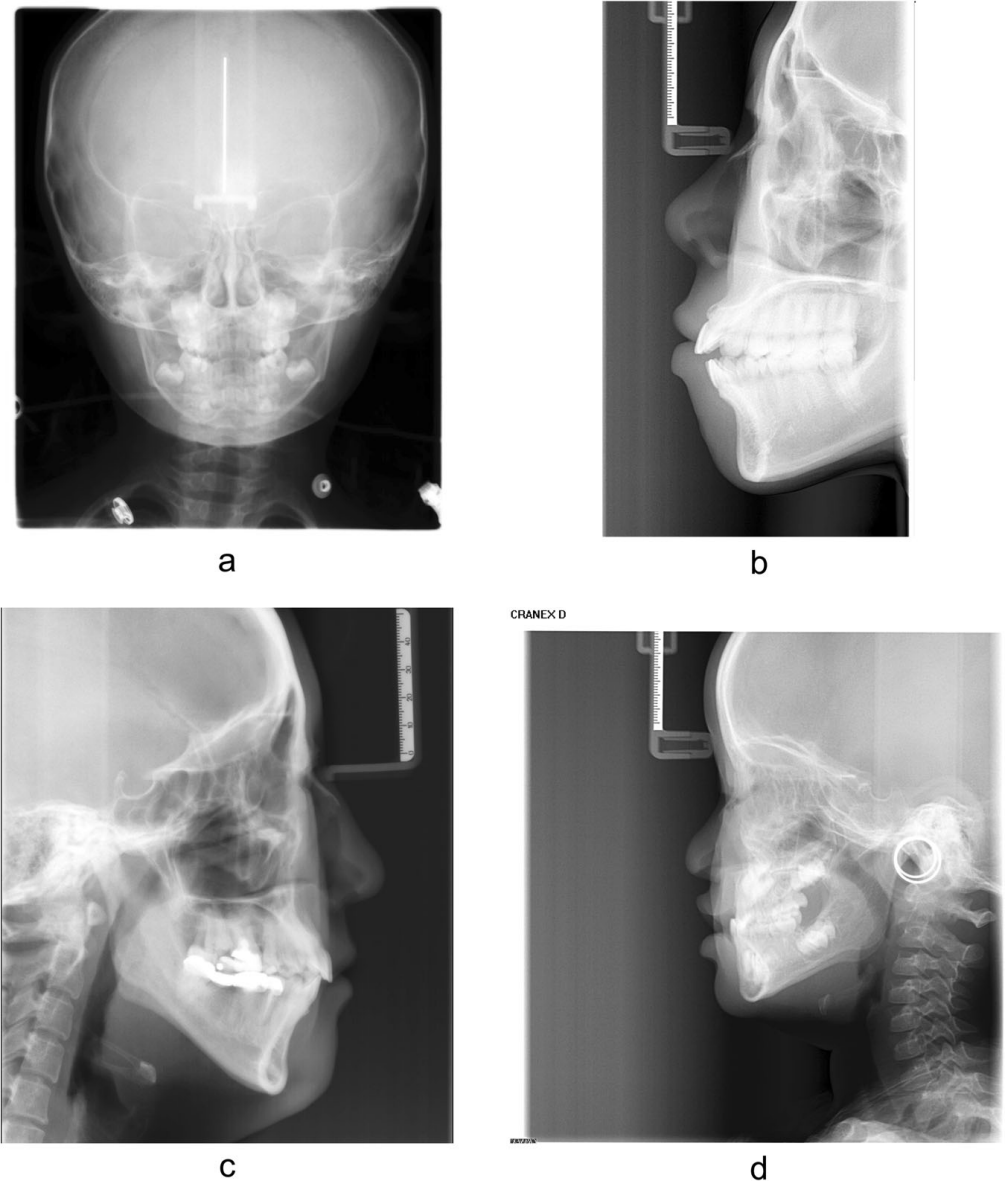
Examples of some typical unqualified images. **a** The actual age of the subject is less than 4 years or greater than 40 years. **b** Incomplete LCR image. **c** Subject with restorations in the teeth. **d** Incorrect imaging posture.

The age of the subjects was calculated by subtracting the imaging date from the date of birth and dividing by 365.25 (due to leap years) and rounding to the nearest hundredth. 20174 LCR images were divided into 7 age groups according to age after screening the unqualified images shown in Fig. 9. The age and gender distribution of the subjects are shown in Table 3. The size distribution of the LCR images is shown in Table 4.

**Table 4:** The size distribution of LCR images.

Width	Height	Number
1804	2136	5338
2136	2304	2050
2140	304	4000
2144	2304	7179
2148	2304	1524
Others		83
Sum		20174

The proposed ARDA is robust to low quality images since it is based on the block-scale, high-dimensional, and abstract features of the images, and it represents the ageing regions without emphasizing pixel-scale precision. The manifestation of ARDA is the representation of the difference between the center of the ageing region from the surrounding region. Therefore, the impact on the accuracy of ARDA of the possible low quality image with blurring, low resolution, and noise is limited.

### Ageing feature extraction

In this study, the ageing features of LCR images were extracted by a pre-trained CNN for age estimation. When the CNN was trained to estimate age, the smaller the error, the more accurately the ageing characteristics in the LCR image can be extracted. For the age estimation tasks using large LCR images (larger than 1500 × 2000), the balance of performance and computing resource requirements needs to be considered.

EfficientNet-B0 can achieve the best result with the fewest parameters compared with other CNNs, so it was used as the baseline age estimation model and ageing feature extractor. The performance comparison for age estimation and the number of parameters from the CNN models that perform well on natural images is shown in Table 5. These models were trained using a tiny version of the training set for quick comparison.

**Table 5:** Age estimation performance comparison of ageing feature extractors.

Model	#Para	Metric	4–10	11–15	16–20	21–25	26–30	31–35	36–40	All
Res18	12M	E.Med	−0.13	−0.48	−0.92	−1.07	0.18	1.80	5.26	−0.20
		MAE	0.93	1.33	2.03	2.49	2.65	3.76	5.99	2.15
		SD	0.76	1.27	1.70	1.90	2.05	3.84	4.77	2.37
		IOR	1.15	1.23	2.31	2.9	2.38	4.56	6.32	2.23
Res34	22M	E.Med	−0.2	−0.08	−0.38	−0.35	−0.18	1.94	3.59	−0.10
		MAE	0.92	1.18	1.64	2.34	2.28	2.89	5.65	1.86
		SD	0.85	0.96	1.22	1.70	1.78	2.50	5.68	2.01
		IOR	1.02	1.12	1.65	2.27	2.16	2.32	5.61	1.96
Res50	26M	E.Med	−0.31	−0.29	−0.92	−1.43	0.21	2.86	4.18	−0.20
		MAE	0.99	1.36	2.15	2.72	2.76	4.04	5.37	2.24
		SD	0.84	1.14	1.86	1.97	2.23	3.70	4.28	2.34
		IOR	1.00	1.25	2.25	2.14	2.62	4.48	3.82	2.37
Res101	45M	E.Med	−0.20	−0.13	−0.85	−0.64	1.16	2.71	5.64	0.00
		MAE	1.00	1.35	1.96	2.27	2.7	3.87	6.05	2.13
		SD	0.89	1.33	1.56	1.89	2.03	3.34	4.37	2.32
		IOR	1.01	1.23	2.31	2.62	2.31	4.42	5.03	2.24
SERes101	45M	E.Med	0.62	0.33	1.58	0.75	−0.72	−4.78	−8.09	0.17
		MAE	1.40	1.75	2.63	2.69	2.59	4.92	7.97	2.59
		SD	1.47	1.83	2.09	2.10	2.14	3.24	5.63	2.65
		IOR	1.38	1.88	2.74	3.14	3.17	4.75	7.2	2.82
Res152	60M	E.Med	−0.20	−0.13	−0.66	−1.05	0.31	2.29	5.21	−0.20
		MAE	1.03	1.38	1.92	2.35	2.45	3.77	5.76	2.09
		SD	1.00	1.17	1.72	1.80	1.56	3.84	4.32	2.31
		IOR	1.14	1.35	2.08	2.56	2.39	3.59	5.25	2.22
Dens121	8M	E.Med	0.33	0.26	−0.30	−0.53	0.62	2.54	5.47	0.31
		MAE	1.02	1.39	2.00	2.06	3.05	3.82	5.72	2.15
		SD	0.78	1.24	1.42	1.58	2.29	3.63	4.29	2.25
		IOR	1.06	1.47	2.11	2.15	2.74	3.26	4.51	2.21
Dems161	29M	E.Med	−0.61	−0.30	−0.33	−0.58	1.57	4.28	7.39	0.06
		MAE	1.02	1.15	1.85	2.37	2.71	4.91	7.94	2.27
		SD	0.80	0.97	1.62	1.92	1.96	3.68	4.53	2.52
		IOR	1.13	1.18	1.73	2.65	2.53	4.15	6.24	2.36
IncepV4	43M	E.Med	−0.27	−0.30	−0.75	−0.37	0.72	2.68	6.71	−0.50
		MAE	0.80	1.16	1.82	2.42	2.50	3.53	6.62	2.02
		SD	0.68	1.12	1.50	2.00	2.16	3.56	4.00	2.30
		IOR	0.77	1.32	1.74	2.74	2.76	3.89	3.92	1.51
IncepRes	29M	E.Med	0.22	0.02	0.32	1.14	−0.24	−2.36	−3.58	0.09
		MAE	0.82	1.22	1.47	2.36	2.38	2.95	5.79	1.85
		SD	0.72	1.16	1.14	1.64	1.85	2.44	6.01	2.07
		IOR	0.74	1.06	1.62	2.47	2.5	2.54	6.48	1.97
DaNet	8M	E.Med	1.54	3.72	3.21	−0.99	−5.82	−10.69	−15.39	0.19
		MAE	3.33	4.44	4.03	3.27	6.16	10.16	15.54	5.11
		SD	3.17	3.10	3.04	2.54	3.77	3.60	4.38	4.28
		IOR	3.52	4.19	4.05	3.59	5.4	5.48	5.59	5.65
EffiB0	5M	E.Med	0.07	0.23	0.17	0.48	-0.33	−0.76	−2.76	0.07
		MAE	**0.75**	**1.02**	**1.39**	**2.03**	**2.10**	**2.33**	**5.14**	**1.60**
		SD	0.68	0.79	1.09	1.76	1.74	2.65	5.62	1.95
		IOR	0.68	0.73	1.12	1.55	2.28	3.34	6.26	1.15

All the error metrics are given in years. The best MAE in each age group is bolded.

#Para: the number of model parameters, *MAE* mean absolute error, *SD* the standard deviation of MAE, *IOR* the interquartile range of the absolute error.

Efficient-B0 was trained for age estimation on the training set, in which the pre-processing process of the LCR images included contrast enhancement, shape fixing, and resizing. In addition, random affine transformation and random horizontal flip were also used for data augmentation.

The main block of Efficient-B0 is a mobile inverted bottleneck (MBConv)^41,42^, to which the squeeze-and-excitation optimization^43^ was also added. The structure of Efficient-B0 is shown in Table 6. See the Supplementary Note 1 for a detailed description of the EfficientNet model structure.

**Table 6:** The structure of Efficient-B0.

Stage	Operator	Resolution	#Channels	#Layers
*i*	${\hat{{{{\mathcal{F}}}}}}_{i}$	${\hat{H}}_{i}\times {\hat{W}}_{i}$	${\hat{C}}_{i}$	${\hat{L}}_{i}$
1	Conv3 × 3	224 × 224	32	1
2	MBConv1,k3 × 3	112 × 112	16	1
3	MBConv6,k3 × 3	112 × 112	24	2
4	MBConv6,k5 × 5	56 × 56	24	2
5	MBConv6,k3 × 3	28 × 28	80	3
6	MBConv6,k5 × 5	14 × 14	112	3
7	MBConv6,k5 × 5	14 × 14	192	4
8	MBConv6,k3 × 3	7 × 7	320	1
9	Conv1 × 1 & Pooling & FC	7 × 7	1280	1

The loss function for ageing feature extractor was set as L1 loss:

1 $$L=\frac{1}{N}\mathop{\sum }\limits_{n=1}^{N}| {y}_{n}^{\prime}-{y}_{n}|$$

where *N* is the number of samples, *y*_*n*_ and ${y}_{n}^{\prime}$ are the age and predicted age of *n*-th LCR image.

The training strategy, image augmentation and preprocessing for the training process are provided in the Supplementary Note 2 and Supplementary Note 3, respectively. The fitted regression models on the true age and the age predicted by different age estimation methods are show in Supplementary Figure 1. The *p*-values of *f*-test and *t*-test between different age estimation methods are shown in Supplementary Table 2 and Supplementary Table 3, respectively.

### ARDA generation

**Fig. 10:**
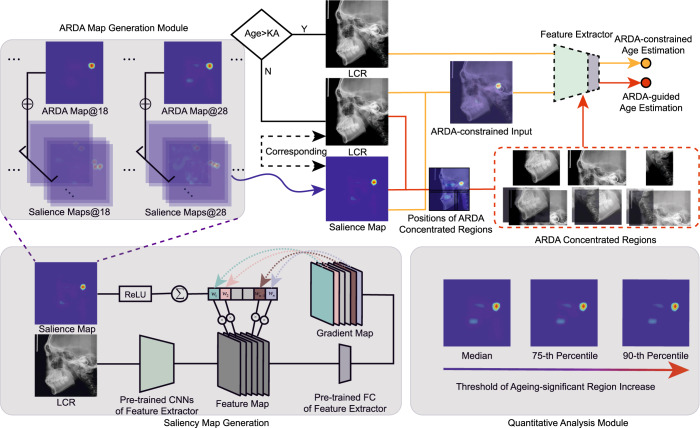
Overview of the method used in this study. The method includes ageing feature extraction, ageing salience map generation and ARDA generation, three ARDA concentrated regions acquisition and quantitative analysis of ageing distribution modules. The data flow of ARDA-constrained age estimation is indicated by yellow lines, while the data flow of ARDA-guided age estimation is indicated by red lines. The feature extractor and fully connected layer of the baseline model in the ARDA Map Generation Model are pre-trained.

With the pursuit of the interpretability of deep learning methods, some methods for gradient visualization of deep CNNs have been proposed, including CAM^44^, Grad-CAM^45^, etc. The gradient visualization method can be used not only to visualize the salience region of the model, but also to represent the ageing-related attention by the ageing feature extractor. The details of the ARDA generation module are shown in Fig. 10. The global average of the gradient of the feature map in the pre-trained model is defined as its weight, and the weight of the *k*-th feature map inputted into the fully connected layer is calculated by

2 $${w}_{k}=\frac{1}{Z}\mathop{\sum}\limits_{i}\mathop{\sum}\limits_{j}\frac{\partial \hat{y}}{\partial {F}_{ij}^{k}}$$

where, *Z* is the number of pixels in feature map *F*, $\hat{y}$ is the output of network, and ${F}_{ij}^{k}$ is the value of pixel (*i*, *j*) in the *k*-th feature map. The ageing salience map *M* is obtained by calculating the weighted sum of all the feature maps:

3 $$M=ReLU\left(\mathop{\sum}\limits_{k}{w}_{k}{F}^{k}\right)$$

The ARDA map of age *a* is generated by:

4 $${A}_{a}=\frac{1}{{N}_{a}}\mathop{\sum }\limits_{n=1}^{{N}_{a}}{M}_{a,n}$$

where *a* ∈ {4, 5, …, 39, 40}, *N*_*a*_ is the number of samples with age *a* in the training set, *M*_*a*,*n*_ is the ageing salience map corresponding to *n*-th LCR image of age *a*, and ∑ is element-wise summation.

Here, the ageing salience maps are generated by the pre-trained age estimation network which can extract generic ageing features from LCR images.

The ARDA is obtained by merging all ageing salience maps of the same age. The general distribution of the ageing salience on the LCR images can be displayed, while avoiding the effect of abnormal samples. The ARDA generated for each age can show the dynamic changes of ageing salience with age in LCR images.

Age-related changes are not significant in adults. Therefore, it is difficult to estimate accurately adult age using only LCR images. Here, we propose a method to apply salience maps as the attention constraint of the neural network, which can make use of the knowledge learned by the baseline model on all training data to all input LCR images and enable the baseline model to focus more on ageing-relevant elements.

In the training phase of the ARDA-constrained age estimation, the LCR images older than the selected KA and their corresponding salience maps are used as the input of the baseline model. Otherwise, only the LCR images are used as the input. This distinction is acceptable because age labels are available during the training phase. However, in the testing phase, as the age labels are not available, we applied a different strategy, i.e., if the predicted age from the age estimation model is greater than KA, we concatenate the LCR images and their corresponding ageing salience map as the input of the network and estimate the age again. The latter estimation result is regarded as the final estimated age. Otherwise, the result of the first test is directly used as the final estimated age. We call this method retest.

### Acquisition of three ARDA concentrated regions

From forensic practice, we know that a complete LCR image is sometimes difficult to obtain, and the ageing salience of the local region in the LCR images can be achieved by analyzing the distribution of ARDA on the LCR images. The distribution of ageing salience on the LCR images represented by the ARDA is shown in Fig. 1c. These ARDA concentrated regions are the teeth, craniofacial and cervical spine regions. Under the guidance of ARDA, each LCR image in our dataset is divided into three overlapping parts with the same strategy, as the position of each part in the LCR image is fixed. We mapped the ARDA to color, ranging from blue to red, to visualize the distribution of ageing salience and ageing regions, i.e., the closer the color of the pixel is to red, the larger the ARDA of the pixel. An LCR image of an individual of the age of 28 years and its corresponding ageing salience map are shown overlapping to show the relative position relationship between the ARDA map and the LCR image in Figs. 3, 4, and 5.

Figure 6 a shows a specific LCR image with a width ${{{\mathcal{W}}}}$ and height ${{{\mathcal{H}}}}$, in which the upper left corner was set as the origin of the coordinate system, the positive *x*-axis is in the down direction, and the positive *y*-axis is in the right direction. Table 7 summarizes the coordinate of the upper left corner and the lower right corner of the three ARDA concentrated regions.

**Table 7:** The position of the three ARDA concentrated regions. Set the upper left corner of the LCR image as the origin of the coordinate system.

Region	Upper Left	Lower Right
Teeth	$(0,\frac{1}{2}{{{\mathcal{H}}}}-100)$	$(\frac{2}{3}{{{\mathcal{W}}}}+100,{{{\mathcal{H}}}})$
Craniofacial	(0, 0)	$({{{\mathcal{W}}}},\frac{1}{2}{{{\mathcal{H}}}}+100)$
Cervical Spine	$(\frac{2}{3}{{{\mathcal{W}}}}-100,\frac{1}{2}{{{\mathcal{H}}}}-100)$	$({{{\mathcal{(W}}}},{{{\mathcal{H}}}})$

The positive *x*-axis is in the down direction, and the positive *y*-axis is in the right direction. The coordinates (*x*, *y*) of the upper left and lower right corners of each ARDA concentrated region. The boxes of the LCR images facing the right are mirror-symmetrical with those of the LCR images facing the left.

Each ARDA concentrated region of the LCR image is selected or discarded as the input of the model for comparing the performance of the age estimation and providing guidance for forensic practice, The ARDA maps of each region are also generated as the same way as we obtain the ARDA maps of the complete LCR images.

### Quantitative analysis of ageing and degeneration

In this study, the quantified analysis of ARDA is performed in the time and space dimensions. In the time dimension, the patterns of dynamic salience and region of ageing with age in LCR images are analyzed. In the spatial dimension, the quantitative ageing salience of LCR images is represented on the pixel scale and the instance scale.

As shown in Fig. 1a, the size of the instances in the LCR images varies greatly. To eliminate the effect of size differences on the quantified ARDA analysis, we only calculate the mean ARDA of the ageing-significant region within each instance. The quantified ARDA generation of the instances is detailed in Supplementary Note 4.

In this study, the ageing-significant region threshold is set to the median value, the 75-th quantile and the 90-th quantile of the average ageing salience corresponding to a specific age. In Fig. 6b, c, d, the three surface plots from the bottom to top are the cases in which the ageing-significant region threshold was set to the median, 75-th and 90-th percentile of the average ageing salience, respectively.

### Reporting summary

Further information on research design is available in the Nature Research Reporting Summary linked to this article.

## Supplementary information

Supplementary Material

Reporting Summary

## Data Availability

The data used in this study is not open access due to privacy and security concerns. After obtaining the sharing agreement, it can be shared with third parties for reasonable use, relevant requests should be addressed to C.Y. (yanchunxia@mail.xjtu.edu.cn) or Z.Z. (zzy20011126@mail.xjtu.edu.cn). To enable a complete run of the code shared in this study, a minimum amount of desensitized sample data is shared with the code. The source data underlying Figs. 1c, 2, 3, 4, 5, and 6b–d are provided as a Source Data file.

## Source data

Source Data File

## Code availability

Source code of this study is provided at https://github.com/LiuNingtao/ARDA.
