# Supplementary material for: Ageing and degeneration analysis using ageing-related dynamic attention on lateral cephalometric radiographs
Source: NPJ Digit Med. 2022 Sep 27;5:151. doi: 10.1038/s41746-022-00681-y (PMC9515216; doi:10.1038/s41746-022-00681-y)
Supplement: Supplementary file 1 — Supplementary Material [file 41746_2022_681_MOESM1_ESM.pdf]

Ageing and degeneration analysis using ageing-related dynamic attention on lateral cephalometric radiographs: Supplementary material

Supplementary Tables

Supplementary Table 1 The Pearson correlation coefficient (PCC) and p-value for testing non-correlation (P) between real ages and the ages predicted by the age estimation methods. PCC and P are used to evaluate the correlation between the actual age and predicted age of the samples in the dataset. BSL: baseline model, AC: ARDA-constrained, RTS: retest, TR: teeth region, CR: craniofacial region, SR: cervical spine region. In each age group, the highest Pearson correlation coefficient is bolded.

	**Age**	**4-10**	**11-15**	**16-20**	**22-25**	**26-30**	**31-35**	**36-40**	**4-25**	**26-40**	**ALL**
**PCC**	**BSL**	0.556	0.653	0.628	0.337	0.103	0.065	0.140	0.947	0.341	0.924
	**AC**	0.765	0.553	0.612	0.300	0.212	0.145	**0.387**	0.945	0.516	0.949
	**RTS**	0.858	**0.690**	**0.638**	**0.357**	0.334	0.033	0.249	**0.960**	0.458	**0.952**
	**TR**	0.869	0.652	0.531	0.355	0.330	0.083	0.359	0.948	**0.542**	0.948
	**CR**	0.842	0.616	0.541	0.217	0.286	0.046	0.243	0.936	0.389	0.931
	**SR**	0.794	0.659	0.513	0.291	**0.346**	0.053	0.277	0.941	0.535	0.945
	**TR+CR**	0.890	0.603	0.586	0.259	0.249	**0.285**	0.224	0.948	0.502	0.947
	**TR+SR**	**0.898**	0.676	0.563	0.340	0.291	0.070	0.341	0.951	0.517	0.949
	**CR+SR**	0.799	0.652	0.561	0.297	0.324	-0.043	0.241	0.943	0.499	0.943
**P**	**BSL**	0.000	0.000	0.000	0.000	0.155	0.580	0.402	0.000	0.000	0.000
	**AC**	0.000	0.000	0.000	0.000	0.003	0.235	0.038	0.000	0.000	0.000
	**RTS**	0.000	0.000	0.000	0.000	0.000	0.778	0.162	0.000	0.000	0.000
	**TR**	0.000	0.000	0.000	0.000	0.000	0.479	0.040	0.000	0.000	0.000
	**CR**	0.000	0.000	0.000	0.000	0.000	0.694	0.174	0.000	0.000	0.000
	**SR**	0.000	0.000	0.000	0.000	0.000	0.653	0.125	0.000	0.000	0.000
	**TR+CR**	0.000	0.000	0.000	0.000	0.001	0.018	0.243	0.000	0.000	0.000
	**TR+SR**	0.000	0.000	0.000	0.000	0.000	0.553	0.052	0.000	0.000	0.000
	**CR+SR**	0.000	0.000	0.000	0.000	0.000	0.715	0.176	0.000	0.000	0.000

Supplementary Table 2 The p-values of the f-test between different age estimations methods. BSL: baseline model, AC: ARDA-constrained, RTS: retest, TR: teeth region, CR: craniofacial region, SR: cervical spine region in different age groups. a 4-10 age group, b 11-15 age group, c 16-20 age group, d 21-25 age group, e 26-30 age group, f 31-35 age group, g 36-40 age group, h 4-25 age group, i 26-40 age group, j all the samples.

a

	BSL	AC	RTS	TR	CR	SR	TR+CR	TR+SR	CR+SR
BSL	0.50								
AC	0.00	0.50							
RTS	0.00	0.00	0.50						
TR	0.00	0.00	0.00	0.50					
CR	0.00	0.00	0.00	0.09	0.50				
SR	0.00	0.00	0.00	0.00	0.00	0.50			
TR+CR	0.00	0.00	0.50	0.00	0.00	0.00	0.50		
TR+SR	0.00	0.00	0.00	0.00	0.00	0.00	0.00	0.50	
CR+SR	0.00	0.01	0.00	0.00	0.00	0.01	0.00	0.00	0.50

b

	BSL	AC	RTS	TR	CR	SR	TR+CR	TR+SR	CR+SR
BSL	0.50								
AC	0.00	0.50							
RTS	0.00	0.00	0.50						
TR	0.00	0.00	0.00	0.50					
CR	0.00	0.00	0.00	0.11	0.50				
SR	0.00	0.00	0.00	0.50	0.11	0.50			
TR+CR	0.00	0.00	0.00	0.00	0.00	0.00	0.50		
TR+SR	0.00	0.00	0.50	0.00	0.00	0.00	0.00	0.50	
CR+SR	0.00	0.00	0.00	0.00	0.00	0.00	0.00	0.00	0.50

c

	BSL	AC	RTS	TR	CR	SR	TR+CR	TR+SR	CR+SR
BSL	0.50								
AC	0.01	0.50							
RTS	0.00	0.02	0.50						
TR	0.00	0.00	0.00	0.50					
CR	0.00	0.00	0.00	0.00	0.50				
SR	0.00	0.00	0.00	0.00	0.00	0.50			
TR+CR	0.00	0.00	0.00	0.29	0.00	0.00	0.50		
TR+SR	0.03	0.26	0.00	0.00	0.00	0.00	0.00	0.50	
CR+SR	0.00	0.00	0.00	0.00	0.02	0.00	0.00	0.00	0.50

d

	BSL	AC	RTS	TR	CR	SR	TR+CR	TR+SR	CR+SR
BSL	0.50								
AC	0.00	0.50							
RTS	0.02	0.00	0.50						
TR	0.00	0.01	0.00	0.50					
CR	0.00	0.00	0.00	0.00	0.50				
SR	0.00	0.14	0.00	0.00	0.00	0.50			
TR+CR	0.00	0.00	0.00	0.06	0.00	0.00	0.50		
TR+SR	0.00	0.36	0.00	0.03	0.00	0.07	0.00	0.50	
CR+SR	0.00	0.14	0.00	0.00	0.00	0.50	0.00	0.07	0.50

e

	BSL	AC	RTS	TR	CR	SR	TR+CR	TR+SR	CR+SR
BSL	0.50								
AC	0.00	0.50							
RTS	0.00	0.01	0.50						
TR	0.00	0.00	0.00	0.50					
CR	0.00	0.00	0.02	0.01	0.50				
SR	0.00	0.00	0.01	0.03	0.35	0.50			
TR+CR	0.00	0.01	0.50	0.00	0.02	0.01	0.50		
TR+SR	0.00	0.00	0.00	0.43	0.01	0.02	0.00	0.50	
CR+SR	0.00	0.00	0.04	0.00	0.35	0.22	0.04	0.00	0.50

f

	BSL	AC	RTS	TR	CR	SR	TR+CR	TR+SR	CR+SR
BSL	0.50								
AC	0.00	0.50							
RTS	0.00	0.00	0.50						
TR	0.00	0.00	0.19	0.50					
CR	0.00	0.00	0.18	0.04	0.50				
SR	0.00	0.00	0.23	0.45	0.05	0.50			
TR+CR	0.00	0.00	0.00	0.01	0.00	0.01	0.50		
TR+SR	0.00	0.00	0.50	0.19	0.18	0.23	0.00	0.50	
CR+SR	0.04	0.00	0.02	0.00	0.12	0.00	0.00	0.02	0.50

g

	BSL	AC	RTS	TR	CR	SR	TR+CR	TR+SR	CR+SR
BSL	0.50								
AC	0.00	0.50							
RTS	0.00	0.00	0.50						
TR	0.00	0.00	0.30	0.50					
CR	0.00	0.01	0.09	0.21	0.50				
SR	0.00	0.01	0.07	0.17	0.45	0.50			
TR+CR	0.00	0.49	0.00	0.00	0.01	0.01	0.50		
TR+SR	0.00	0.00	0.12	0.26	0.43	0.38	0.00	0.50	
CR+SR	0.00	0.00	0.24	0.43	0.26	0.22	0.00	0.32	0.50

h

	BSL	AC	RTS	TR	CR	SR	TR+CR	TR+SR	CR+SR
BSL	0.50								
AC	0.00	0.50							
RTS	0.00	0.00	0.50						
TR	0.00	0.01	0.00	0.50					
CR	0.14	0.00	0.00	0.00	0.50				
SR	0.01	0.00	0.00	0.00	0.00	0.50			
TR+CR	0.00	0.01	0.00	0.00	0.00	0.00	0.50		
TR+SR	0.00	0.00	0.00	0.00	0.00	0.00	0.00	0.50	
CR+SR	0.00	0.00	0.00	0.00	0.00	0.00	0.00	0.00	0.50

i

	BSL	AC	RTS	TR	CR	SR	TR+CR	TR+SR	CR+SR
BSL	0.50								
AC	0.00	0.50							
RTS	0.00	0.00	0.50						
TR	0.00	0.01	0.00	0.50					
CR	0.14	0.00	0.00	0.00	0.50				
SR	0.01	0.00	0.00	0.00	0.00	0.50			
TR+CR	0.00	0.01	0.00	0.00	0.00	0.00	0.50		
TR+SR	0.00	0.00	0.00	0.00	0.00	0.00	0.00	0.50	
CR+SR	0.00	0.00	0.00	0.00	0.00	0.00	0.00	0.00	0.50

j

	BSL	AC	RTS	TR	CR	SR	TR+CR	TR+SR	CR+SR
BSL	0.50								
AC	0.00	0.50							
RTS	0.00	0.00	0.50						
TR	0.00	0.00	0.03	0.50					
CR	0.00	0.00	0.00	0.00	0.50				
SR	0.00	0.01	0.01	0.27	0.00	0.50			
TR+CR	0.00	0.01	0.00	0.22	0.00	0.44	0.50		
TR+SR	0.00	0.00	0.15	0.22	0.00	0.08	0.06	0.50	
CR+SR	0.00	0.00	0.44	0.05	0.00	0.01	0.01	0.18	0.50

Supplementary Table 3 The p-values of the t-test between different age estimations methods. BSL: baseline model, AC: ARDA-constrained, RTS: retest, TR: teeth region, CR: craniofacial region, SR: cervical spine region in different age groups. a 4-10 age group, b 11-15 age group, c 16-20 age group, d 21-25 age group, e 26-30 age group, f 31-35 age group, g 36-40 age group, h 4-25 age group, i 26-40 age group, j all the samples.

a

	BSL	AC	RTS	TR	CR	SR	TR+CR	TR+SR	CR+SR
BSL	1.00								
AC	0.00	1.00							
RTS	0.05	0.00	1.00						
TR	0.00	0.00	0.05	1.00					
CR	0.81	0.00	0.00	0.00	1.00				
SR	0.06	0.00	0.00	0.00	0.00	1.00			
TR+CR	0.00	0.00	0.00	0.01	0.00	0.00	1.00		
TR+SR	0.00	0.00	0.00	0.00	0.00	0.00	0.10	1.00	
CR+SR	0.23	0.00	0.00	0.00	0.08	0.23	0.00	0.00	1.00

b

	BSL	AC	RTS	TR	CR	SR	TR+CR	TR+SR	CR+SR
BSL	1.00								
AC	0.00	1.00							
RTS	0.07	0.00	1.00						
TR	0.00	0.00	0.00	1.00					
CR	0.00	0.00	0.00	0.00	1.00				
SR	0.00	0.00	0.00	1.00	0.00	1.00			
TR+CR	0.00	0.00	0.00	0.00	0.00	0.00	1.00		
TR+SR	0.01	0.00	0.32	0.00	0.00	0.00	0.01	1.00	
CR+SR	0.00	0.00	0.00	0.38	0.00	0.38	0.00	0.00	1.00

c

	BSL	AC	RTS	TR	CR	SR	TR+CR	TR+SR	CR+SR
BSL	1.00								
AC	0.00	1.00							
RTS	0.17	0.00	1.00						
TR	0.00	0.20	0.00	1.00					
CR	0.00	0.00	0.00	0.00	1.00				
SR	0.00	0.00	0.00	0.00	0.08	1.00			
TR+CR	0.00	0.40	0.00	0.05	0.00	0.00	1.00		
TR+SR	0.00	0.00	0.00	0.09	0.00	0.00	0.00	1.00	
CR+SR	0.00	0.00	0.00	0.00	0.00	0.08	0.02	0.00	1.00

d

	BSL	AC	RTS	TR	CR	SR	TR+CR	TR+SR	CR+SR
BSL	1.00								
AC	0.00	1.00							
RTS	0.13	0.00	1.00						
TR	0.00	0.00	0.05	1.00					
CR	0.00	0.00	0.00	0.00	1.00				
SR	0.00	0.01	0.00	0.00	0.00	1.00			
TR+CR	0.00	0.43	0.00	0.00	0.00	0.04	1.00		
TR+SR	0.00	0.12	0.00	0.00	0.00	0.20	0.42	1.00	
CR+SR	0.00	0.45	0.00	0.00	0.00	0.04	1.00	0.44	1.00

e

	BSL	AC	RTS	TR	CR	SR	TR+CR	TR+SR	CR+SR
BSL	1.00								
AC	0.00	1.00							
RTS	0.01	0.19	1.00						
TR	0.58	0.00	0.00	1.00					
CR	0.19	0.00	0.00	0.01	1.00				
SR	0.00	0.89	0.27	0.00	0.00	1.00			
TR+CR	0.30	0.00	0.02	0.50	0.00	0.00	1.00		
TR+SR	0.71	0.00	0.00	0.21	0.19	0.00	0.05	1.00	
CR+SR	0.03	0.05	0.49	0.03	0.00	0.08	0.10	0.00	1.00

f

	BSL	AC	RTS	TR	CR	SR	TR+CR	TR+SR	CR+SR
BSL	1.00								
AC	0.92	1.00							
RTS	0.00	0.00	1.00						
TR	0.00	0.00	0.32	1.00					
CR	0.00	0.00	0.52	0.10	1.00				
SR	0.02	0.00	0.00	0.03	0.00	1.00			
TR+CR	0.08	0.02	0.00	0.00	0.00	0.51	1.00		
TR+SR	0.00	0.00	0.96	0.34	0.49	0.00	0.00	1.00	
CR+SR	0.00	0.00	0.69	0.17	0.83	0.00	0.00	0.65	1.00

g

	BSL	AC	RTS	TR	CR	SR	TR+CR	TR+SR	CR+SR
BSL	1.00								
AC	0.09	1.00							
RTS	0.14	0.64	1.00						
TR	0.00	0.03	0.00	1.00					
CR	0.92	0.01	0.02	0.00	1.00				
SR	0.00	0.00	0.00	0.12	0.00	1.00			
TR+CR	0.05	0.71	0.38	0.08	0.00	0.00	1.00		
TR+SR	0.00	0.01	0.00	0.48	0.00	0.39	0.02	1.00	
CR+SR	0.00	0.00	0.00	0.19	0.00	0.79	0.00	0.55	1.00

h

	BSL	AC	RTS	TR	CR	SR	TR+CR	TR+SR	CR+SR
BSL	1.00								
AC	0.00	1.00							
RTS	1.00	0.00	1.00						
TR	0.00	0.00	0.00	1.00					
CR	0.00	0.00	0.00	0.00	1.00				
SR	0.00	0.41	0.00	0.00	0.00	1.00			
TR+CR	0.00	0.00	0.00	0.39	0.00	0.00	1.00		
TR+SR	0.00	0.00	0.00	0.07	0.00	0.00	0.01	1.00	
CR+SR	0.00	0.00	0.00	0.00	0.00	0.02	0.00	0.00	1.00

i

	BSL	AC	RTS	TR	CR	SR	TR+CR	TR+SR	CR+SR
BSL	1.00								
AC	0.00	1.00							
RTS	0.63	0.00	1.00						
TR	0.42	0.00	0.67	1.00					
CR	0.01	0.00	0.00	0.00	1.00				
SR	0.00	0.50	0.00	0.00	0.00	1.00			
TR+CR	0.03	0.00	0.02	0.06	0.00	0.03	1.00		
TR+SR	0.87	0.00	0.67	0.39	0.00	0.00	0.01	1.00	
CR+SR	0.15	0.00	0.21	0.39	0.00	0.00	0.33	0.09	1.00

j

	BSL	AC	RTS	TR	CR	SR	TR+CR	TR+SR	CR+SR
BSL	1.00								
AC	0.00	1.00							
RTS	0.30	0.00	1.00						
TR	0.01	0.00	0.00	1.00					
CR	0.00	0.00	0.00	0.00	1.00				
SR	0.00	0.53	0.00	0.00	0.00	1.00			
TR+CR	0.01	0.00	0.00	1.00	0.00	0.00	1.00		
TR+SR	0.04	0.00	0.00	0.54	0.00	0.00	0.54	1.00	
CR+SR	0.00	1.00	0.00	0.00	0.00	0.55	0.00	0.00	1.00

Supplementary Figures

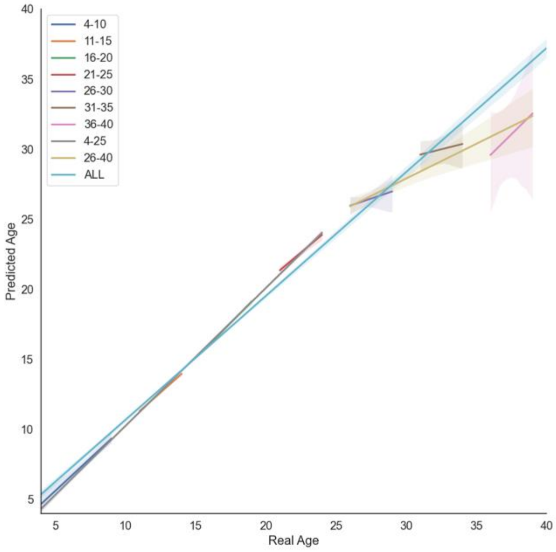

a

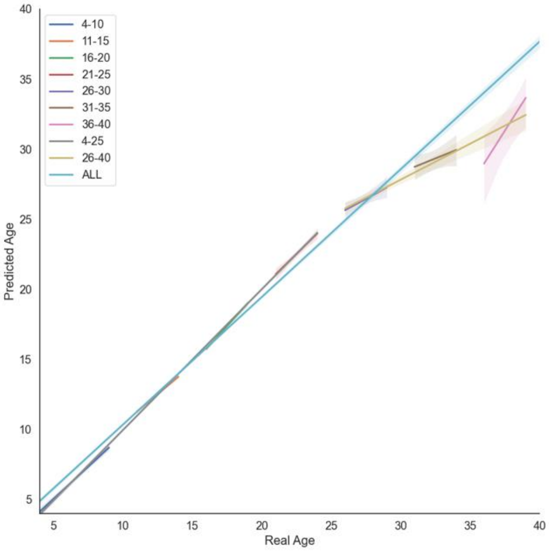

b

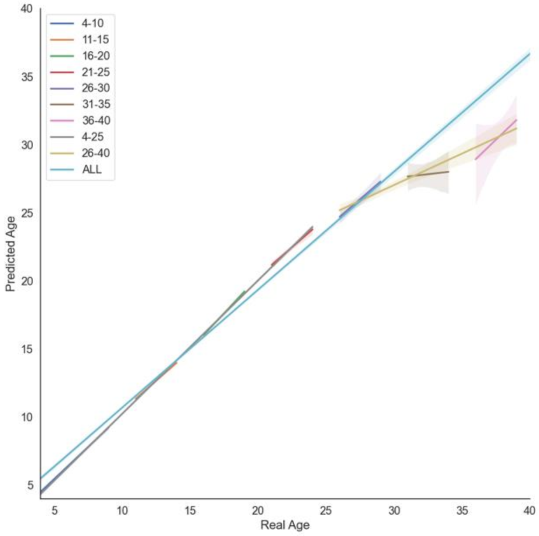

c

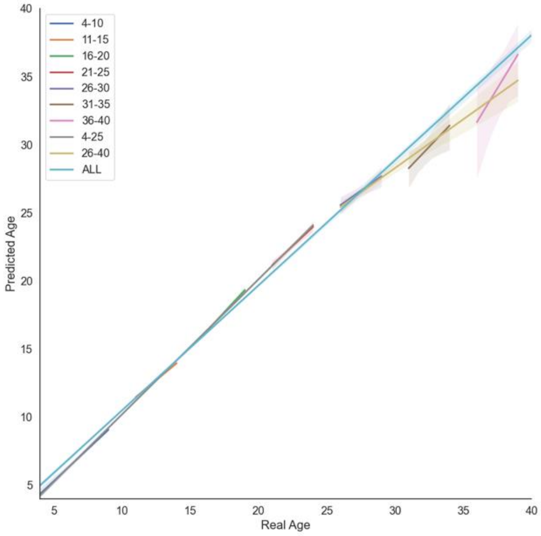

d

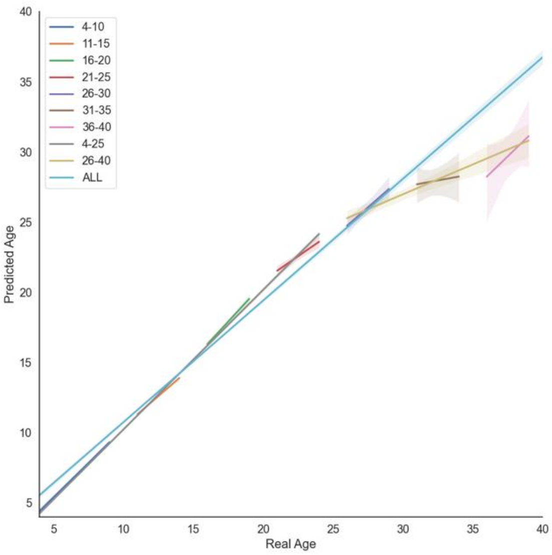
4-10
11-15
16-20
21-25
26-30
31-35
36-40
4-25
26-40
ALL
Predicted Age
Real Age

e

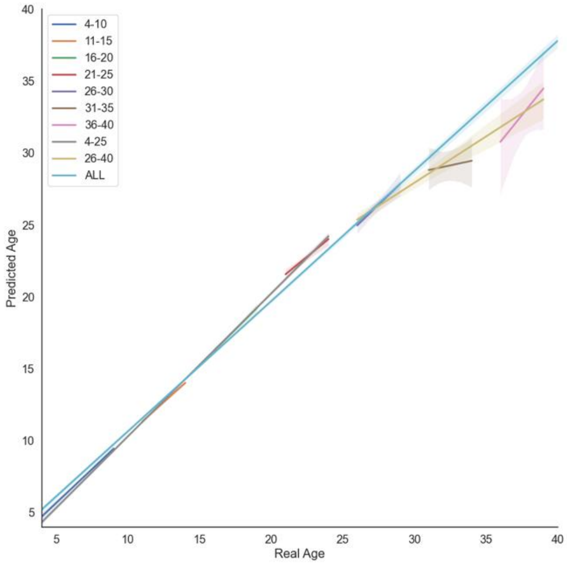
4-10
11-15
16-20
21-25
26-30
31-35
36-40
4-25
26-40
ALL
Predicted Age
Real Age

f

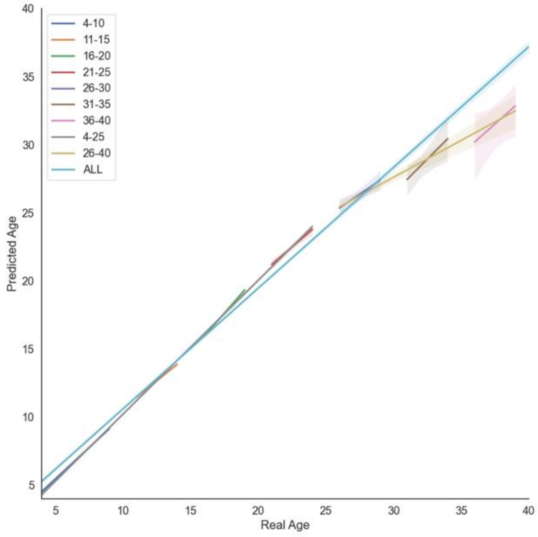
4-10
11-15
16-20
21-25
26-30
31-35
36-40
4-25
26-40
ALL
Predicted Age
Real Age

g

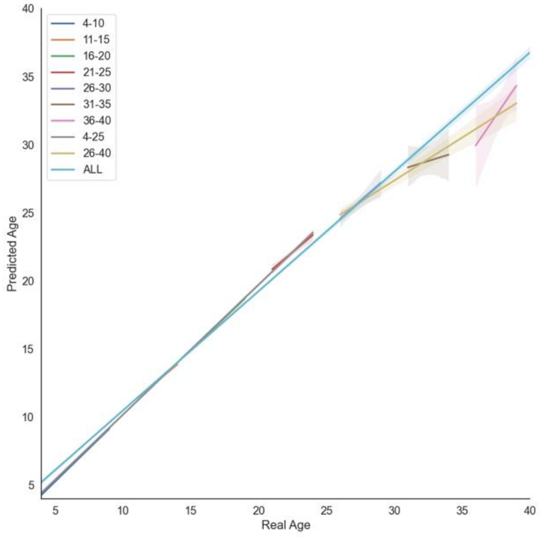
4-10
11-15
16-20
21-25
26-30
31-35
36-40
4-25
26-40
ALL
Predicted Age
Real Age

h

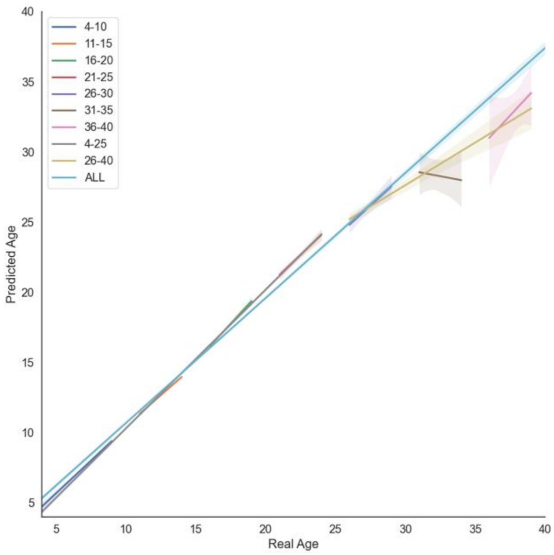

i

Supplementary Figure 1 The fitted regression models on the true age and the age predicted by different methods. a baseline model, b ARDA-constrained, c retest, d teeth region, e craniofacial region, f cervical spine region, g teeth region + craniofacial region, h teeth region + cervical spine region, i craniofacial region + cervical spine region.

Supplementary Notes

Supplementary Note 1

The Squeeze-and-Excitation (SE)[1] module is architectural unit embedded in MBCov[2][3], which is the main block of EfficentNet[4]. In EfficientNet, SE follows the feature map U with Cchannels and $H \times W$ spatial size ($U \in R^{C \times H \times W}$) obtained by convolution. Firstly, a statistic $z \in R^{c}$ is generated by computing the global average for each channel of U, where the $c - th$ element of z is calculated by:

$$z_c = \frac{1}{H \times W} \sum_{i=1}^{H} \sum_{j=1}^{W} u_c(i, j) \tag{1}$$

where $u_c(i, j)$ is the value of channel c and position (i, j) in the feature map U. Then the scale vector $s \in R^{C}$ is obtained by:

$$s = \sigma\left(W_2 \delta(W_1 z)\right) \tag{2}$$

where $\sigma(\cdot)$ and $\delta(\cdot)$ are the sigmoid and ReLU function, respectively, $W_1 \in R^{\frac{C}{r} \times C}$ and $W_2 \in R^{C \times \frac{C}{r}}$ are the learnable parameters. Finally, the feature map is scaled by s and fed into next convolution layer:

$$u'_c = s_c \cdot z_c \tag{3}$$

EfficientNet uses a compound coefficient ϕto uniformly scale the network width, depth, and resolution in a principled way:

$$\begin{gathered} depth: d = \alpha^{\phi} \\ width: w = \beta^{\phi} \\ resolution: r = \gamma^{\phi} \\ s.t. \alpha \cdot \beta^2 \cdot \gamma^2 \approx 2 \\ \alpha \geq 1, \beta \geq 1, \gamma \geq 1 \end{gathered} \tag{4}$$

where α, β, and γ are constants that can be determined by a small grid search. ϕ is a coefficient that describes how many more resources are available for model scaling, while α, β, and γ specify how to assign these extra resources to the width, depth, and resolution of the network respectively.

Supplementary Note 2

The LCR images at each age were split into training, validation and test datasets according to the ratio of 7: 1.5: 1.5, which allows the age distribution of LCR images in each dataset to be consistent with that of the overall dataset.

The weights of the model were randomly initialized with a normal distribution. In this study, the mini-batch strategy was used to train the model, i.e., in each training iteration, batch-size samples are fed into the model. The optimizer used in our study is Adam, and its weight decay was set to 0.0001.

To prevent the model from converging to the local optimum and speed up the convergence, the learning rate also decreased with the number of times to traverse the training dataset (epoch). When training the network, if the performance on the validation dataset did not improve for 3 consecutive

epochs or the epoch reaches the max value, which is set at 100 in this study, the training stops. After training, we selected the parameters of the network that performed best on the validation dataset and obtained final performances using the test.

Suppose the initial learning rate is η, we get the learning rate of $epoch\ i$:

$$\eta_i = \eta \times 0.8^{\lfloor epoch/4 \rfloor}, \tag{5}$$

where the initial learning rate $\eta = 0.001$. The parameters were updated according to its gradient and current training iteration learning rate:

$$w_i = w_{i-1} - \eta_i \nabla_{w_{i-1}}, \tag{6}$$

where w_i is the model parameters after i $epochs$. ∇_{w_i} is the gradient of the parameters obtained by the back propagation algorithm. The loss function used for training the model was $L1$ loss:

$$L = \frac{1}{N}\sum_{n=1}^{N}\left|\hat{y}_n - y_n\right|, \tag{7}$$

where N is the number of samples, y_n and $\hat{y}_n$ are the age and predicted age of nth LCR image.

Supplementary Note 3

The baseline model we used in this study requires the size of the image must be fixed, since the feature map is flattened into a one-dimensional vector before being input to the fixed-size fully connected layer. Meanwhile, considering the preservation of the texture and structure information in the image and the efficiency of the model, the LCR images and the ARDA concentrated ageing region were resized to 1000×1000 and 500×500 respectively. However, the aspect ratios of the LCR image and the ARDA concentrated ageing are not 1:1, thus the direct resize operation will inevitably cause image distortion. Therefore, before resizing the short sides of LCR images and the ARDA concentrated ageing regions were padded to the length of their long sides so that the aspect ratios of the images were always 1:1.

Supplementary Note 4

Every LCR image and ARDA concentrated ageing region were also been augmented to increase the diversity of the samples, thereby improving models' generalization performance. The samples in the training dataset were augmented by Random Affine, Horizontal Flip, and Vertical Flip operations provided in PyTorch. Before the data augmentation, the contrast of the input image is enhanced by contrast limited adaptive histogram equalization. The source code of the Contrast Enhancement and Shape Fixing is provided in source code repository (https://github.com/LiuNingtao/ARDA/blob/master/consum_transformer.py).

To eliminate the effect of size differences on the quantified ARDA analysis, we only calculate the mean ARDA of the ageing-significant region within each instance. For a specific age a:

$$Q_s = \frac{1}{P_s}\sum_{i}^{\mathrm{P}} \mathbb{I}(p_{\mathrm{i}} \geq T_a)p_{\mathrm{i}} \tag{8}$$

where Q_s is the quantified ARDA of instance s, P is the number of elements in the instance, P_s is the number of elements with average ageing salience greater than or equal to the ageing-significant region threshold T_a of age a, and $\mathbb{I}(\cdot)$ is the indicator function to determine whether the element with average ageing salience p_i in ARDA belong to an ageing-significant region. In this study, the

ageing-significant region threshold was set to the median value, the 75th quantile and the 90th quantile of the average ageing salience corresponding to a specific age.

Supplementary References

[1] Hu, Jie, Li Shen, and Gang Sun. "Squeeze-and-excitation networks." Proceedings of the IEEE conference on computer vision and pattern recognition. 2018.

[2] Sandler, Mark, et al. "Mobilenetv2: Inverted residuals and linear bottlenecks." Proceedings of the IEEE conference on computer vision and pattern recognition. 2018.

[3] Tan, Mingxing, et al. "Mnasnet: Platform-aware neural architecture search for mobile." Proceedings of the IEEE/CVF Conference on Computer Vision and Pattern Recognition. 2019.

[4] Tan, Mingxing, and Quoc Le. "Efficientnet: Rethinking model scaling for convolutional neural networks." International conference on machine learning. PMLR, 2019.
