# Supplementary material for: Ageing and degeneration analysis using ageing-related dynamic attention on lateral cephalometric radiographs
Source: NPJ Digit Med. 2022 Sep 27;5:151. doi: 10.1038/s41746-022-00681-y (PMC9515216; doi:10.1038/s41746-022-00681-y)
Supplement: Supplementary file 2 — Reporting Summary [file 41746_2022_681_MOESM2_ESM.pdf]

nature portfolio

Corresponding author(s): Ningtao Liu
Last updated by author(s): Jun 13, 2022

Reporting Summary

Nature Portfolio wishes to improve the reproducibility of the work that we publish. This form provides structure for consistency and transparency in reporting. For further information on Nature Portfolio policies, see our Editorial Policies and the Editorial Policy Checklist.

Statistics

For all statistical analyses, confirm that the following items are present in the figure legend, table legend, main text, or Methods section.

n/a	Confirmed	
☐	☒	The exact sample size (*n*) for each experimental group/condition, given as a discrete number and unit of measurement
☐	☒	A statement on whether measurements were taken from distinct samples or whether the same sample was measured repeatedly
☐	☒	The statistical test(s) used AND whether they are one- or two-sided<br>*Only common tests should be described solely by name; describe more complex techniques in the Methods section.*
☐	☒	A description of all covariates tested
☐	☒	A description of any assumptions or corrections, such as tests of normality and adjustment for multiple comparisons
☐	☒	A full description of the statistical parameters including central tendency (e.g. means) or other basic estimates (e.g. regression coefficient) AND variation (e.g. standard deviation) or associated estimates of uncertainty (e.g. confidence intervals)
☐	☒	For null hypothesis testing, the test statistic (e.g. *F*, *t*, *r*) with confidence intervals, effect sizes, degrees of freedom and *P* value noted<br>*Give P values as exact values whenever suitable.*
☒	☐	For Bayesian analysis, information on the choice of priors and Markov chain Monte Carlo settings
☒	☐	For hierarchical and complex designs, identification of the appropriate level for tests and full reporting of outcomes
☐	☒	Estimates of effect sizes (e.g. Cohen's *d*, Pearson's *r*), indicating how they were calculated

Our web collection on statistics for biologists contains articles on many of the points above.

Software and code

Policy information about availability of computer code

Data collection	The Python libraries of Pydicom, SimpleITK, and nibable were used for reading data and the meta information. The ageing salience and ageing salience map were generated by open source library provided in https://github.com/jacobgil/pytorch-grad-cam. The ageing feature extractor is based on open source implementation of Efficient provided in https://github.com/lukemelas/EfficientNet-PyTorch.
Data analysis	The code for data analysis is shown in https://github.com/LiuNingtao/ARDA

For manuscripts utilizing custom algorithms or software that are central to the research but not yet described in published literature, software must be made available to editors and reviewers. We strongly encourage code deposition in a community repository (e.g. GitHub). See the Nature Portfolio guidelines for submitting code & software for further information.

Data

Policy information about availability of data

All manuscripts must include a data availability statement. This statement should provide the following information, where applicable:

- Accession codes, unique identifiers, or web links for publicly available datasets
- A description of any restrictions on data availability
- For clinical datasets or third party data, please ensure that the statement adheres to our policy

The data used in this study is not open access due to privacy and security concerns. After obtaining the sharing agreement, it can be shared with third parties for

reasonable use, relevant requests should be addressed to C.Y. (yanchunxia@mail.xjtu.edu.cn) or Z.Z. (zzy20011126@mail.xjtu.edu.cn). To enable a complete run of the code shared in this study, a minimum amount of desensitized sample data is shared with the code.

Human research participants

Policy information about studies involving human research participants and Sex and Gender in Research.

Reporting on sex and gender	Of the 20,174 samples used in this study, 7,302 were from males and 12,872 were from females. Sex information was not included in the study because we wanted to obtain generic ageing characteristics independent of sex.
Population characteristics	The inclusion criteria for the studied subjects in the study is: LCR images of orthodontic patients aged 4 to 40 years. The age distribution of the dataset is shown in the Table 4 of manuscript.
Recruitment	The data used for the study were cephalometric lateral views of orthodontic patients aged 4 to 40 years from Stomatological Hospital of Xi'an Jiaotong University Health Science Center. No other specific selection criteria were included.
Ethics oversight	This study is approved and supervised by the Affiliated Stomatological Hospital of Xi'an Jiaotong University Health Science Center (Approval number: xjkqll[2022]NO.30)

Note that full information on the approval of the study protocol must also be provided in the manuscript.

Field-specific reporting

Please select the one below that is the best fit for your research. If you are not sure, read the appropriate sections before making your selection.

☒ Life sciences ☐ Behavioural & social sciences ☐ Ecological, evolutionary & environmental sciences

For a reference copy of the document with all sections, see nature.com/documents/nr-reporting-summary-flat.pdf

Life sciences study design

All studies must disclose on these points even when the disclosure is negative.

Sample size	12872
Data exclusions	The image with actual age of the subject is less than 4 years or greater than 40 years, restorations, or incorrect imaging posture, and incomplete LCR images were excluded in this study.
Replication	The mean absolute error can be used to measure the performance of age estimation, and the weight parameters are also shared. Any investigator interested in this study can reproduce this study using similar data.
Randomization	A randomly sampled dataset was used to save training time when choosing the baseline model. The sampled dataset is consistent with the age distribution of the full dataset.
Blinding	Data collection was blinding to participants in this study.

Reporting for specific materials, systems and methods

We require information from authors about some types of materials, experimental systems and methods used in many studies. Here, indicate whether each material, system or method listed is relevant to your study. If you are not sure if a list item applies to your research, read the appropriate section before selecting a response.

Materials & experimental systems

n/a	Involved in the study	
☒	☐	Antibodies
☒	☐	Eukaryotic cell lines
☒	☐	Palaeontology and archaeology
☒	☐	Animals and other organisms
☒	☐	Clinical data
☒	☐	Dual use research of concern

Methods

n/a	Involved in the study	
☒	☐	ChIP-seq
☒	☐	Flow cytometry
☒	☐	MRI-based neuroimaging
