# Supplementary figures and images for: Ageing and degeneration analysis using ageing-related dynamic attention on lateral cephalometric radiographs (part 2 of 2)
Source: NPJ Digit Med. 2022 Sep 27;5:151. doi: 10.1038/s41746-022-00681-y (PMC9515216; doi:10.1038/s41746-022-00681-y)

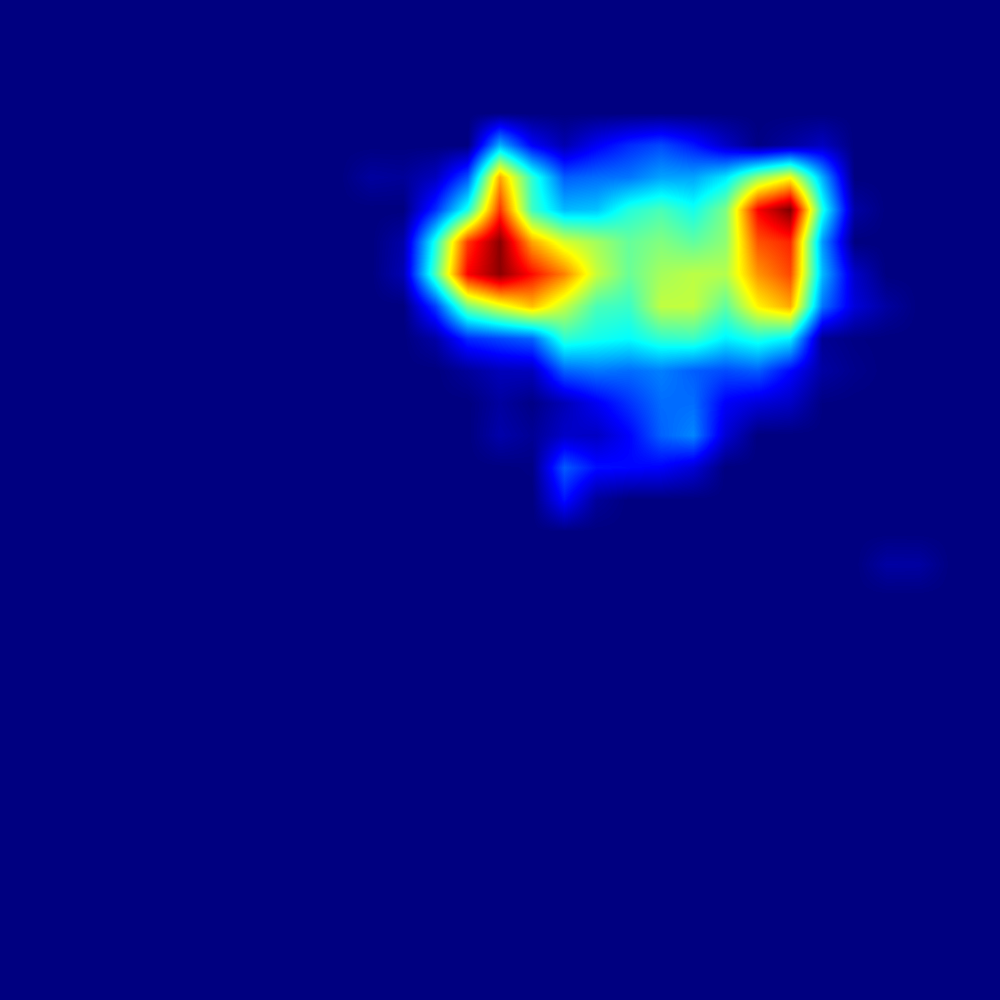

Supplement: Supplementary file 3 — Source Data File [file 41746_2022_681_MOESM3_ESM.zip › ARDA Map/Figure 3/40.png]

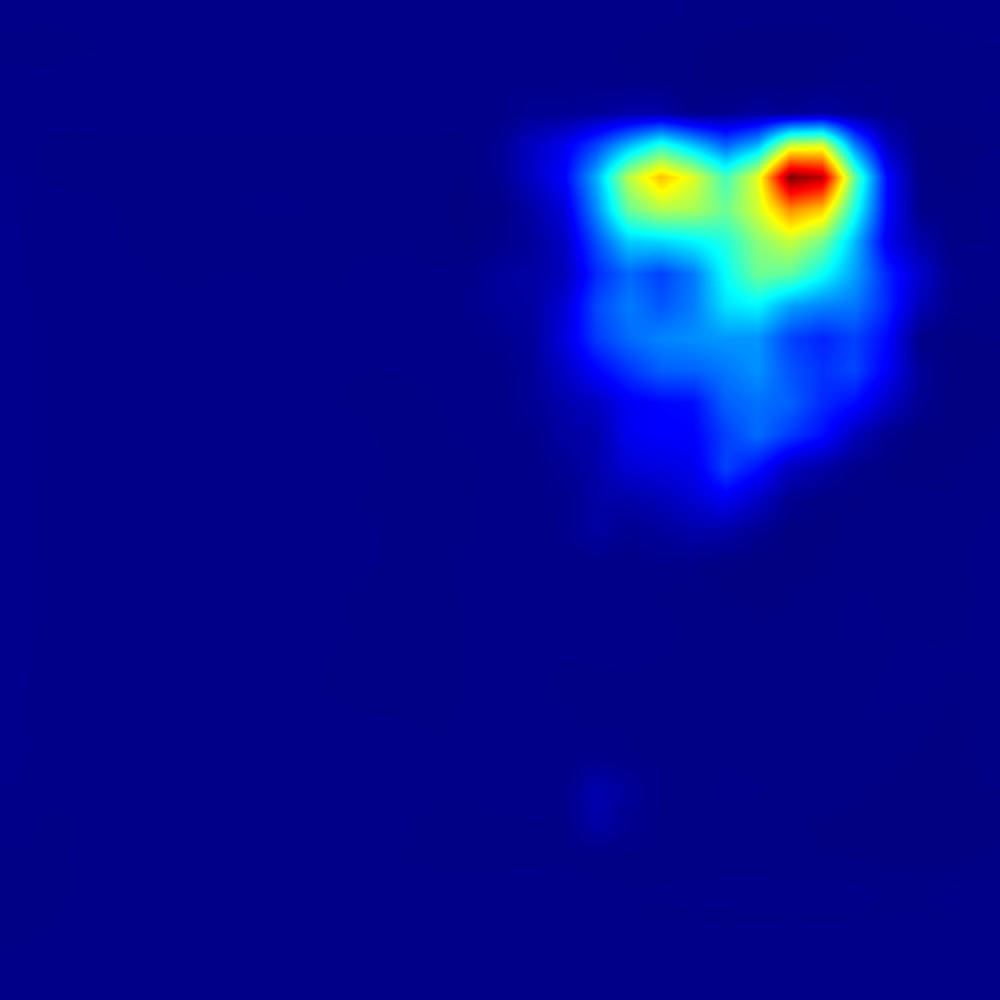

Supplement: Supplementary file 3 — Source Data File [file 41746_2022_681_MOESM3_ESM.zip › ARDA Map/Figure 3/5.png]

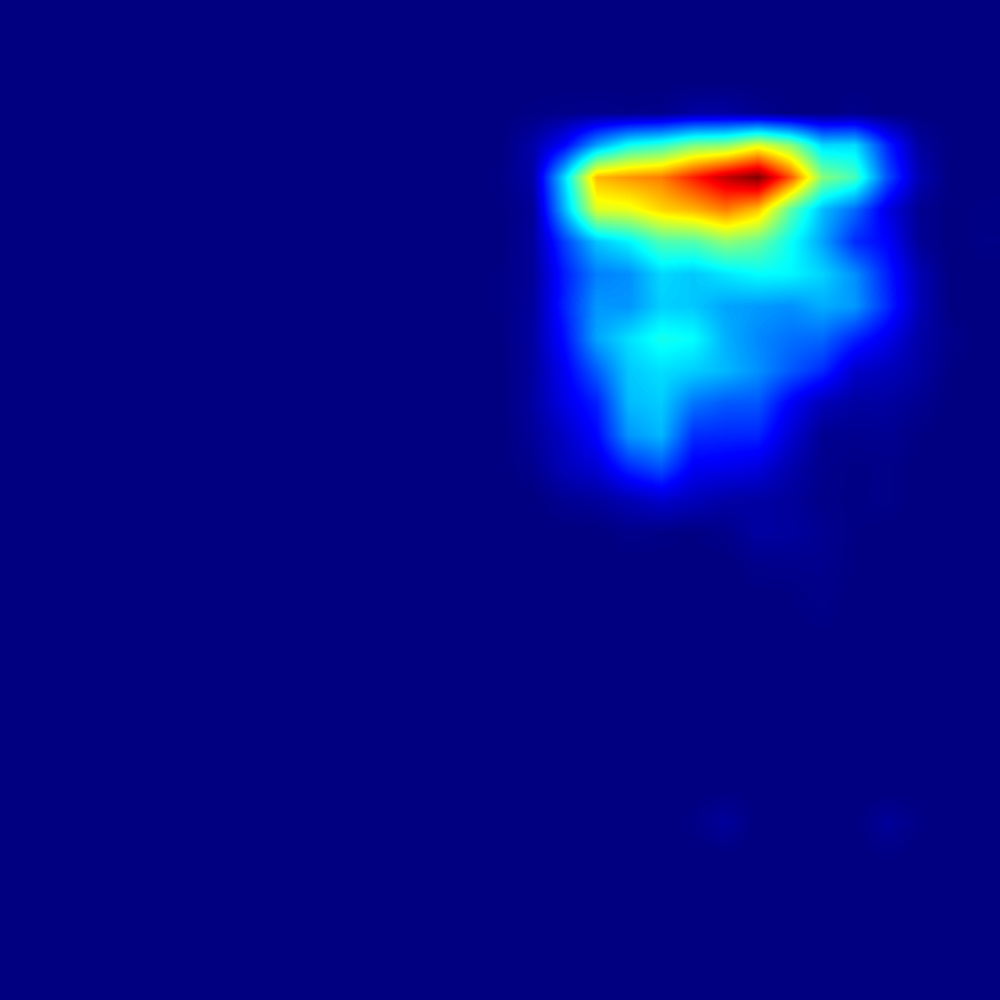

Supplement: Supplementary file 3 — Source Data File [file 41746_2022_681_MOESM3_ESM.zip › ARDA Map/Figure 3/6.png]

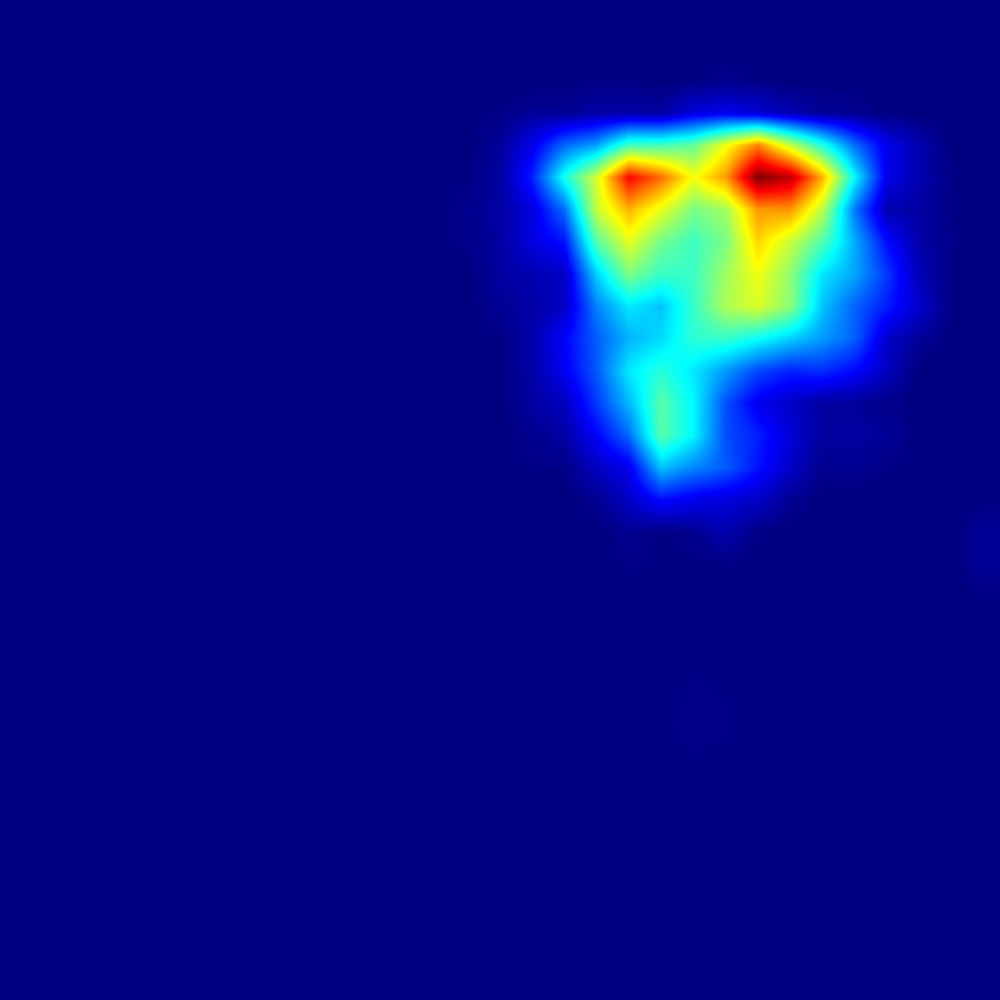

Supplement: Supplementary file 3 — Source Data File [file 41746_2022_681_MOESM3_ESM.zip › ARDA Map/Figure 3/7.png]

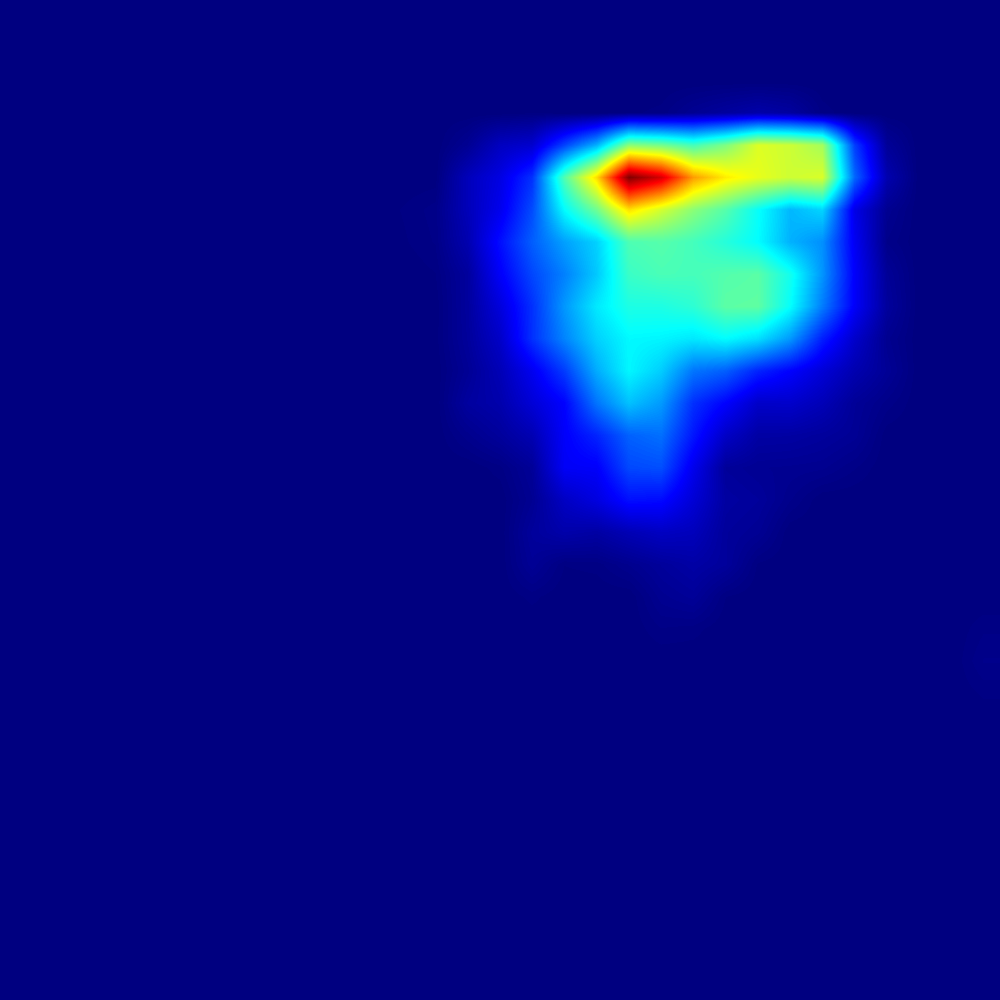

Supplement: Supplementary file 3 — Source Data File [file 41746_2022_681_MOESM3_ESM.zip › ARDA Map/Figure 3/8.png]

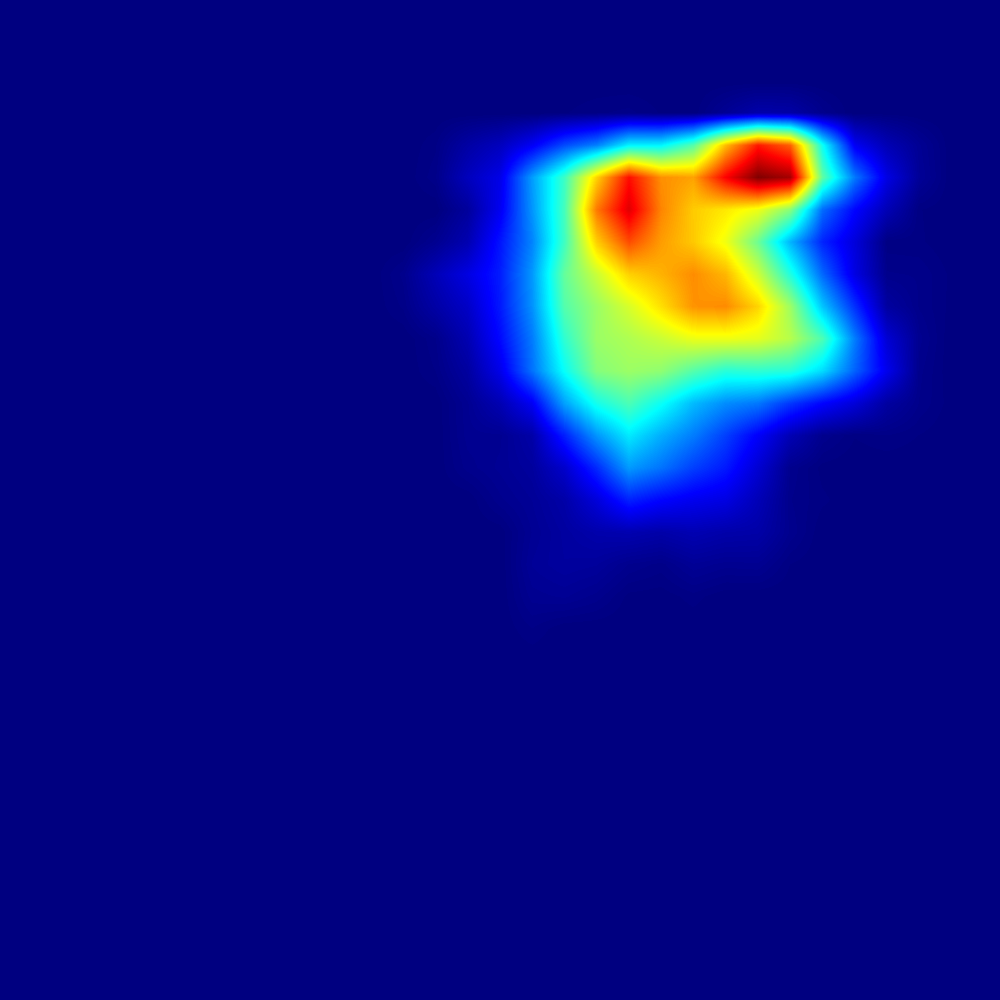

Supplement: Supplementary file 3 — Source Data File [file 41746_2022_681_MOESM3_ESM.zip › ARDA Map/Figure 3/9.png]

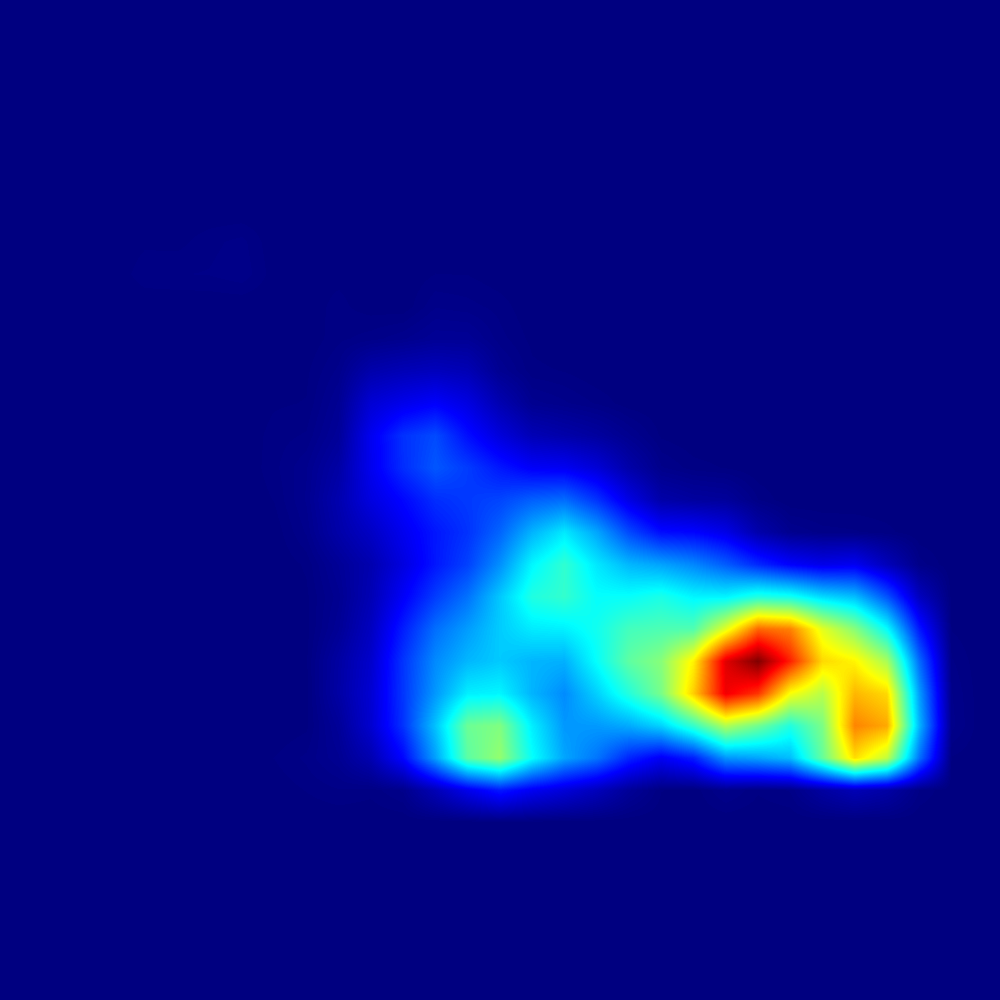

Supplement: Supplementary file 3 — Source Data File [file 41746_2022_681_MOESM3_ESM.zip › ARDA Map/Figure 4/10.png]

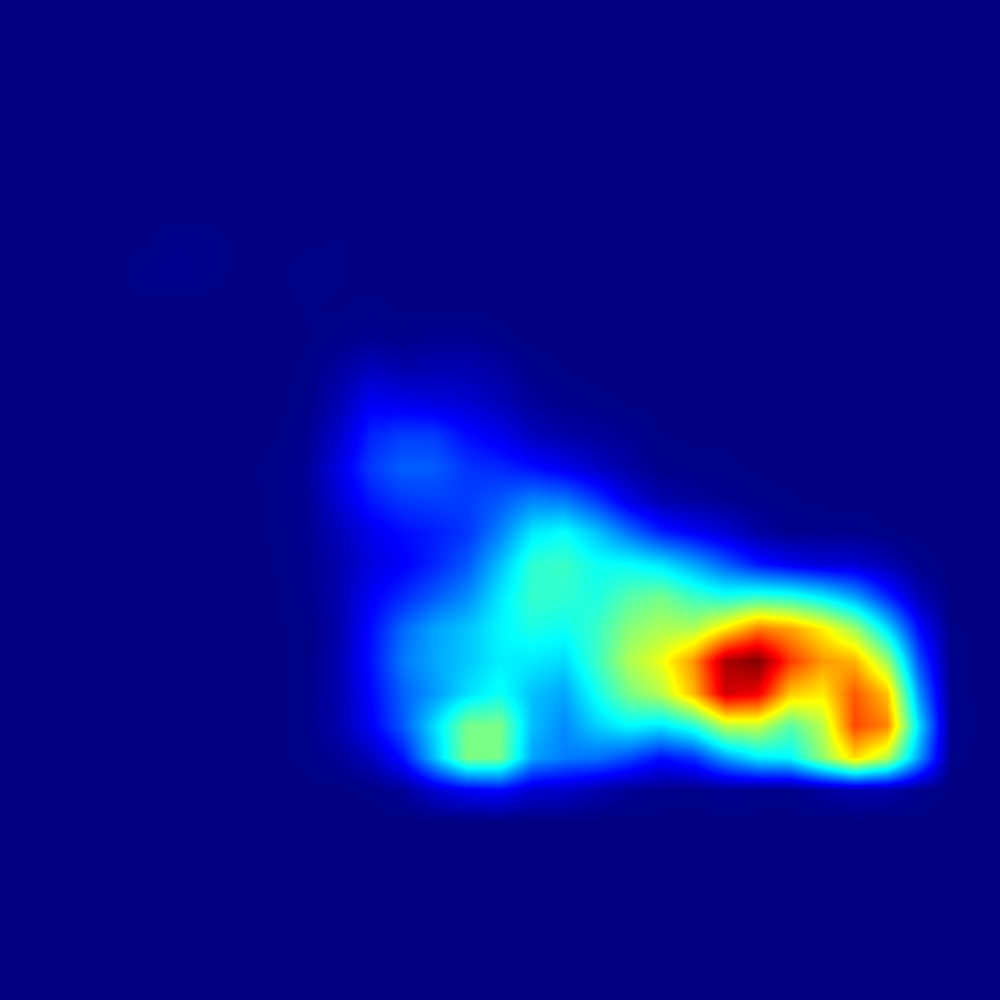

Supplement: Supplementary file 3 — Source Data File [file 41746_2022_681_MOESM3_ESM.zip › ARDA Map/Figure 4/11.png]

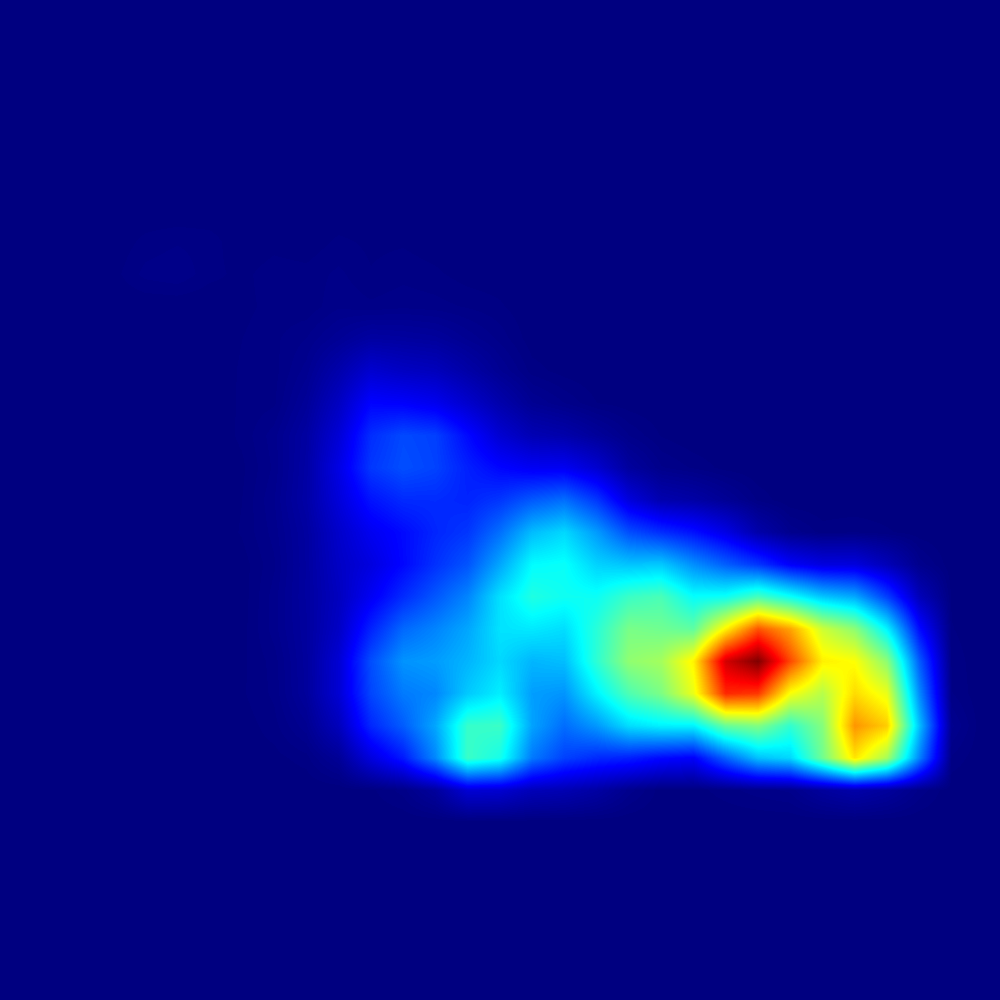

Supplement: Supplementary file 3 — Source Data File [file 41746_2022_681_MOESM3_ESM.zip › ARDA Map/Figure 4/12.png]

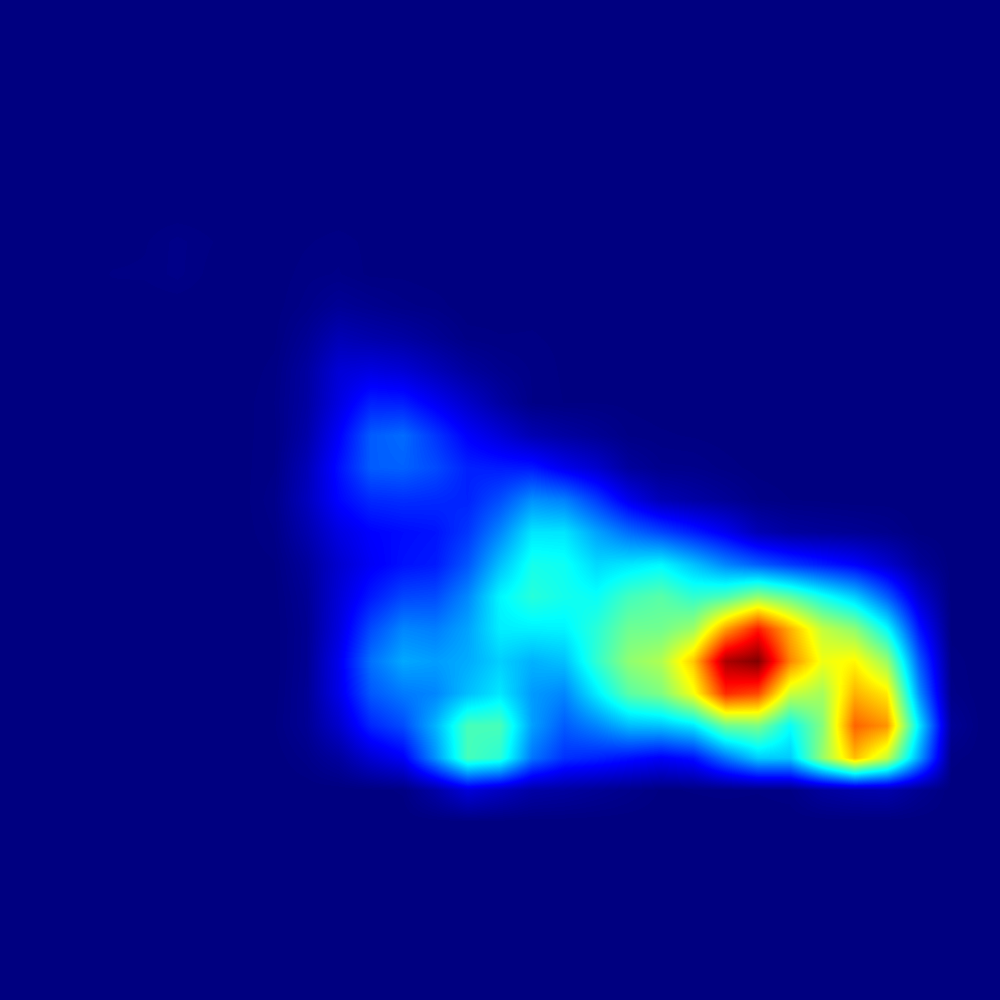

Supplement: Supplementary file 3 — Source Data File [file 41746_2022_681_MOESM3_ESM.zip › ARDA Map/Figure 4/13.png]

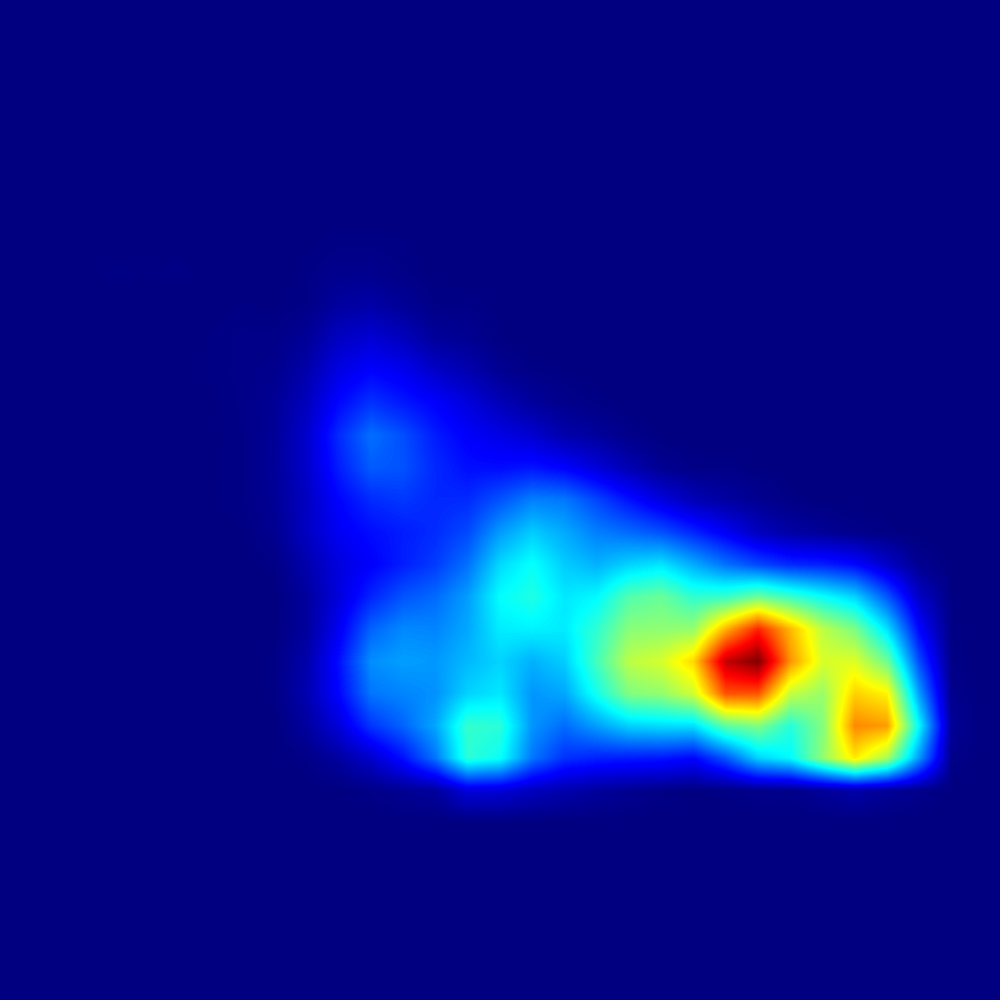

Supplement: Supplementary file 3 — Source Data File [file 41746_2022_681_MOESM3_ESM.zip › ARDA Map/Figure 4/14.png]

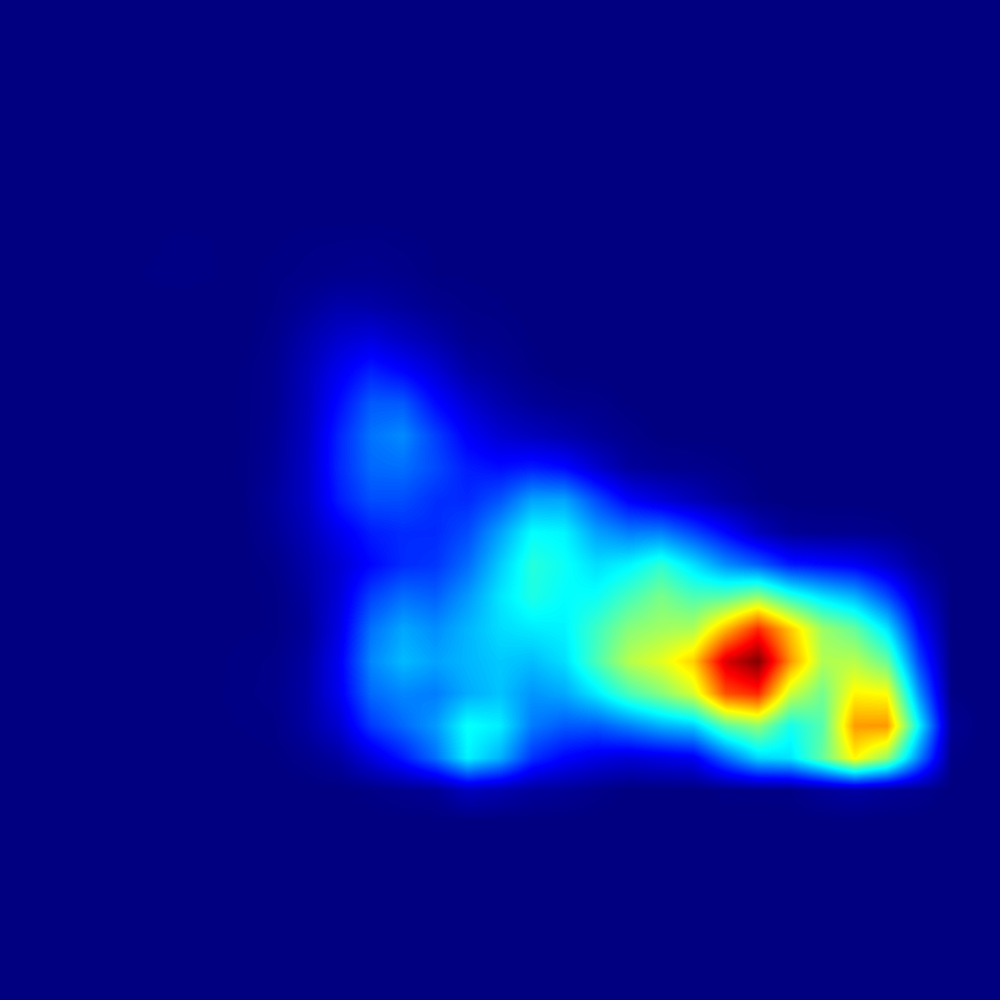

Supplement: Supplementary file 3 — Source Data File [file 41746_2022_681_MOESM3_ESM.zip › ARDA Map/Figure 4/15.png]

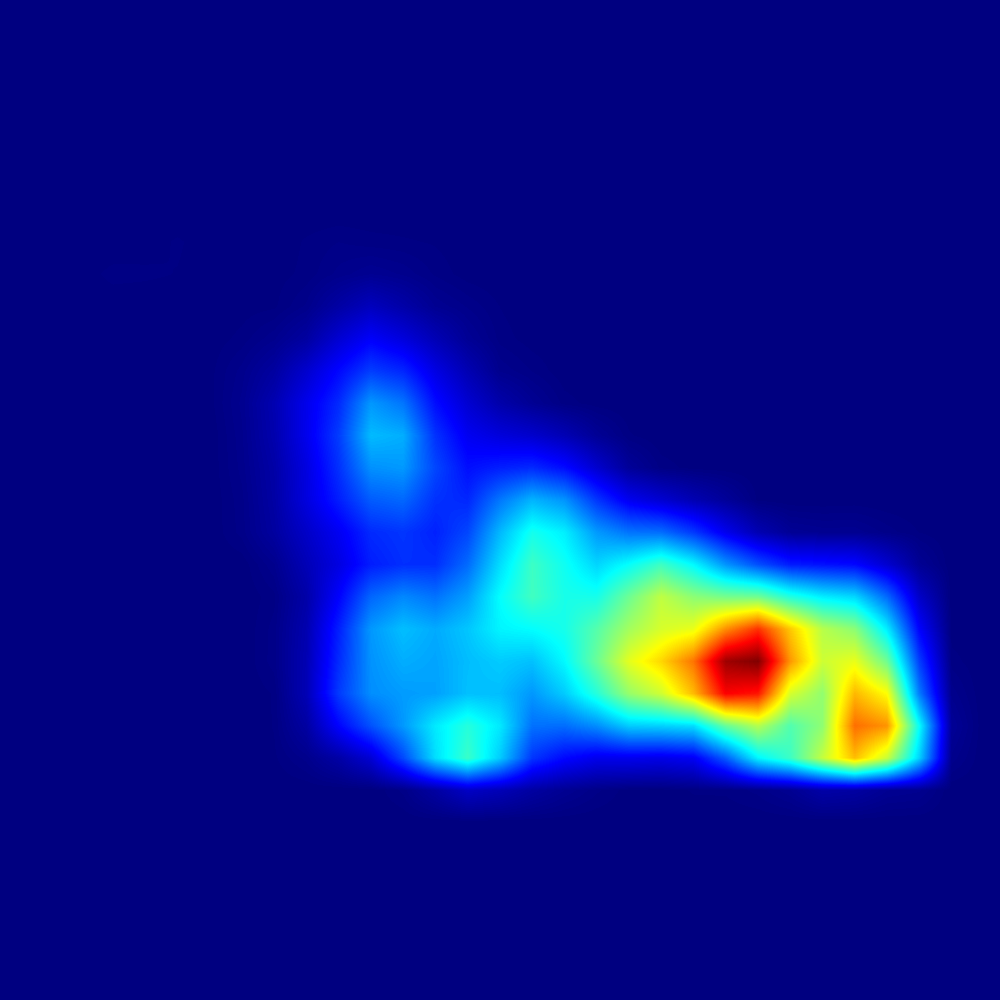

Supplement: Supplementary file 3 — Source Data File [file 41746_2022_681_MOESM3_ESM.zip › ARDA Map/Figure 4/16.png]

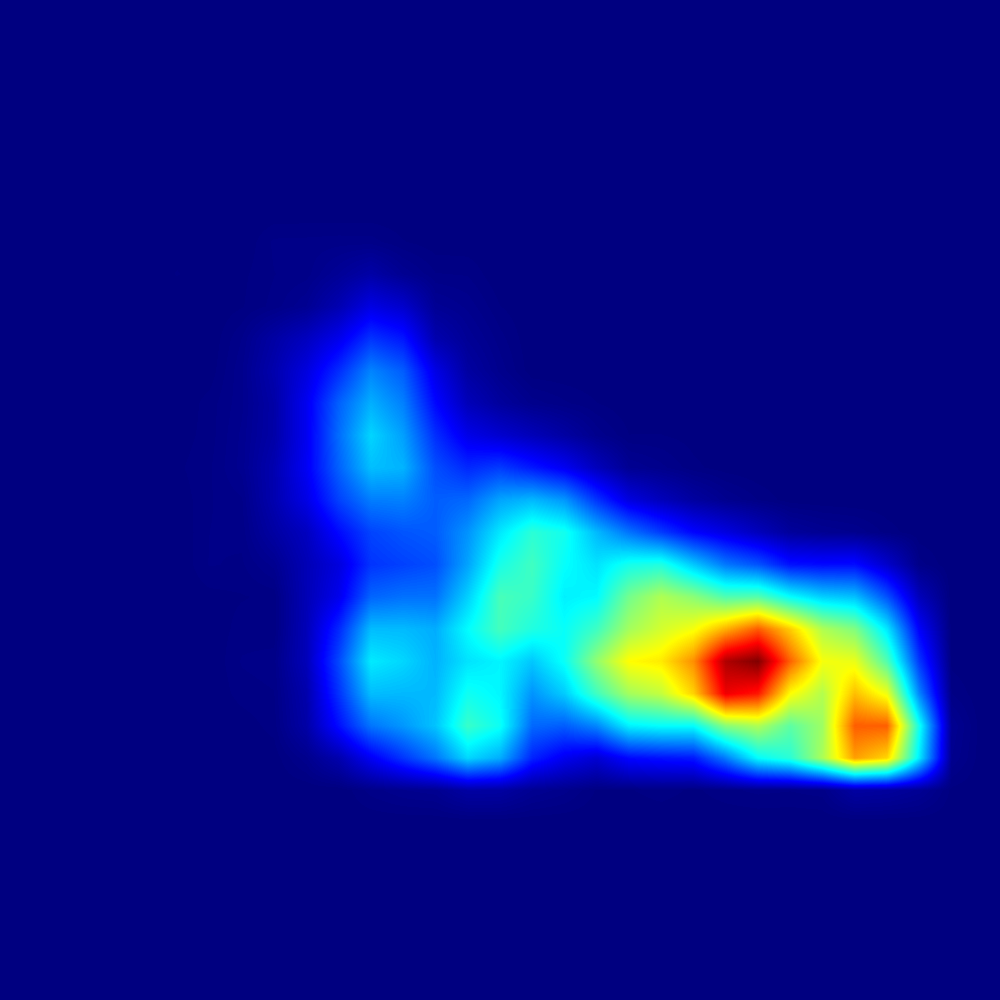

Supplement: Supplementary file 3 — Source Data File [file 41746_2022_681_MOESM3_ESM.zip › ARDA Map/Figure 4/17.png]

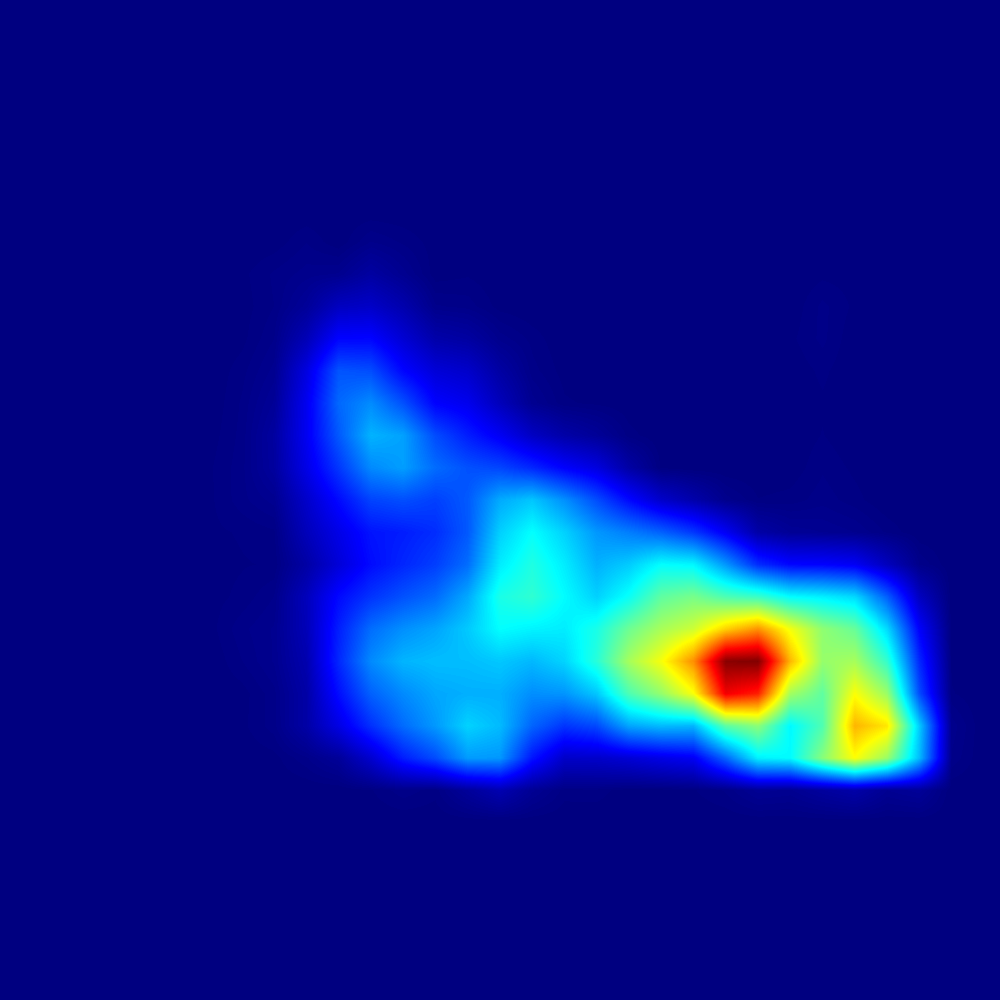

Supplement: Supplementary file 3 — Source Data File [file 41746_2022_681_MOESM3_ESM.zip › ARDA Map/Figure 4/18.png]

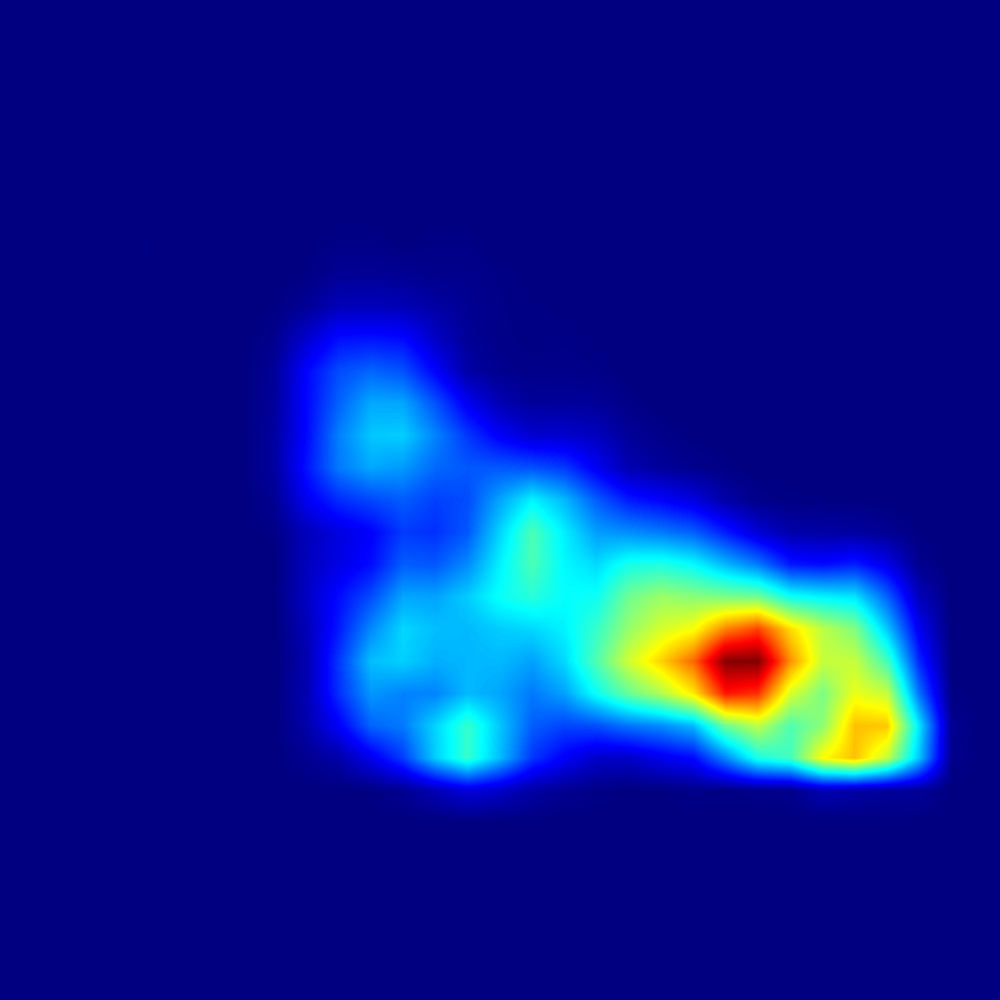

Supplement: Supplementary file 3 — Source Data File [file 41746_2022_681_MOESM3_ESM.zip › ARDA Map/Figure 4/19.png]

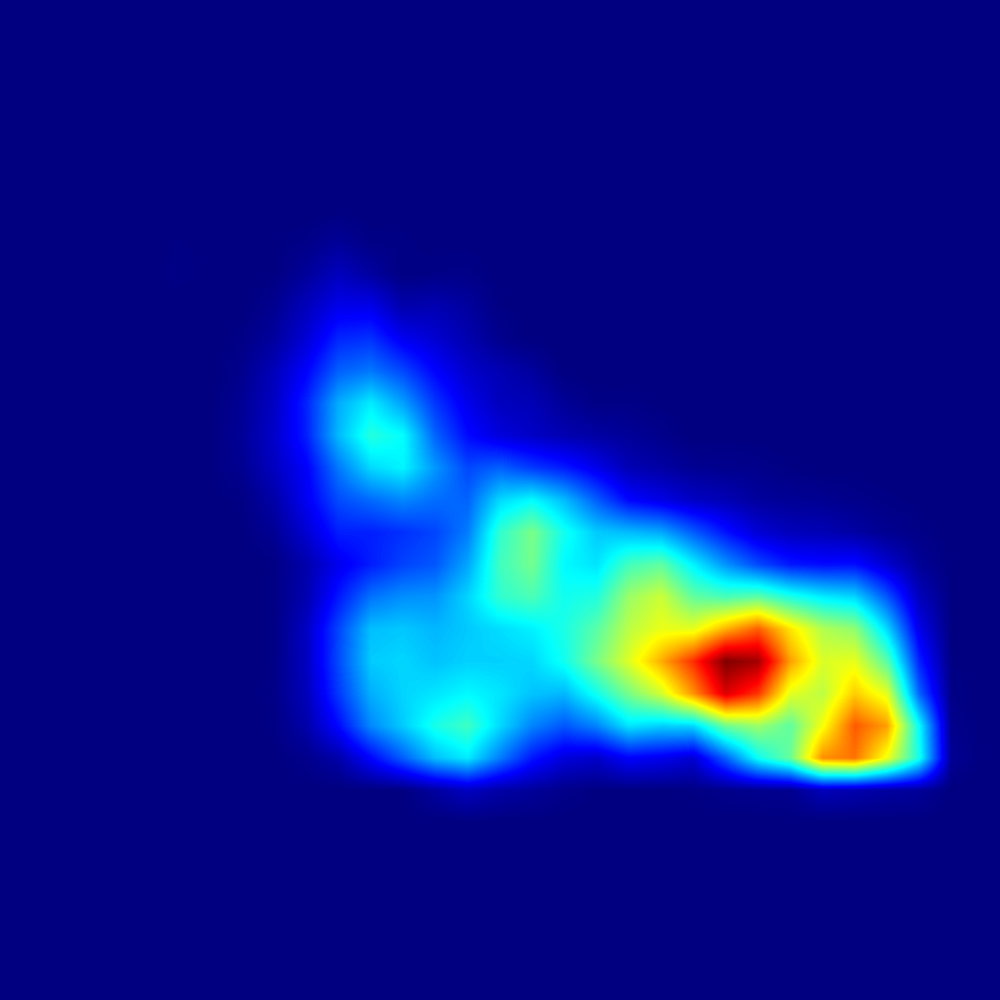

Supplement: Supplementary file 3 — Source Data File [file 41746_2022_681_MOESM3_ESM.zip › ARDA Map/Figure 4/20.png]

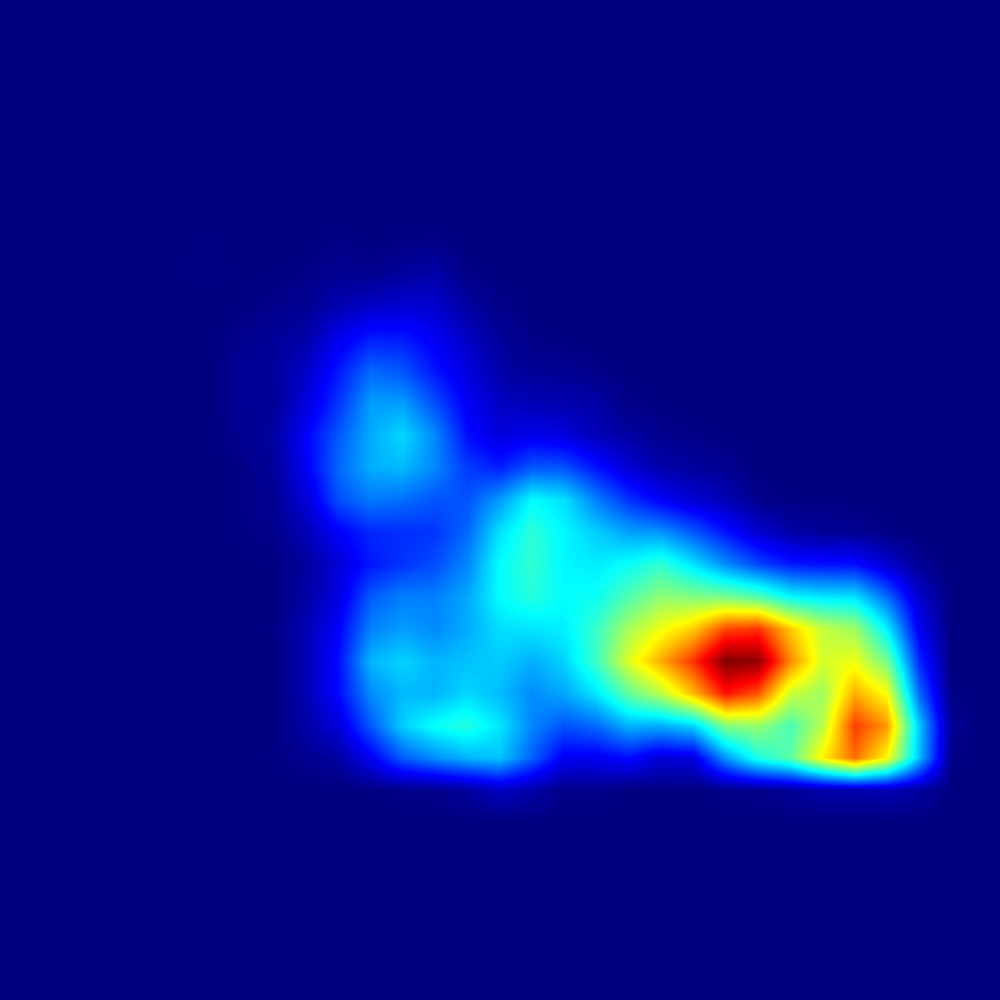

Supplement: Supplementary file 3 — Source Data File [file 41746_2022_681_MOESM3_ESM.zip › ARDA Map/Figure 4/21.png]

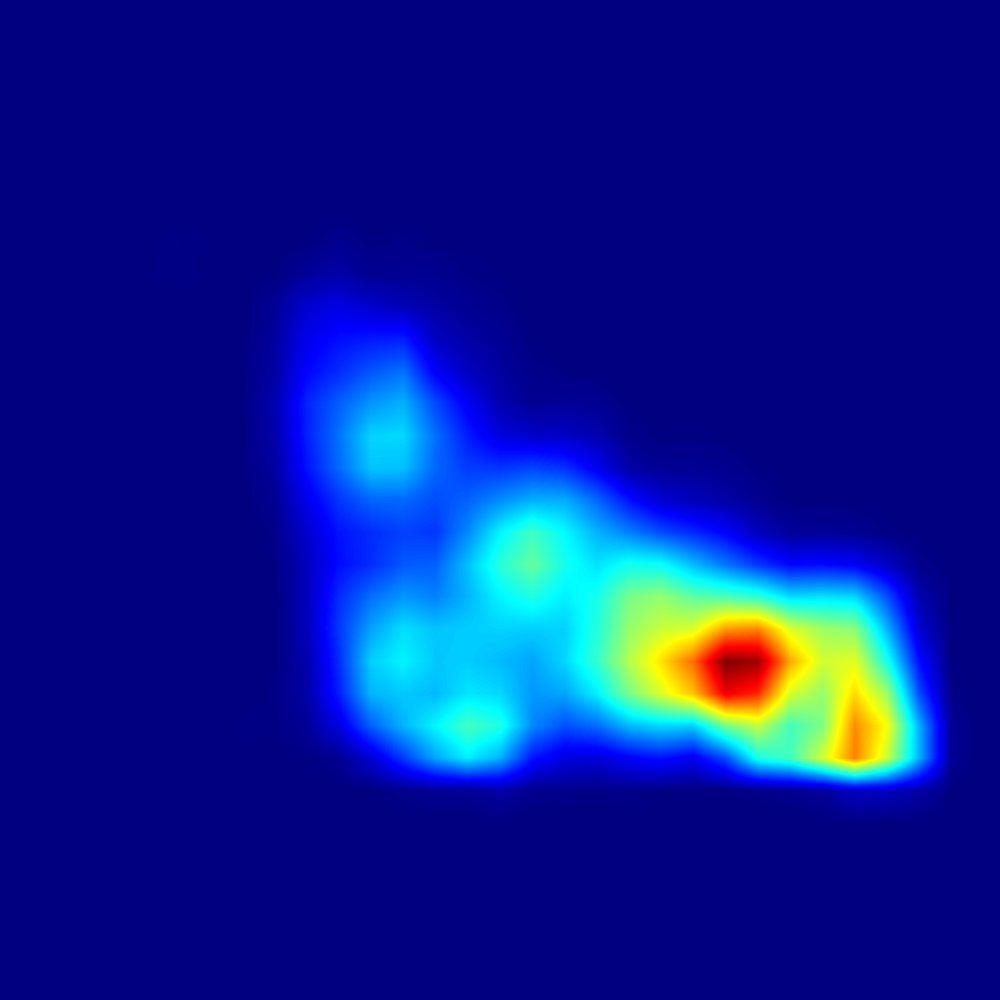

Supplement: Supplementary file 3 — Source Data File [file 41746_2022_681_MOESM3_ESM.zip › ARDA Map/Figure 4/22.png]

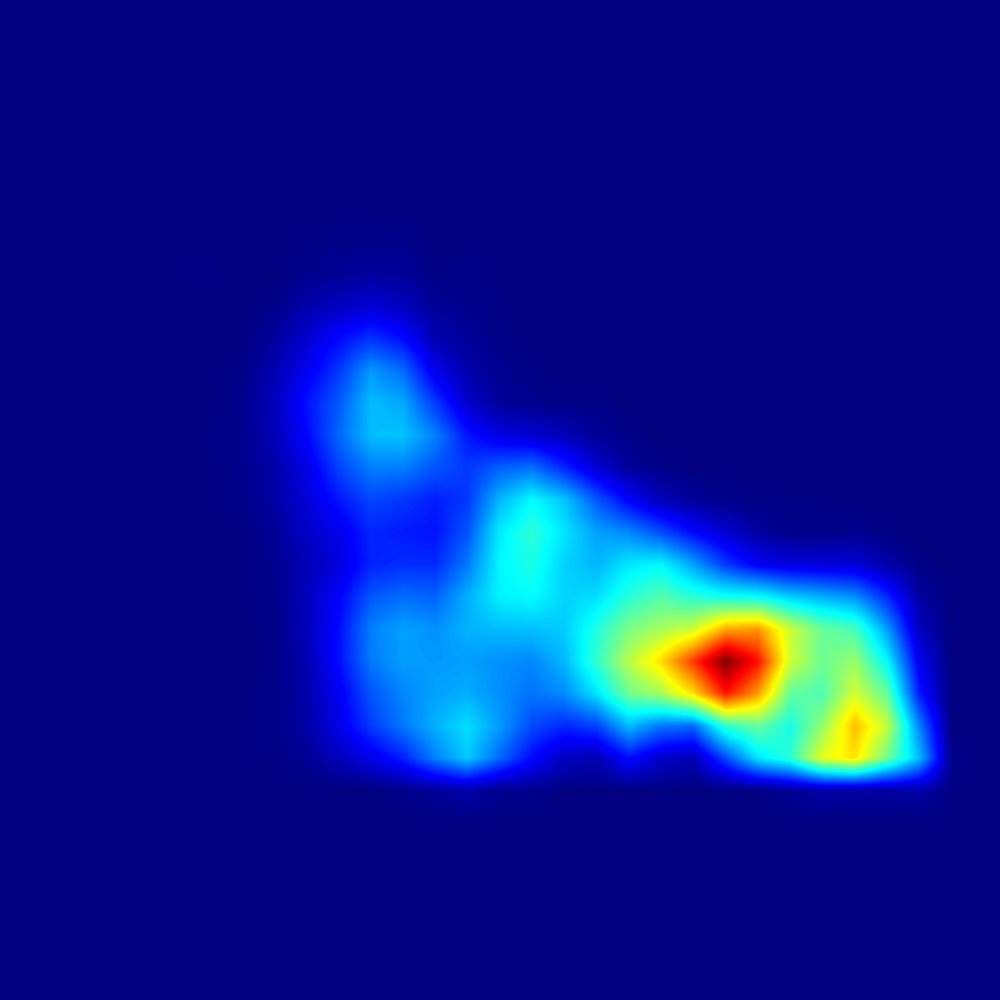

Supplement: Supplementary file 3 — Source Data File [file 41746_2022_681_MOESM3_ESM.zip › ARDA Map/Figure 4/23.png]

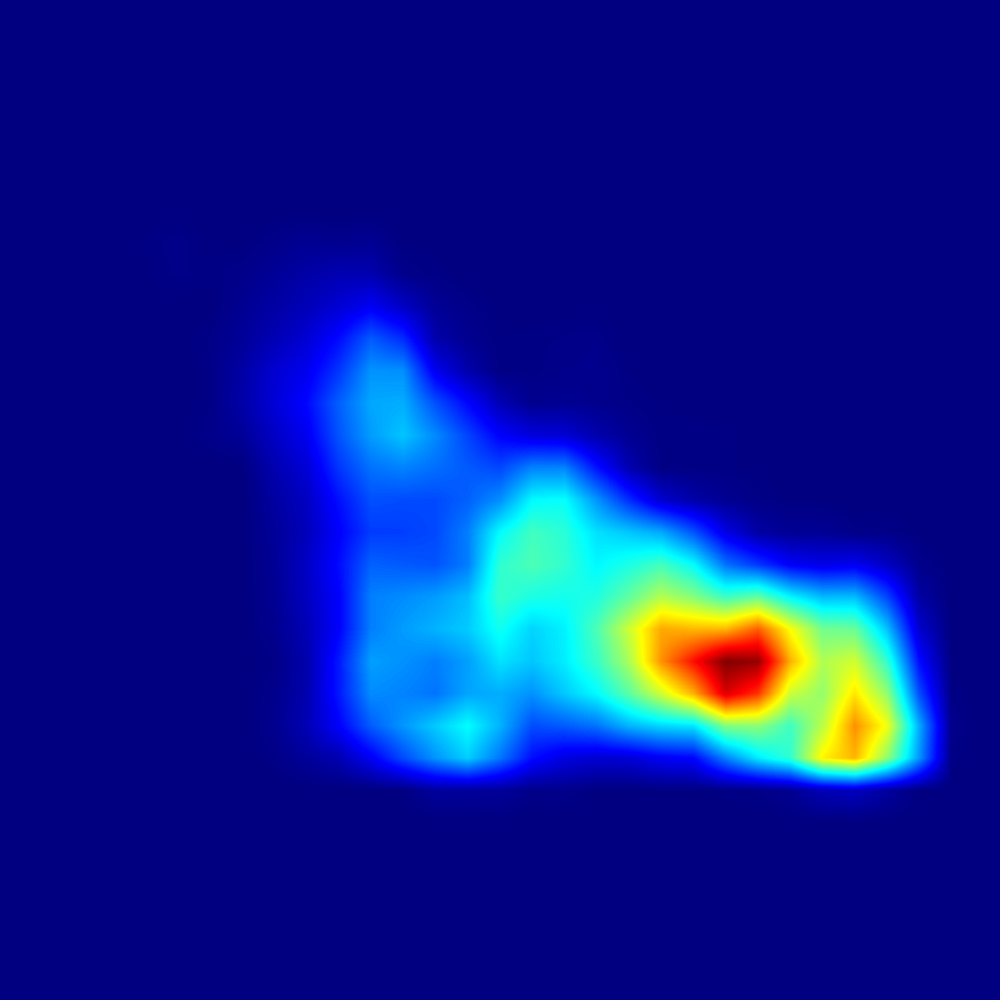

Supplement: Supplementary file 3 — Source Data File [file 41746_2022_681_MOESM3_ESM.zip › ARDA Map/Figure 4/24.png]

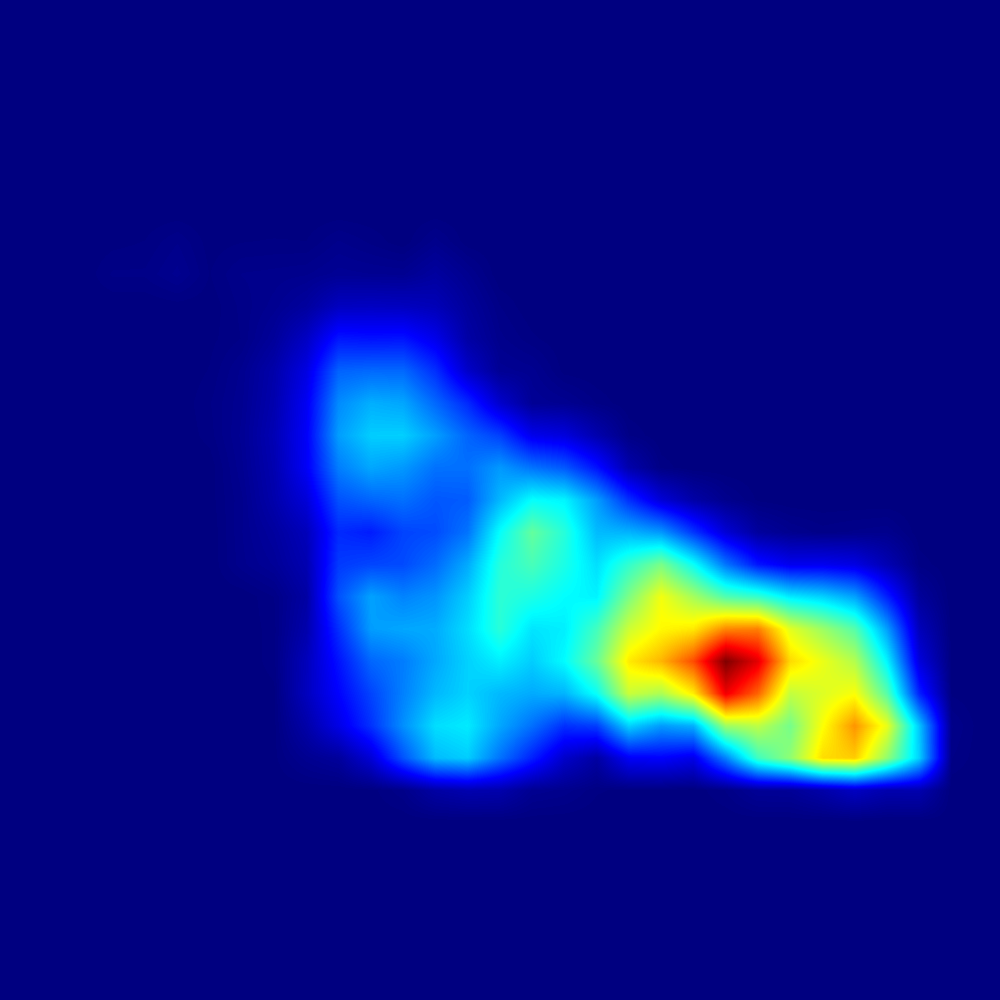

Supplement: Supplementary file 3 — Source Data File [file 41746_2022_681_MOESM3_ESM.zip › ARDA Map/Figure 4/25.png]

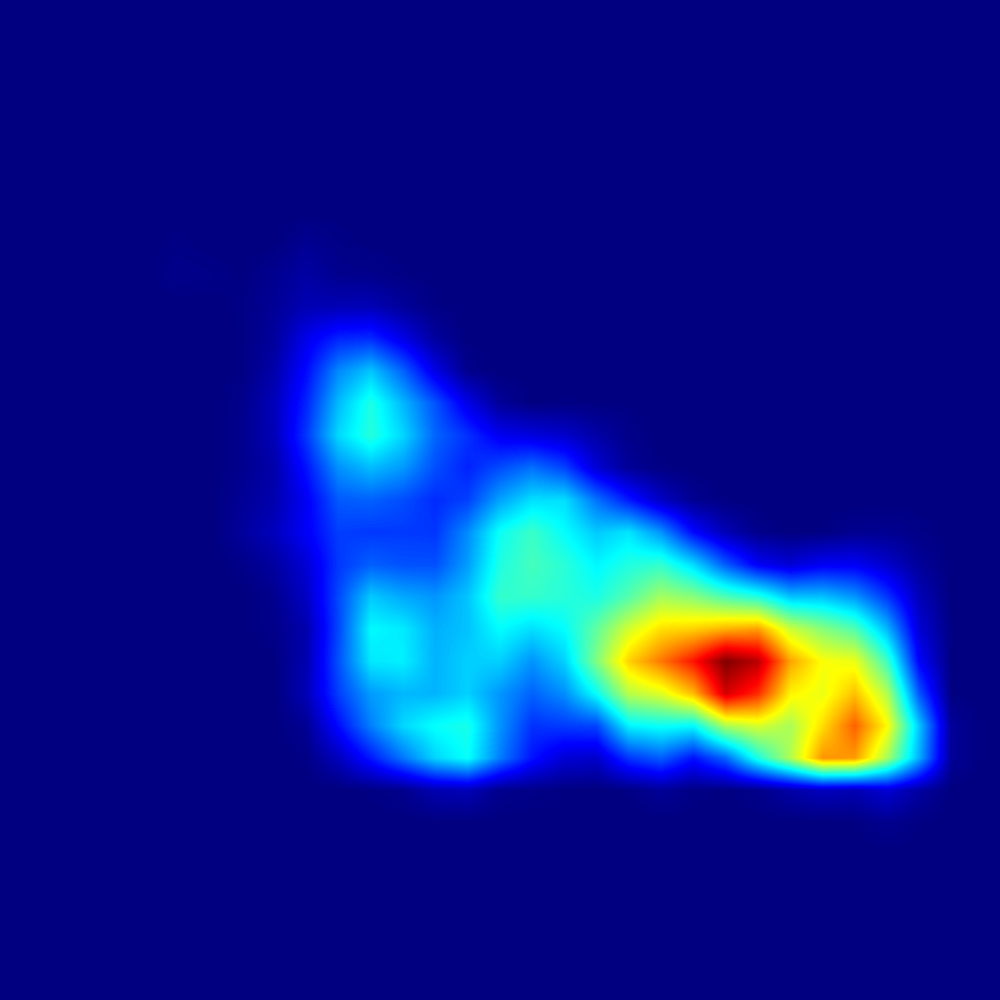

Supplement: Supplementary file 3 — Source Data File [file 41746_2022_681_MOESM3_ESM.zip › ARDA Map/Figure 4/26.png]

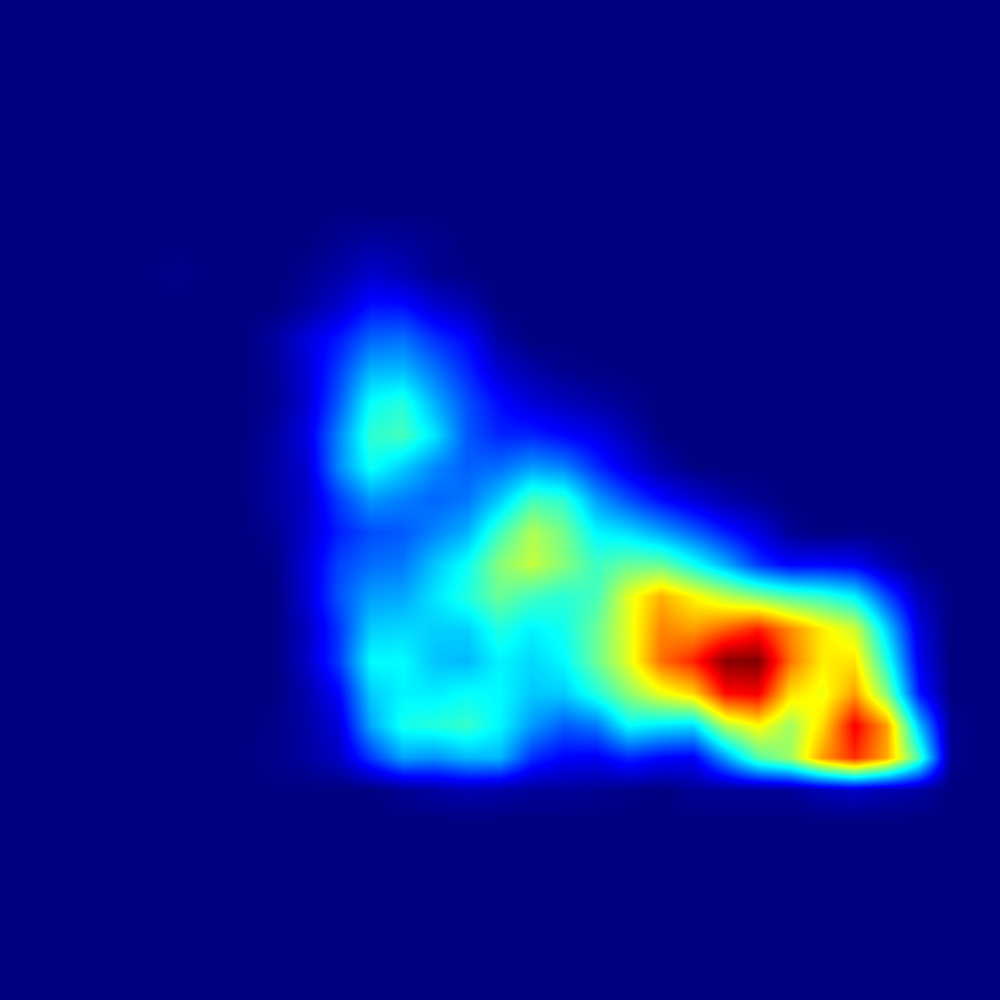

Supplement: Supplementary file 3 — Source Data File [file 41746_2022_681_MOESM3_ESM.zip › ARDA Map/Figure 4/27.png]

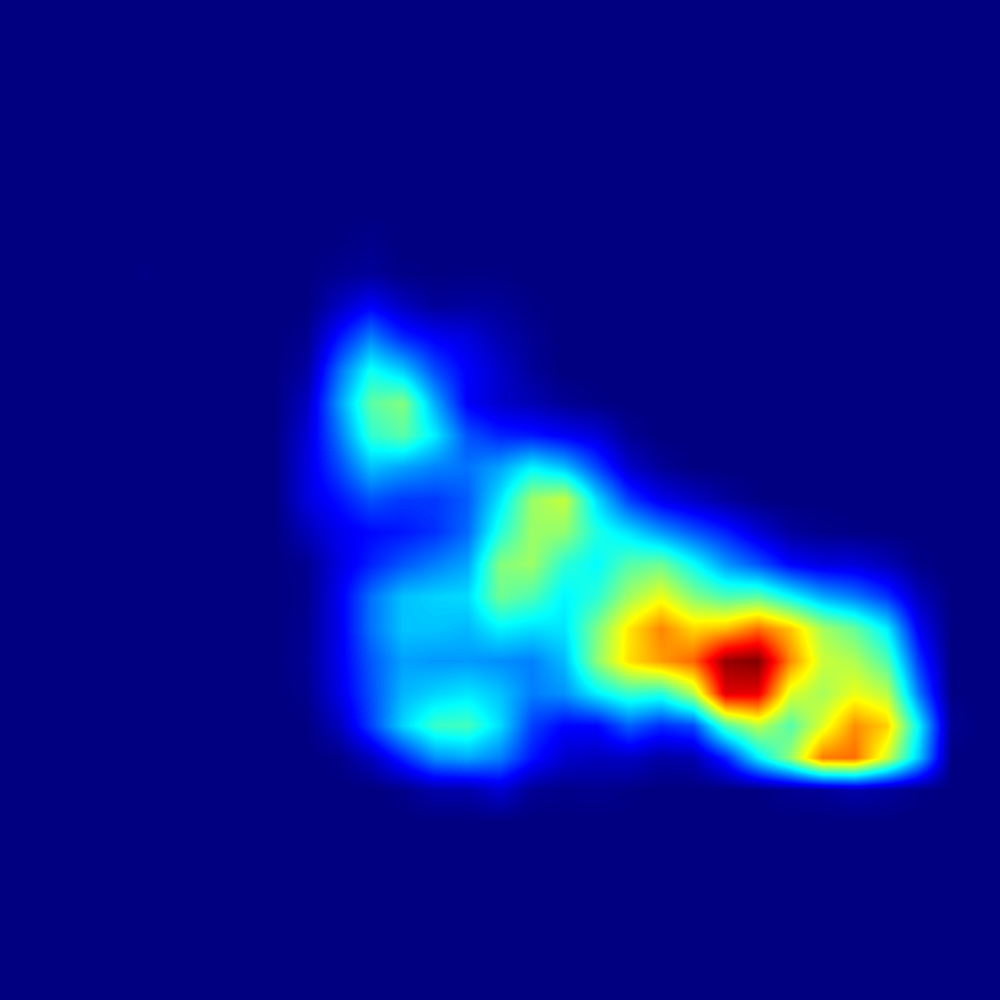

Supplement: Supplementary file 3 — Source Data File [file 41746_2022_681_MOESM3_ESM.zip › ARDA Map/Figure 4/28.png]

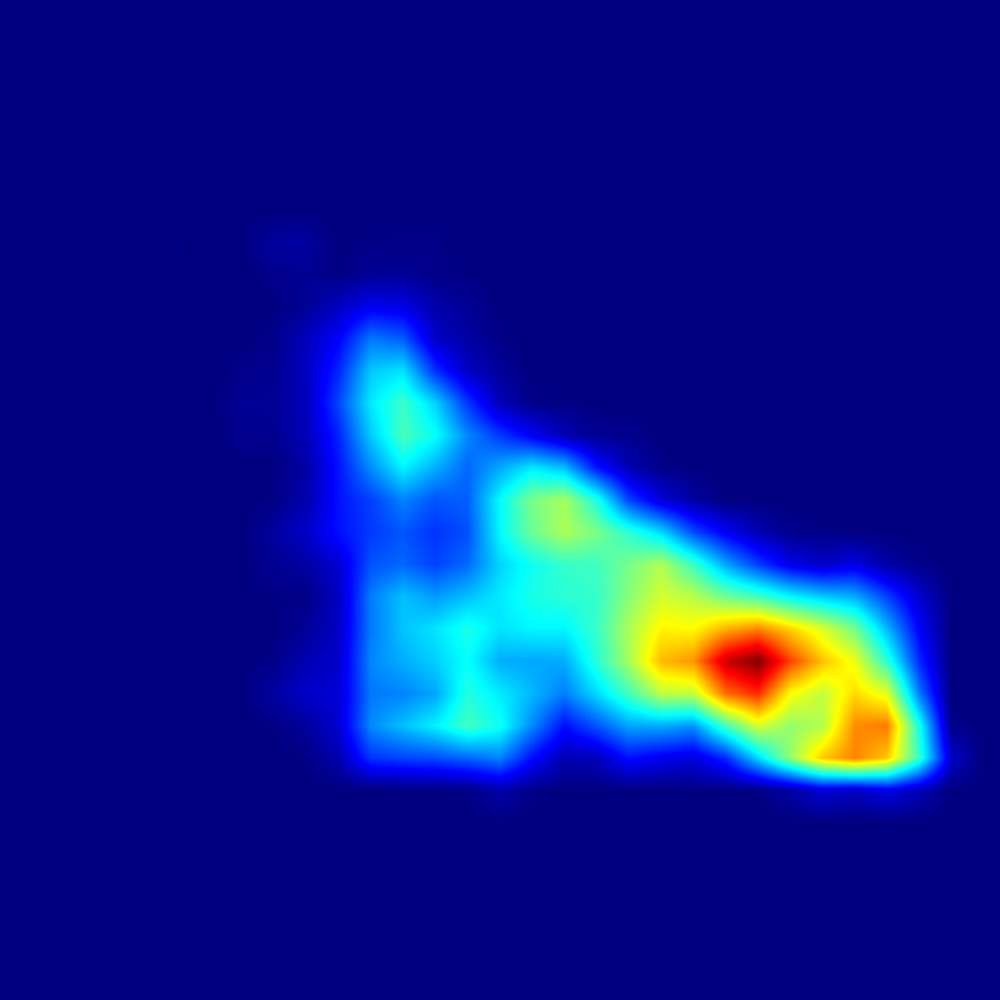

Supplement: Supplementary file 3 — Source Data File [file 41746_2022_681_MOESM3_ESM.zip › ARDA Map/Figure 4/29.png]

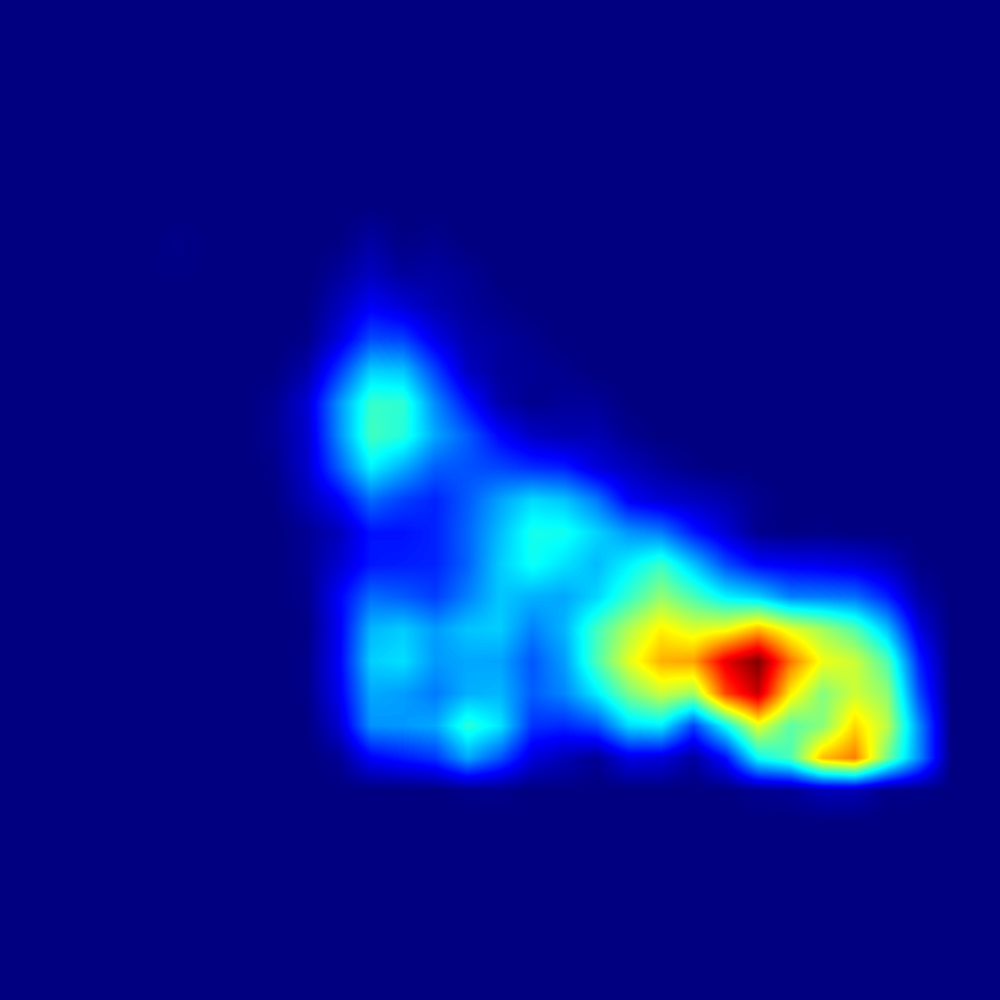

Supplement: Supplementary file 3 — Source Data File [file 41746_2022_681_MOESM3_ESM.zip › ARDA Map/Figure 4/30.png]

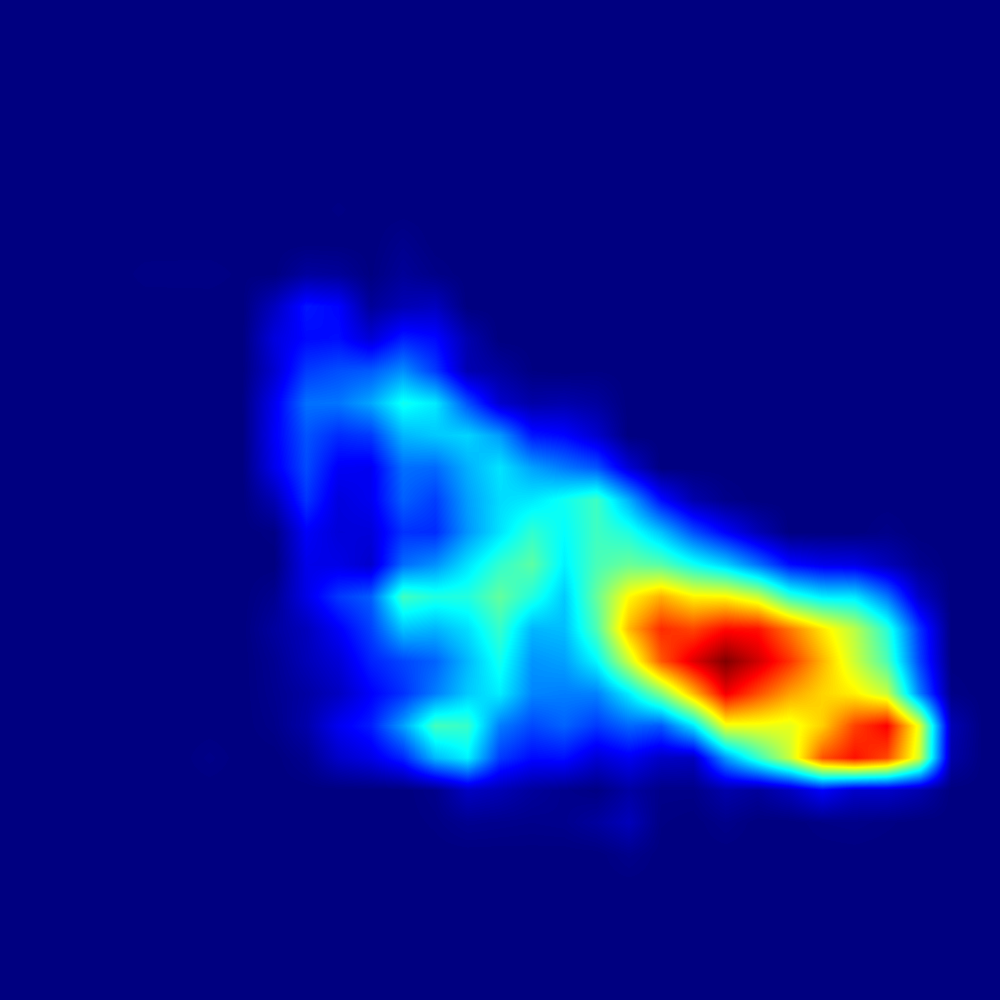

Supplement: Supplementary file 3 — Source Data File [file 41746_2022_681_MOESM3_ESM.zip › ARDA Map/Figure 4/31.png]

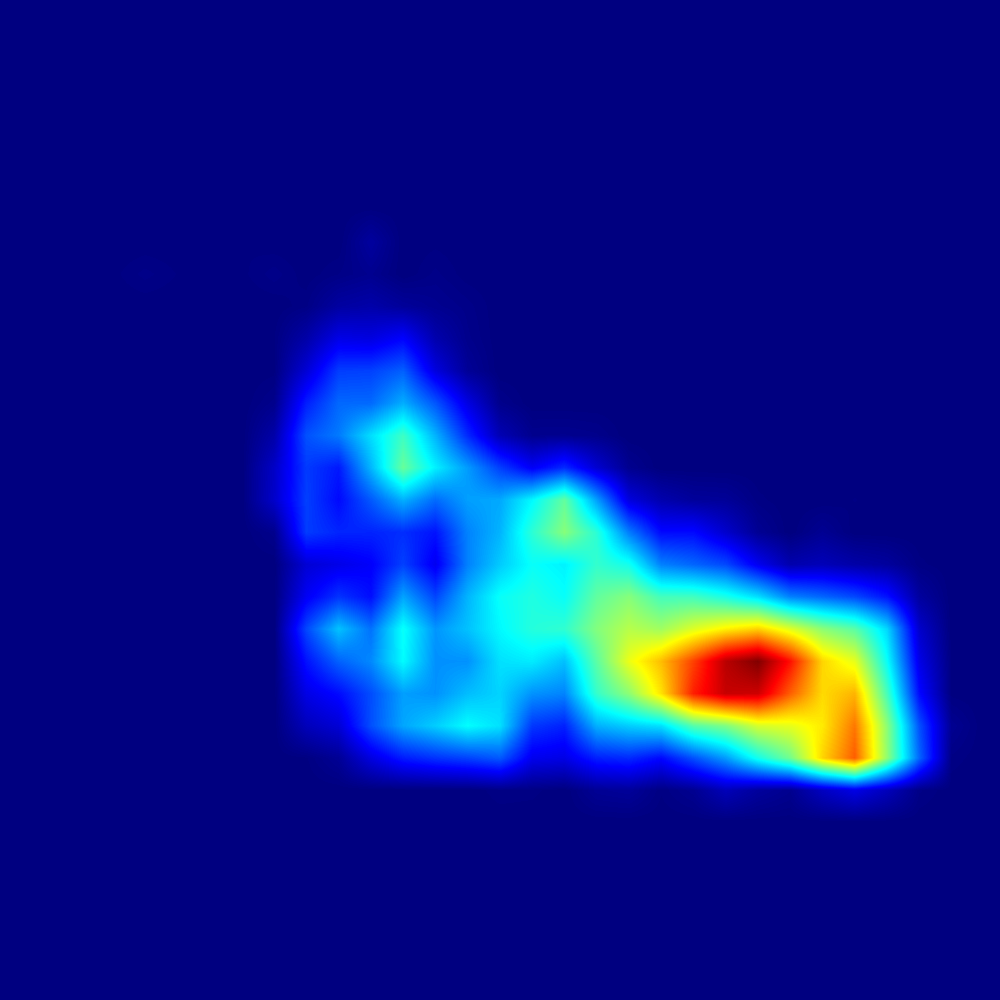

Supplement: Supplementary file 3 — Source Data File [file 41746_2022_681_MOESM3_ESM.zip › ARDA Map/Figure 4/32.png]

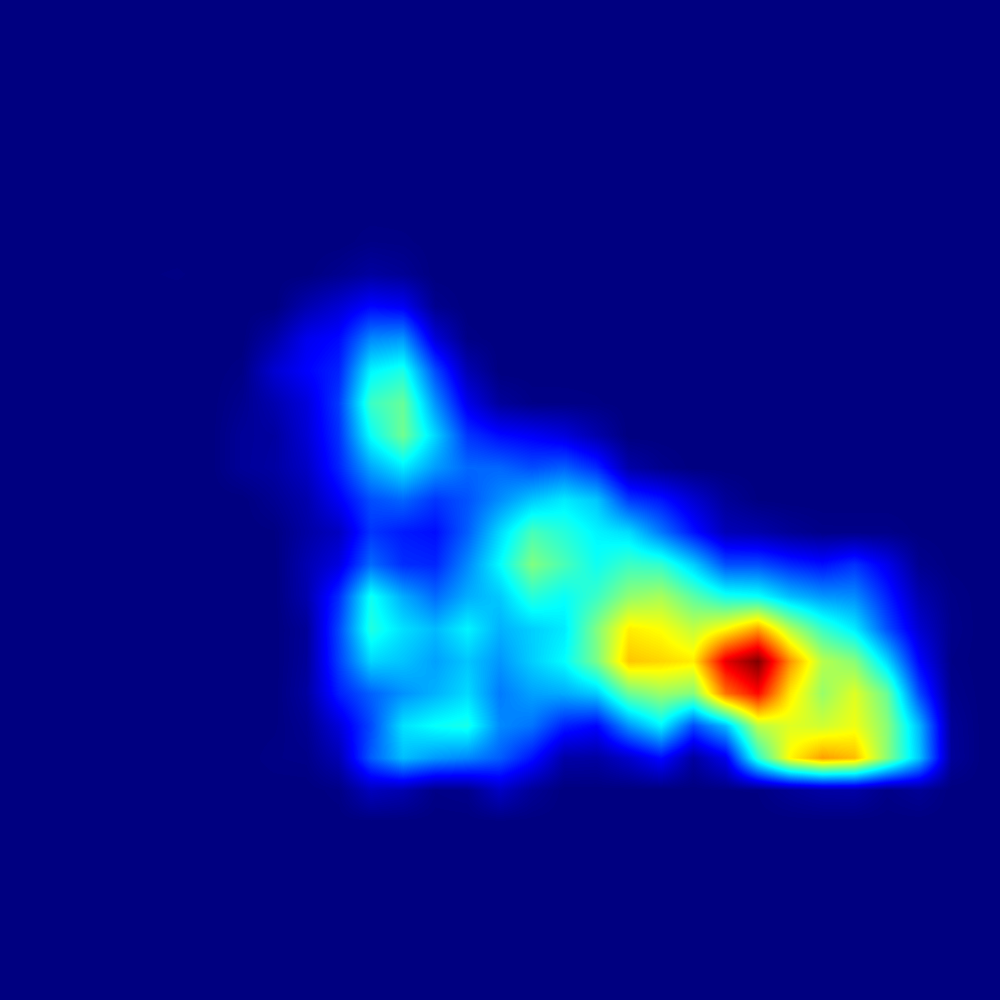

Supplement: Supplementary file 3 — Source Data File [file 41746_2022_681_MOESM3_ESM.zip › ARDA Map/Figure 4/33.png]

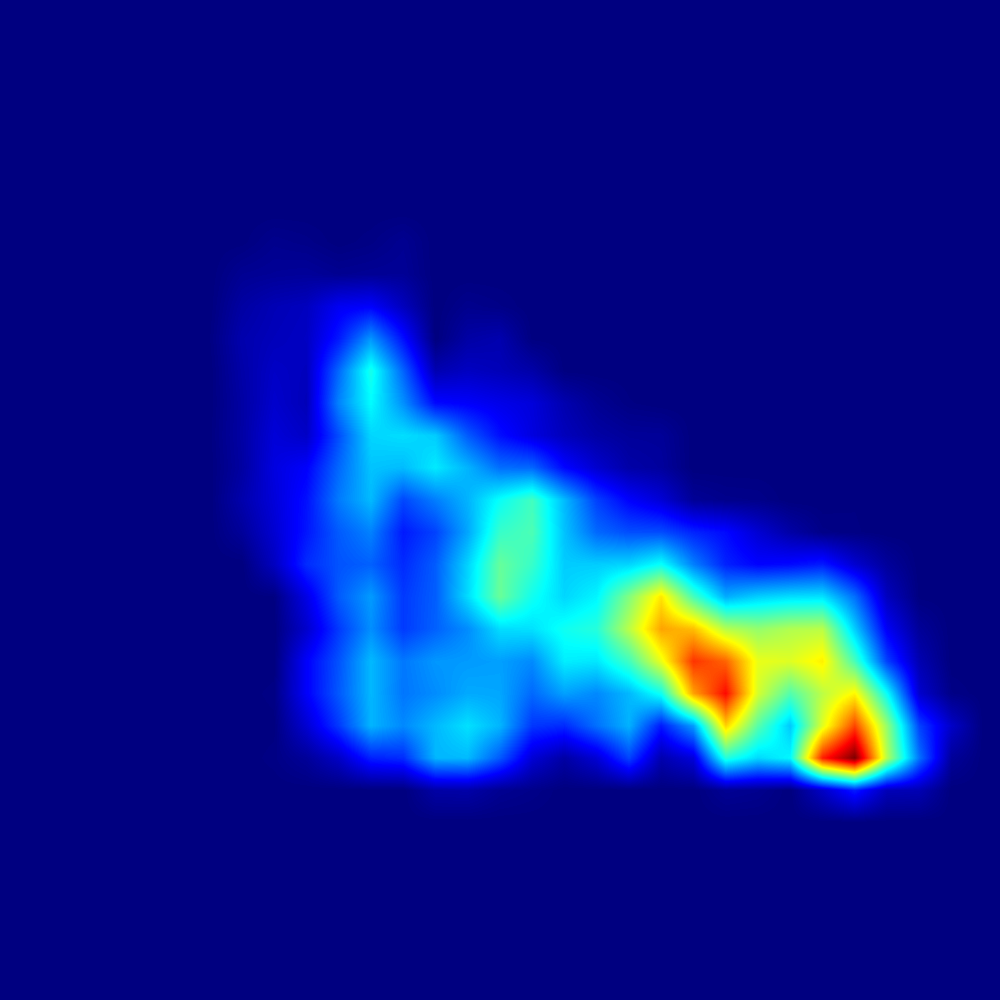

Supplement: Supplementary file 3 — Source Data File [file 41746_2022_681_MOESM3_ESM.zip › ARDA Map/Figure 4/34.png]

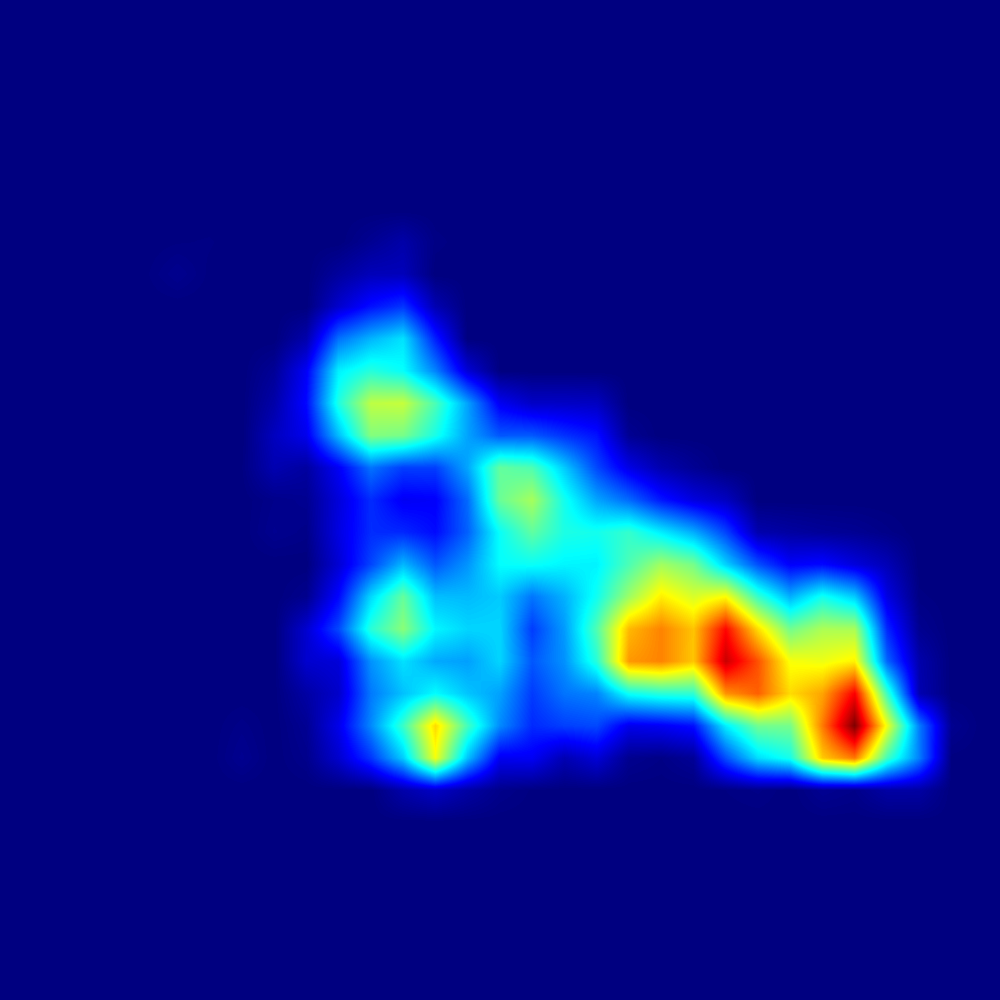

Supplement: Supplementary file 3 — Source Data File [file 41746_2022_681_MOESM3_ESM.zip › ARDA Map/Figure 4/35.png]

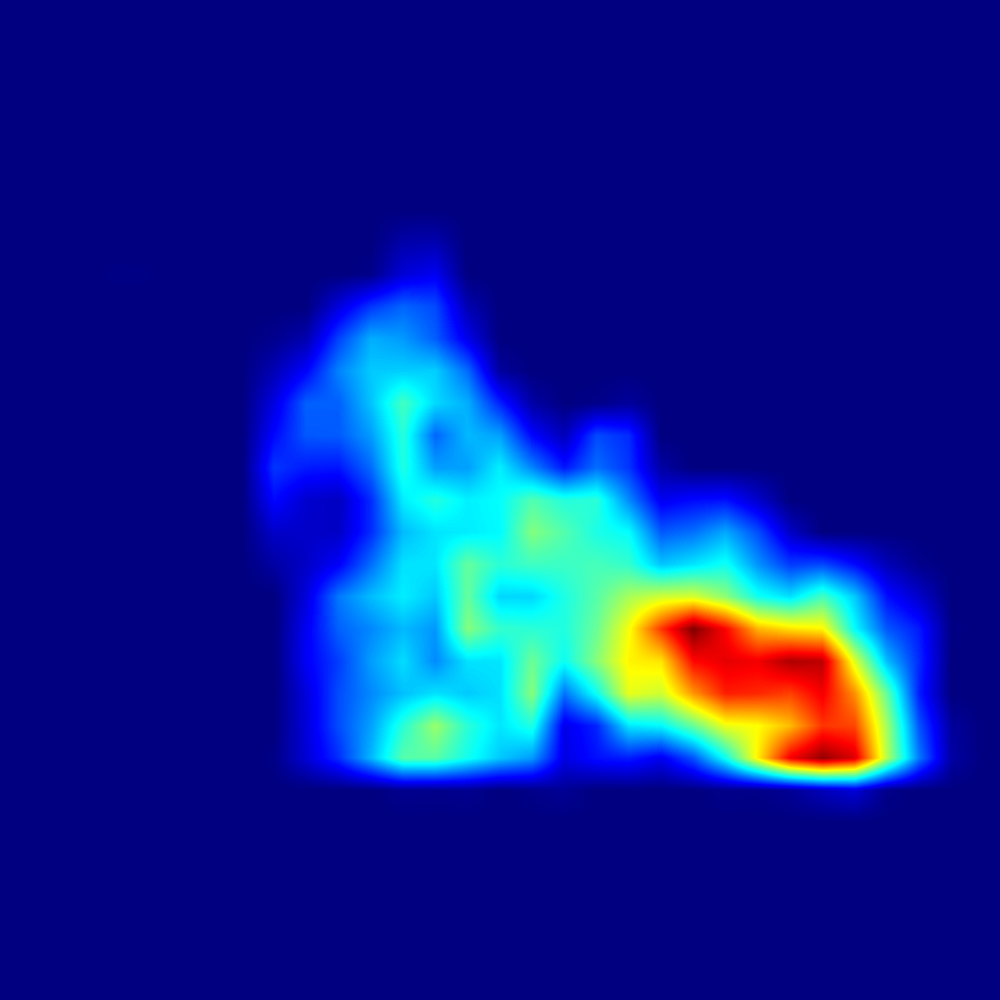

Supplement: Supplementary file 3 — Source Data File [file 41746_2022_681_MOESM3_ESM.zip › ARDA Map/Figure 4/37.png]

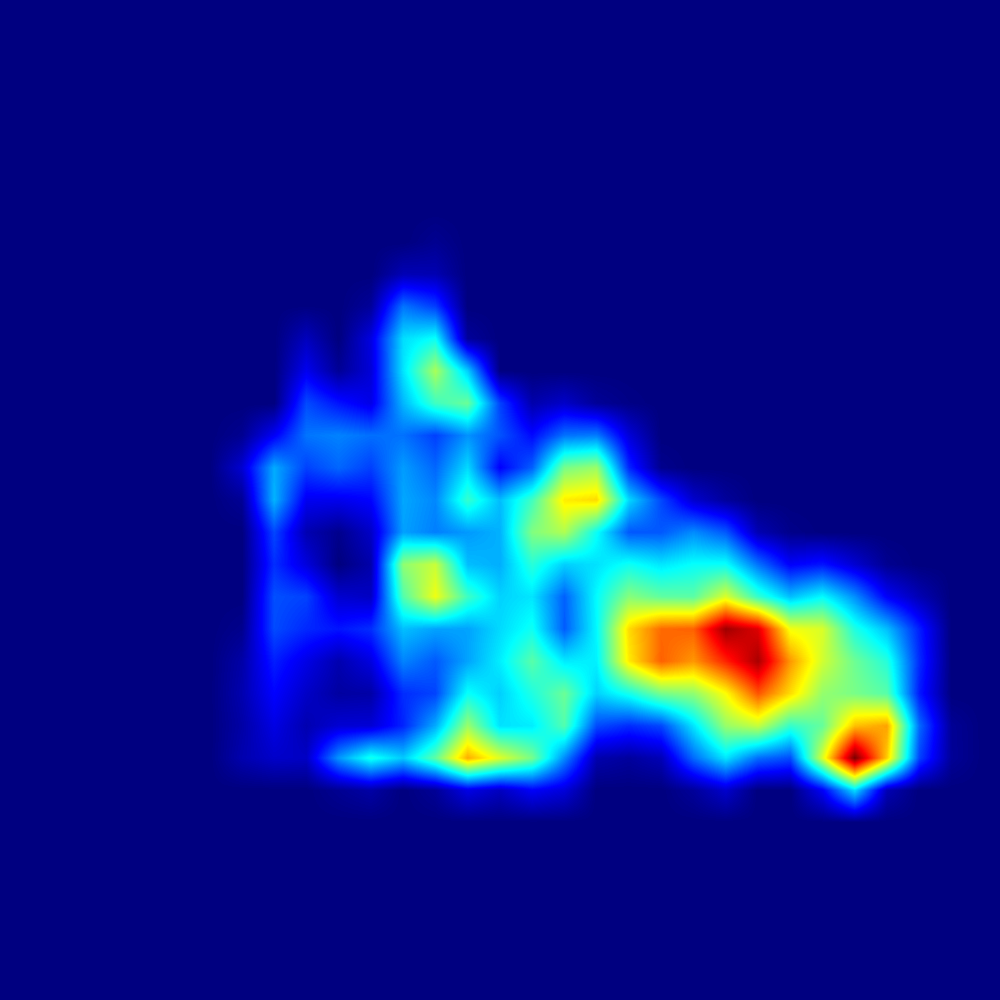

Supplement: Supplementary file 3 — Source Data File [file 41746_2022_681_MOESM3_ESM.zip › ARDA Map/Figure 4/38.png]

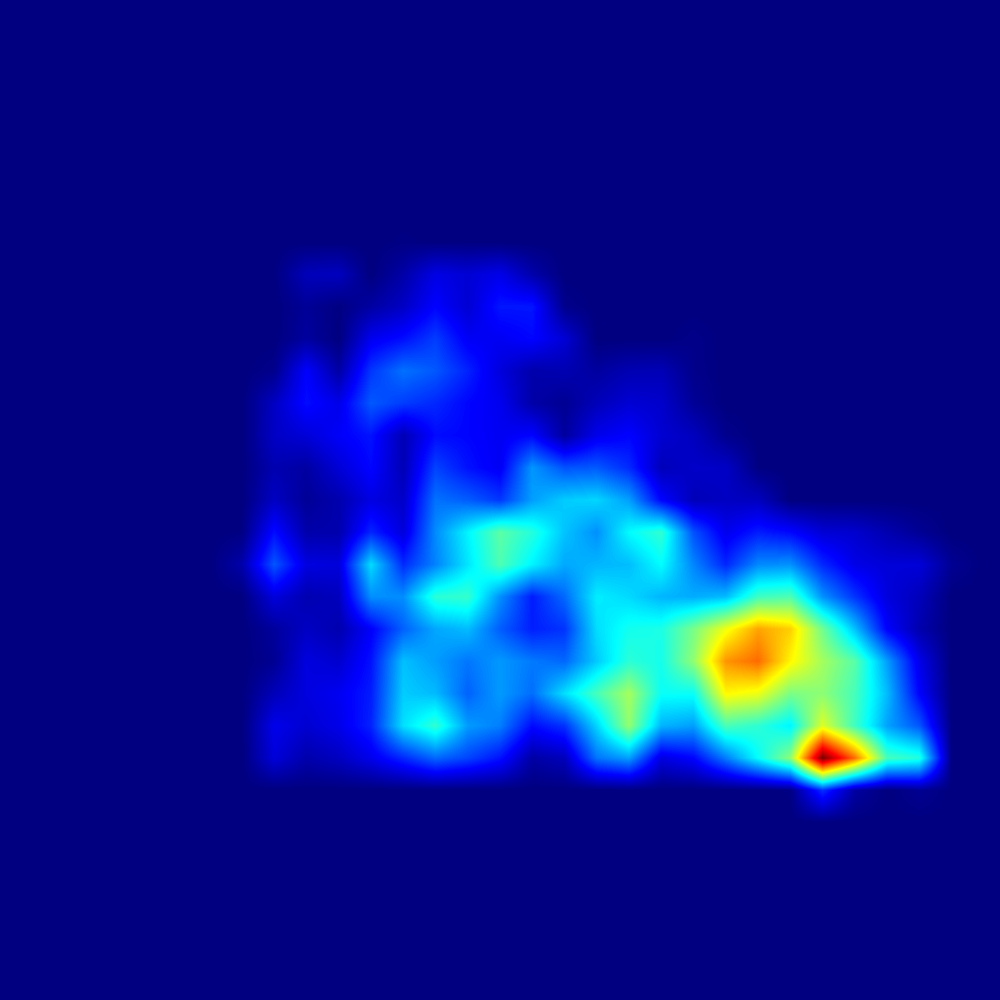

Supplement: Supplementary file 3 — Source Data File [file 41746_2022_681_MOESM3_ESM.zip › ARDA Map/Figure 4/39.png]

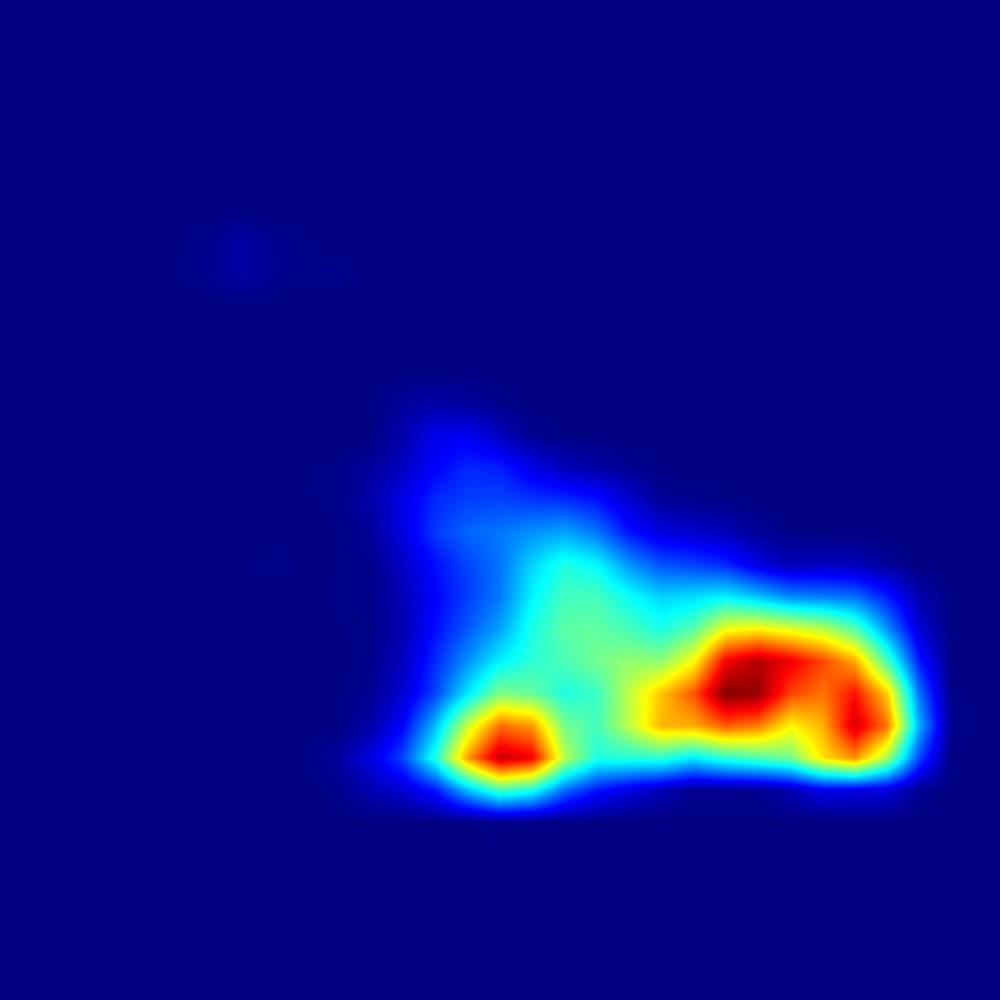

Supplement: Supplementary file 3 — Source Data File [file 41746_2022_681_MOESM3_ESM.zip › ARDA Map/Figure 4/4.png]

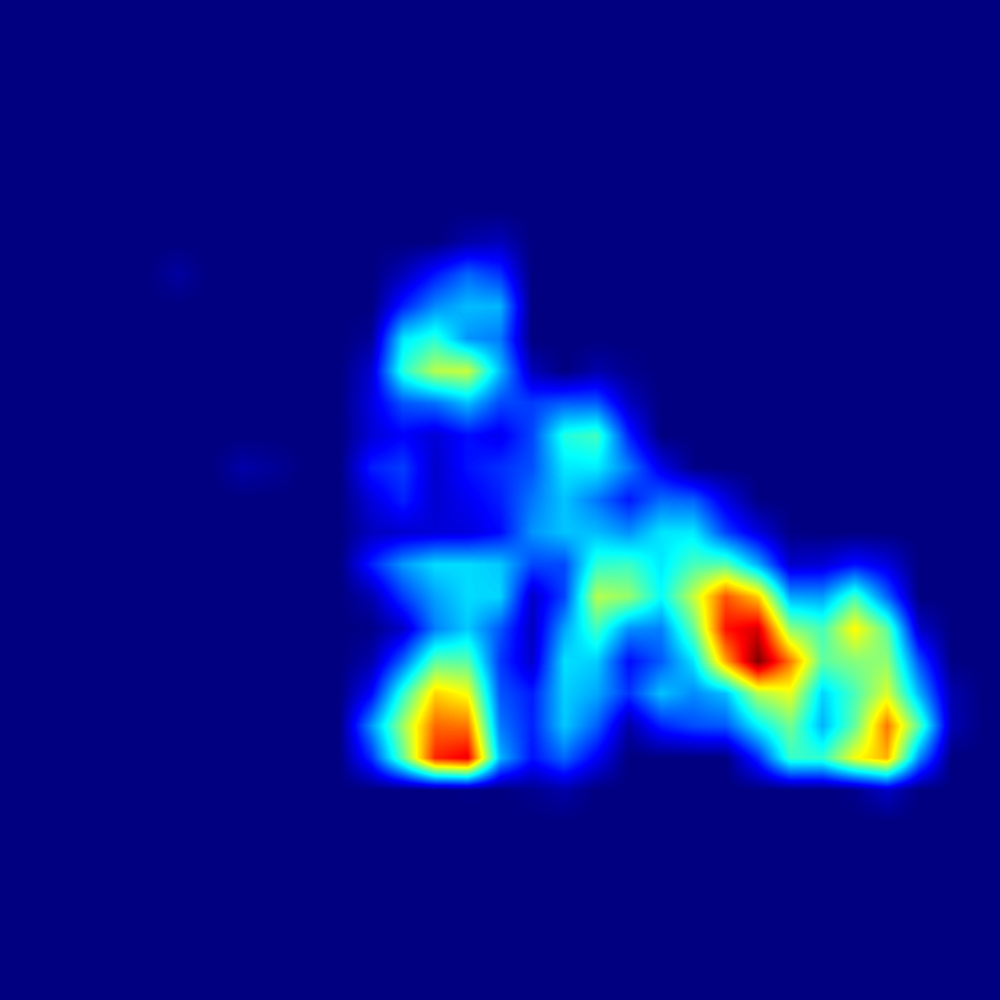

Supplement: Supplementary file 3 — Source Data File [file 41746_2022_681_MOESM3_ESM.zip › ARDA Map/Figure 4/40.png]

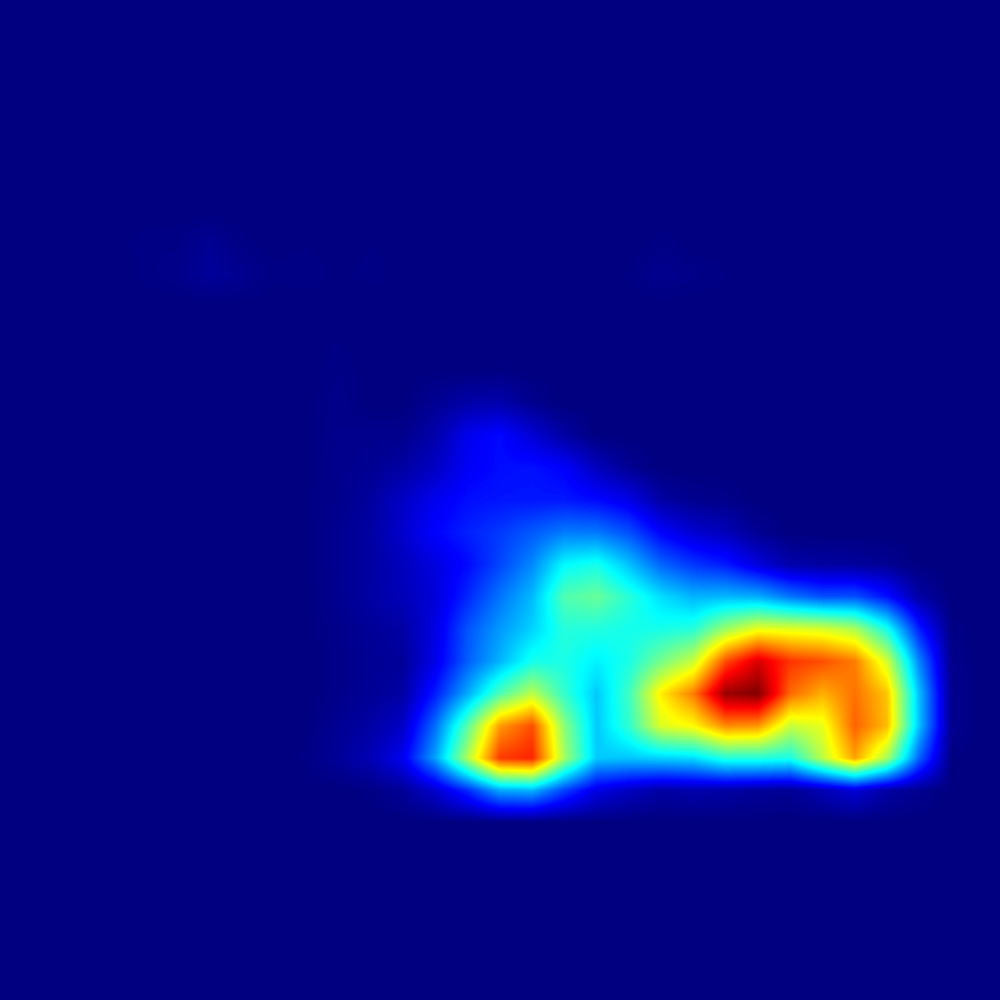

Supplement: Supplementary file 3 — Source Data File [file 41746_2022_681_MOESM3_ESM.zip › ARDA Map/Figure 4/5.png]

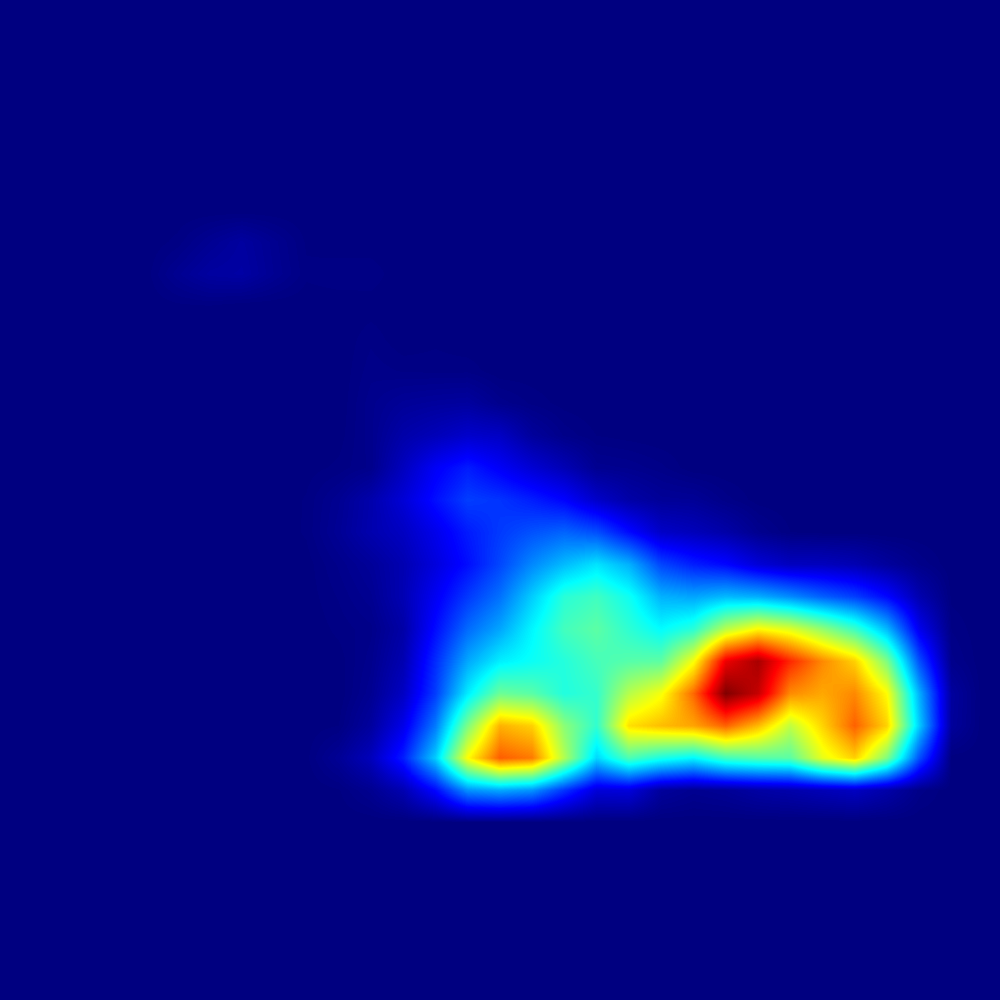

Supplement: Supplementary file 3 — Source Data File [file 41746_2022_681_MOESM3_ESM.zip › ARDA Map/Figure 4/6.png]

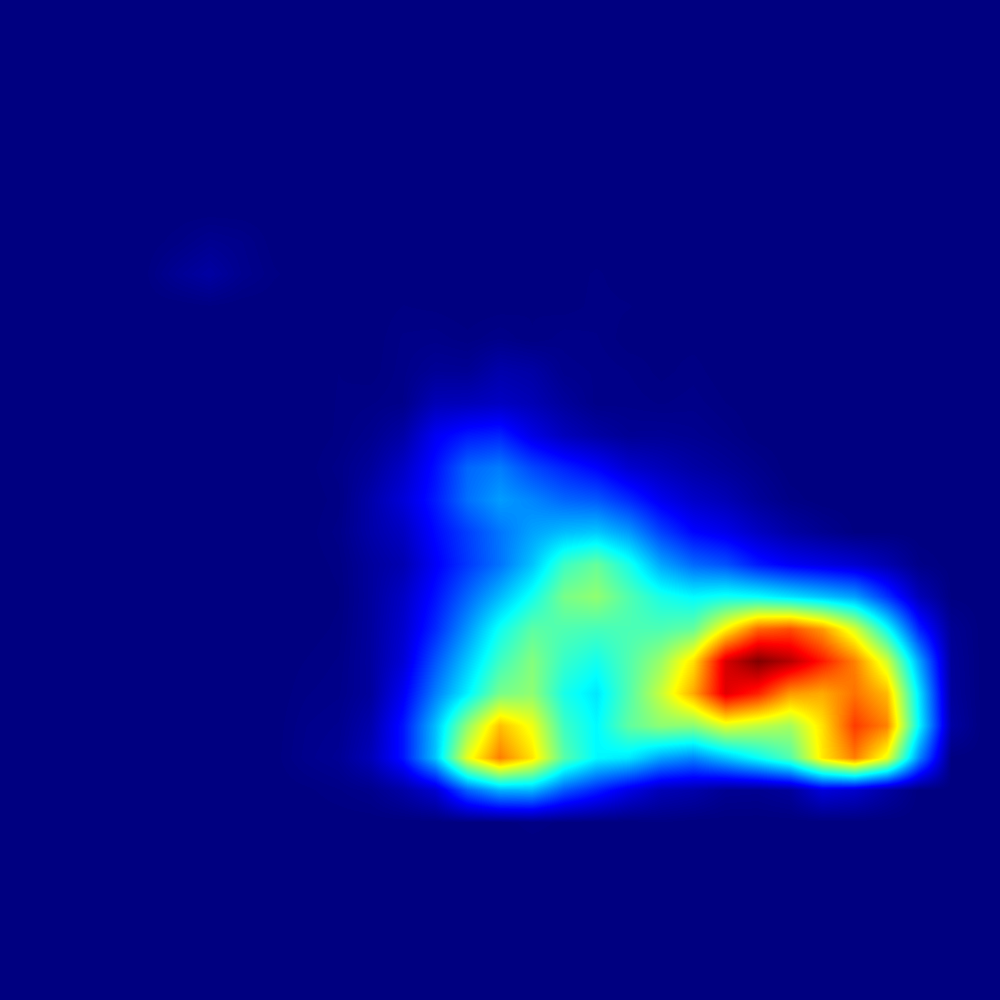

Supplement: Supplementary file 3 — Source Data File [file 41746_2022_681_MOESM3_ESM.zip › ARDA Map/Figure 4/7.png]

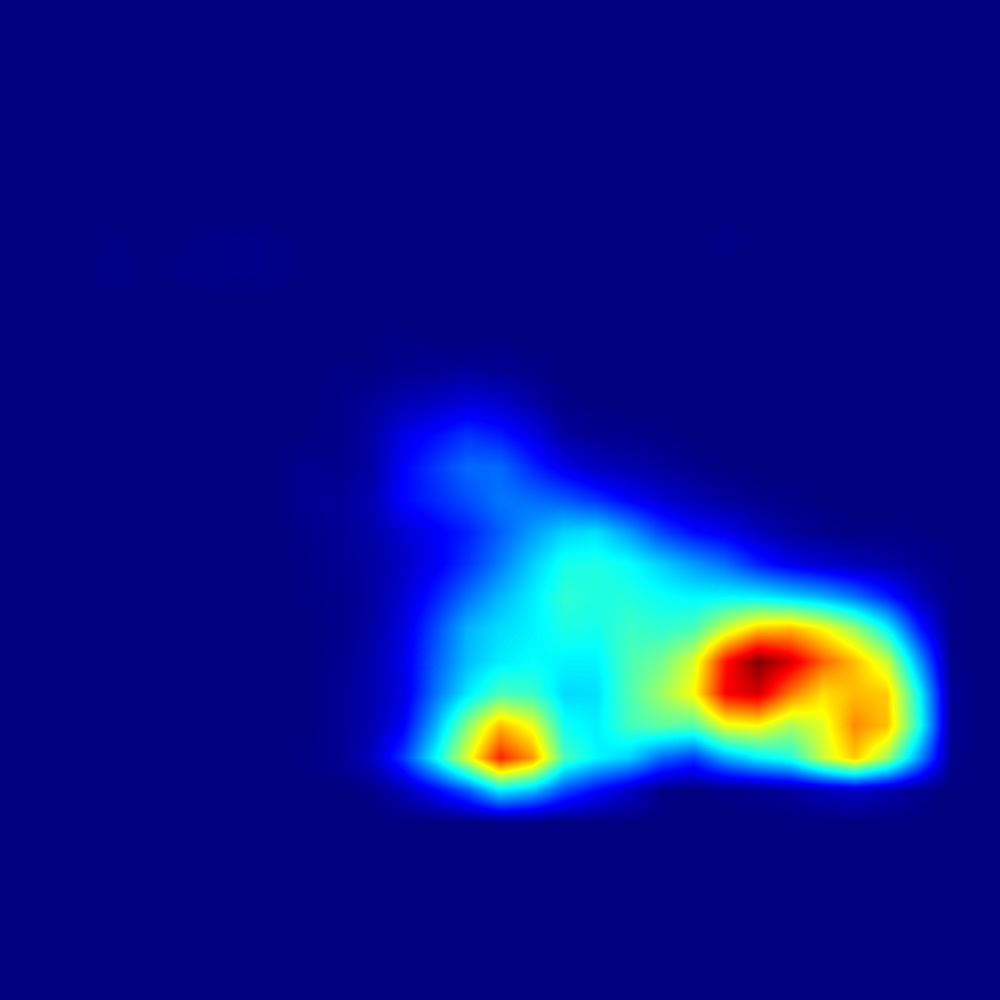

Supplement: Supplementary file 3 — Source Data File [file 41746_2022_681_MOESM3_ESM.zip › ARDA Map/Figure 4/8.png]

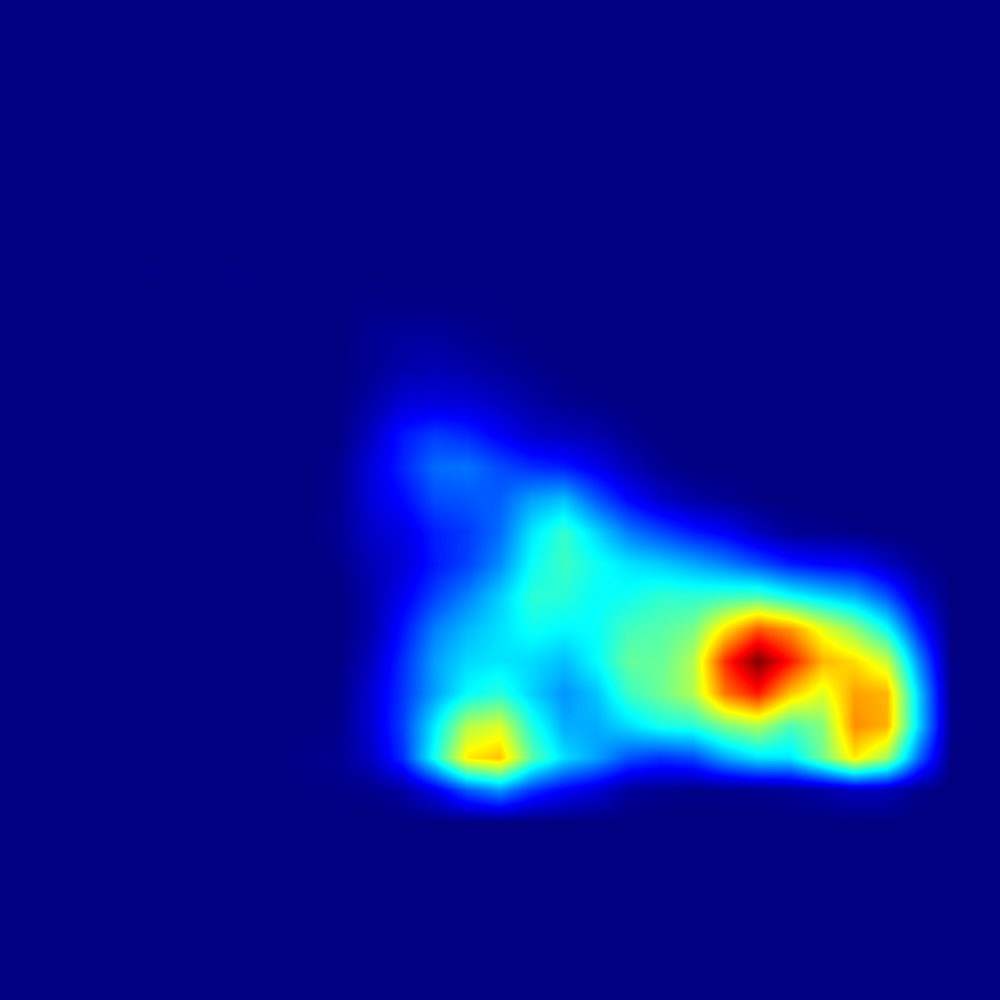

Supplement: Supplementary file 3 — Source Data File [file 41746_2022_681_MOESM3_ESM.zip › ARDA Map/Figure 4/9.png]

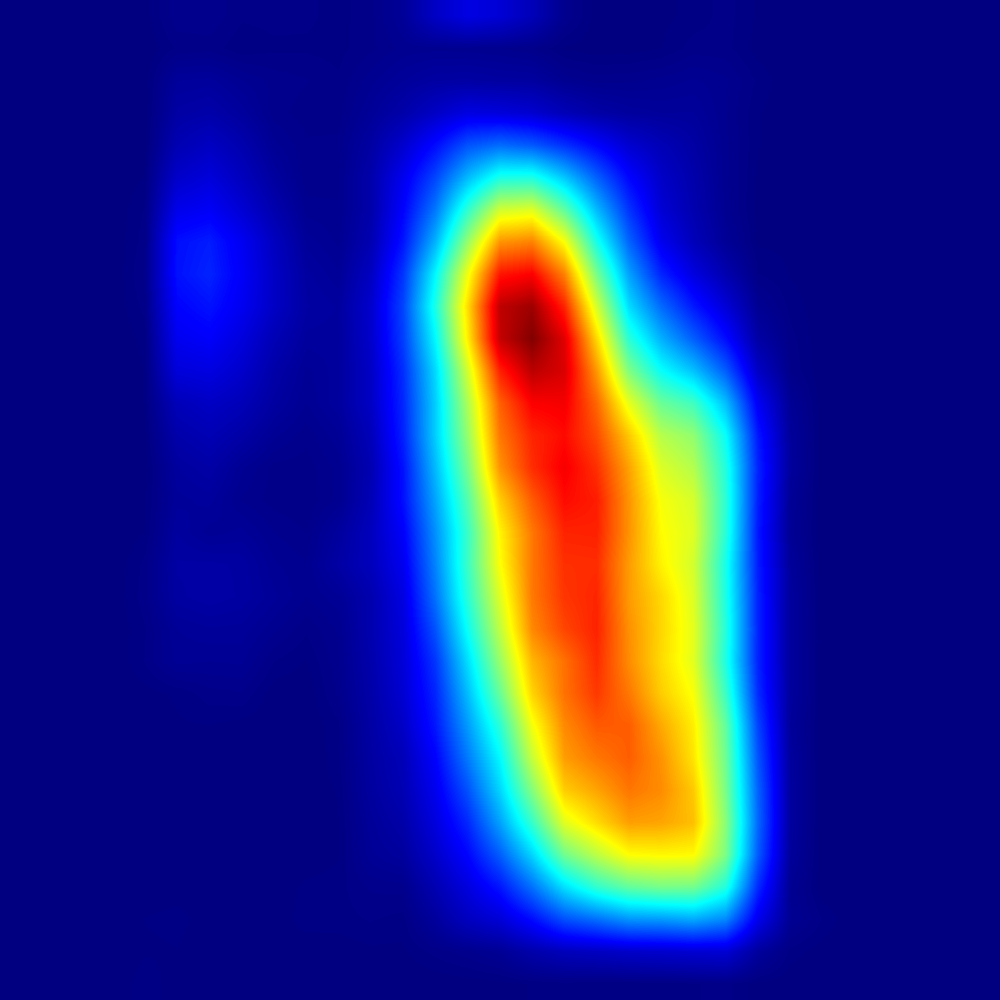

Supplement: Supplementary file 3 — Source Data File [file 41746_2022_681_MOESM3_ESM.zip › ARDA Map/Figure 5/10.png]

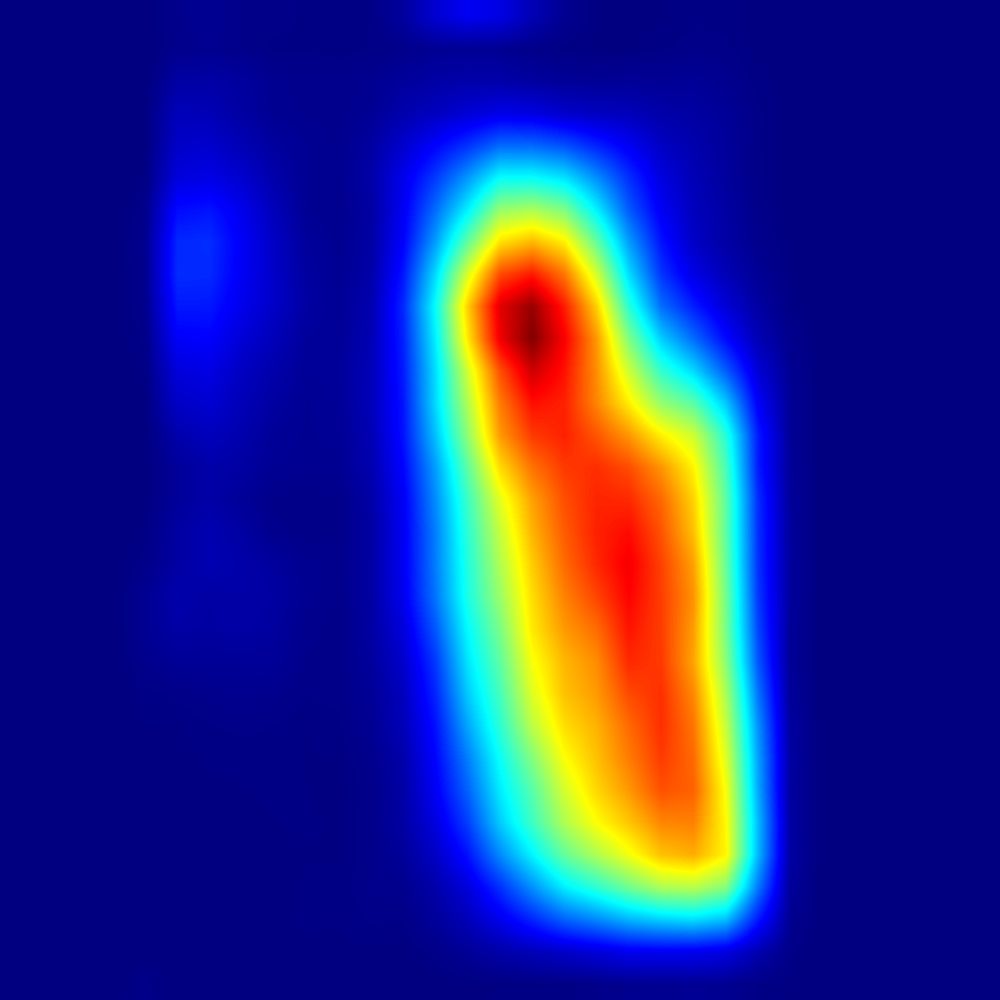

Supplement: Supplementary file 3 — Source Data File [file 41746_2022_681_MOESM3_ESM.zip › ARDA Map/Figure 5/11.png]

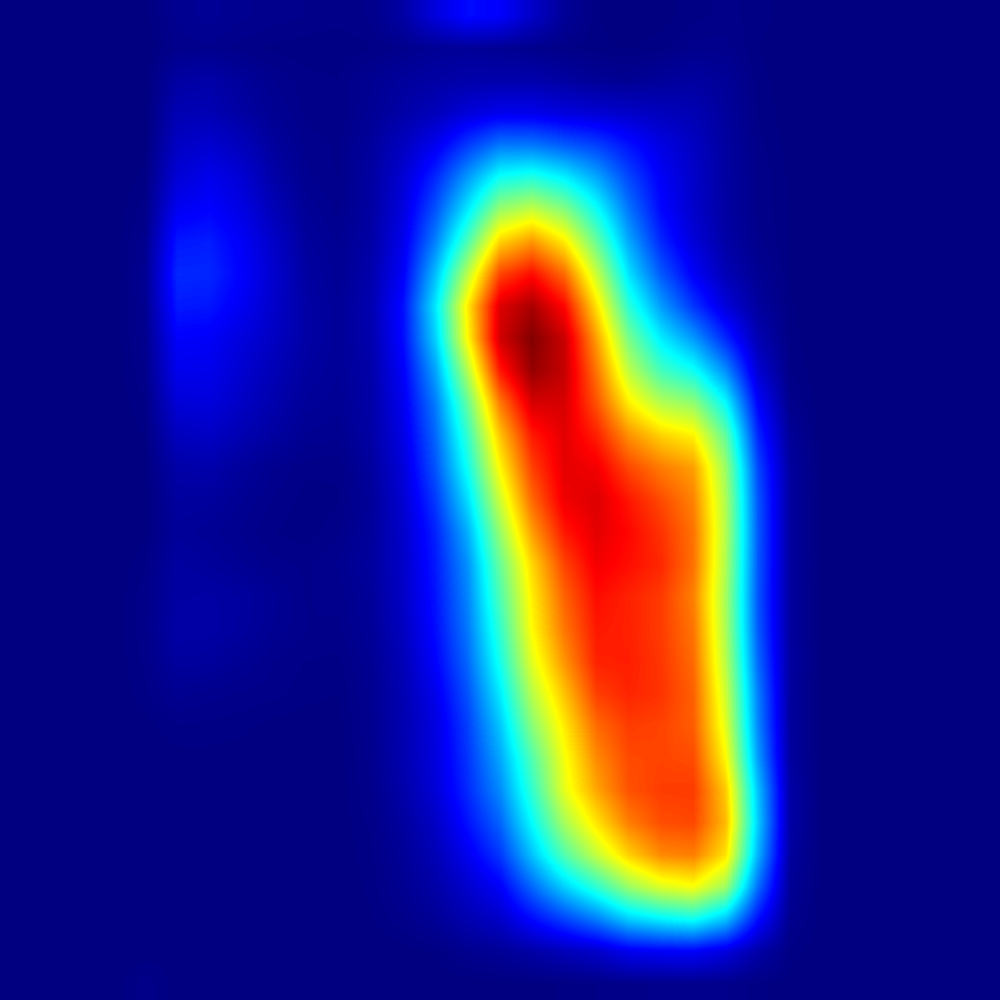

Supplement: Supplementary file 3 — Source Data File [file 41746_2022_681_MOESM3_ESM.zip › ARDA Map/Figure 5/12.png]

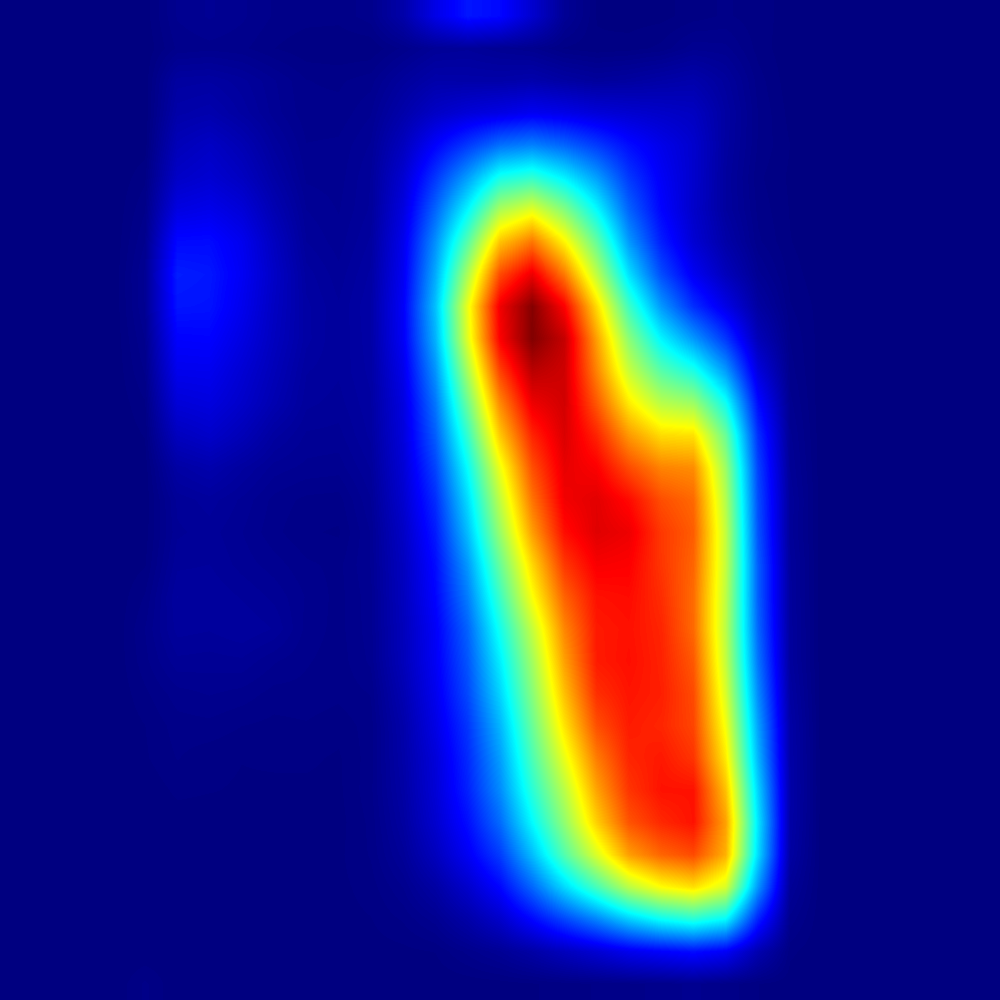

Supplement: Supplementary file 3 — Source Data File [file 41746_2022_681_MOESM3_ESM.zip › ARDA Map/Figure 5/13.png]

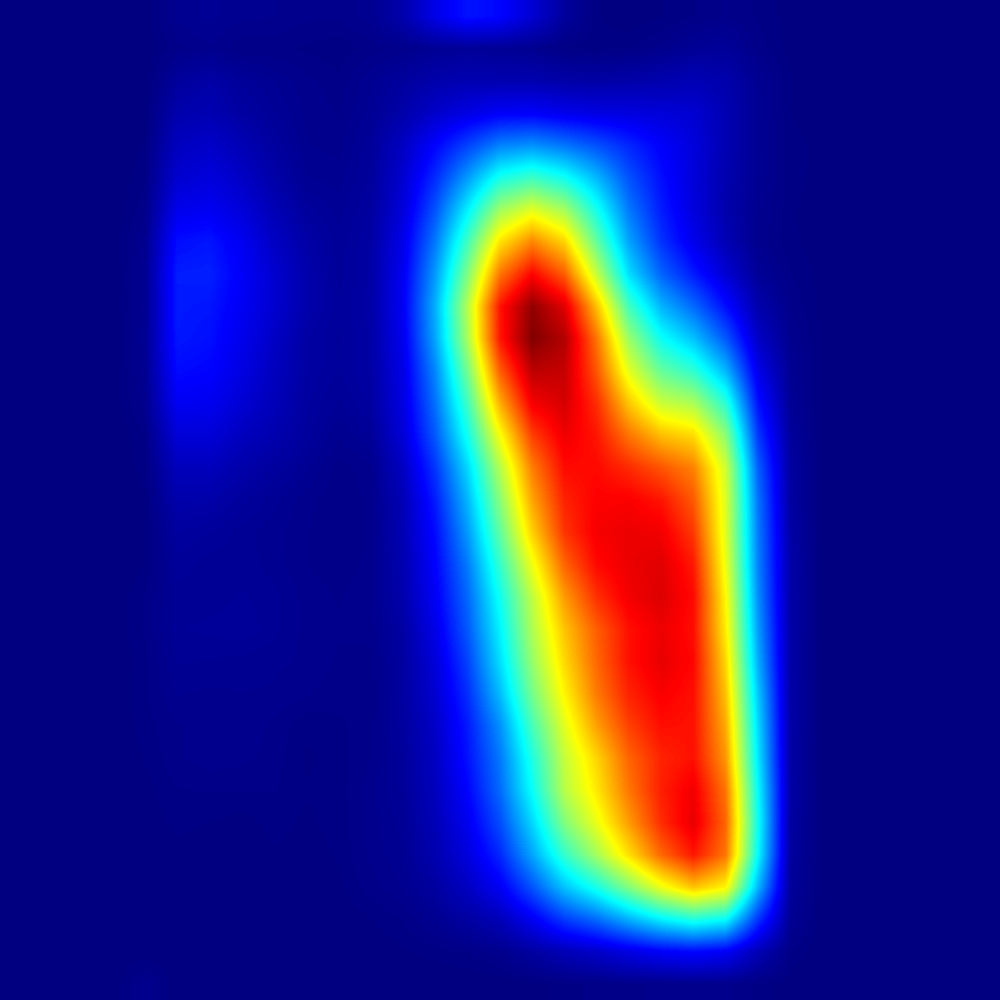

Supplement: Supplementary file 3 — Source Data File [file 41746_2022_681_MOESM3_ESM.zip › ARDA Map/Figure 5/14.png]

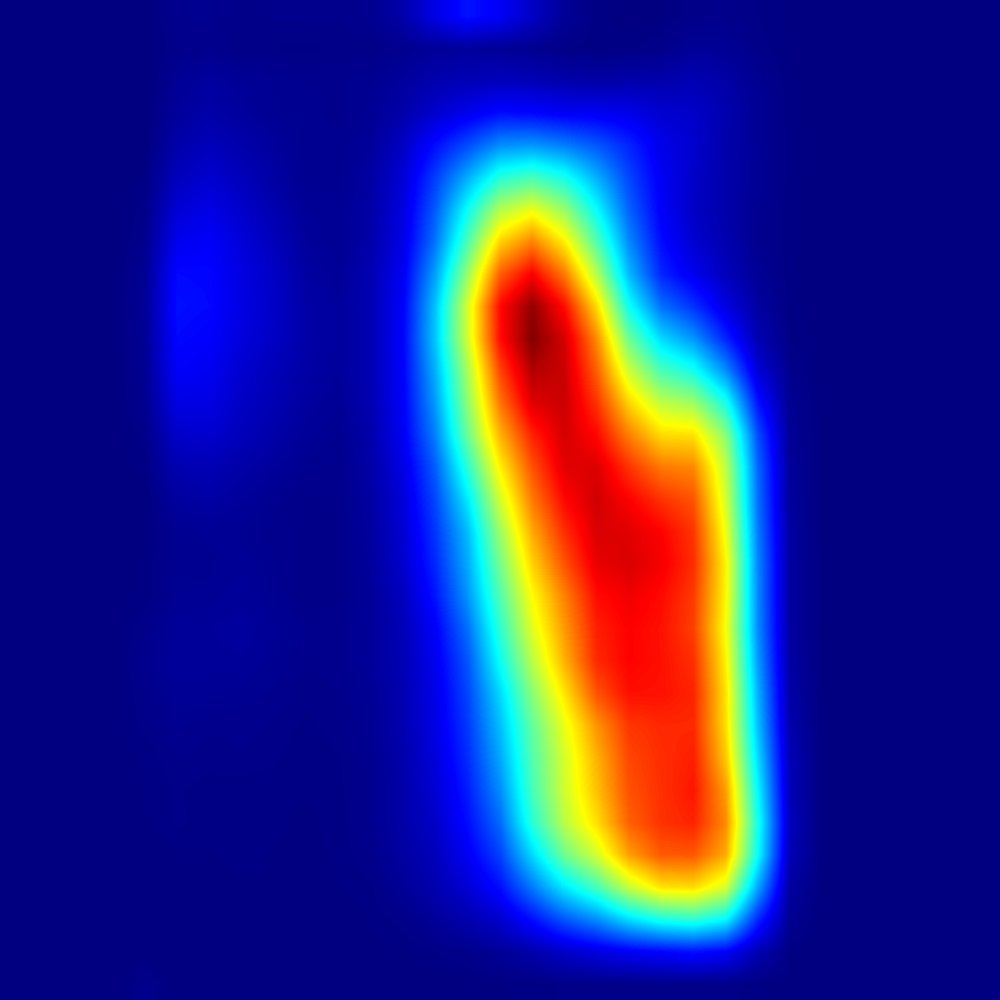

Supplement: Supplementary file 3 — Source Data File [file 41746_2022_681_MOESM3_ESM.zip › ARDA Map/Figure 5/15.png]

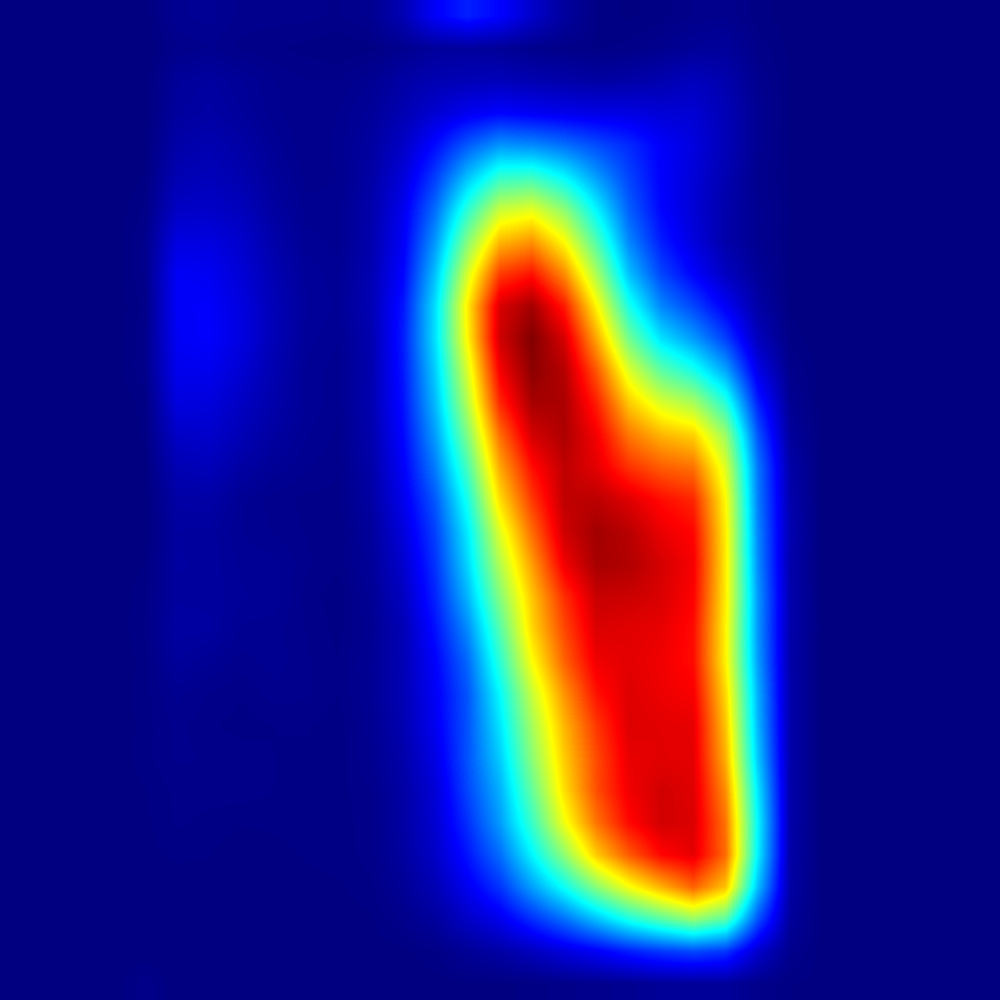

Supplement: Supplementary file 3 — Source Data File [file 41746_2022_681_MOESM3_ESM.zip › ARDA Map/Figure 5/16.png]

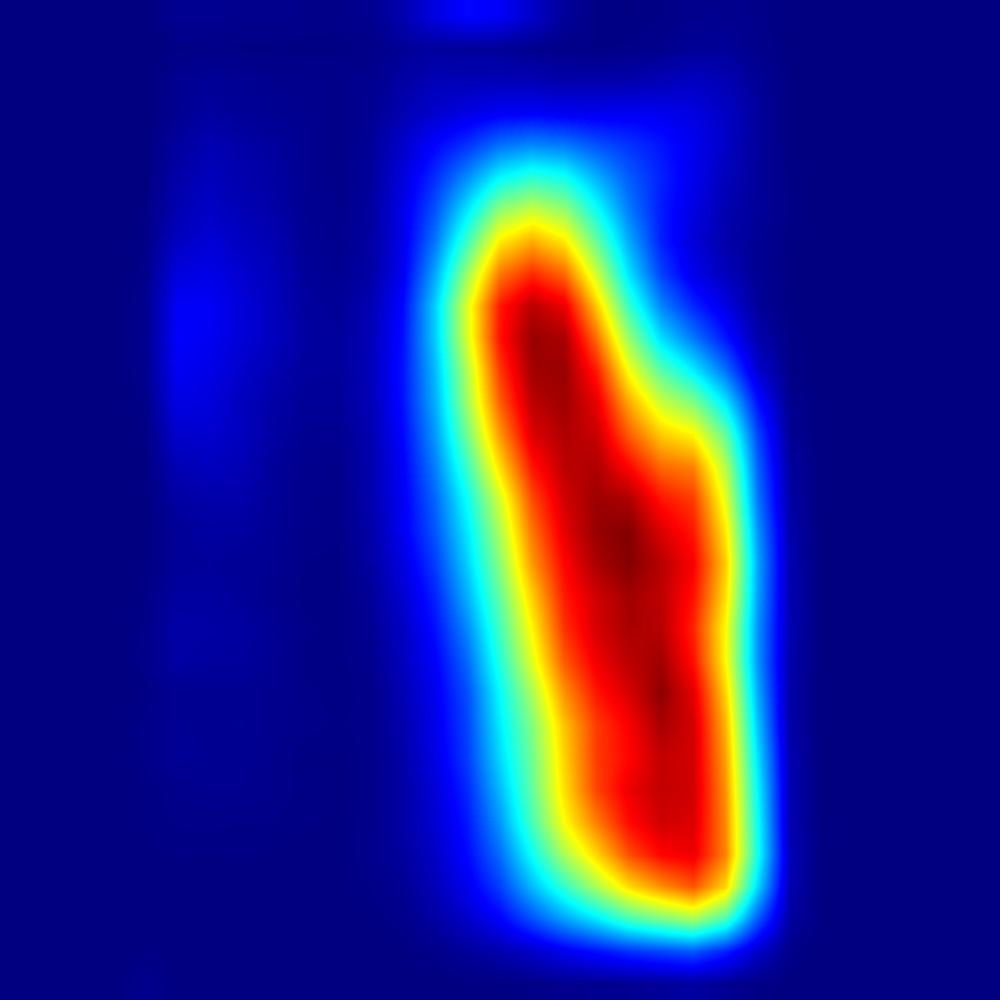

Supplement: Supplementary file 3 — Source Data File [file 41746_2022_681_MOESM3_ESM.zip › ARDA Map/Figure 5/17.png]

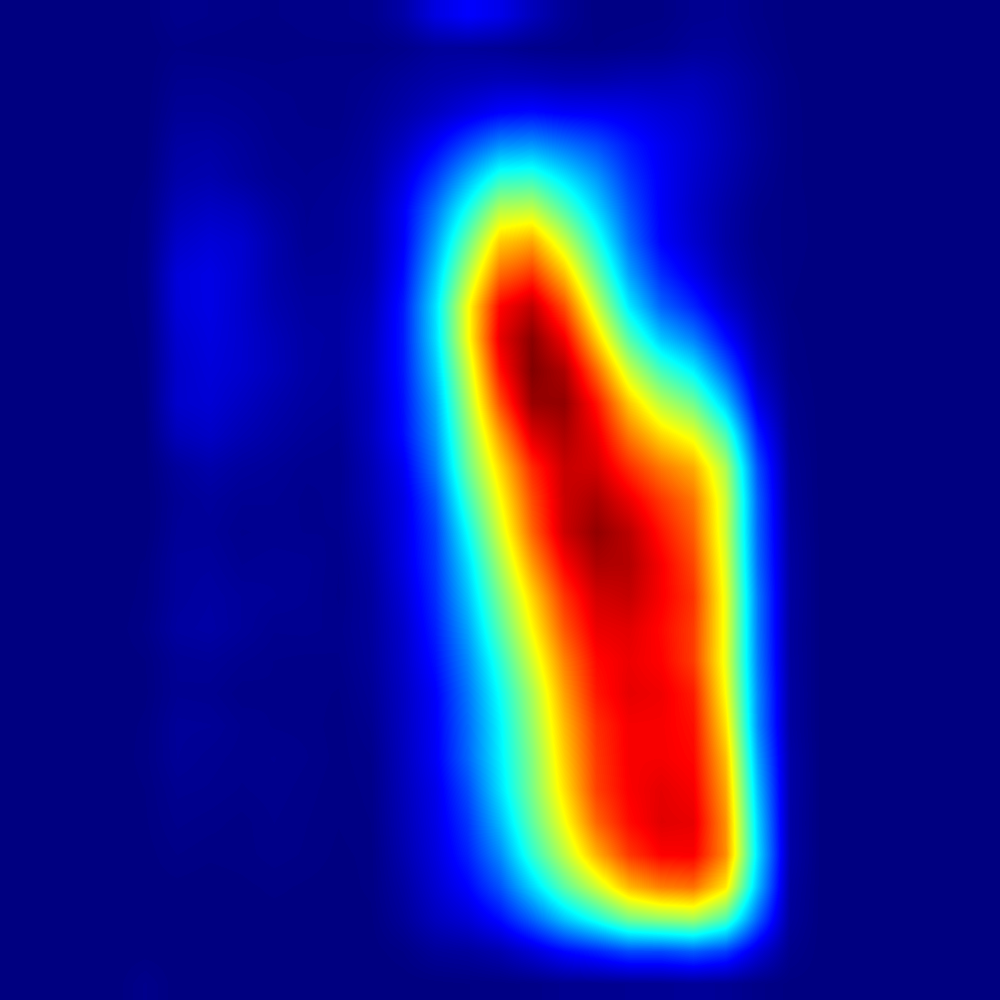

Supplement: Supplementary file 3 — Source Data File [file 41746_2022_681_MOESM3_ESM.zip › ARDA Map/Figure 5/18.png]

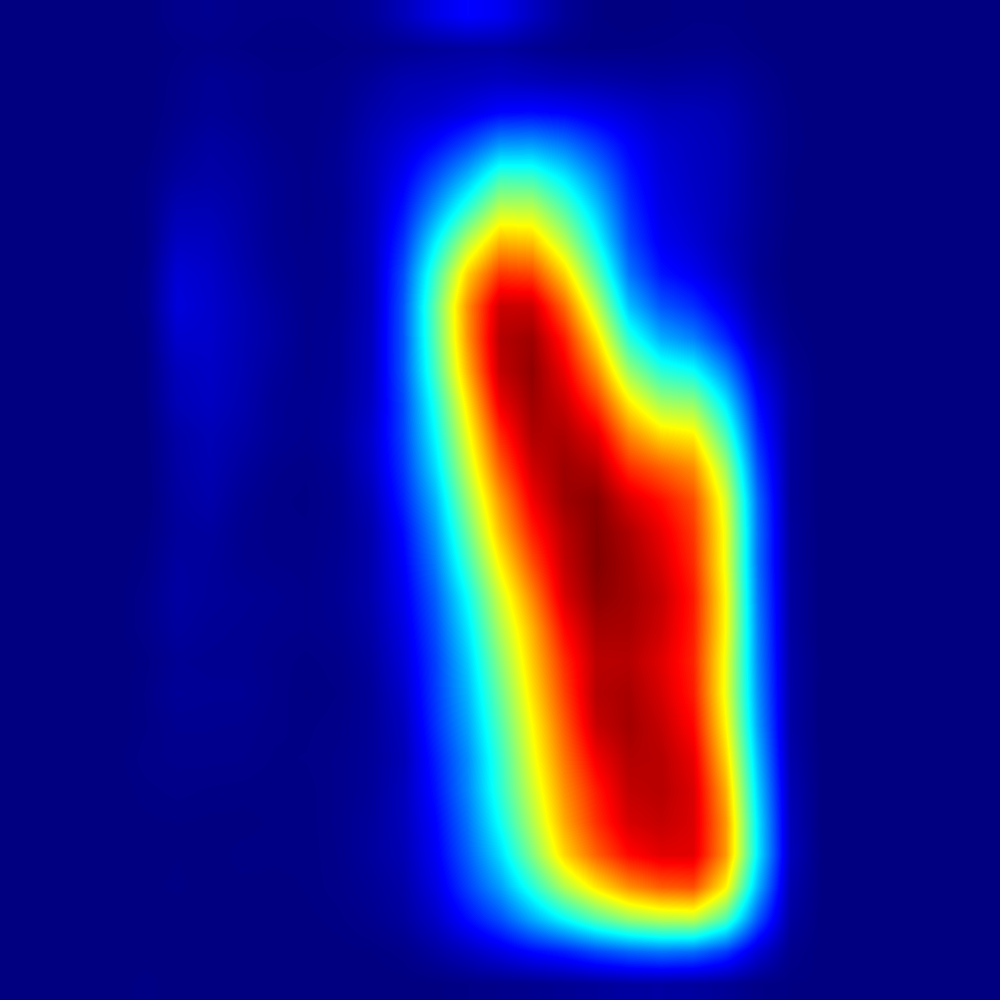

Supplement: Supplementary file 3 — Source Data File [file 41746_2022_681_MOESM3_ESM.zip › ARDA Map/Figure 5/19.png]

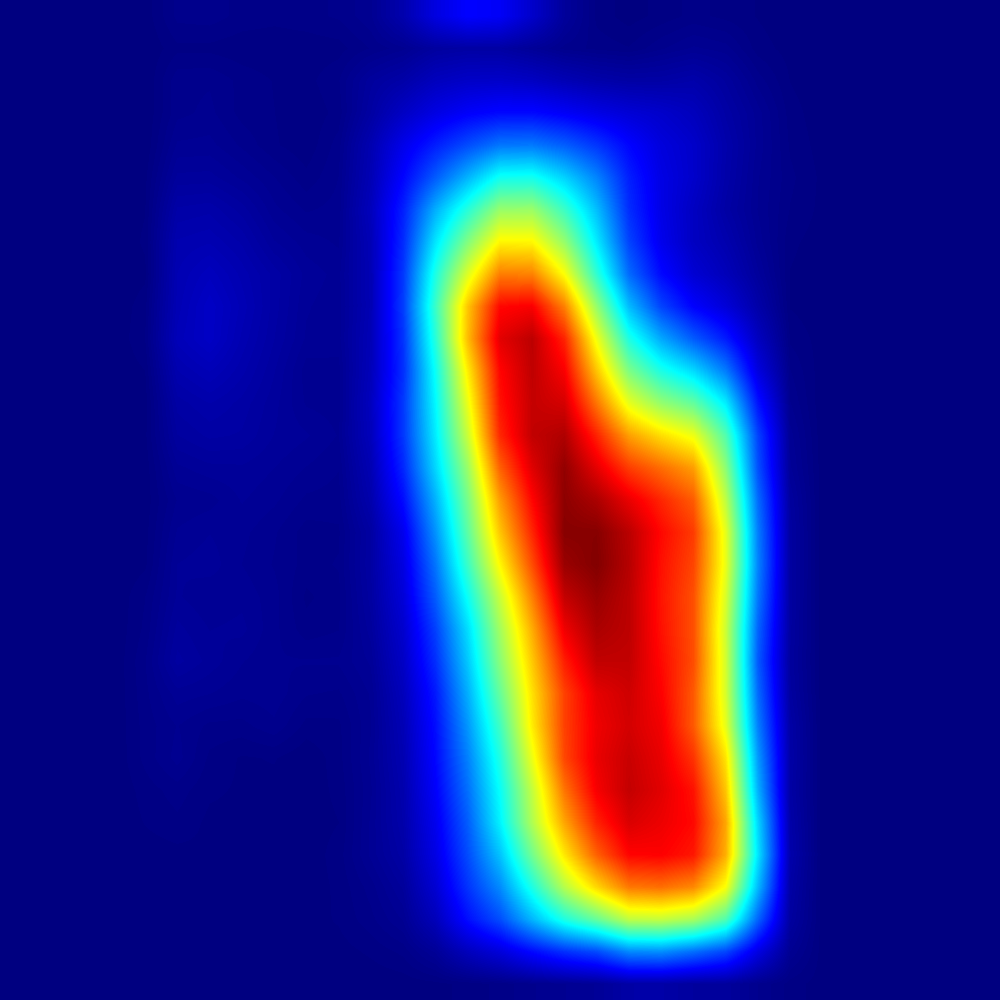

Supplement: Supplementary file 3 — Source Data File [file 41746_2022_681_MOESM3_ESM.zip › ARDA Map/Figure 5/20.png]

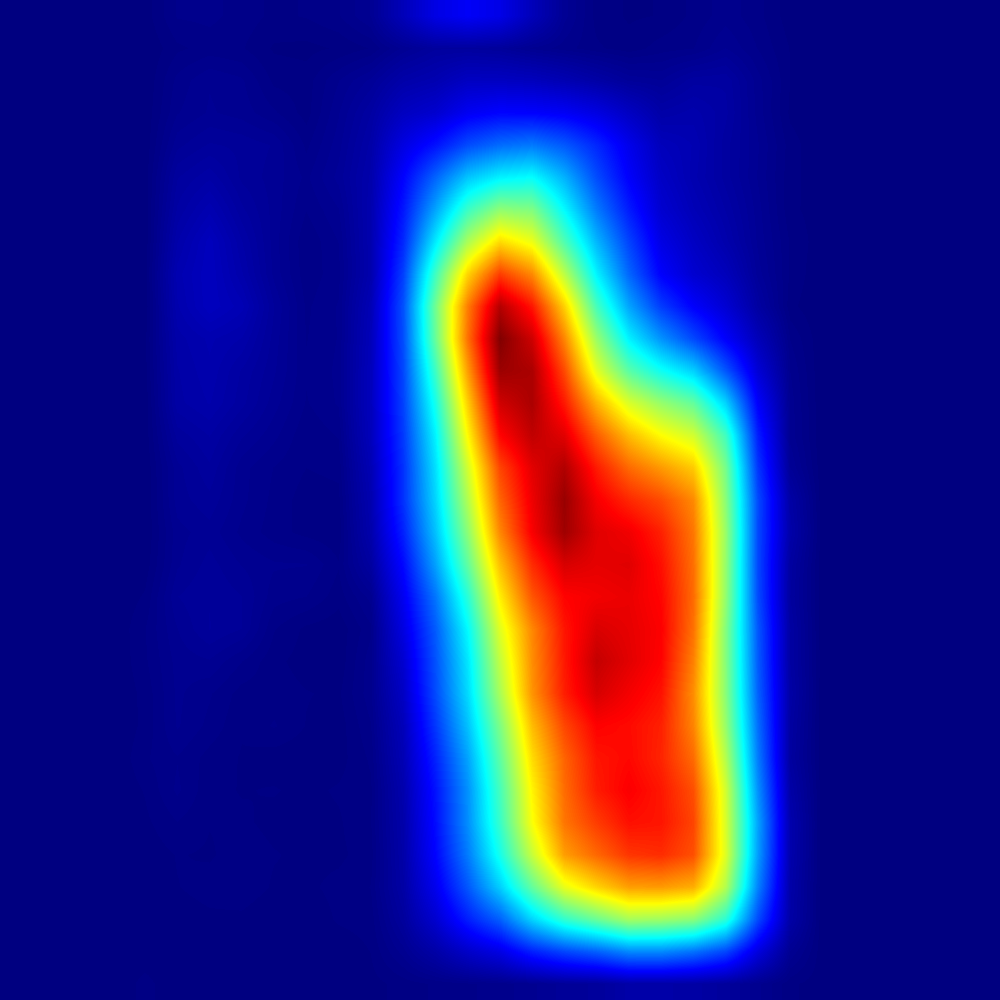

Supplement: Supplementary file 3 — Source Data File [file 41746_2022_681_MOESM3_ESM.zip › ARDA Map/Figure 5/21.png]

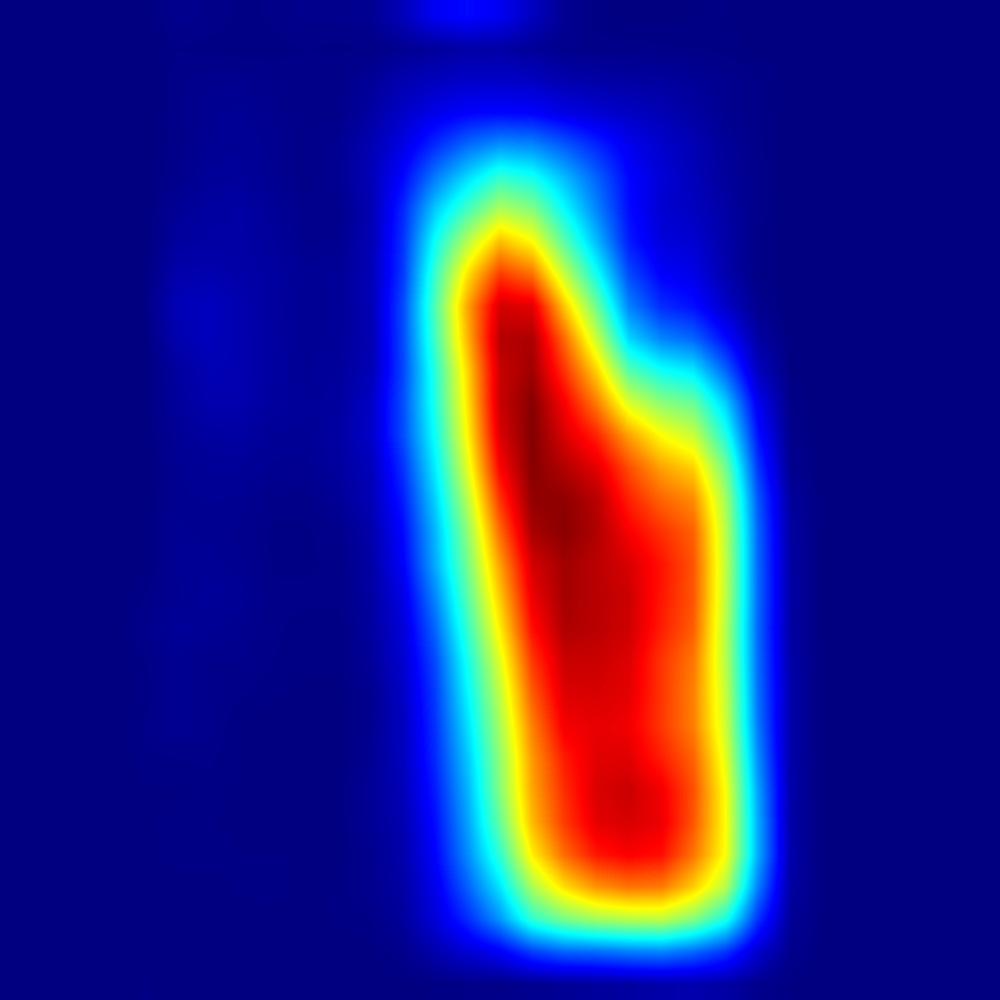

Supplement: Supplementary file 3 — Source Data File [file 41746_2022_681_MOESM3_ESM.zip › ARDA Map/Figure 5/22.png]

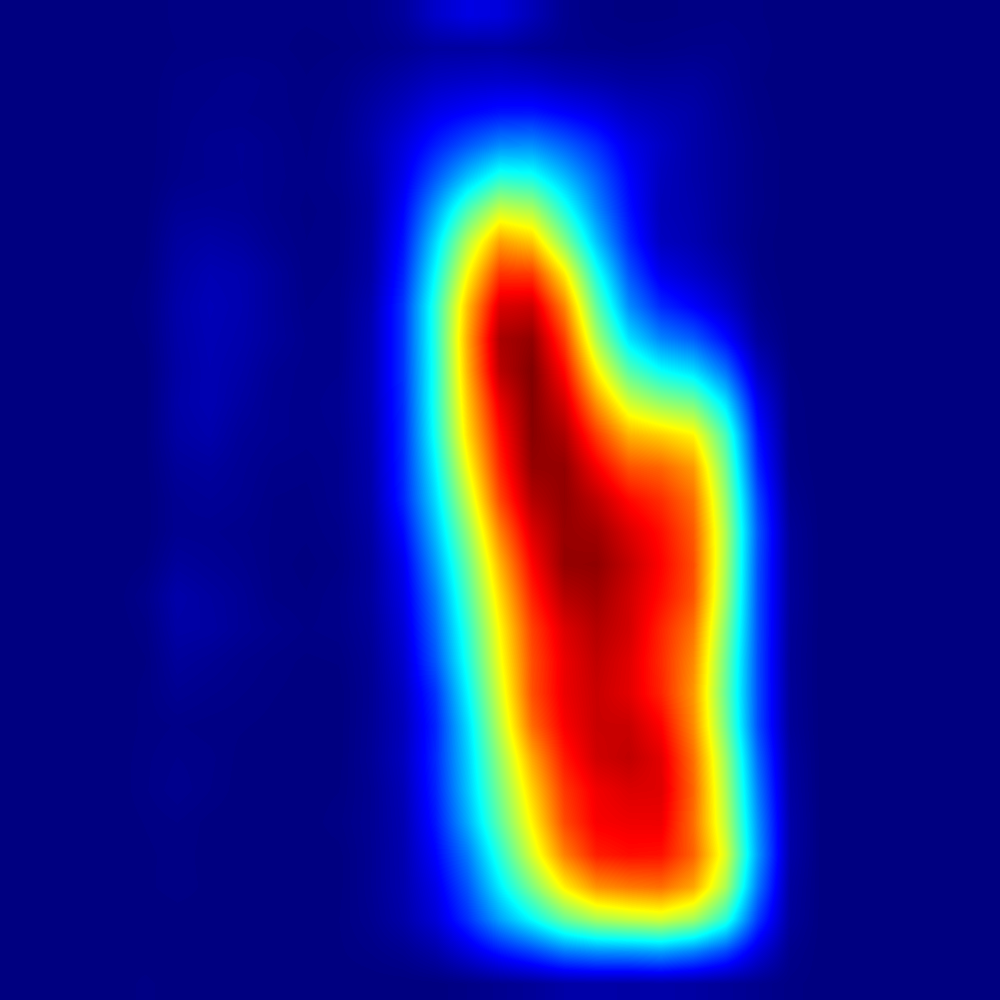

Supplement: Supplementary file 3 — Source Data File [file 41746_2022_681_MOESM3_ESM.zip › ARDA Map/Figure 5/23.png]

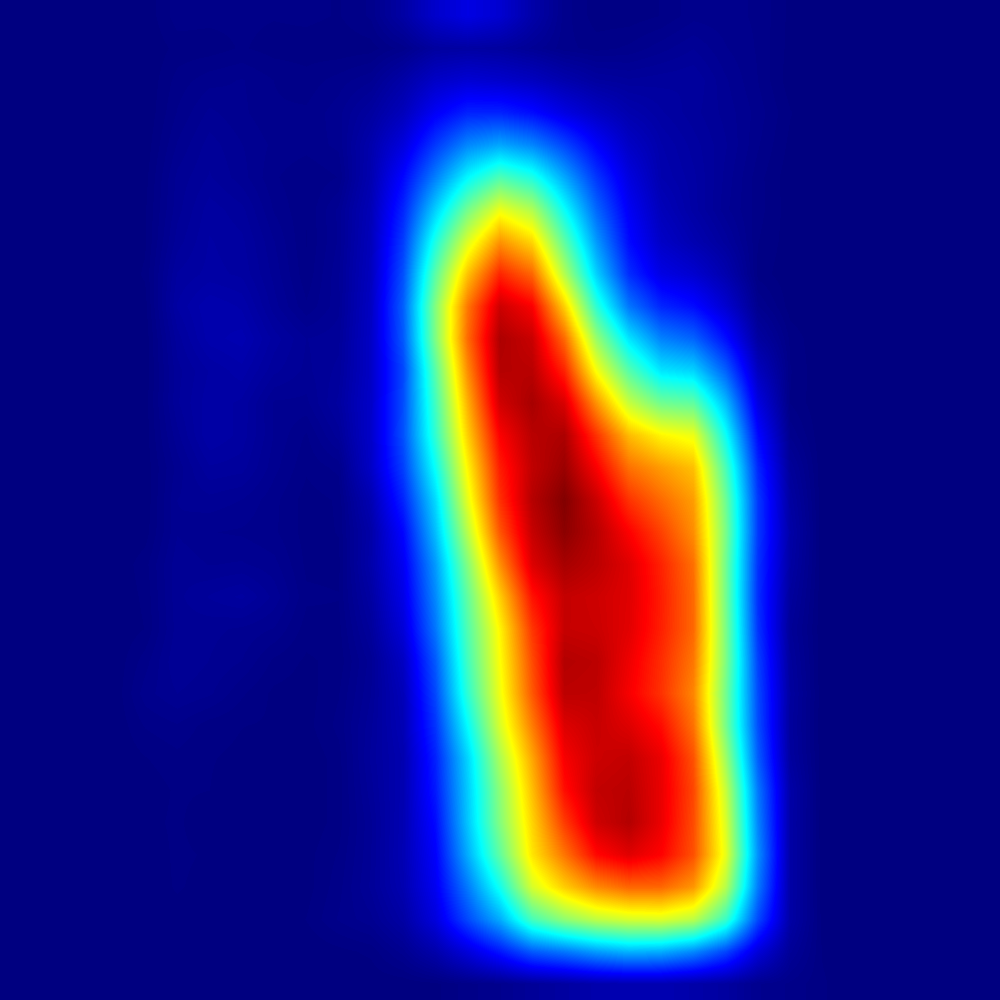

Supplement: Supplementary file 3 — Source Data File [file 41746_2022_681_MOESM3_ESM.zip › ARDA Map/Figure 5/24.png]

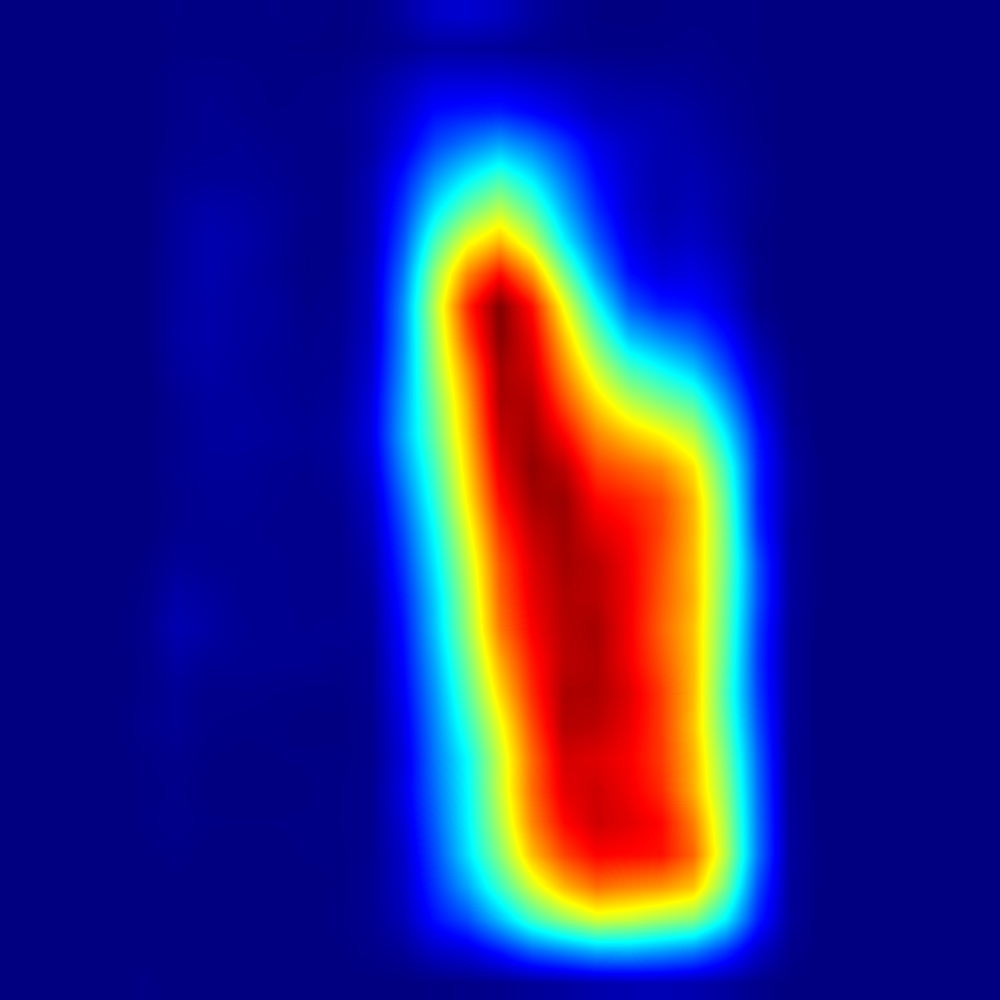

Supplement: Supplementary file 3 — Source Data File [file 41746_2022_681_MOESM3_ESM.zip › ARDA Map/Figure 5/25.png]

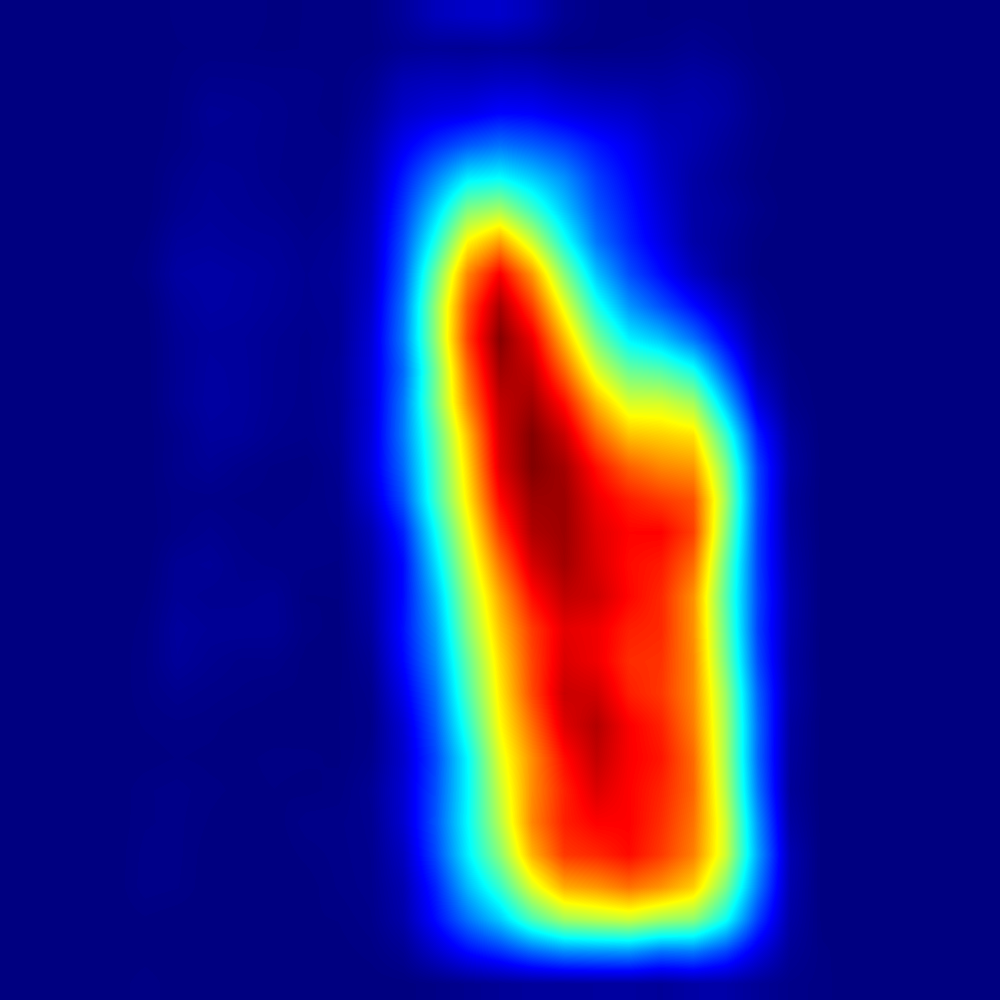

Supplement: Supplementary file 3 — Source Data File [file 41746_2022_681_MOESM3_ESM.zip › ARDA Map/Figure 5/26.png]

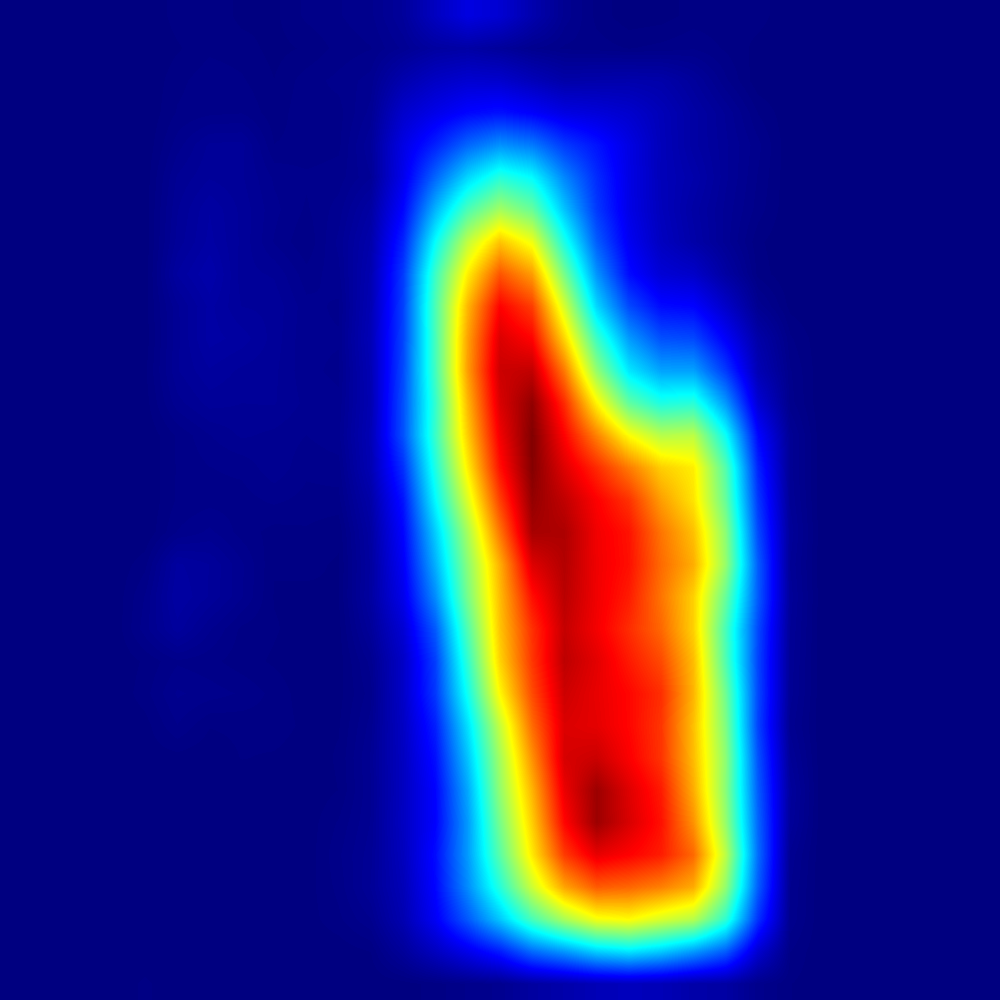

Supplement: Supplementary file 3 — Source Data File [file 41746_2022_681_MOESM3_ESM.zip › ARDA Map/Figure 5/27.png]

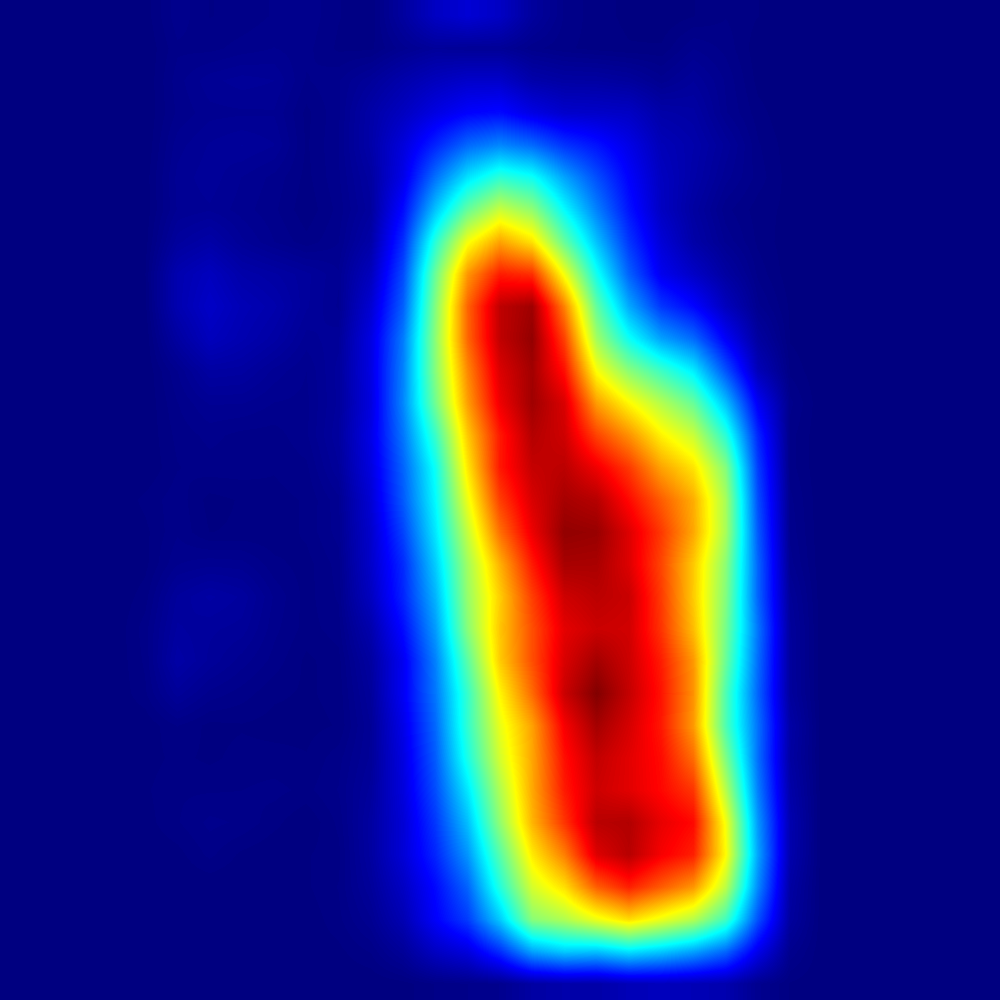

Supplement: Supplementary file 3 — Source Data File [file 41746_2022_681_MOESM3_ESM.zip › ARDA Map/Figure 5/28.png]

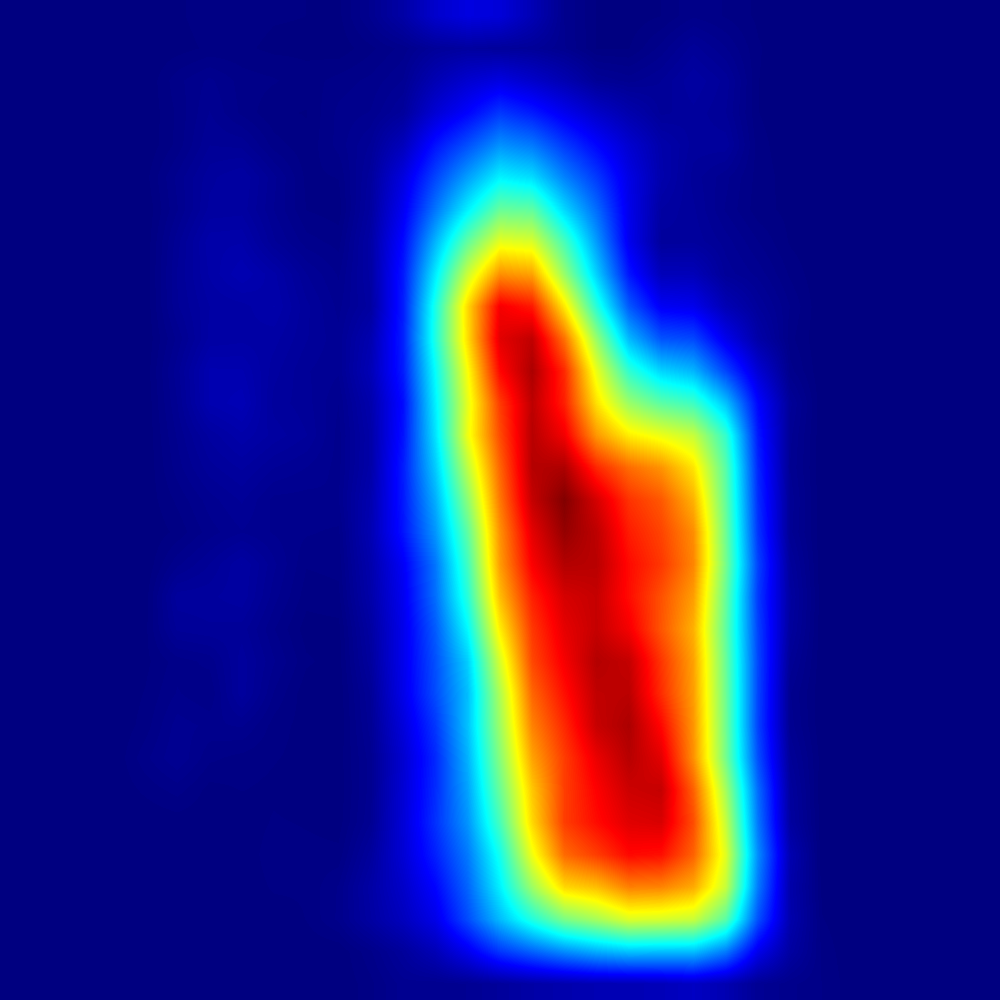

Supplement: Supplementary file 3 — Source Data File [file 41746_2022_681_MOESM3_ESM.zip › ARDA Map/Figure 5/29.png]

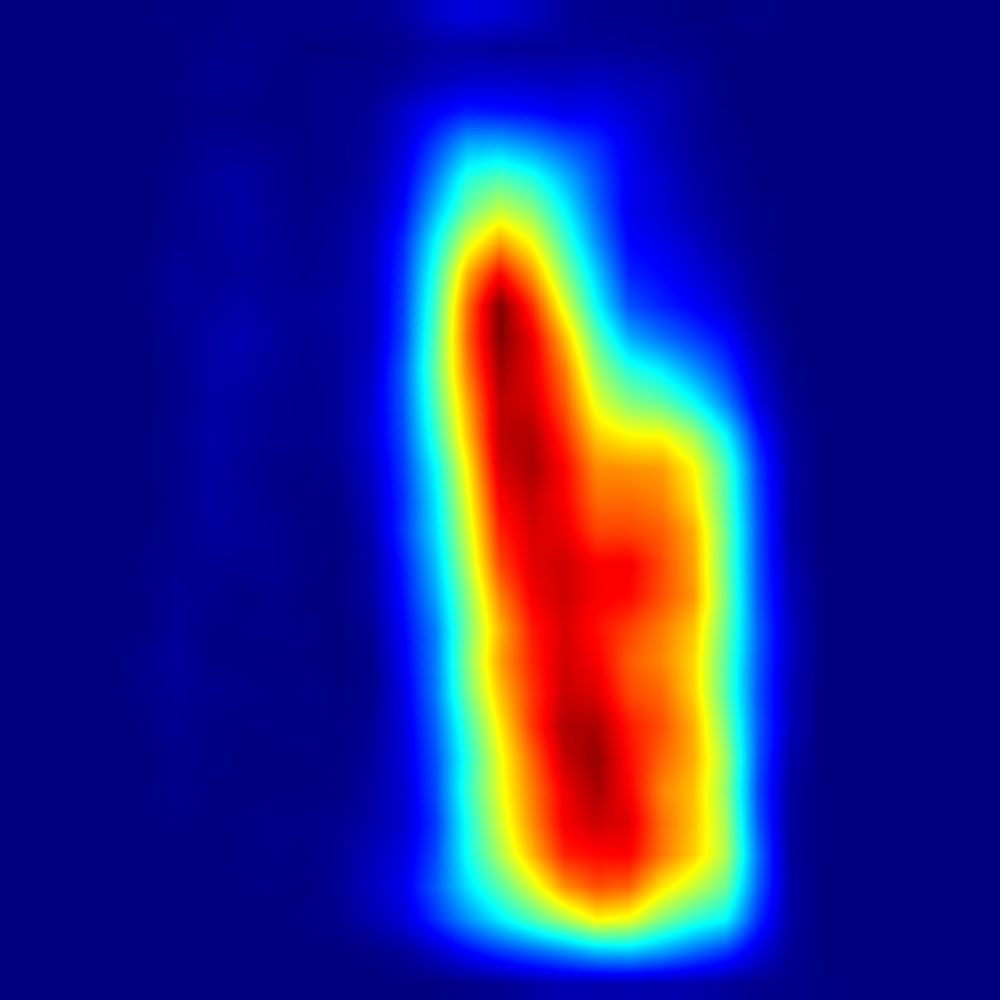

Supplement: Supplementary file 3 — Source Data File [file 41746_2022_681_MOESM3_ESM.zip › ARDA Map/Figure 5/30.png]

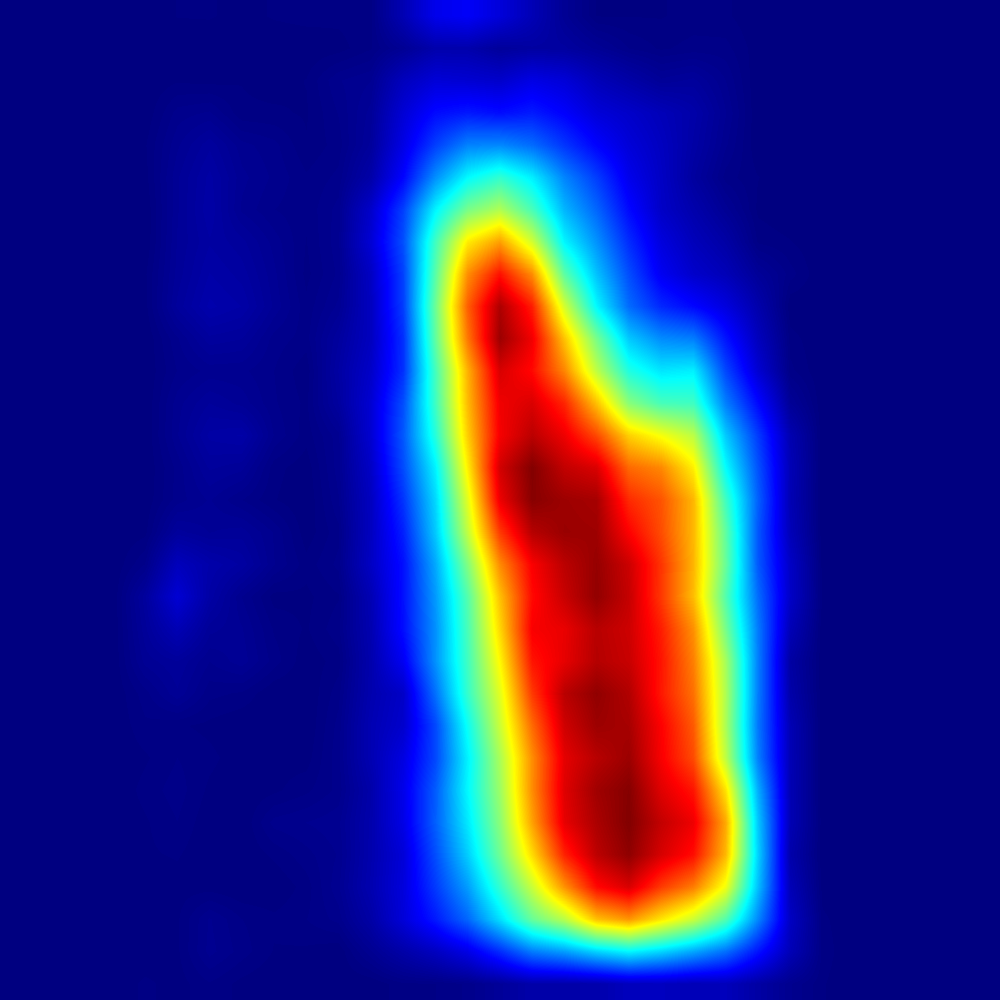

Supplement: Supplementary file 3 — Source Data File [file 41746_2022_681_MOESM3_ESM.zip › ARDA Map/Figure 5/31.png]

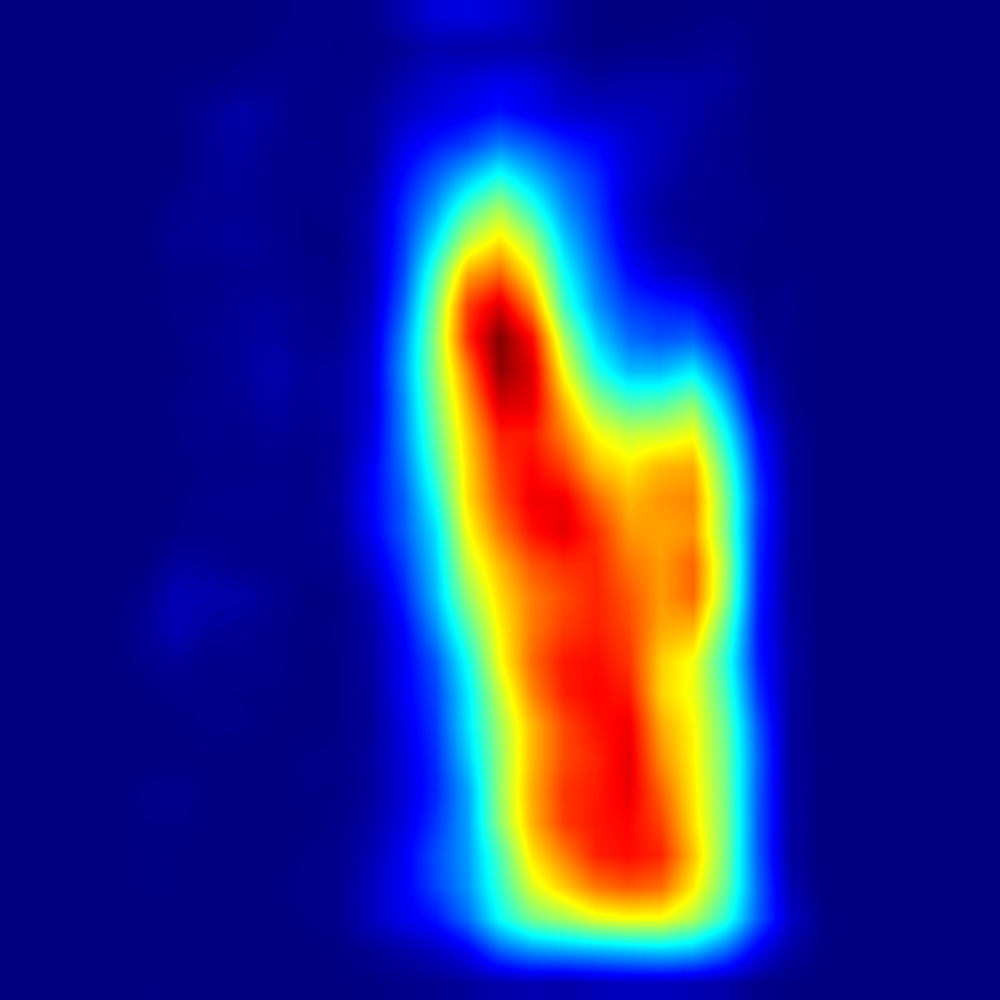

Supplement: Supplementary file 3 — Source Data File [file 41746_2022_681_MOESM3_ESM.zip › ARDA Map/Figure 5/32.png]

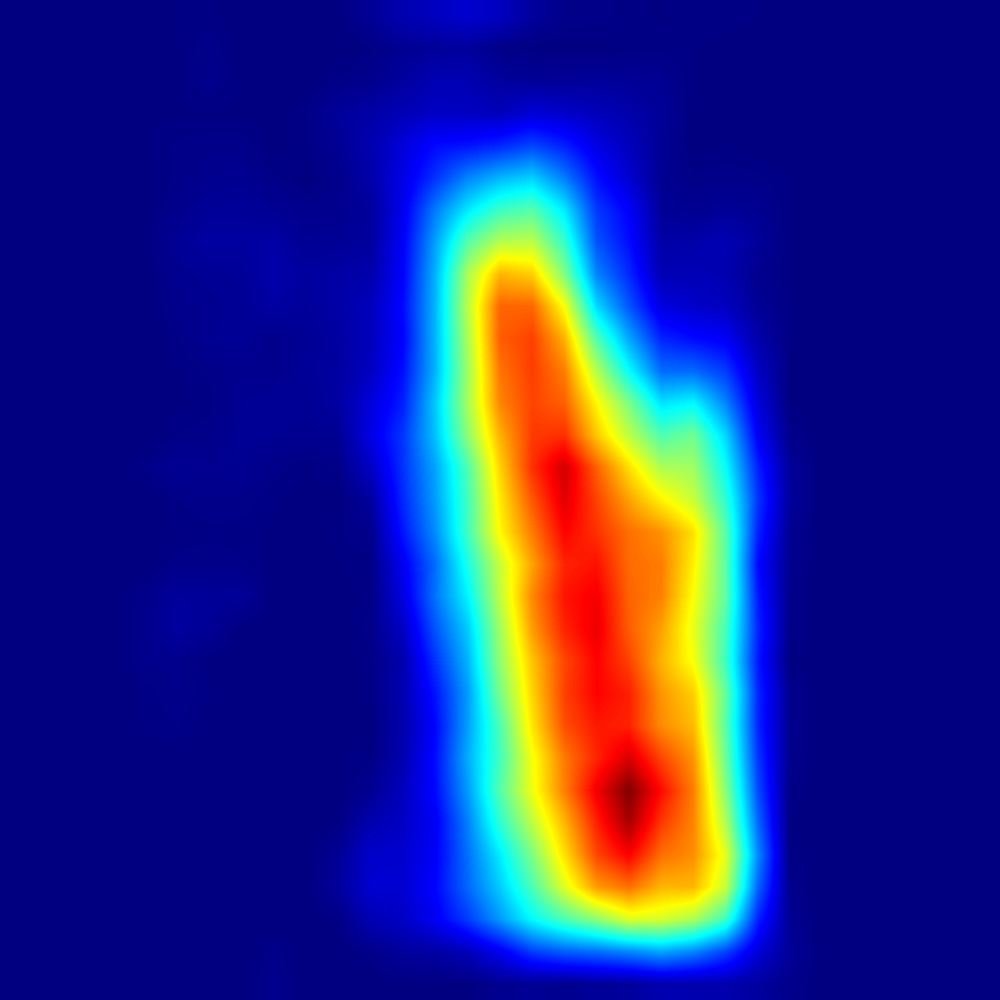

Supplement: Supplementary file 3 — Source Data File [file 41746_2022_681_MOESM3_ESM.zip › ARDA Map/Figure 5/33.png]

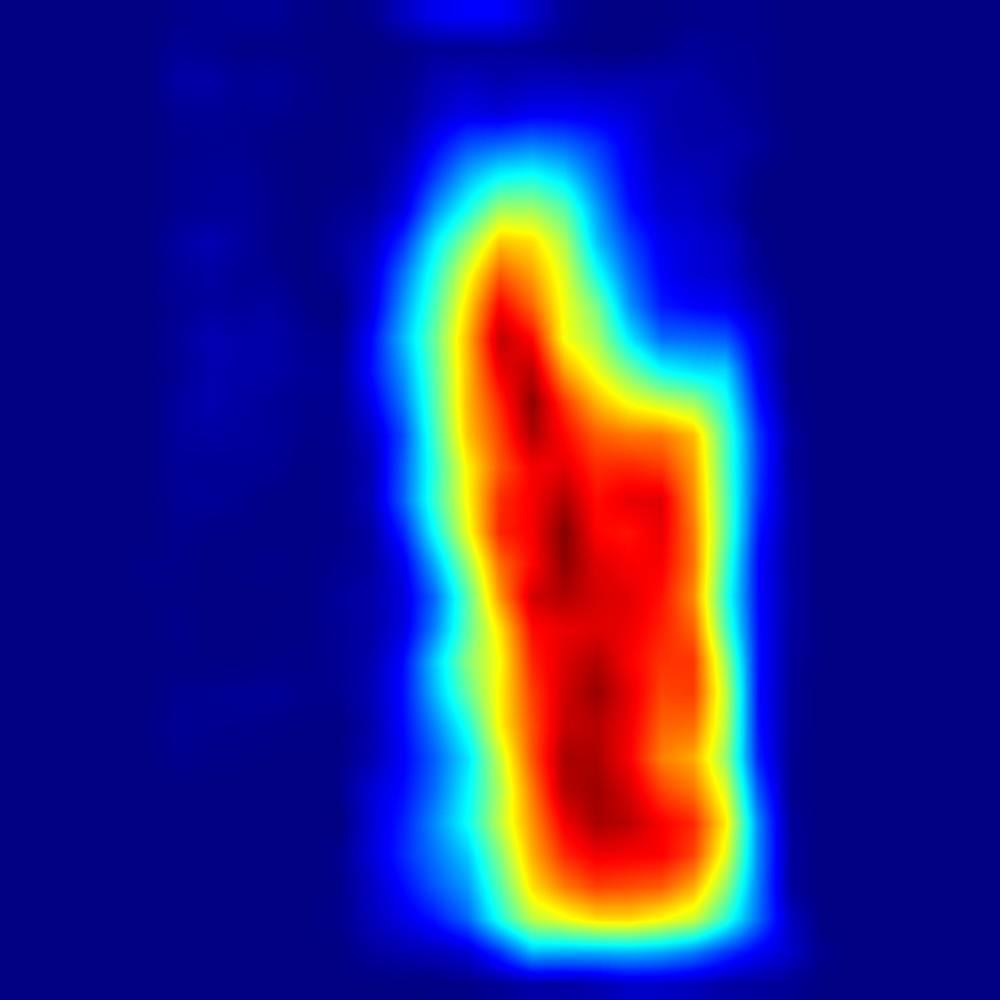

Supplement: Supplementary file 3 — Source Data File [file 41746_2022_681_MOESM3_ESM.zip › ARDA Map/Figure 5/34.png]

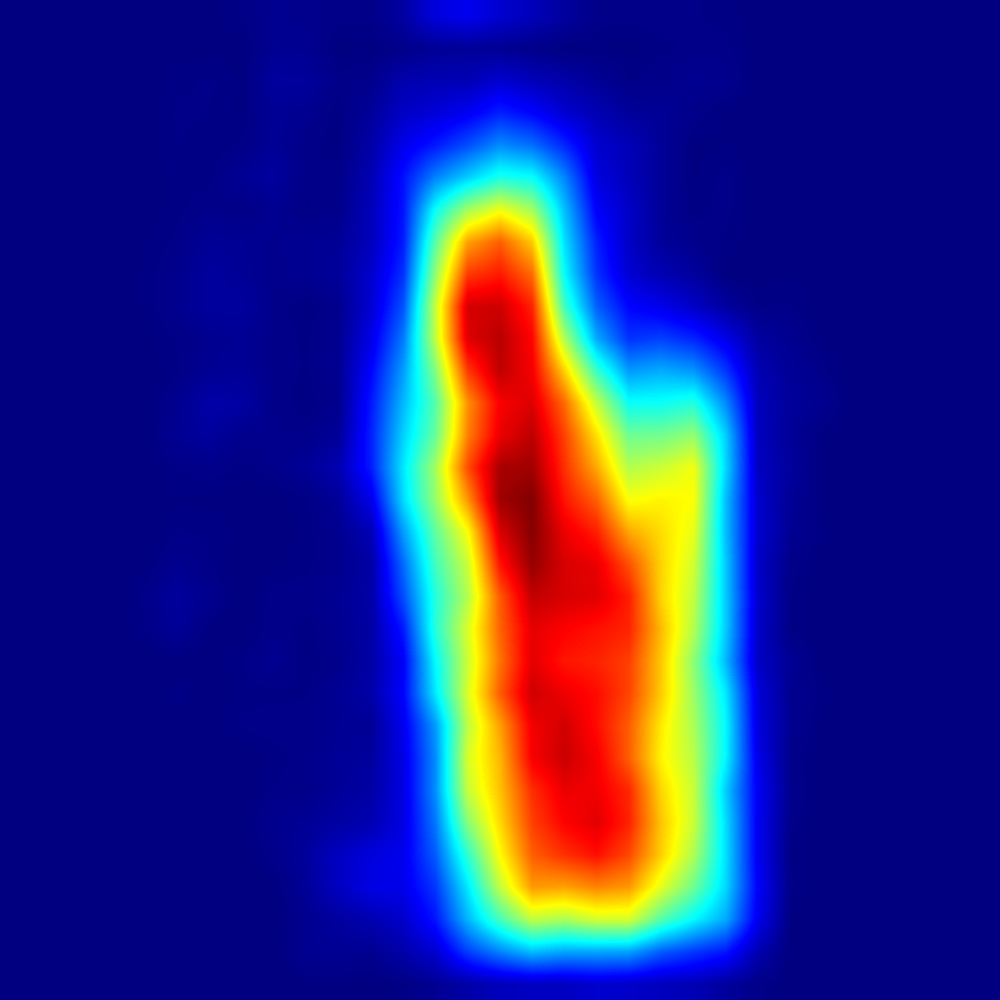

Supplement: Supplementary file 3 — Source Data File [file 41746_2022_681_MOESM3_ESM.zip › ARDA Map/Figure 5/35.png]

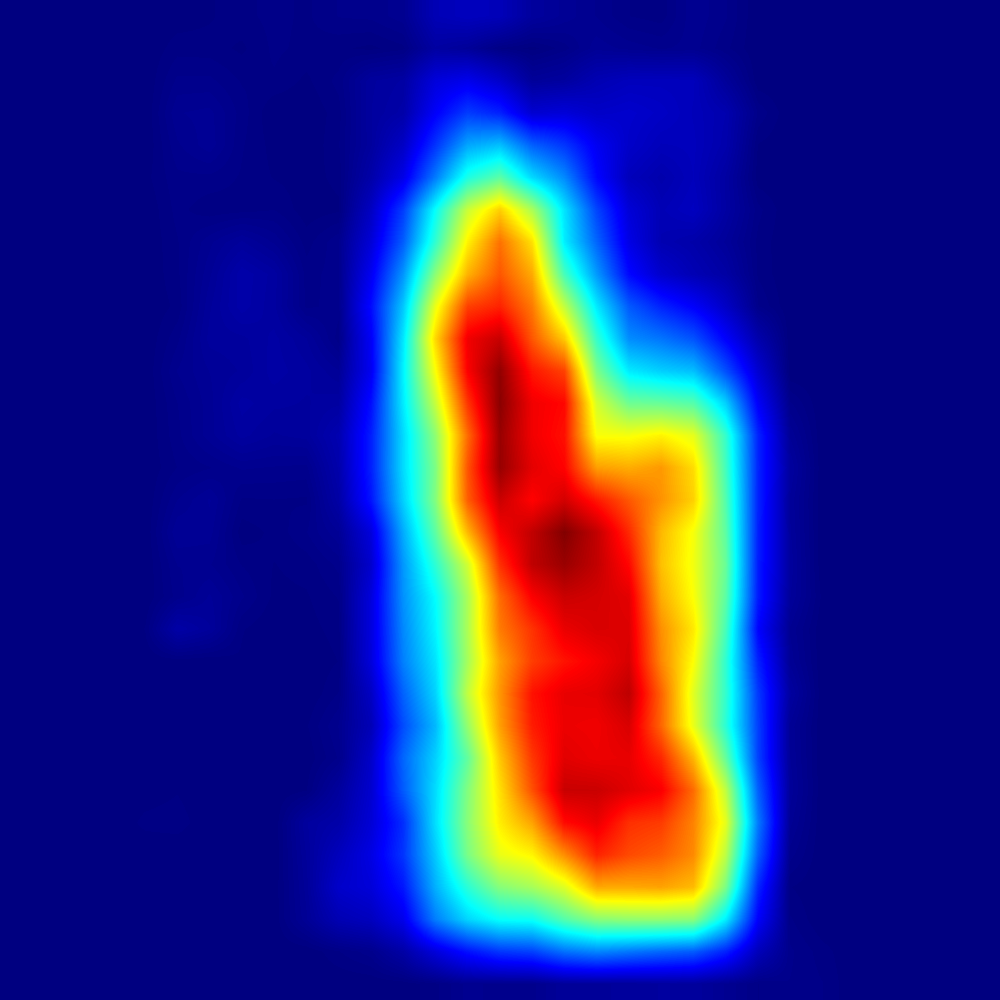

Supplement: Supplementary file 3 — Source Data File [file 41746_2022_681_MOESM3_ESM.zip › ARDA Map/Figure 5/36.png]

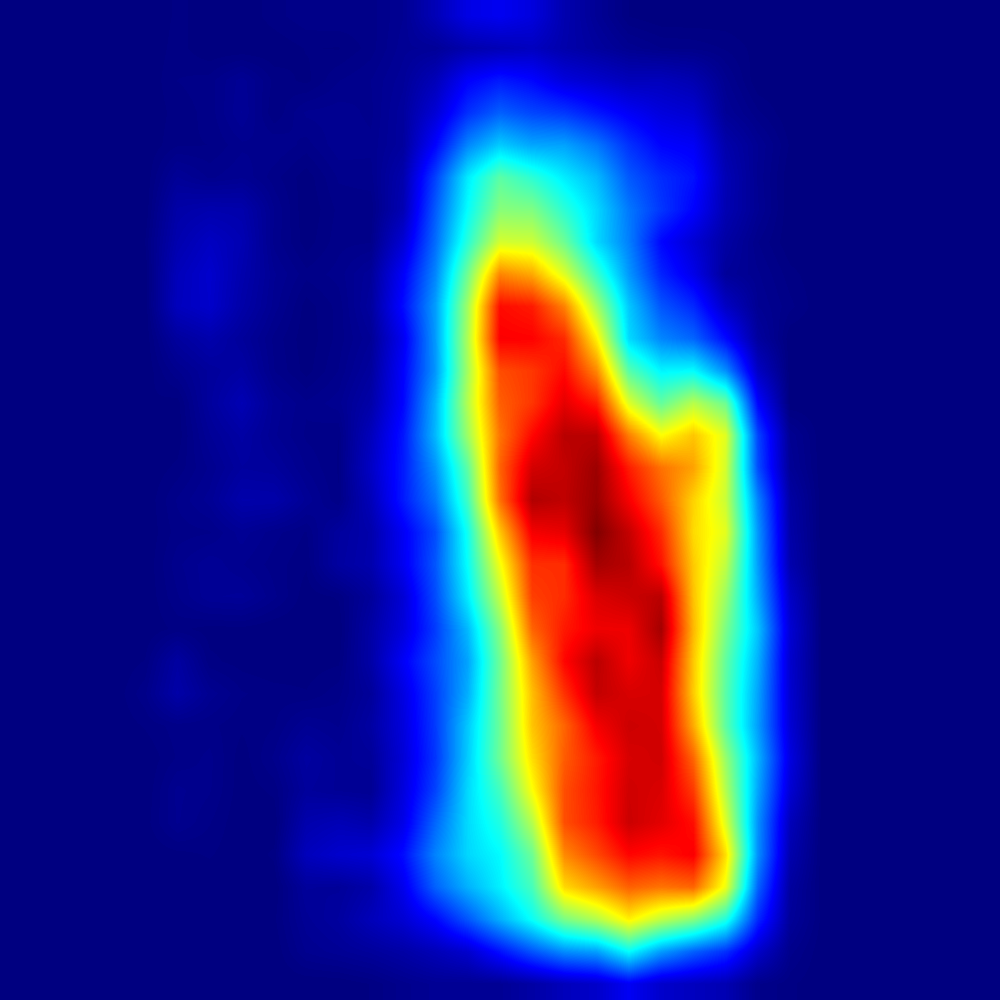

Supplement: Supplementary file 3 — Source Data File [file 41746_2022_681_MOESM3_ESM.zip › ARDA Map/Figure 5/37.png]

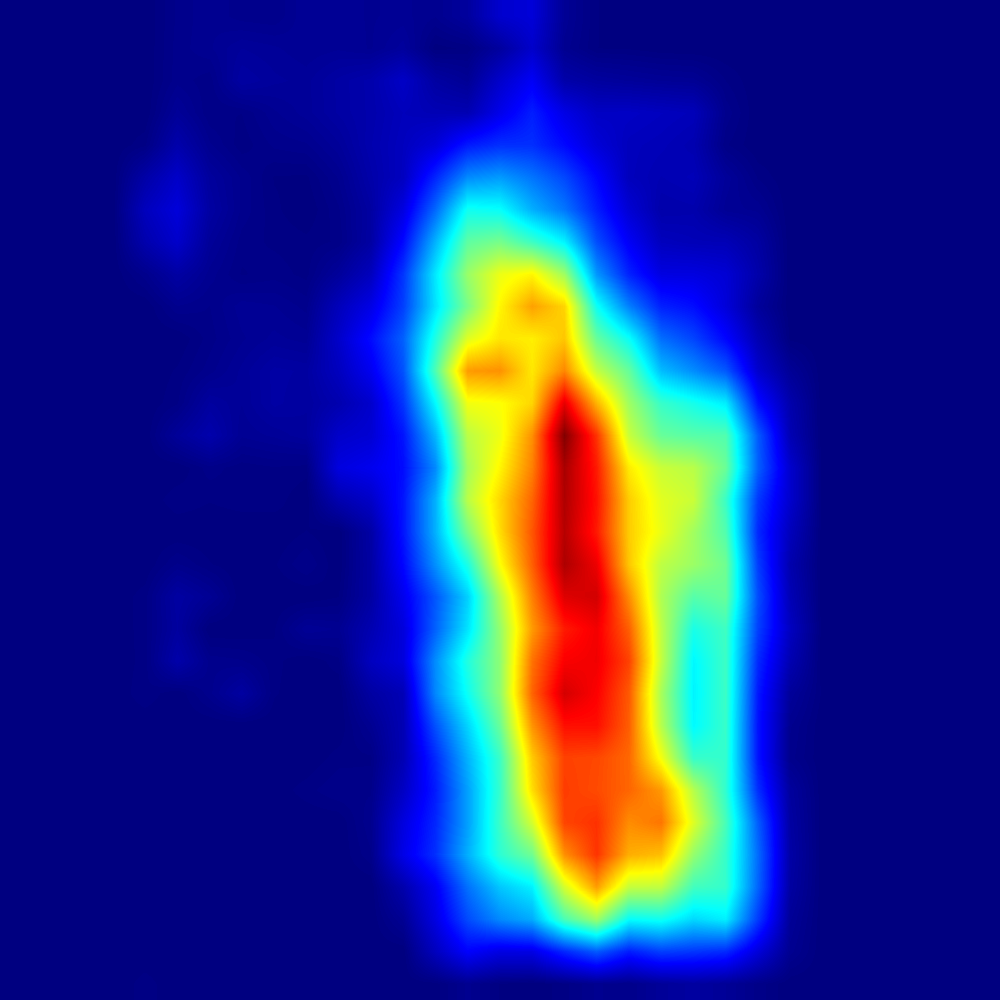

Supplement: Supplementary file 3 — Source Data File [file 41746_2022_681_MOESM3_ESM.zip › ARDA Map/Figure 5/38.png]

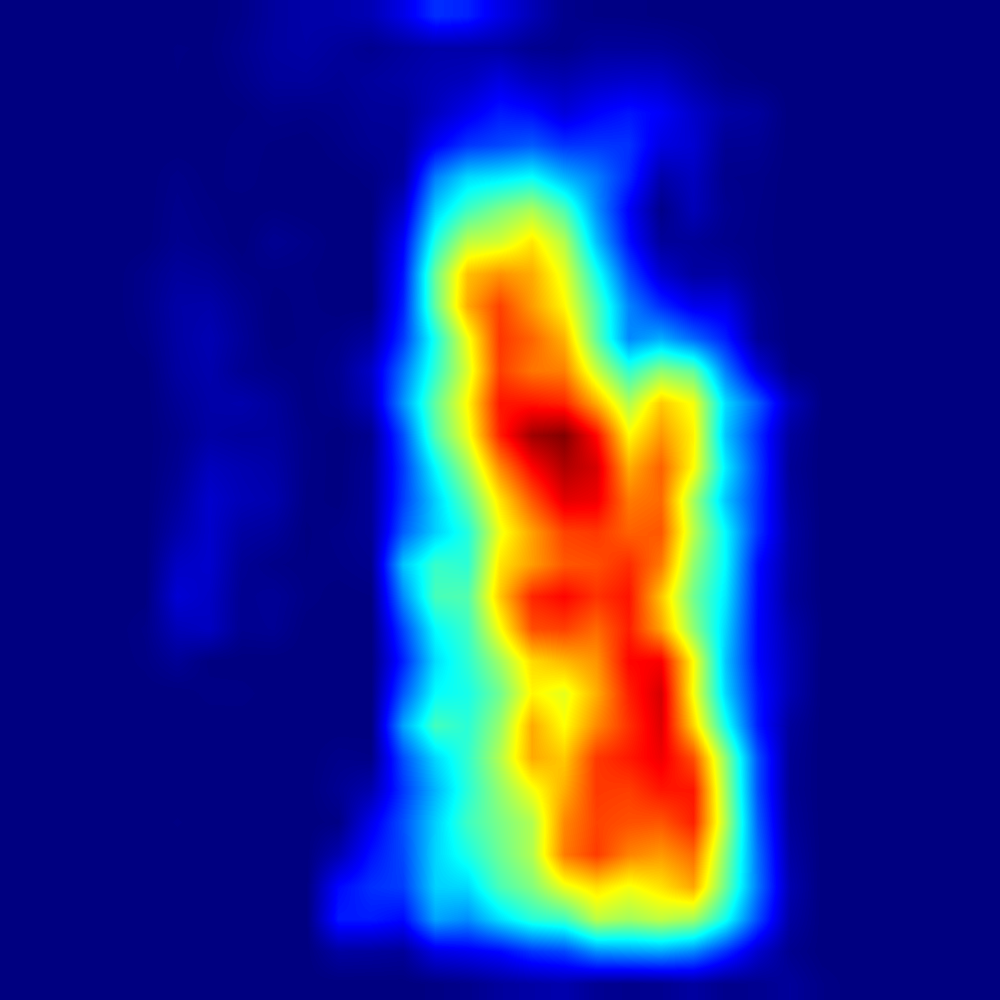

Supplement: Supplementary file 3 — Source Data File [file 41746_2022_681_MOESM3_ESM.zip › ARDA Map/Figure 5/39.png]

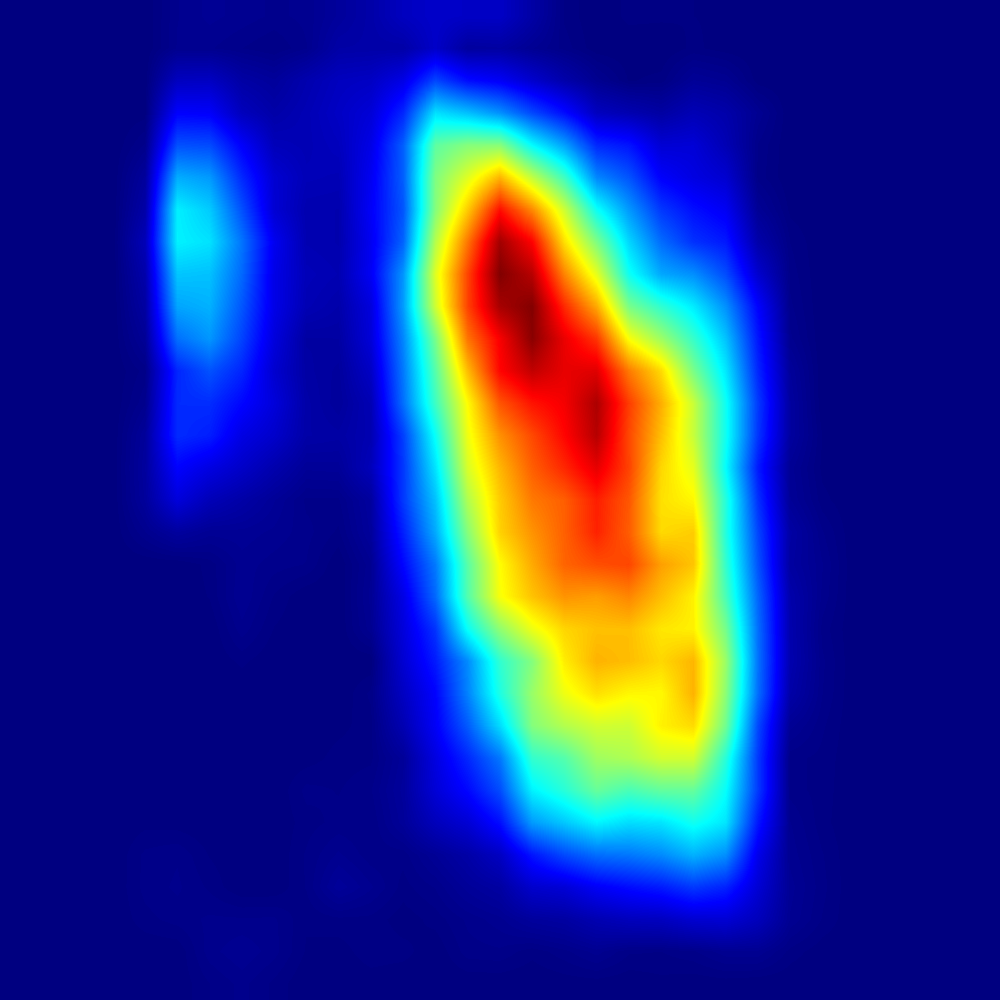

Supplement: Supplementary file 3 — Source Data File [file 41746_2022_681_MOESM3_ESM.zip › ARDA Map/Figure 5/4.png]

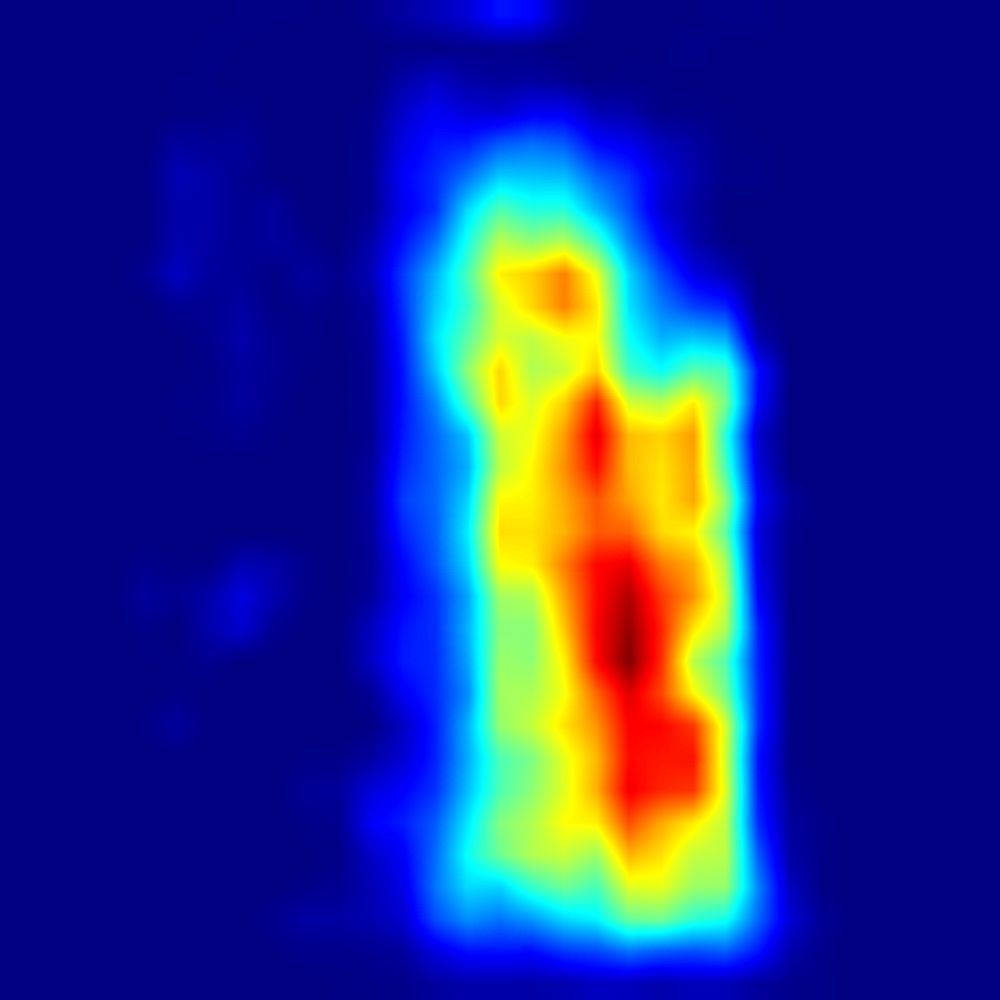

Supplement: Supplementary file 3 — Source Data File [file 41746_2022_681_MOESM3_ESM.zip › ARDA Map/Figure 5/40.png]

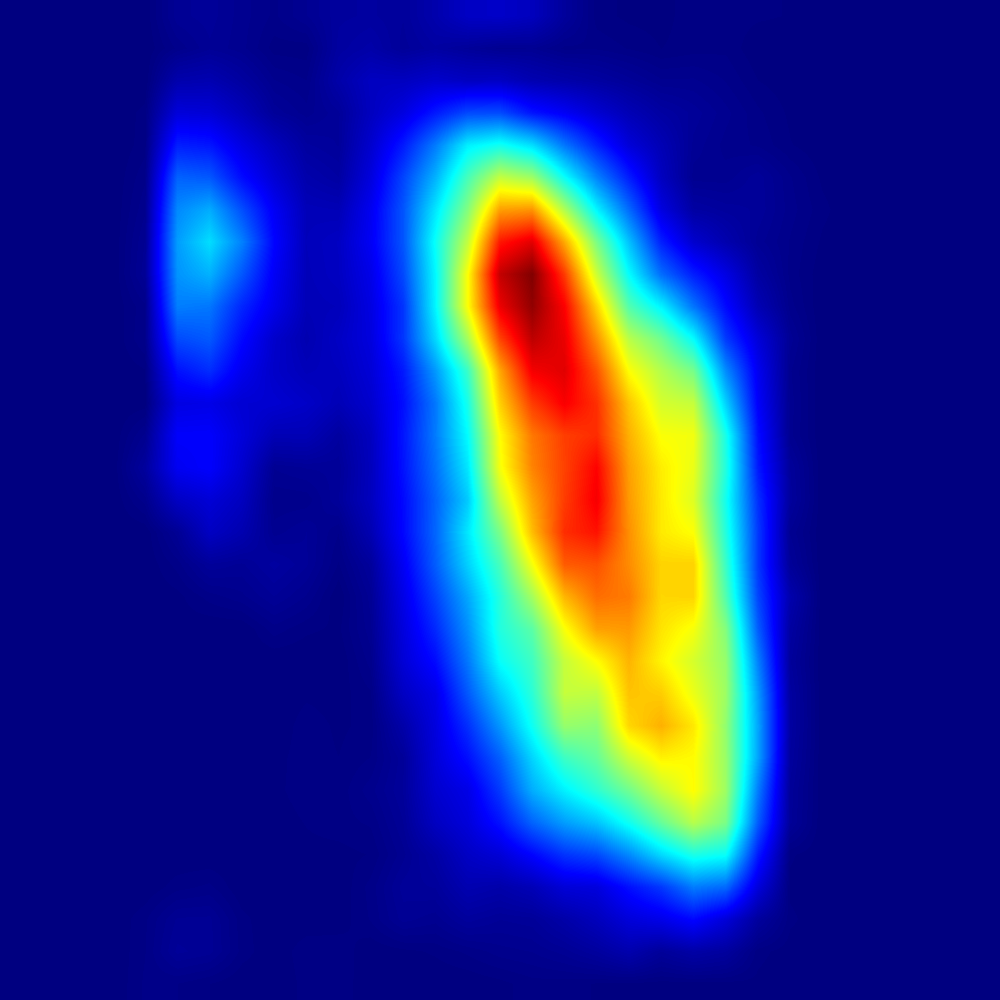

Supplement: Supplementary file 3 — Source Data File [file 41746_2022_681_MOESM3_ESM.zip › ARDA Map/Figure 5/5.png]

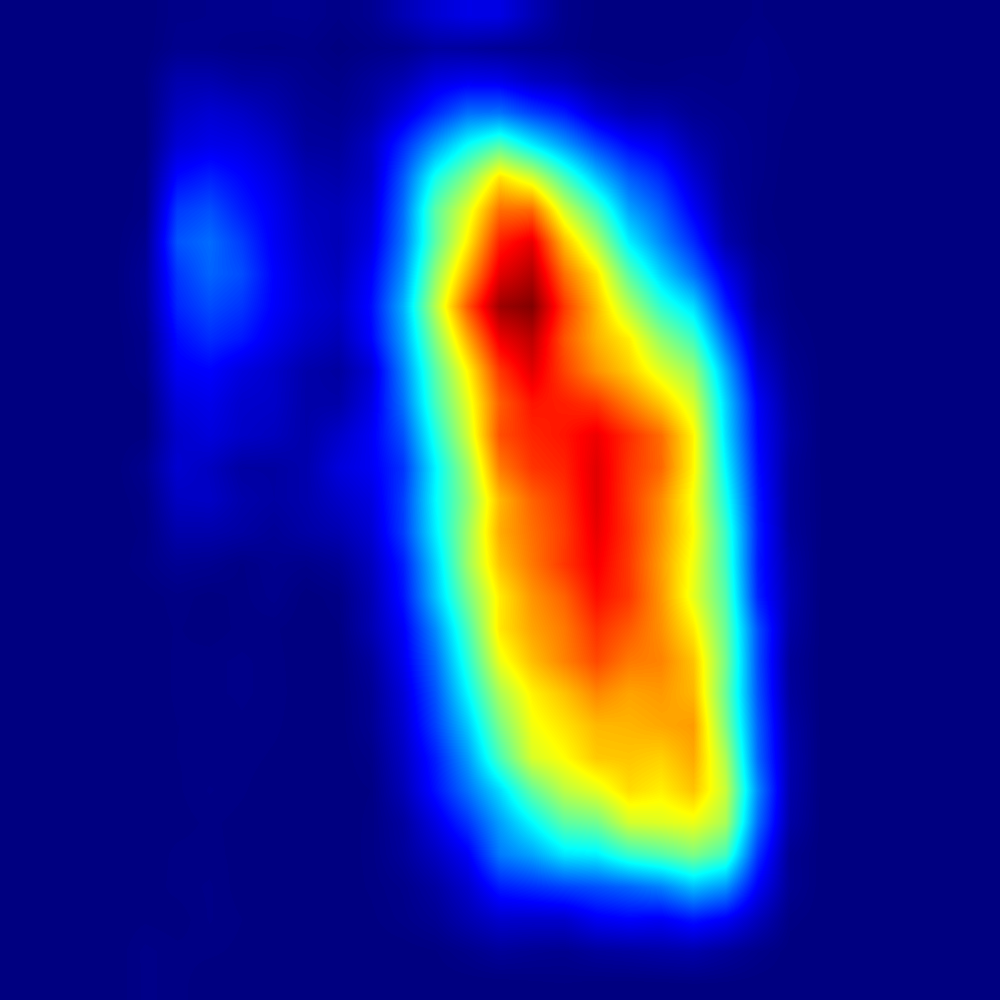

Supplement: Supplementary file 3 — Source Data File [file 41746_2022_681_MOESM3_ESM.zip › ARDA Map/Figure 5/6.png]

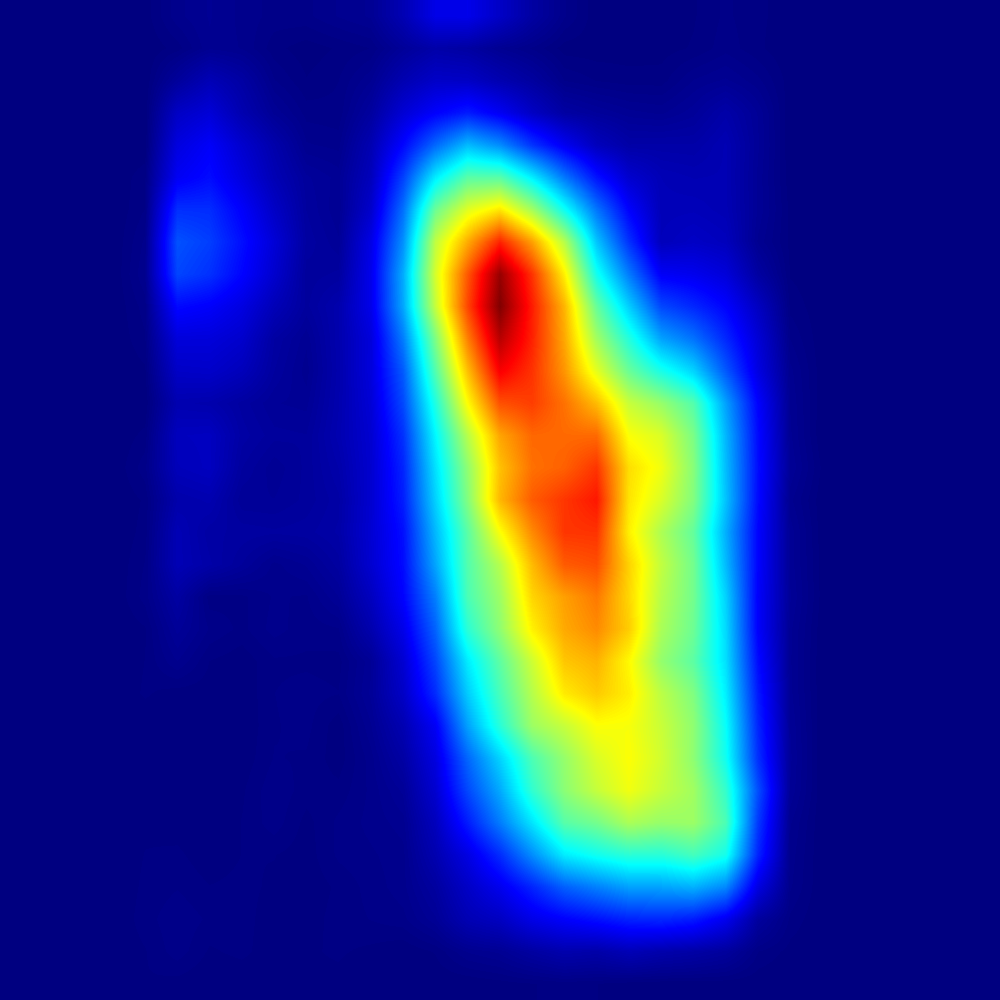

Supplement: Supplementary file 3 — Source Data File [file 41746_2022_681_MOESM3_ESM.zip › ARDA Map/Figure 5/7.png]

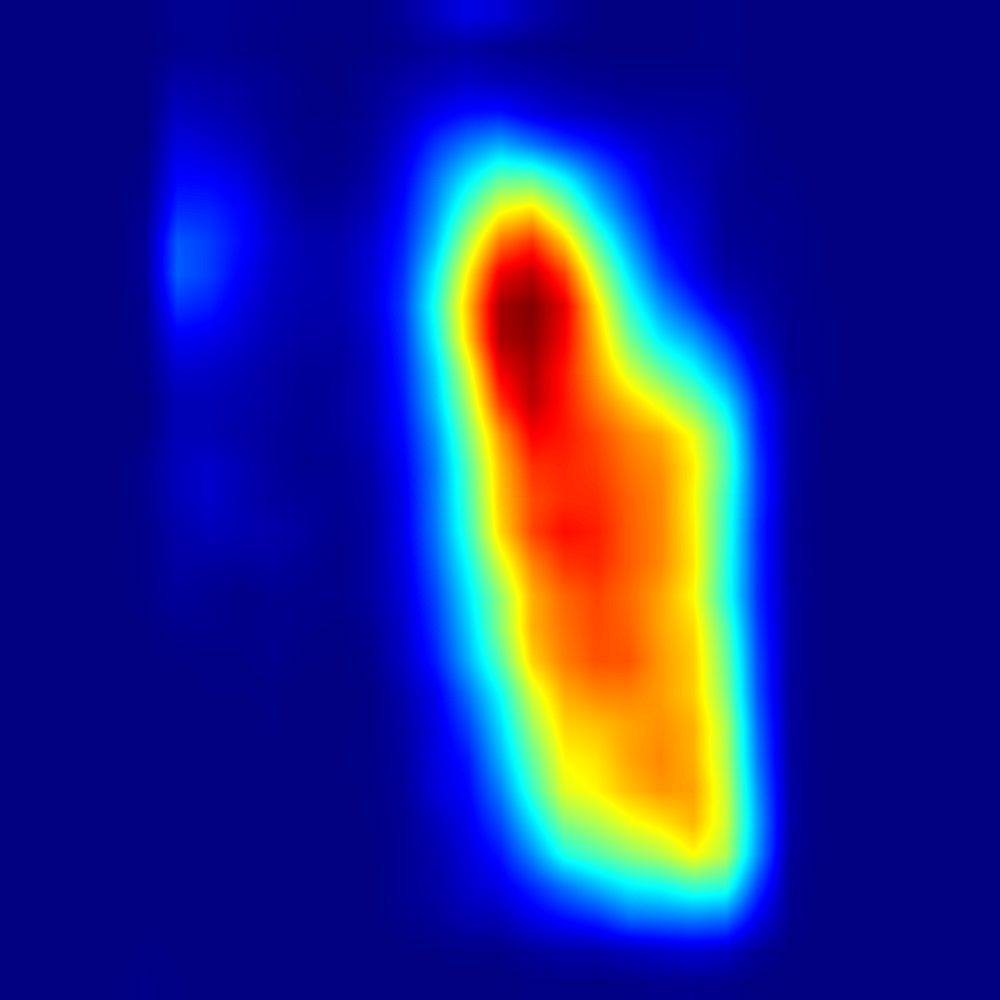

Supplement: Supplementary file 3 — Source Data File [file 41746_2022_681_MOESM3_ESM.zip › ARDA Map/Figure 5/8.png]

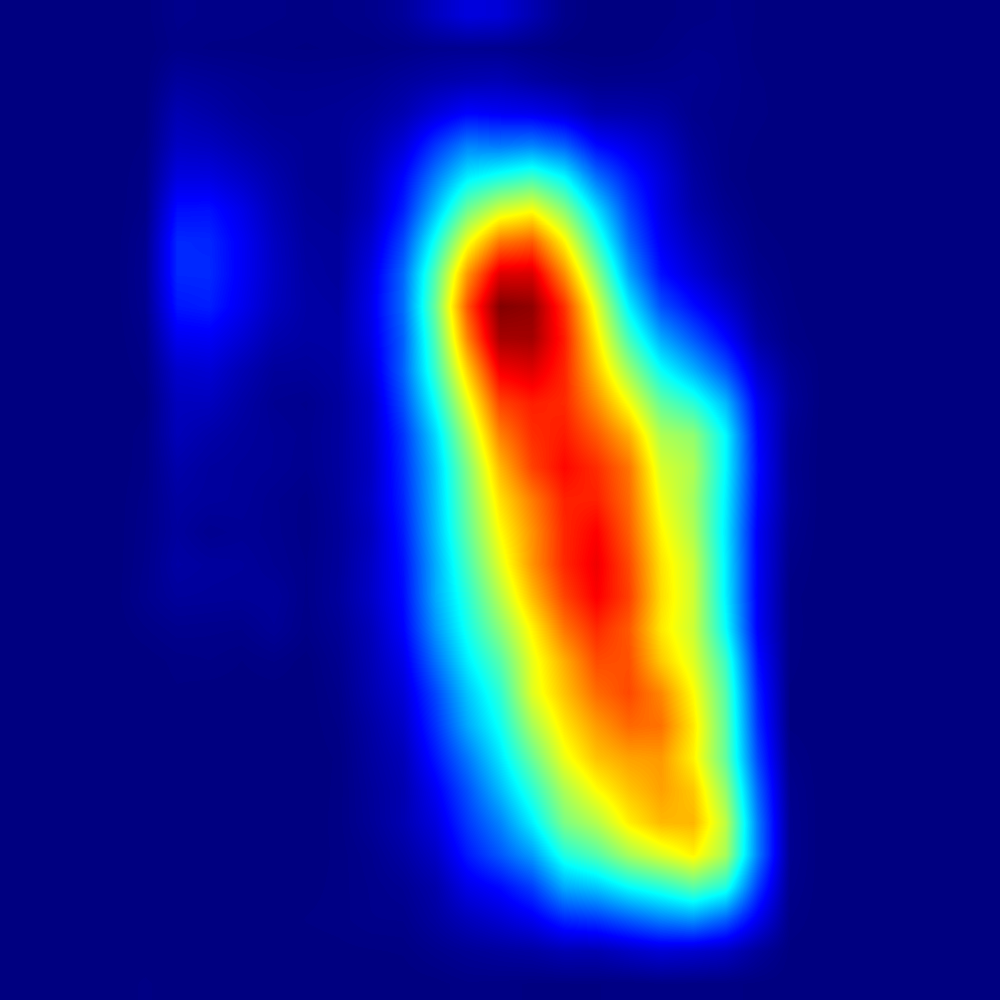

Supplement: Supplementary file 3 — Source Data File [file 41746_2022_681_MOESM3_ESM.zip › ARDA Map/Figure 5/9.png]
