# Supplementary figures and images for: Ageing and degeneration analysis using ageing-related dynamic attention on lateral cephalometric radiographs (part 1 of 2)
Source: NPJ Digit Med. 2022 Sep 27;5:151. doi: 10.1038/s41746-022-00681-y (PMC9515216; doi:10.1038/s41746-022-00681-y)

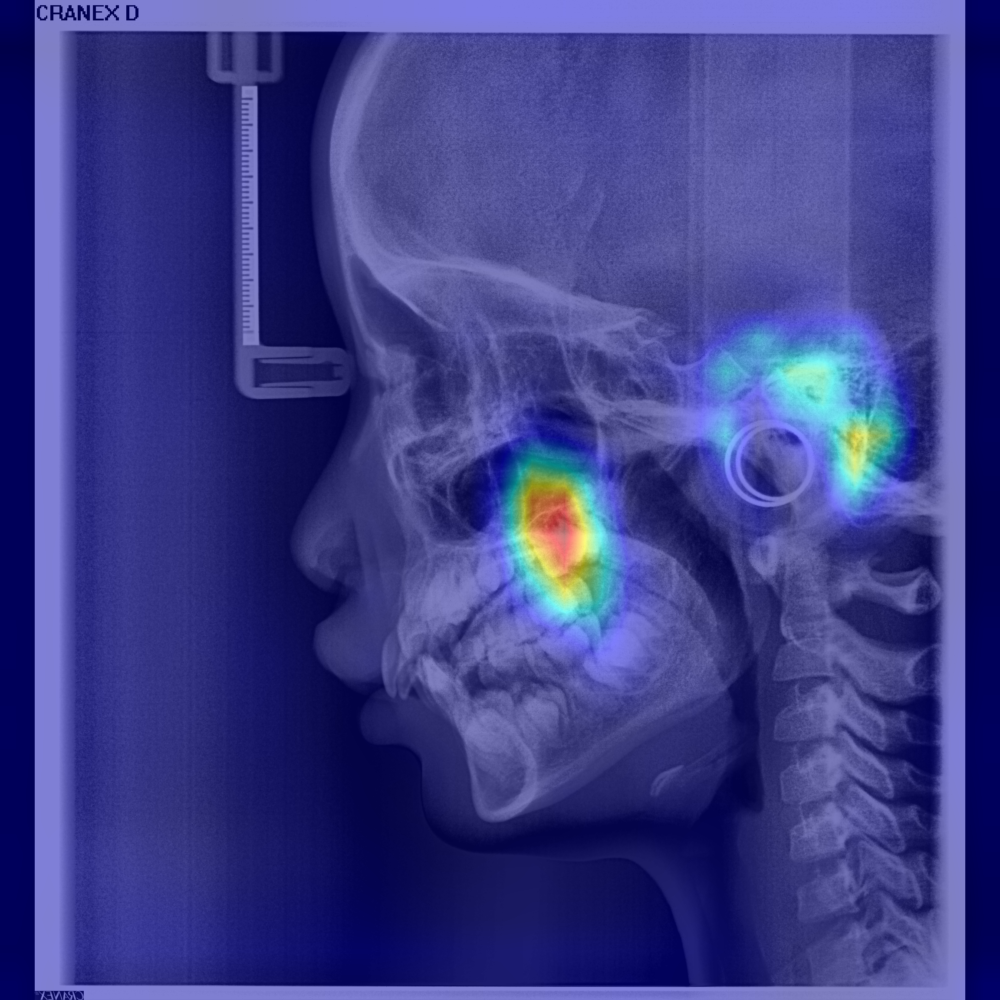

Supplement: Supplementary file 3 — Source Data File [file 41746_2022_681_MOESM3_ESM.zip › Ageing Saliency Map/10.png]

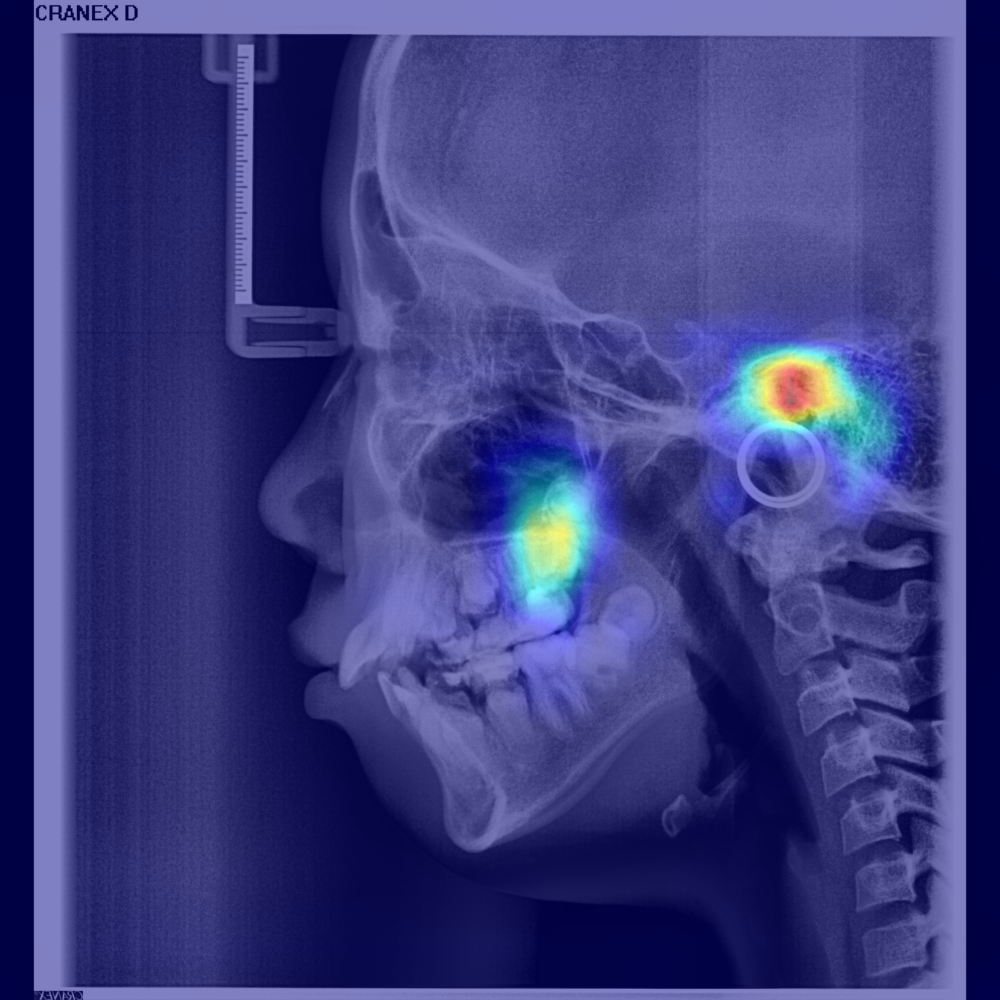

Supplement: Supplementary file 3 — Source Data File [file 41746_2022_681_MOESM3_ESM.zip › Ageing Saliency Map/11.png]

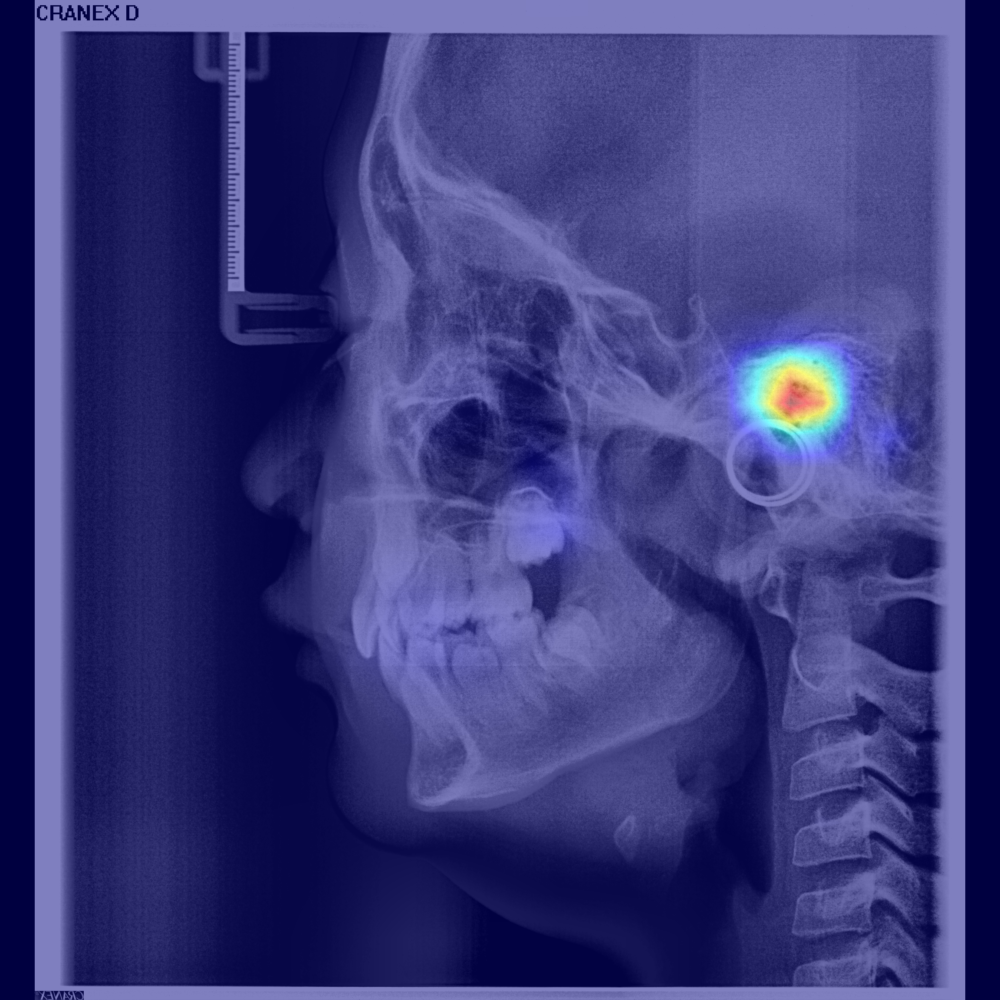

Supplement: Supplementary file 3 — Source Data File [file 41746_2022_681_MOESM3_ESM.zip › Ageing Saliency Map/12.png]

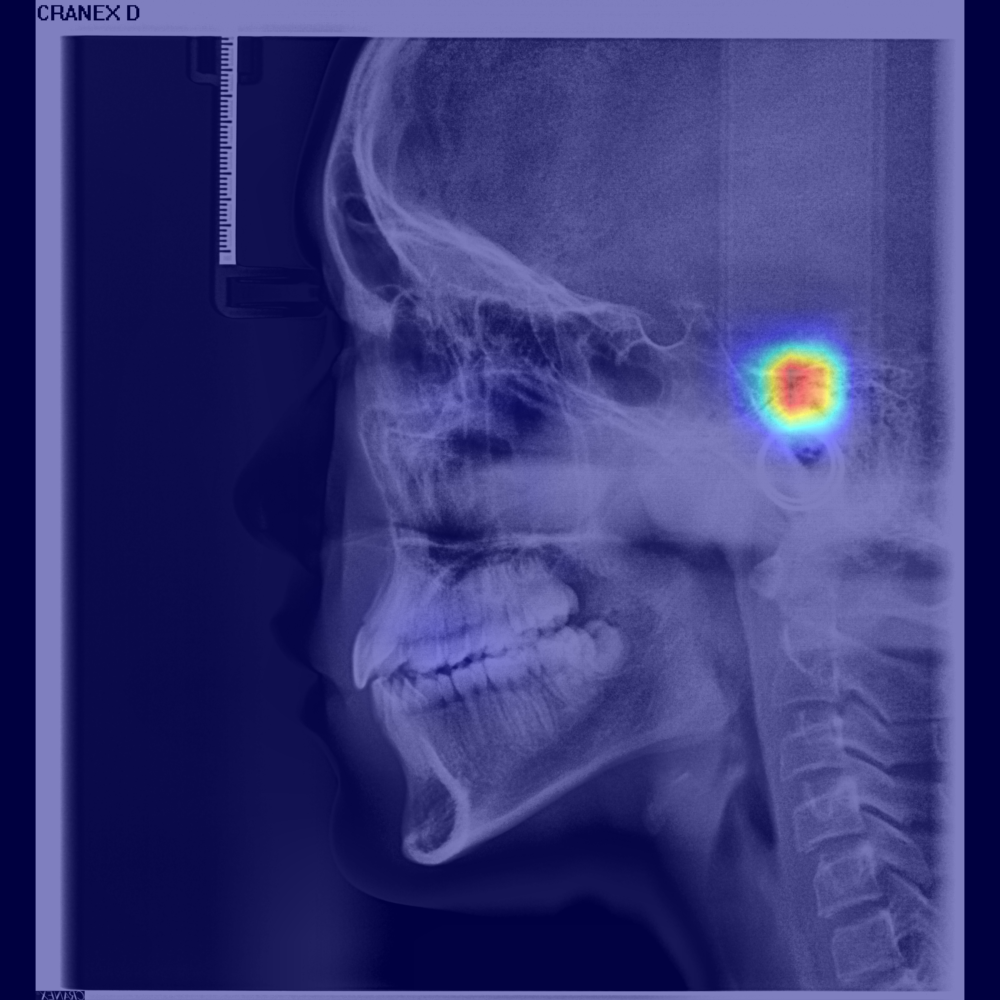

Supplement: Supplementary file 3 — Source Data File [file 41746_2022_681_MOESM3_ESM.zip › Ageing Saliency Map/13.png]

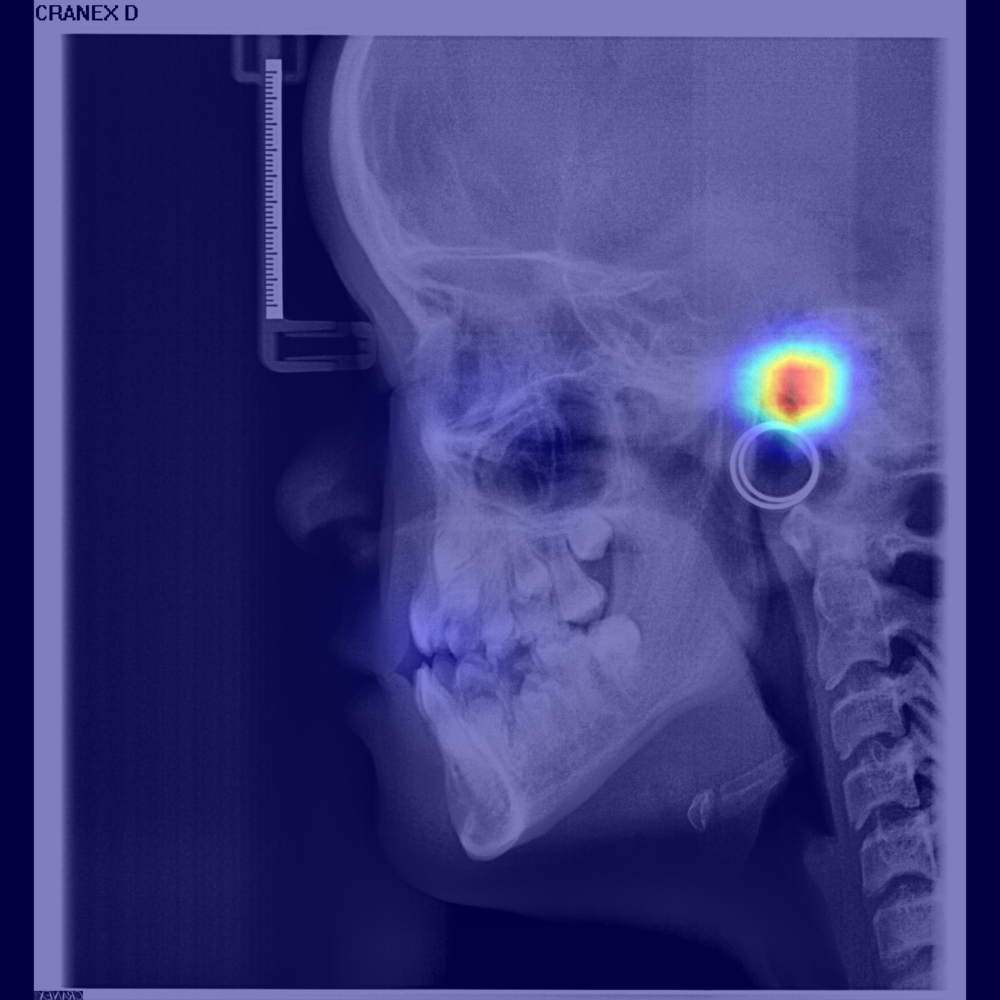

Supplement: Supplementary file 3 — Source Data File [file 41746_2022_681_MOESM3_ESM.zip › Ageing Saliency Map/14.png]

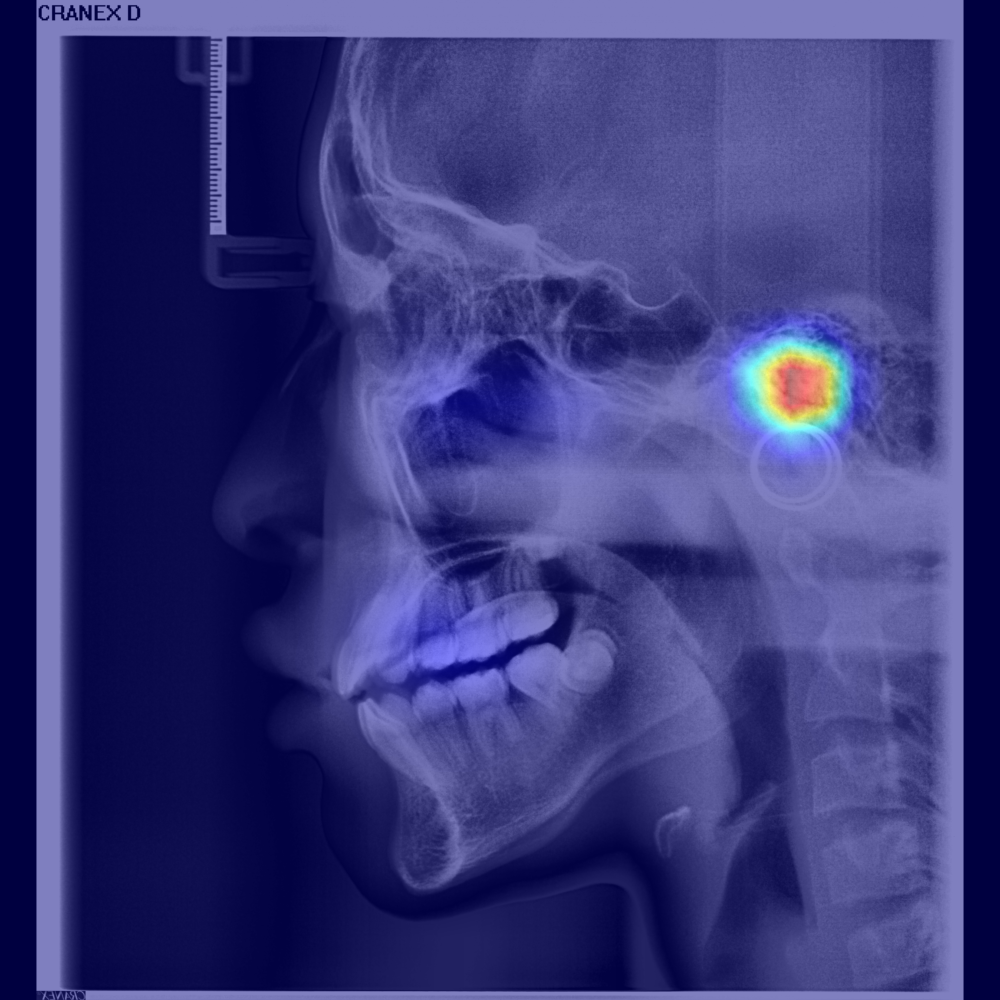

Supplement: Supplementary file 3 — Source Data File [file 41746_2022_681_MOESM3_ESM.zip › Ageing Saliency Map/15.png]

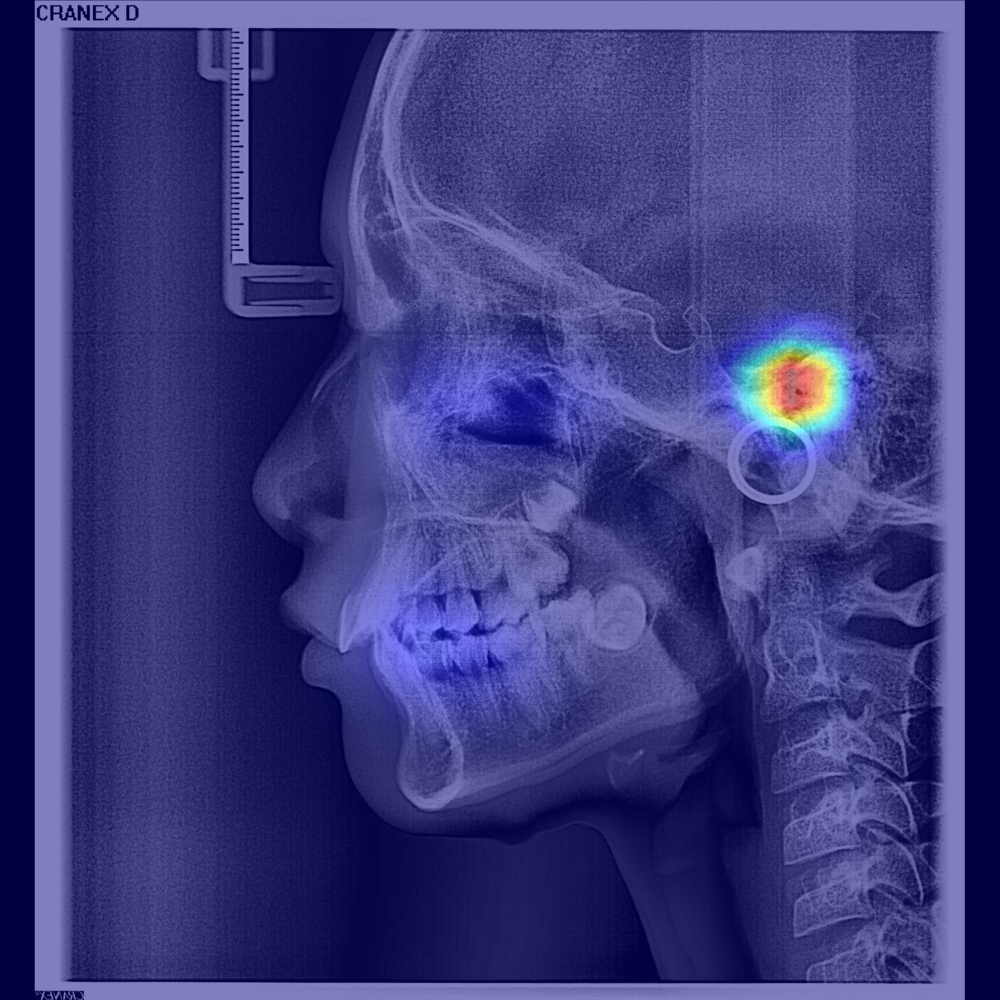

Supplement: Supplementary file 3 — Source Data File [file 41746_2022_681_MOESM3_ESM.zip › Ageing Saliency Map/16.png]

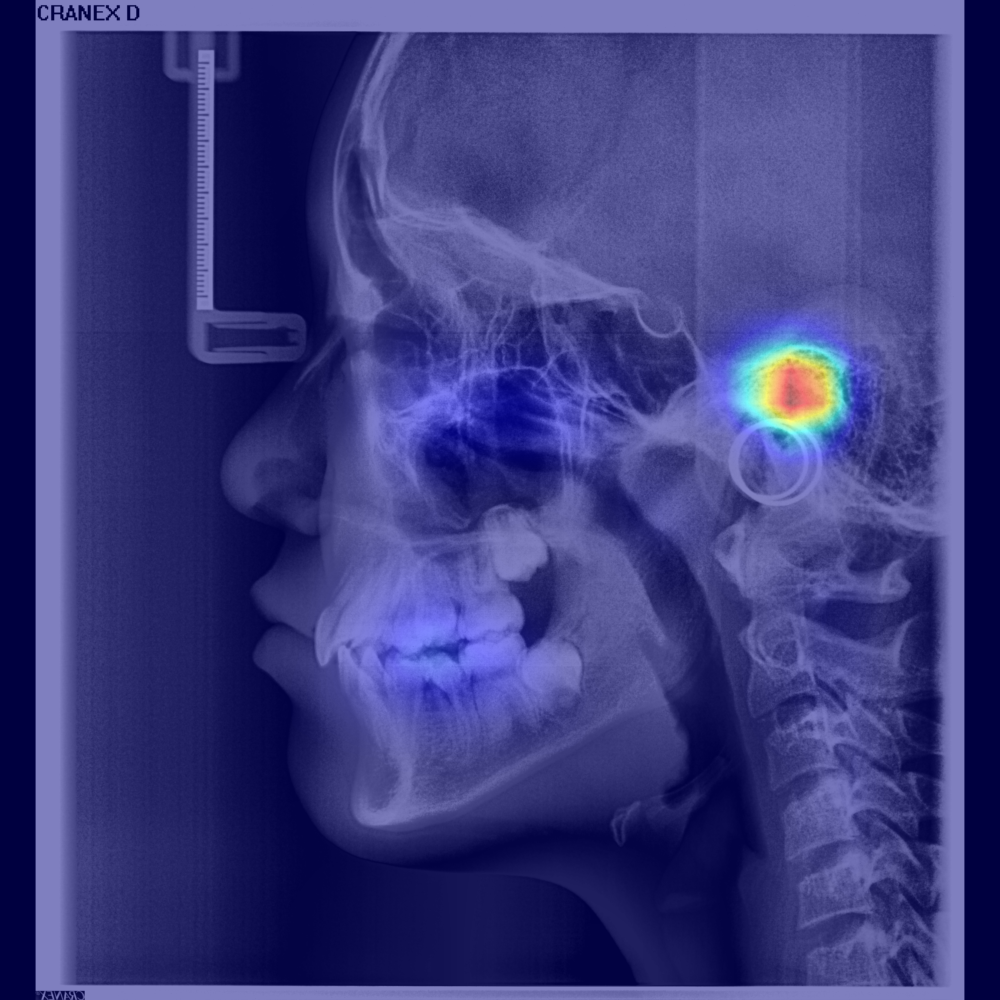

Supplement: Supplementary file 3 — Source Data File [file 41746_2022_681_MOESM3_ESM.zip › Ageing Saliency Map/17.png]

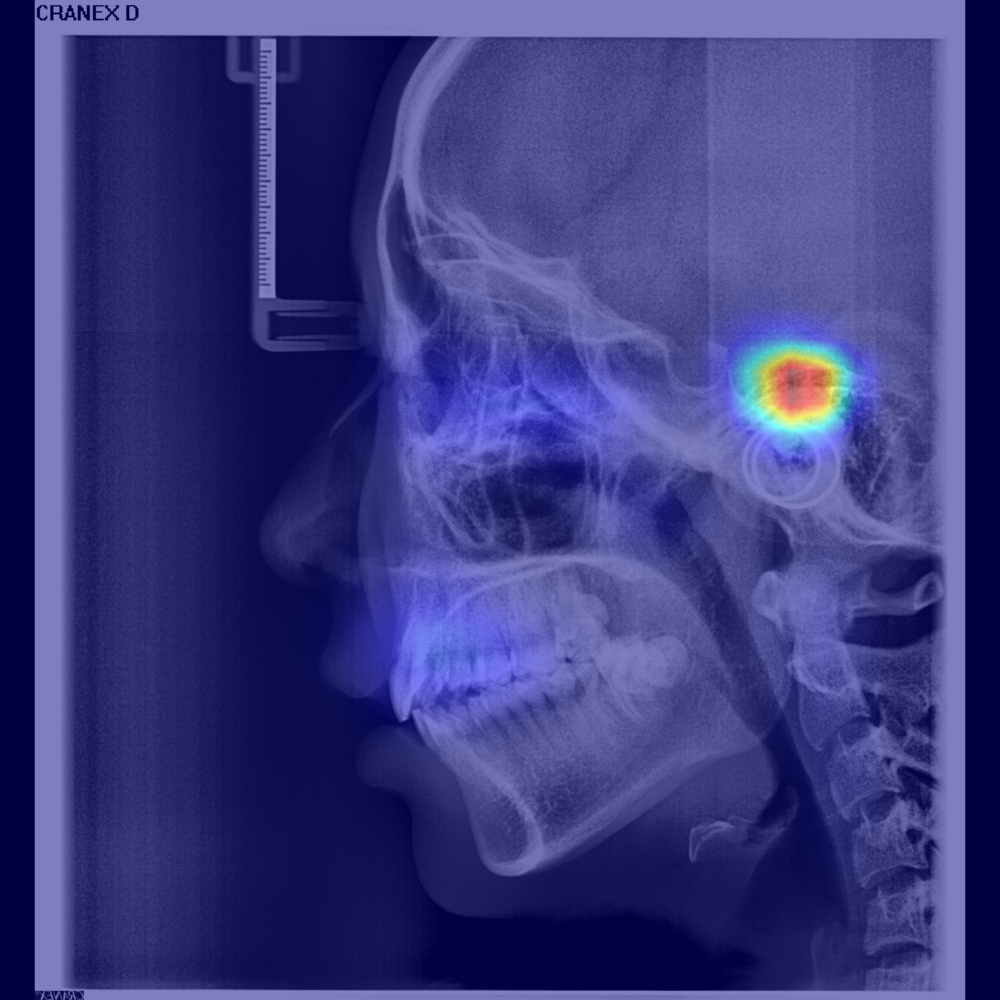

Supplement: Supplementary file 3 — Source Data File [file 41746_2022_681_MOESM3_ESM.zip › Ageing Saliency Map/18.png]

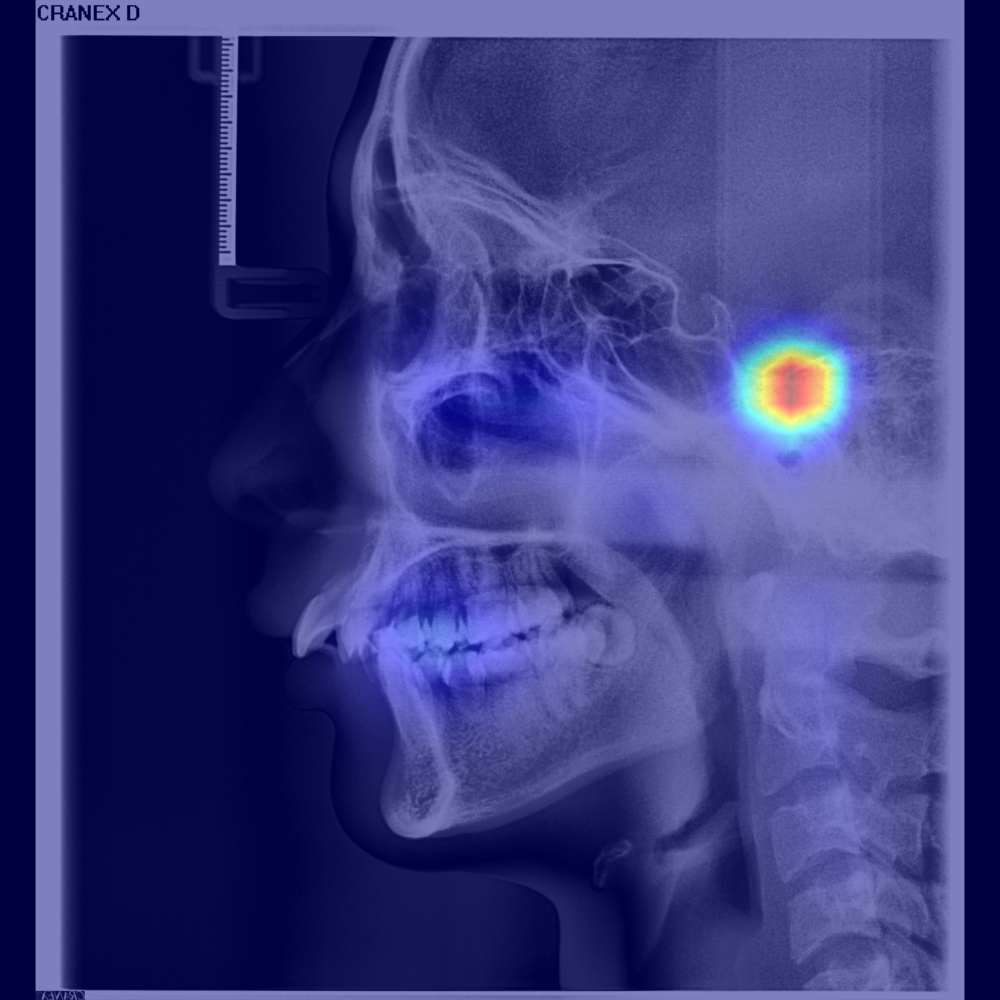

Supplement: Supplementary file 3 — Source Data File [file 41746_2022_681_MOESM3_ESM.zip › Ageing Saliency Map/19.png]

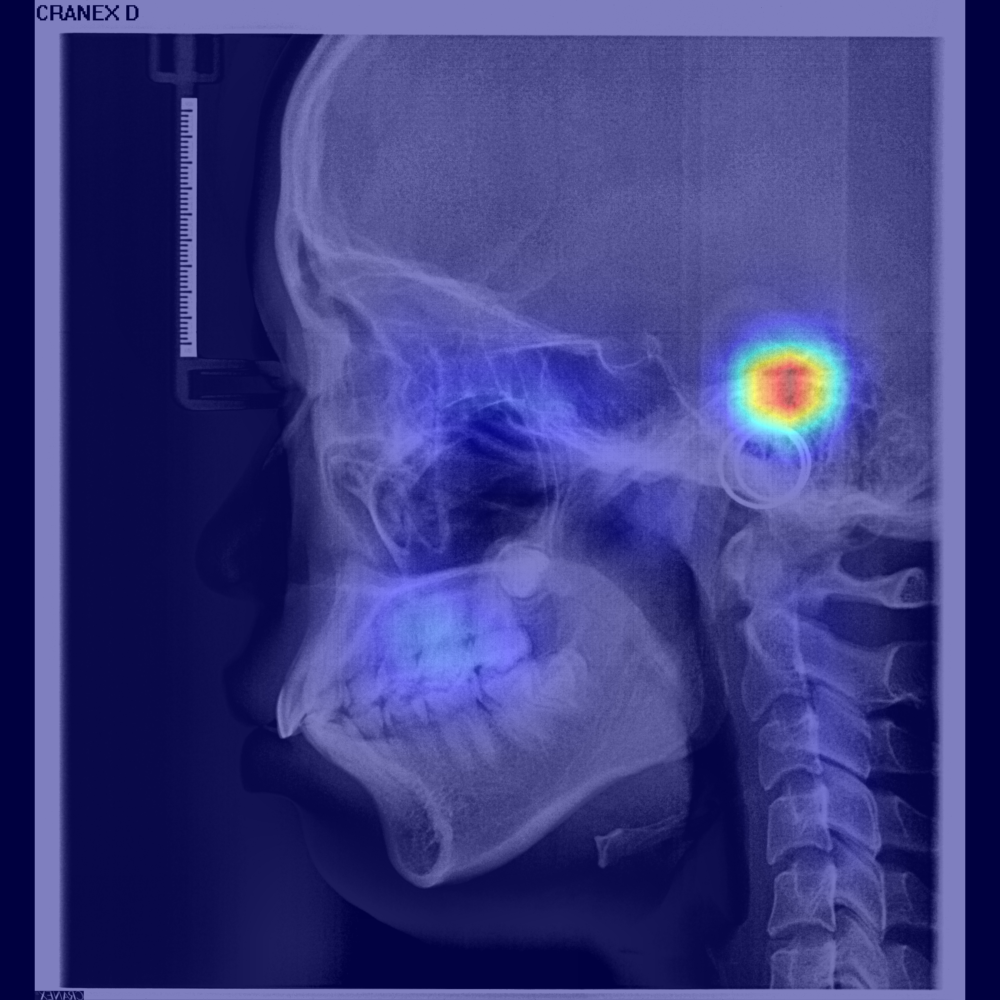

Supplement: Supplementary file 3 — Source Data File [file 41746_2022_681_MOESM3_ESM.zip › Ageing Saliency Map/20.png]

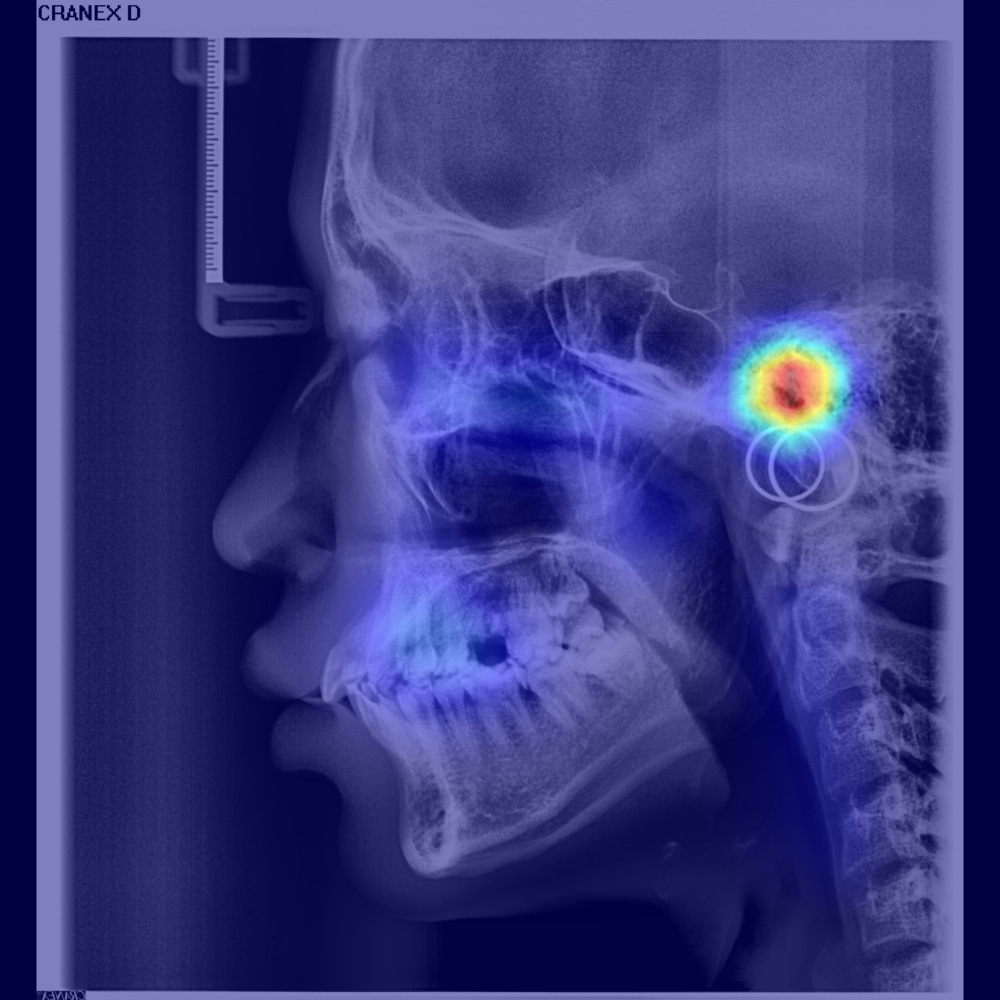

Supplement: Supplementary file 3 — Source Data File [file 41746_2022_681_MOESM3_ESM.zip › Ageing Saliency Map/22.png]

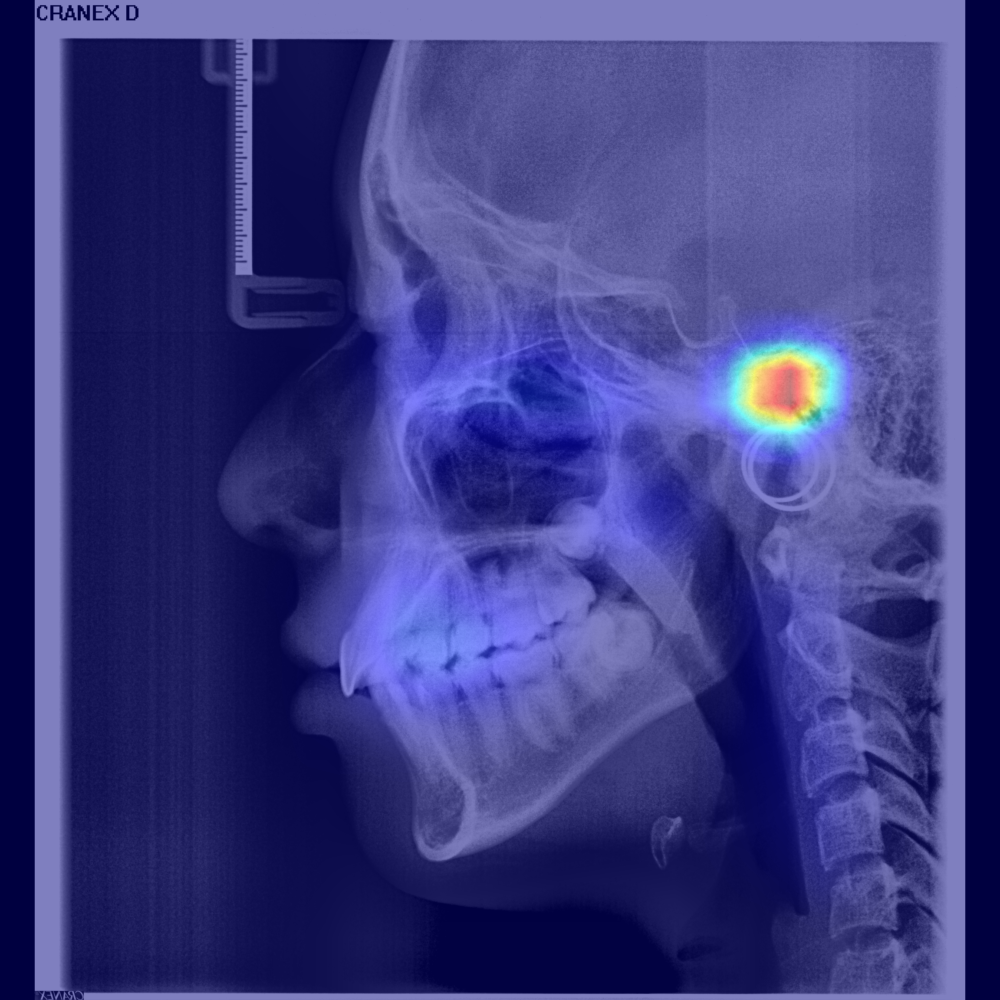

Supplement: Supplementary file 3 — Source Data File [file 41746_2022_681_MOESM3_ESM.zip › Ageing Saliency Map/23.png]

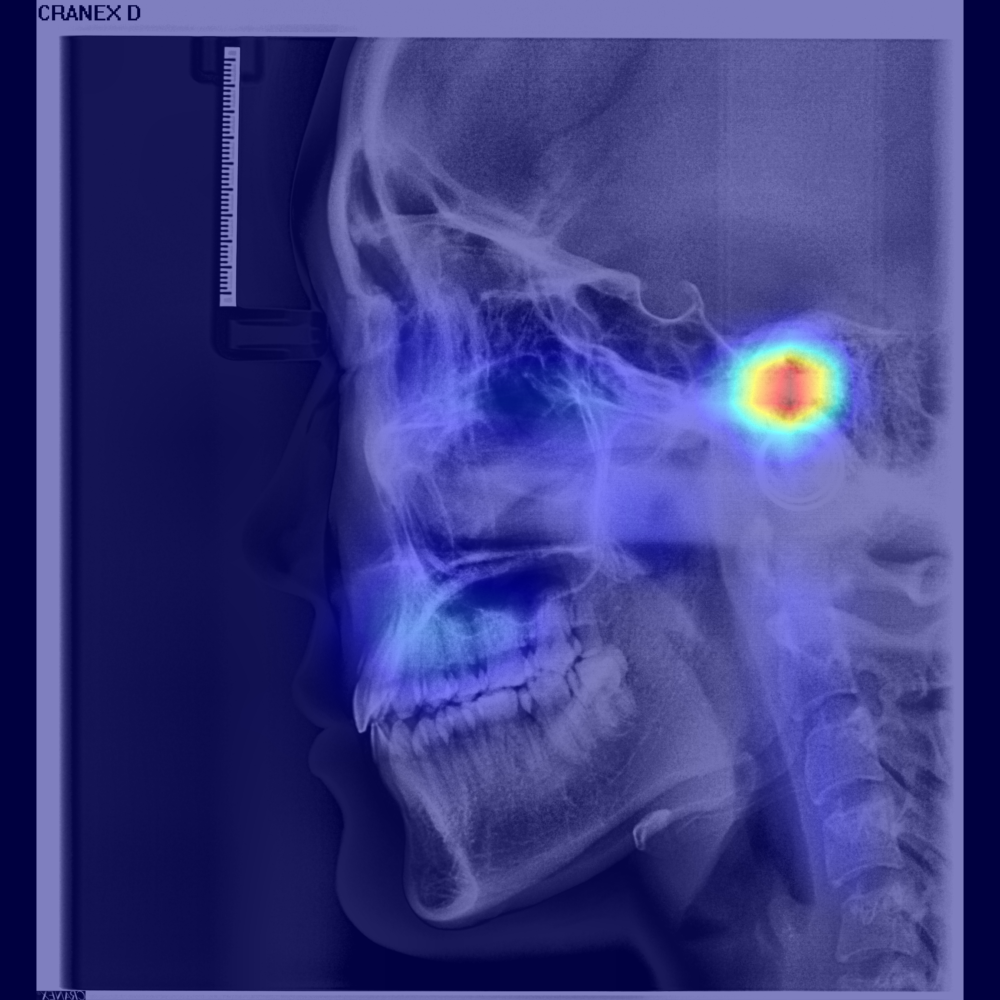

Supplement: Supplementary file 3 — Source Data File [file 41746_2022_681_MOESM3_ESM.zip › Ageing Saliency Map/24.png]

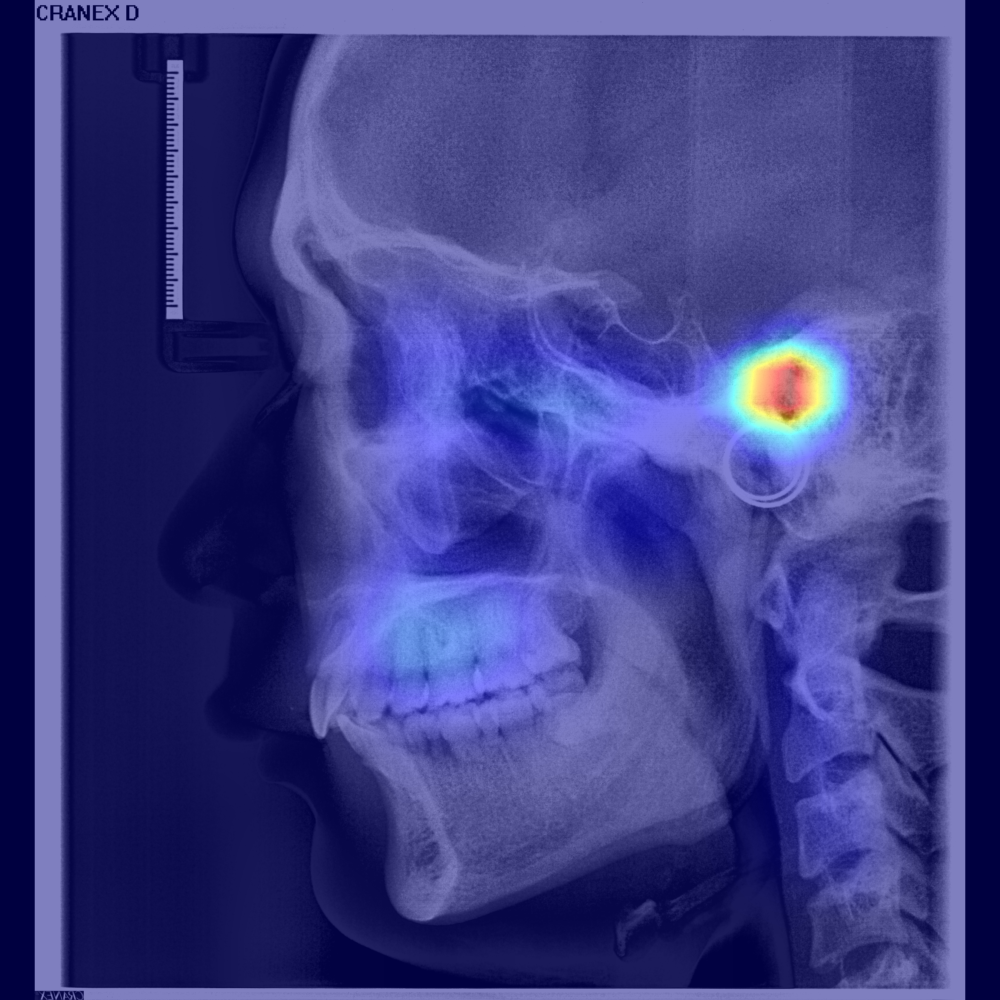

Supplement: Supplementary file 3 — Source Data File [file 41746_2022_681_MOESM3_ESM.zip › Ageing Saliency Map/25.png]

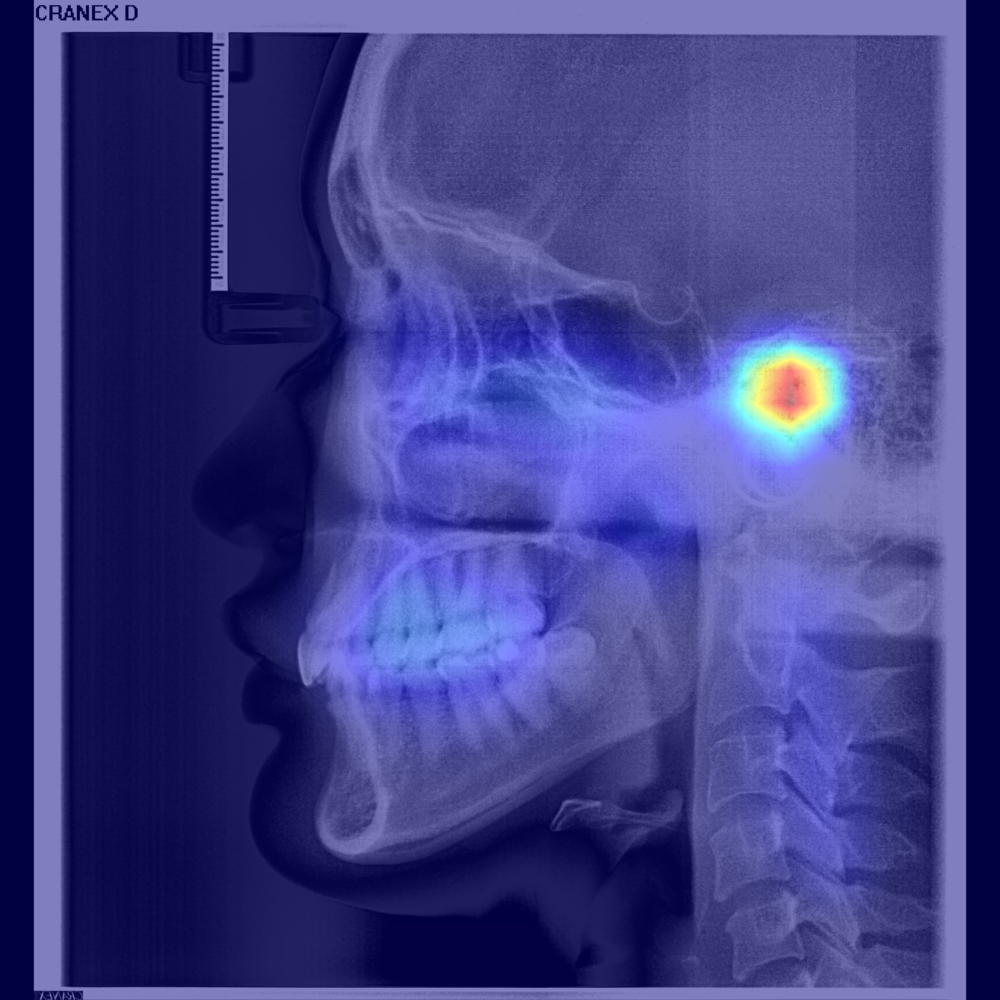

Supplement: Supplementary file 3 — Source Data File [file 41746_2022_681_MOESM3_ESM.zip › Ageing Saliency Map/26.png]

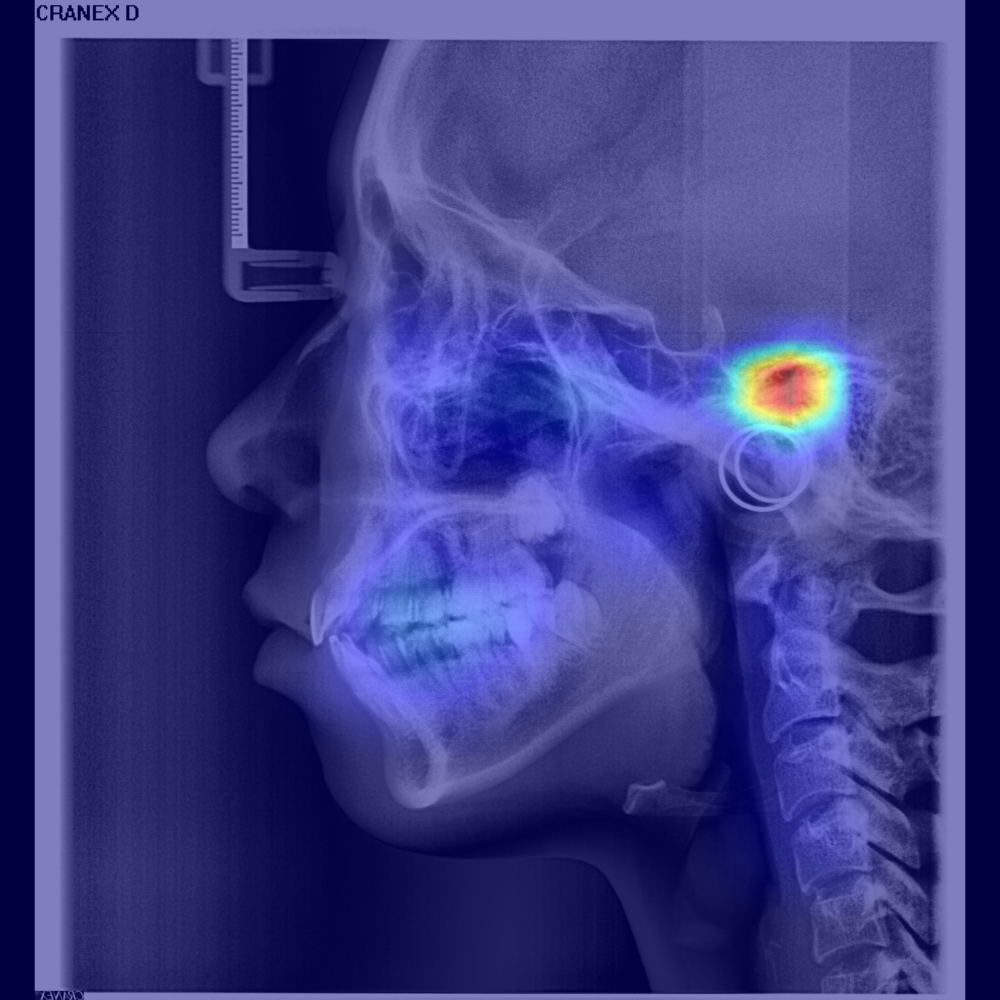

Supplement: Supplementary file 3 — Source Data File [file 41746_2022_681_MOESM3_ESM.zip › Ageing Saliency Map/27.png]

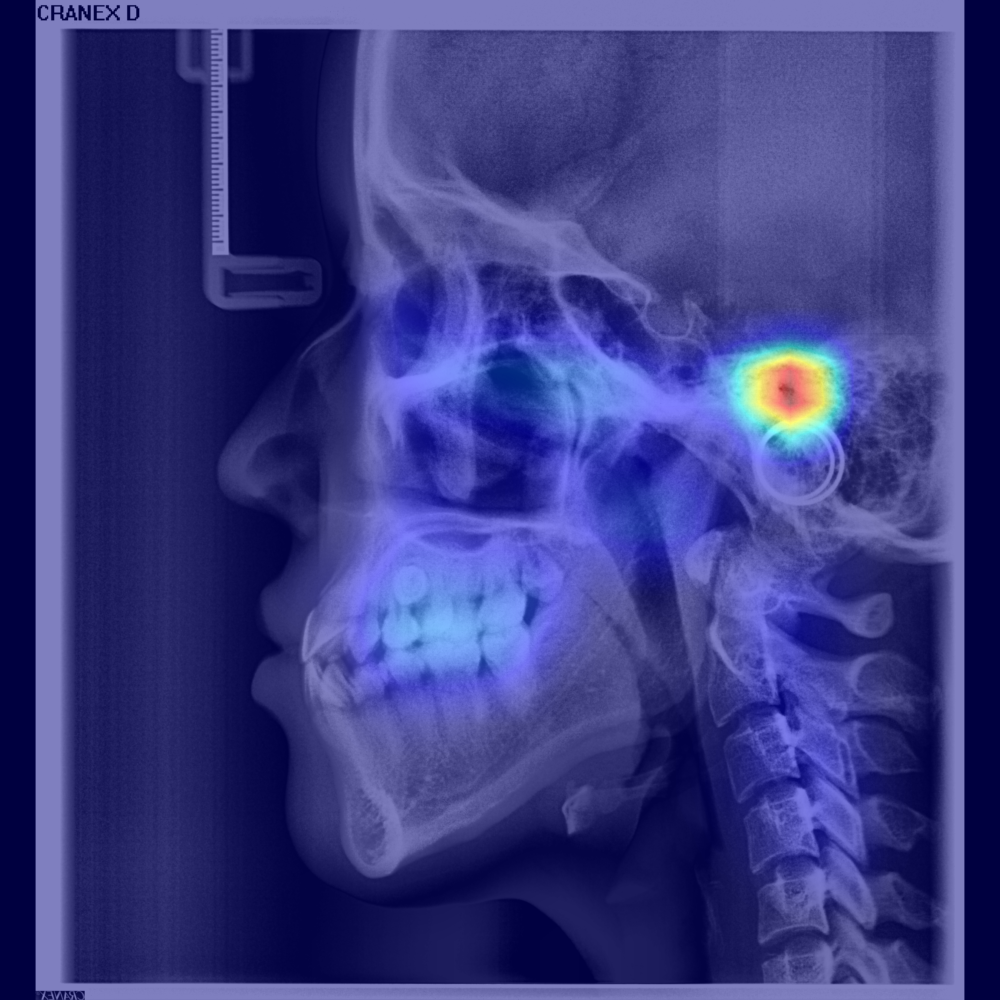

Supplement: Supplementary file 3 — Source Data File [file 41746_2022_681_MOESM3_ESM.zip › Ageing Saliency Map/28.png]

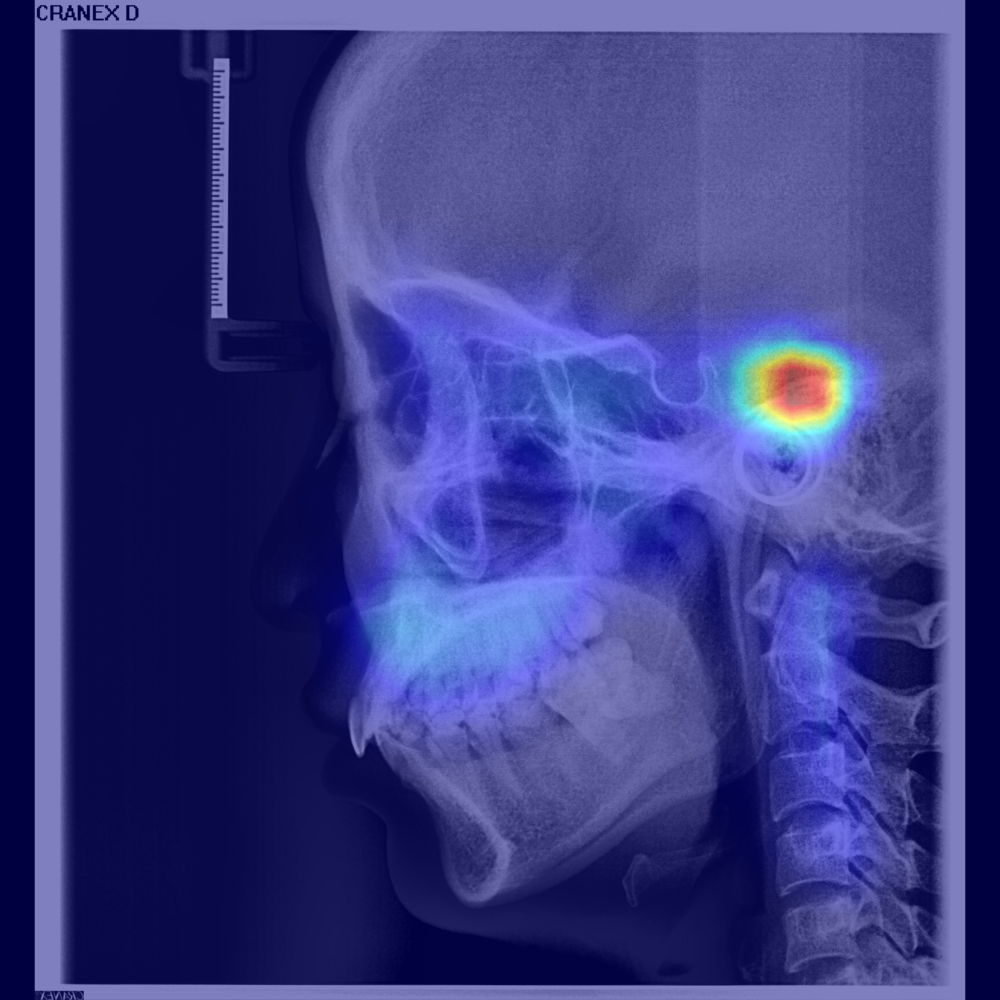

Supplement: Supplementary file 3 — Source Data File [file 41746_2022_681_MOESM3_ESM.zip › Ageing Saliency Map/29.png]

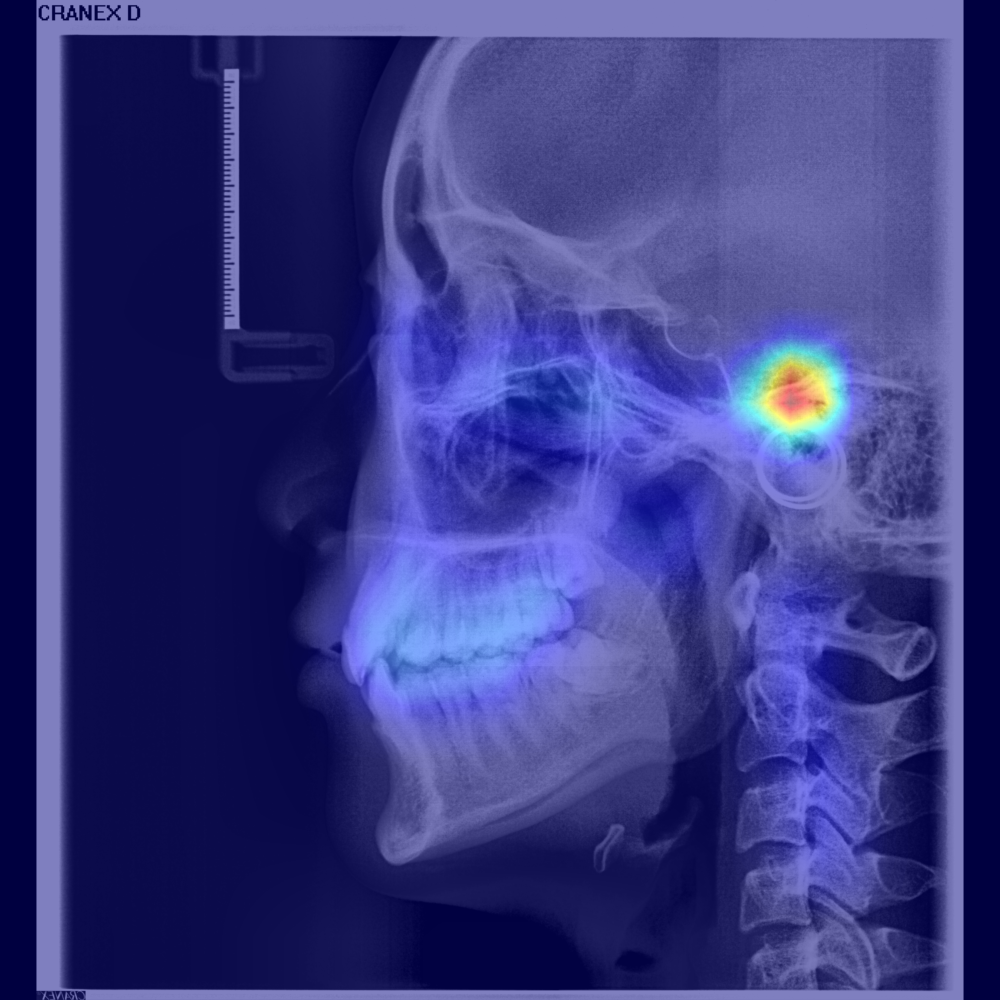

Supplement: Supplementary file 3 — Source Data File [file 41746_2022_681_MOESM3_ESM.zip › Ageing Saliency Map/30.png]

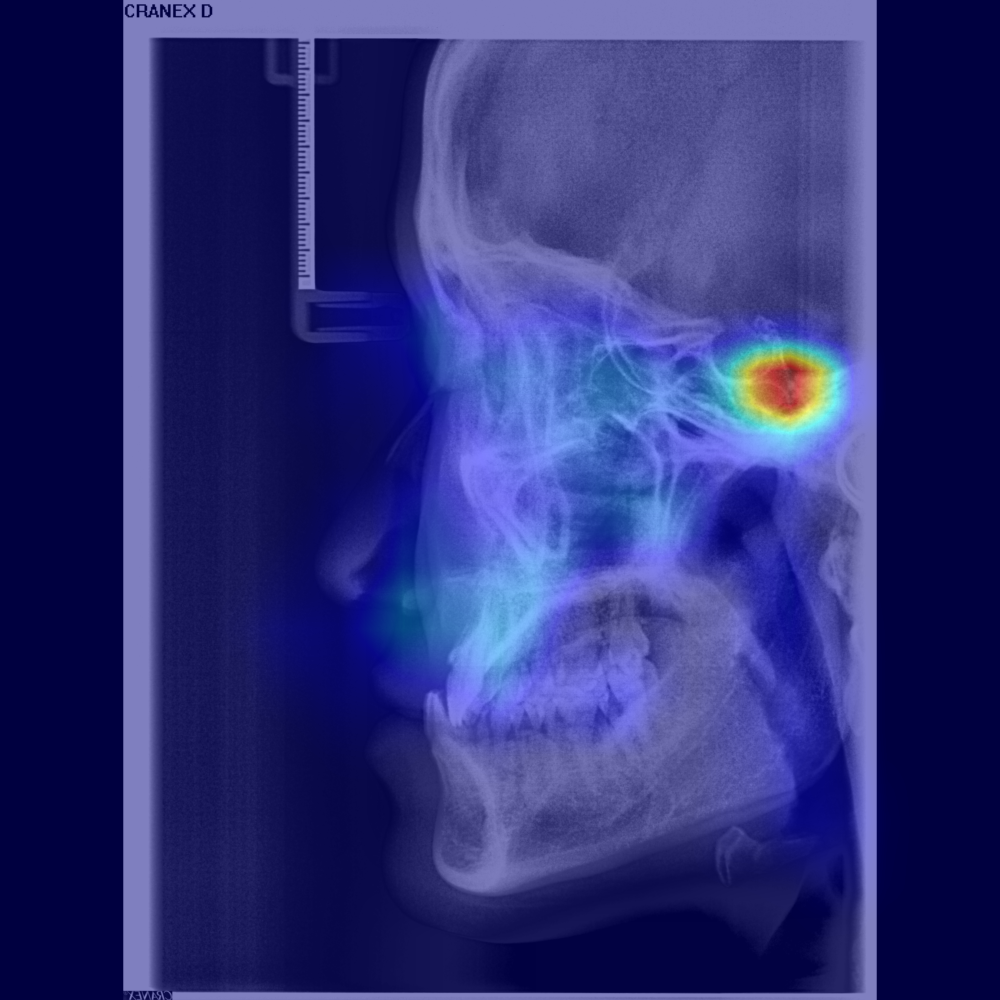

Supplement: Supplementary file 3 — Source Data File [file 41746_2022_681_MOESM3_ESM.zip › Ageing Saliency Map/31.png]

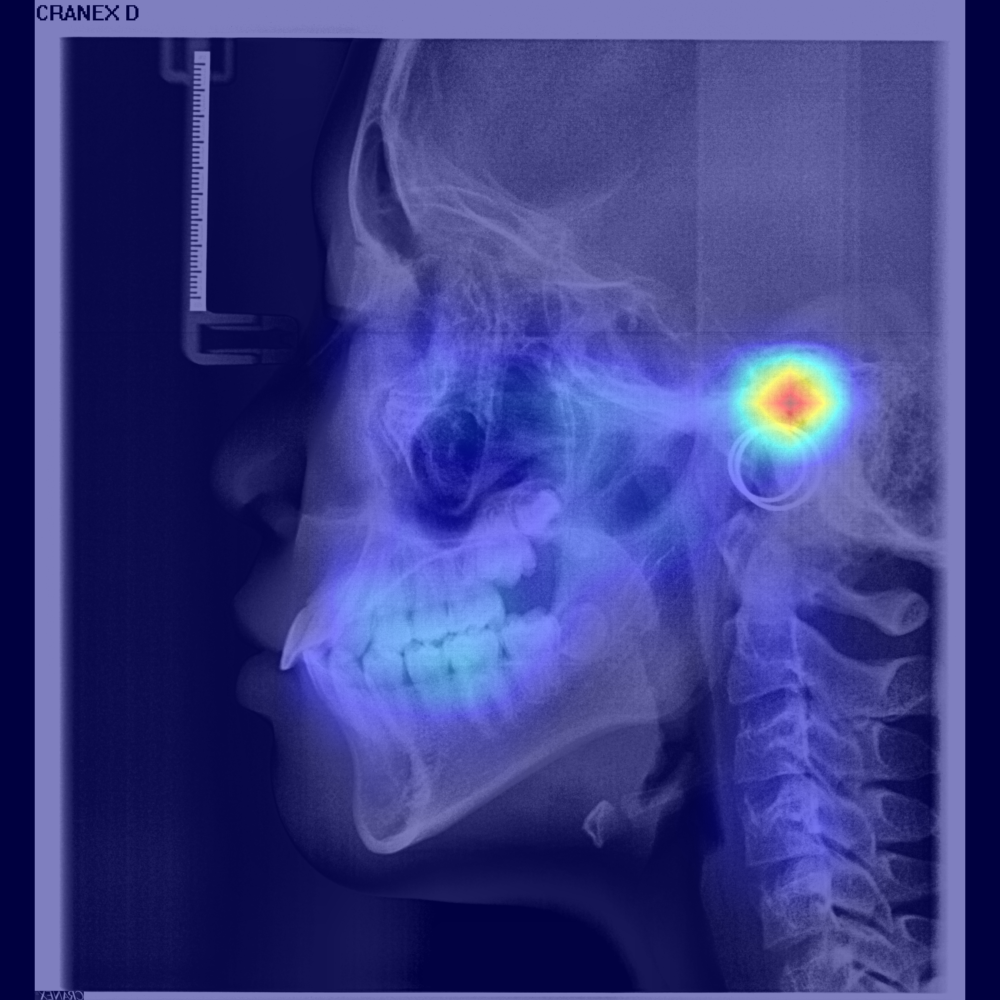

Supplement: Supplementary file 3 — Source Data File [file 41746_2022_681_MOESM3_ESM.zip › Ageing Saliency Map/32.png]

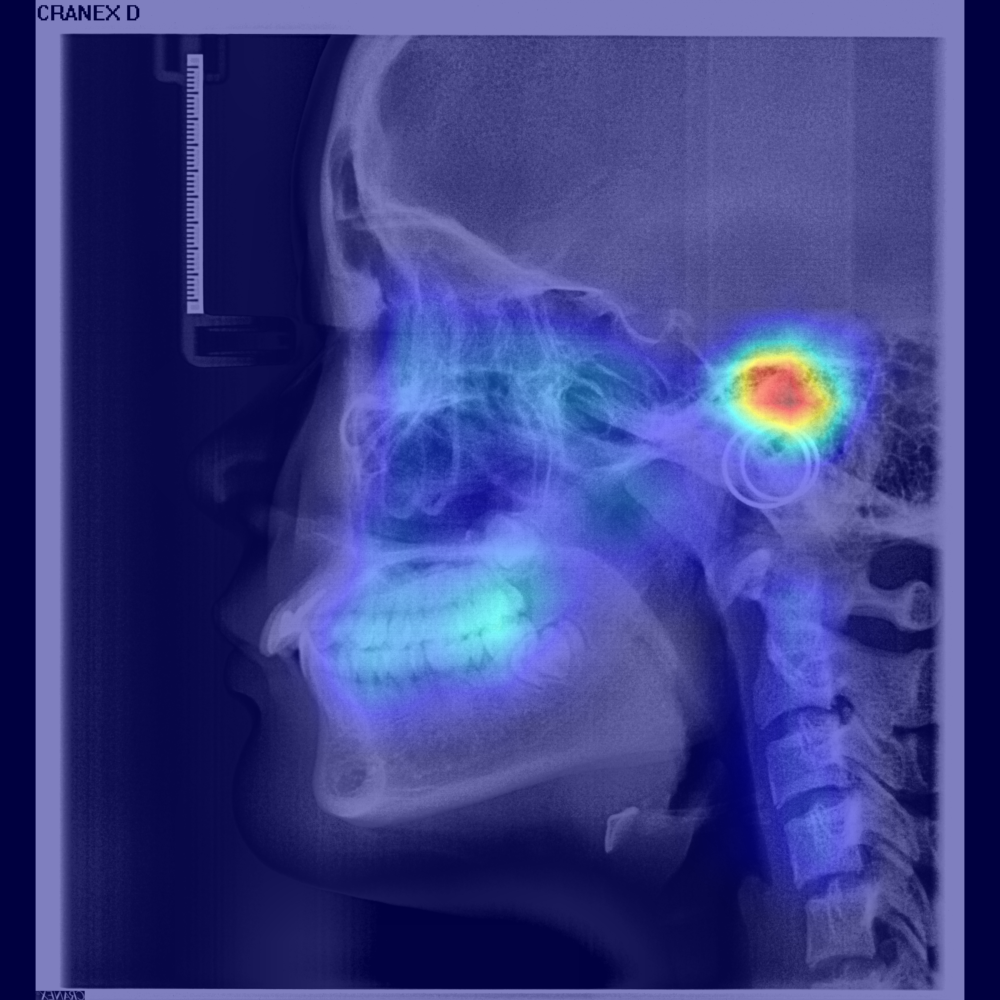

Supplement: Supplementary file 3 — Source Data File [file 41746_2022_681_MOESM3_ESM.zip › Ageing Saliency Map/33.png]

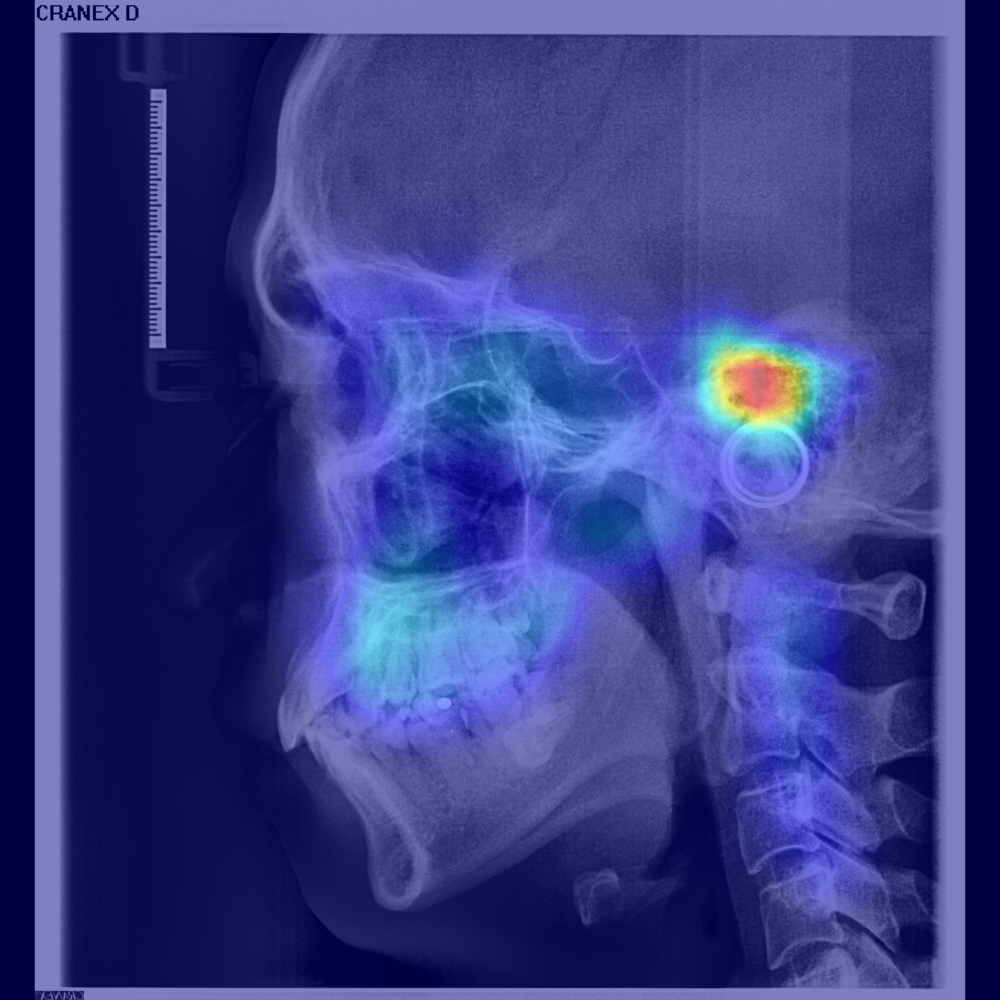

Supplement: Supplementary file 3 — Source Data File [file 41746_2022_681_MOESM3_ESM.zip › Ageing Saliency Map/34.png]

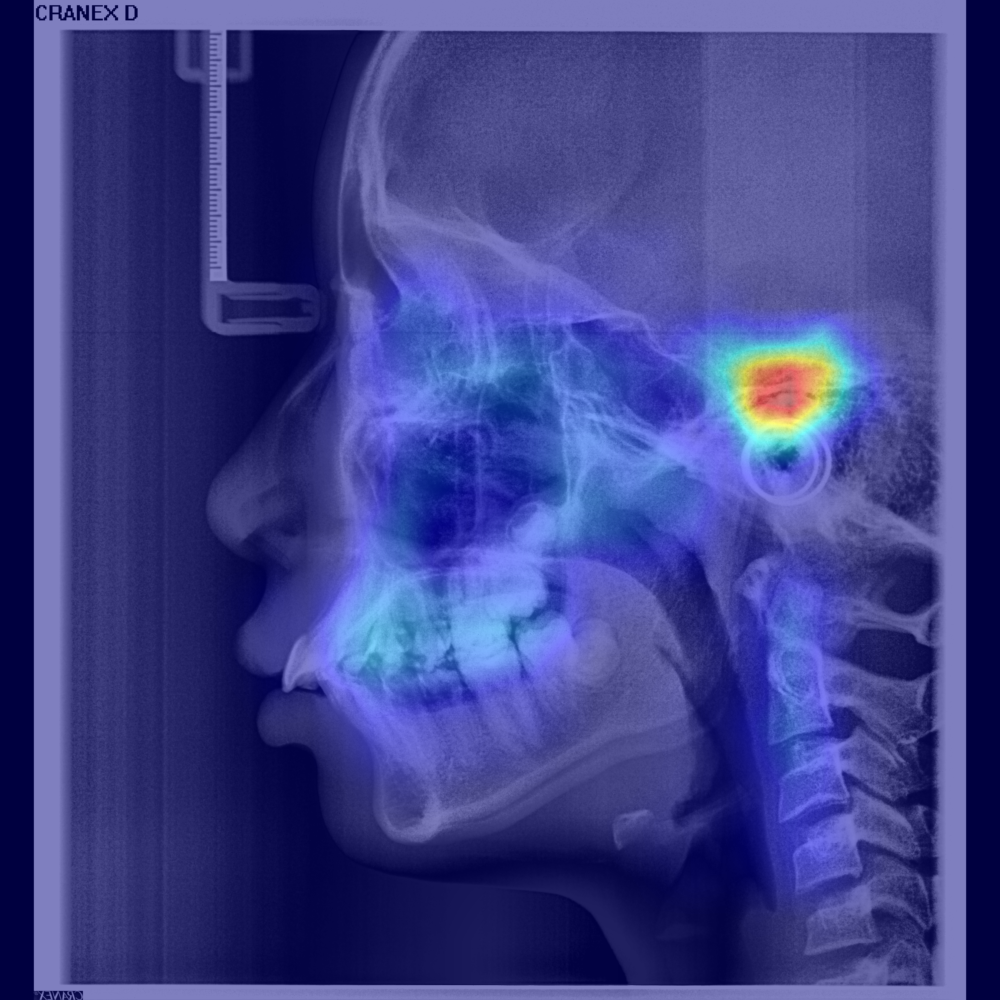

Supplement: Supplementary file 3 — Source Data File [file 41746_2022_681_MOESM3_ESM.zip › Ageing Saliency Map/36.png]

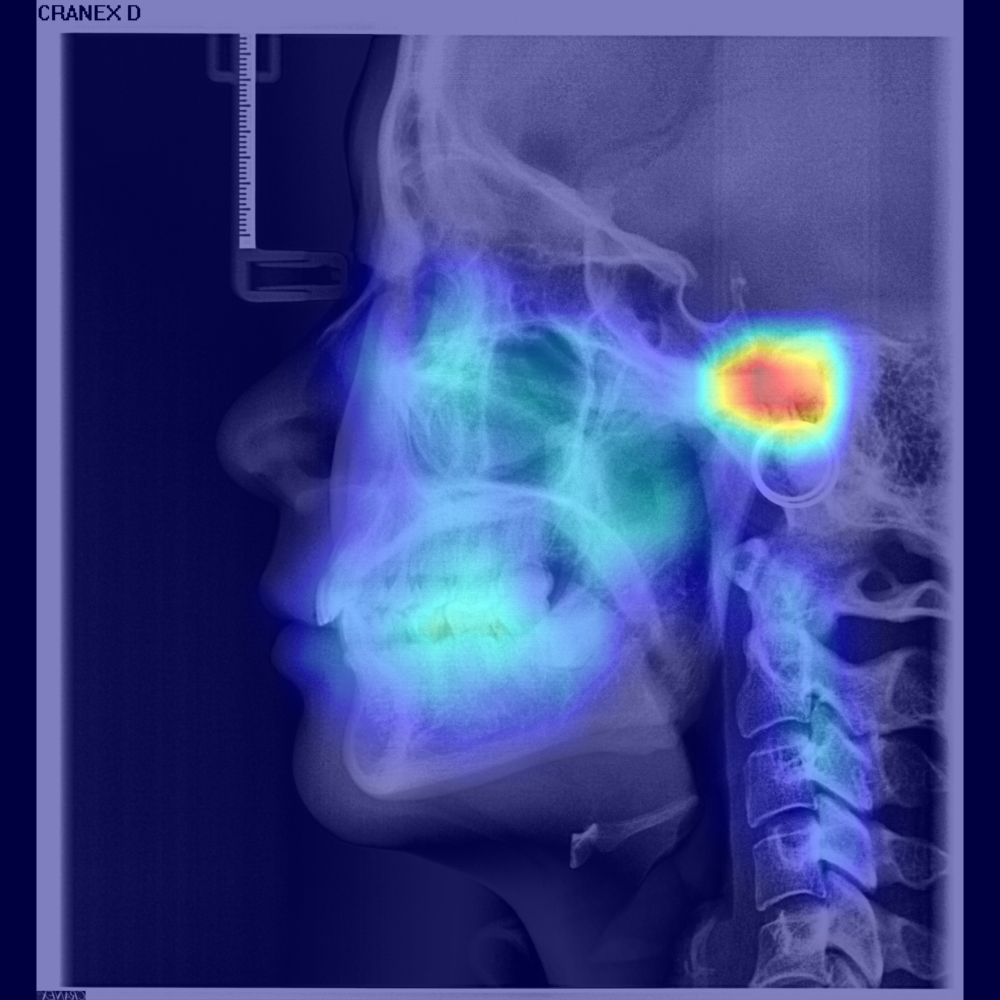

Supplement: Supplementary file 3 — Source Data File [file 41746_2022_681_MOESM3_ESM.zip › Ageing Saliency Map/37.png]

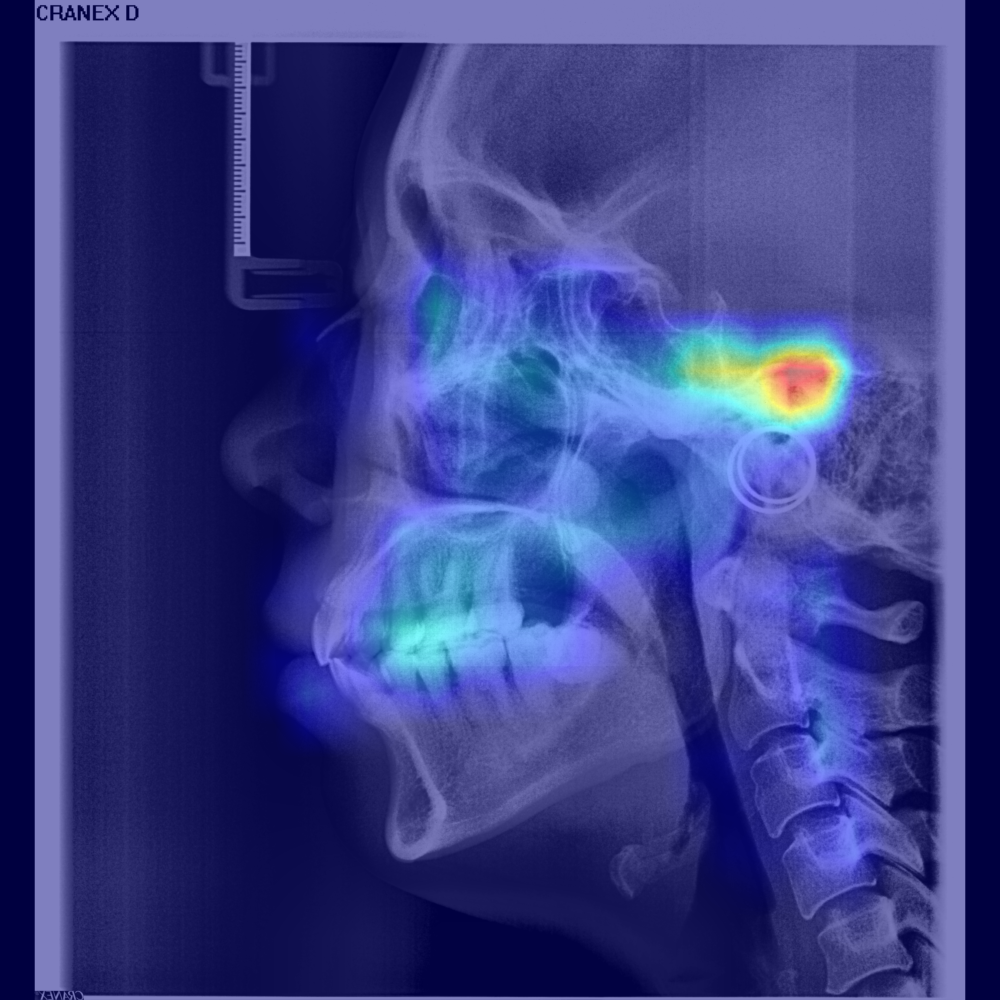

Supplement: Supplementary file 3 — Source Data File [file 41746_2022_681_MOESM3_ESM.zip › Ageing Saliency Map/38.png]

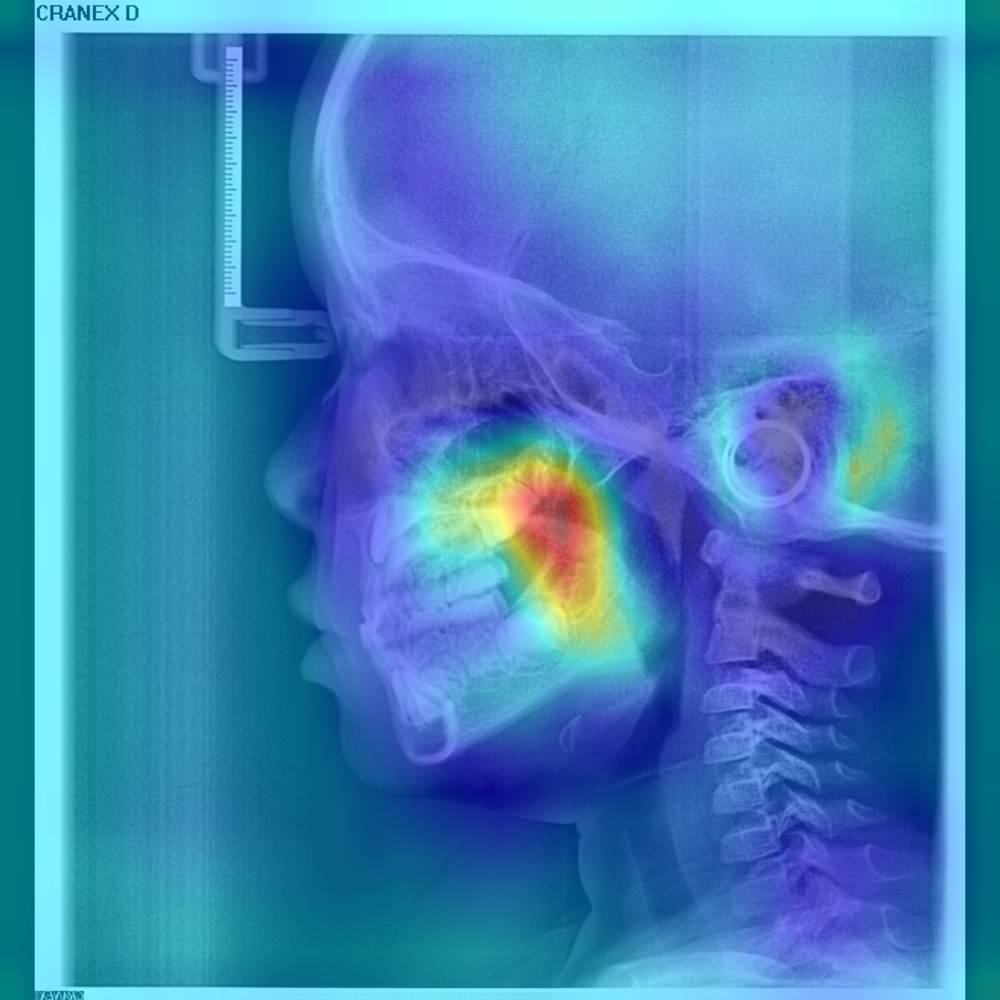

Supplement: Supplementary file 3 — Source Data File [file 41746_2022_681_MOESM3_ESM.zip › Ageing Saliency Map/4.png]

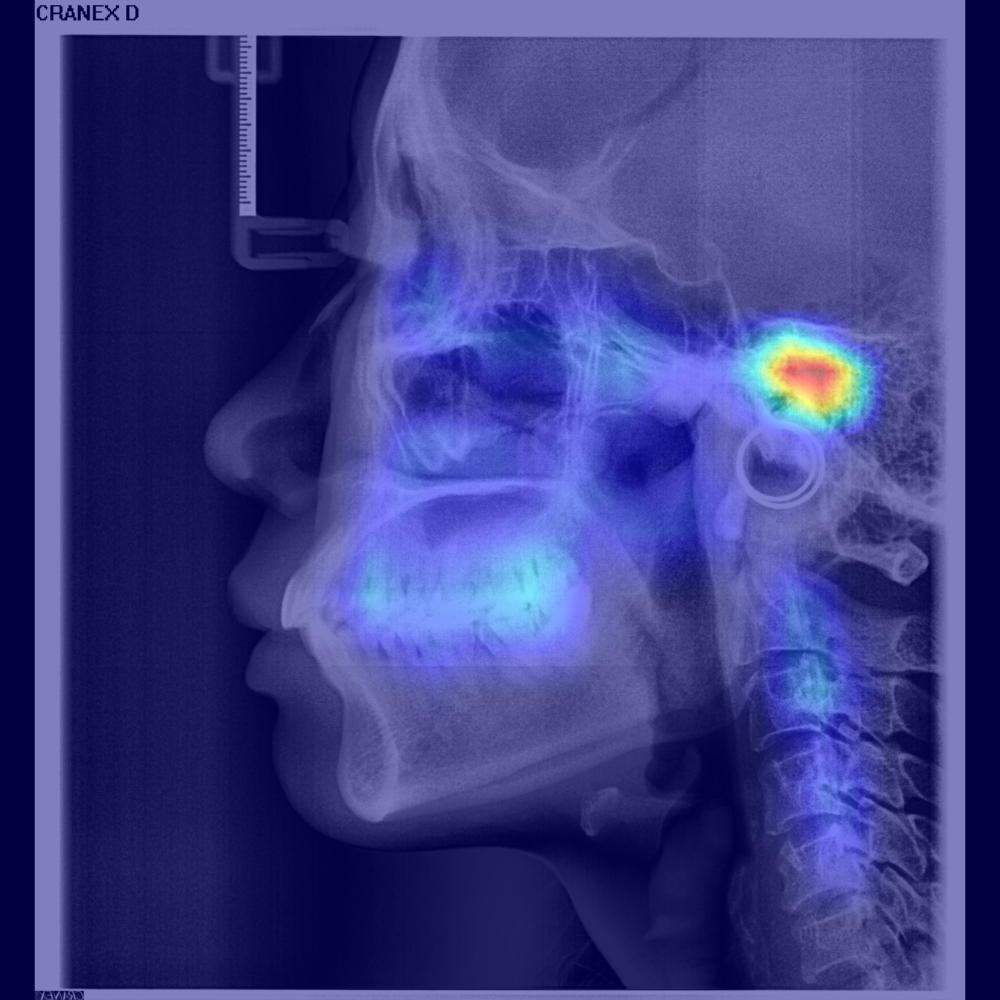

Supplement: Supplementary file 3 — Source Data File [file 41746_2022_681_MOESM3_ESM.zip › Ageing Saliency Map/40.png]

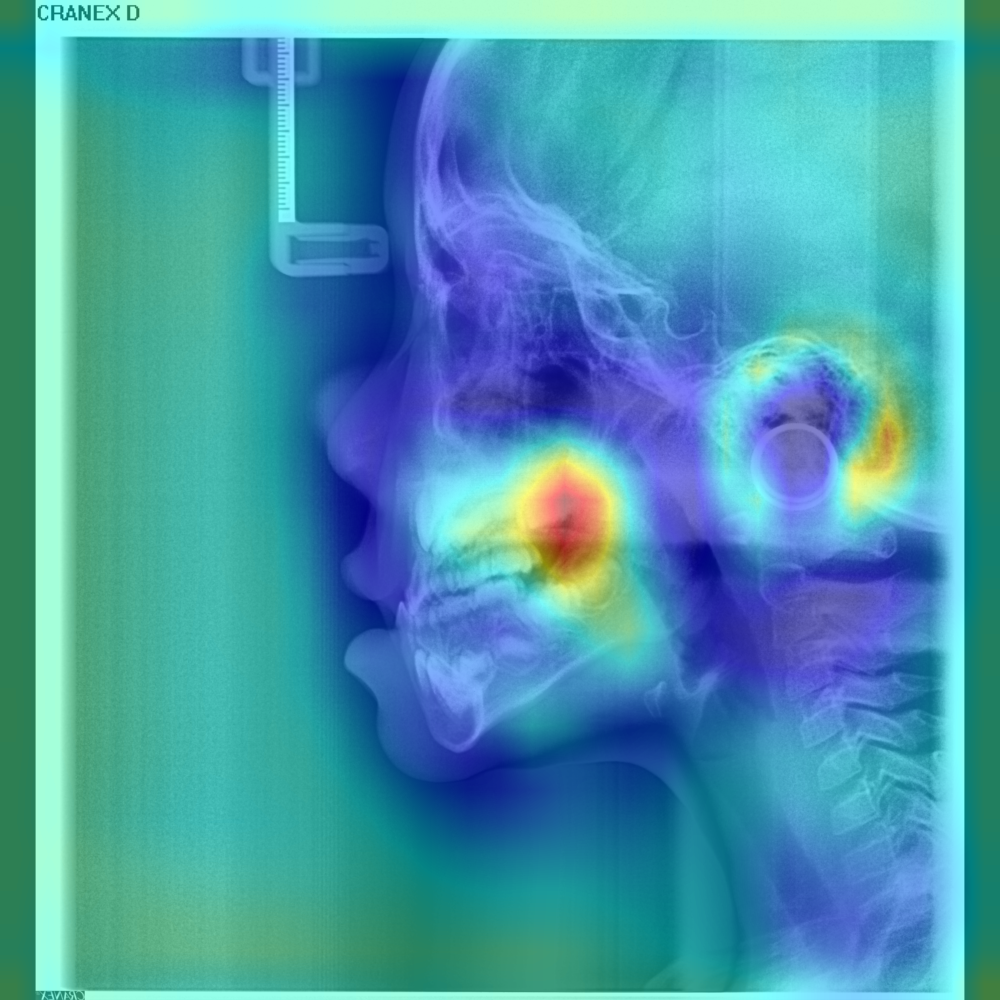

Supplement: Supplementary file 3 — Source Data File [file 41746_2022_681_MOESM3_ESM.zip › Ageing Saliency Map/5.png]

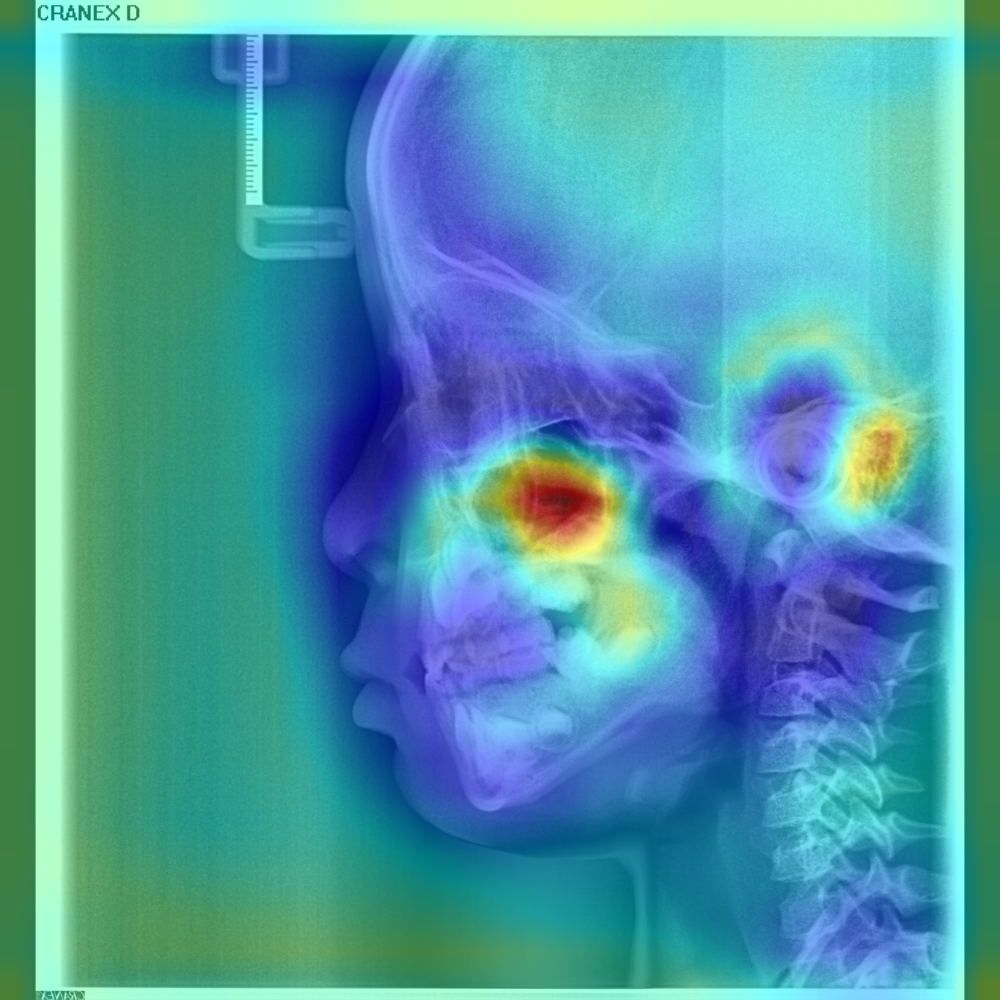

Supplement: Supplementary file 3 — Source Data File [file 41746_2022_681_MOESM3_ESM.zip › Ageing Saliency Map/6.png]

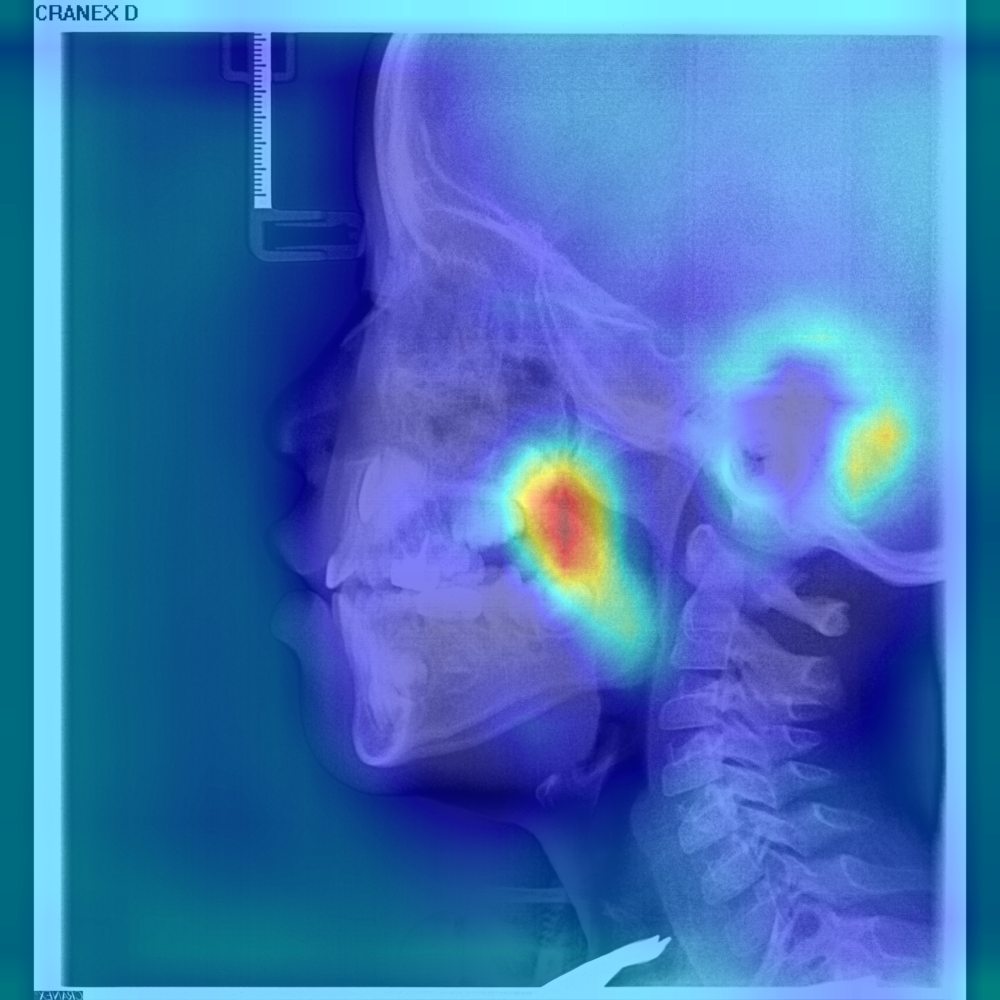

Supplement: Supplementary file 3 — Source Data File [file 41746_2022_681_MOESM3_ESM.zip › Ageing Saliency Map/7.png]

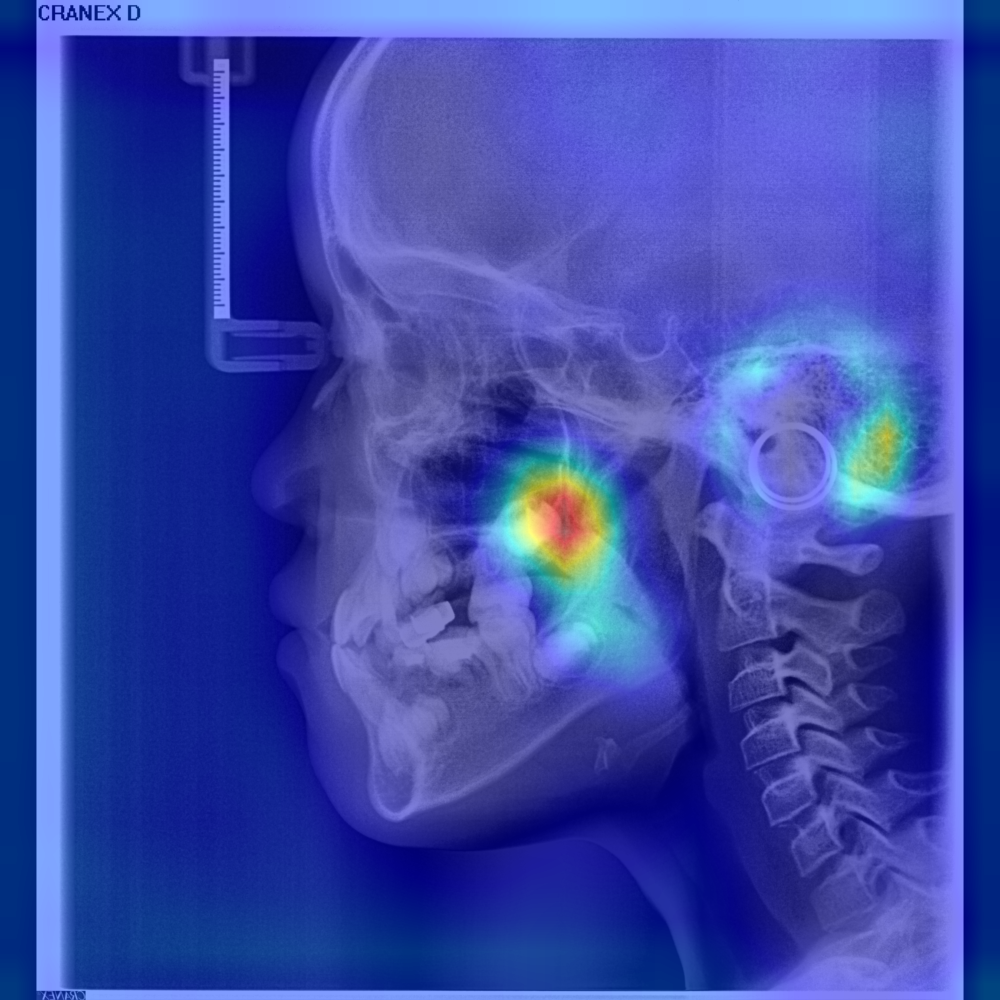

Supplement: Supplementary file 3 — Source Data File [file 41746_2022_681_MOESM3_ESM.zip › Ageing Saliency Map/8.png]

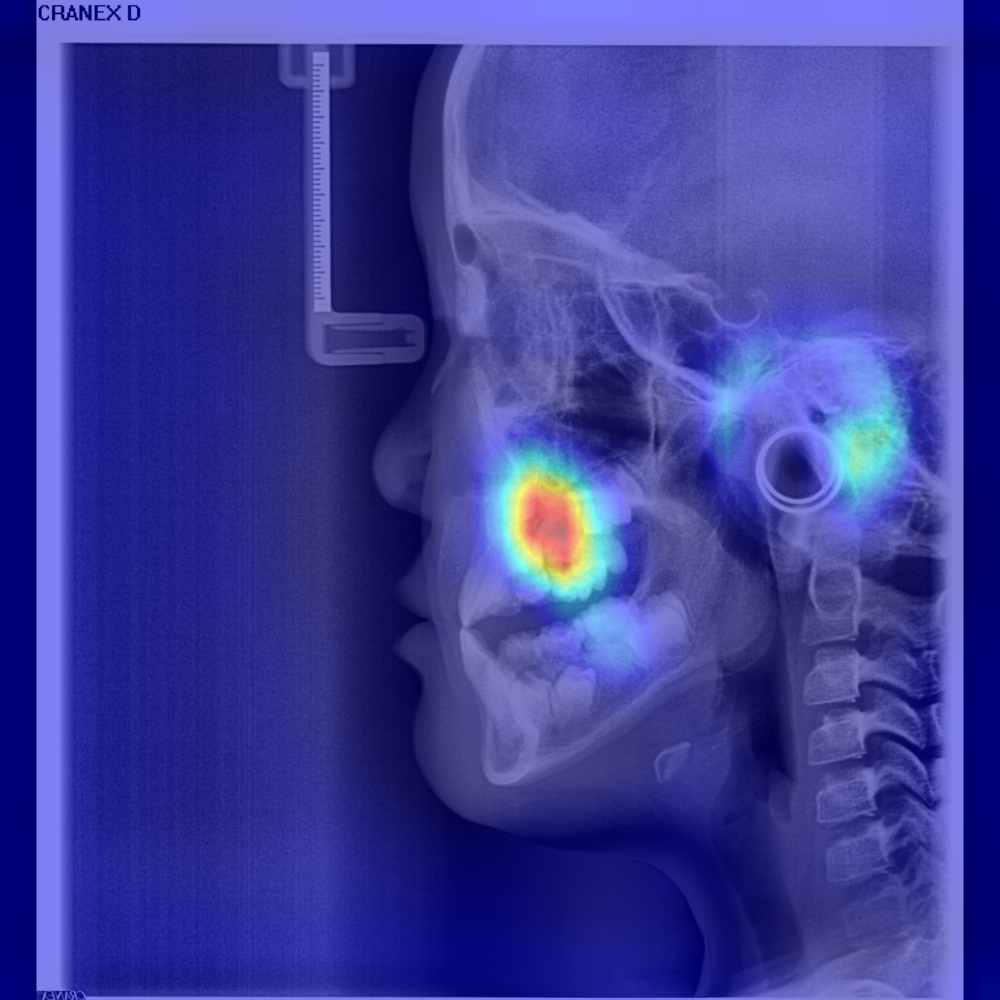

Supplement: Supplementary file 3 — Source Data File [file 41746_2022_681_MOESM3_ESM.zip › Ageing Saliency Map/9.png]

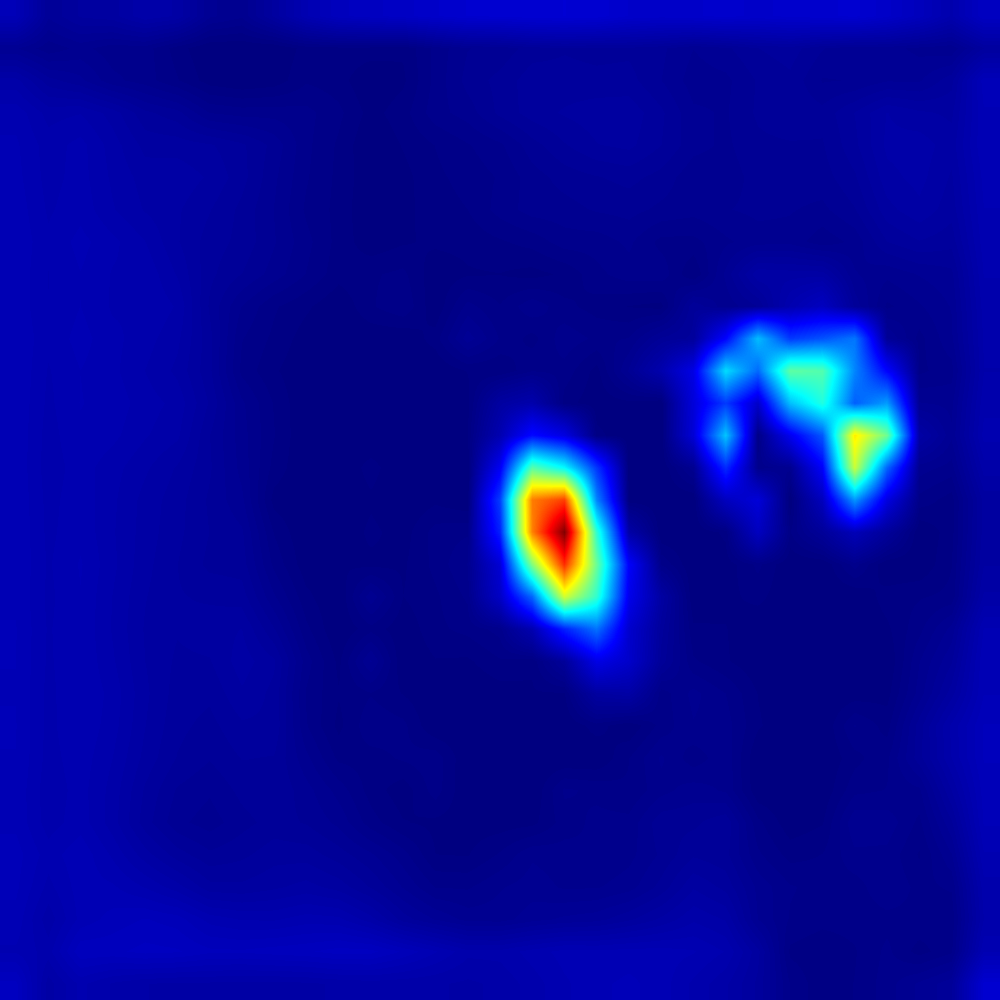

Supplement: Supplementary file 3 — Source Data File [file 41746_2022_681_MOESM3_ESM.zip › ARDA Map/Figure 1c/10.png]

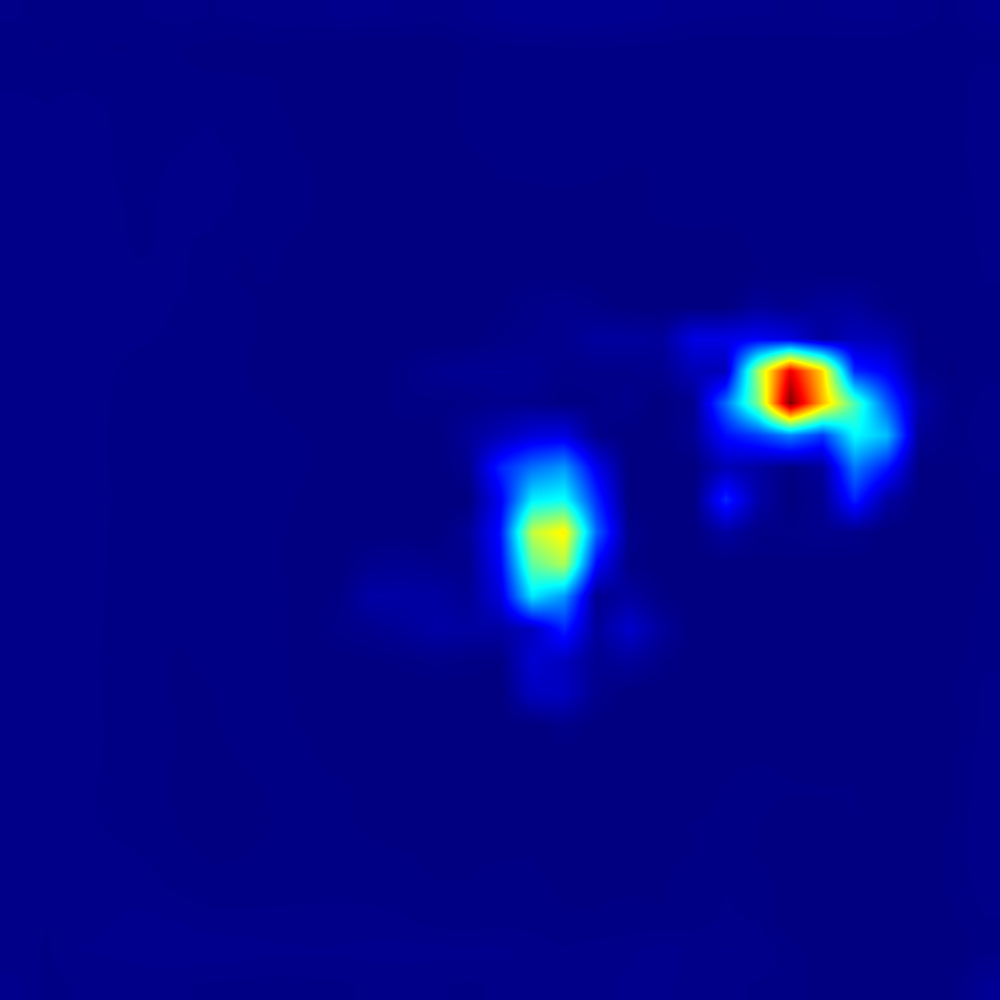

Supplement: Supplementary file 3 — Source Data File [file 41746_2022_681_MOESM3_ESM.zip › ARDA Map/Figure 1c/11.png]

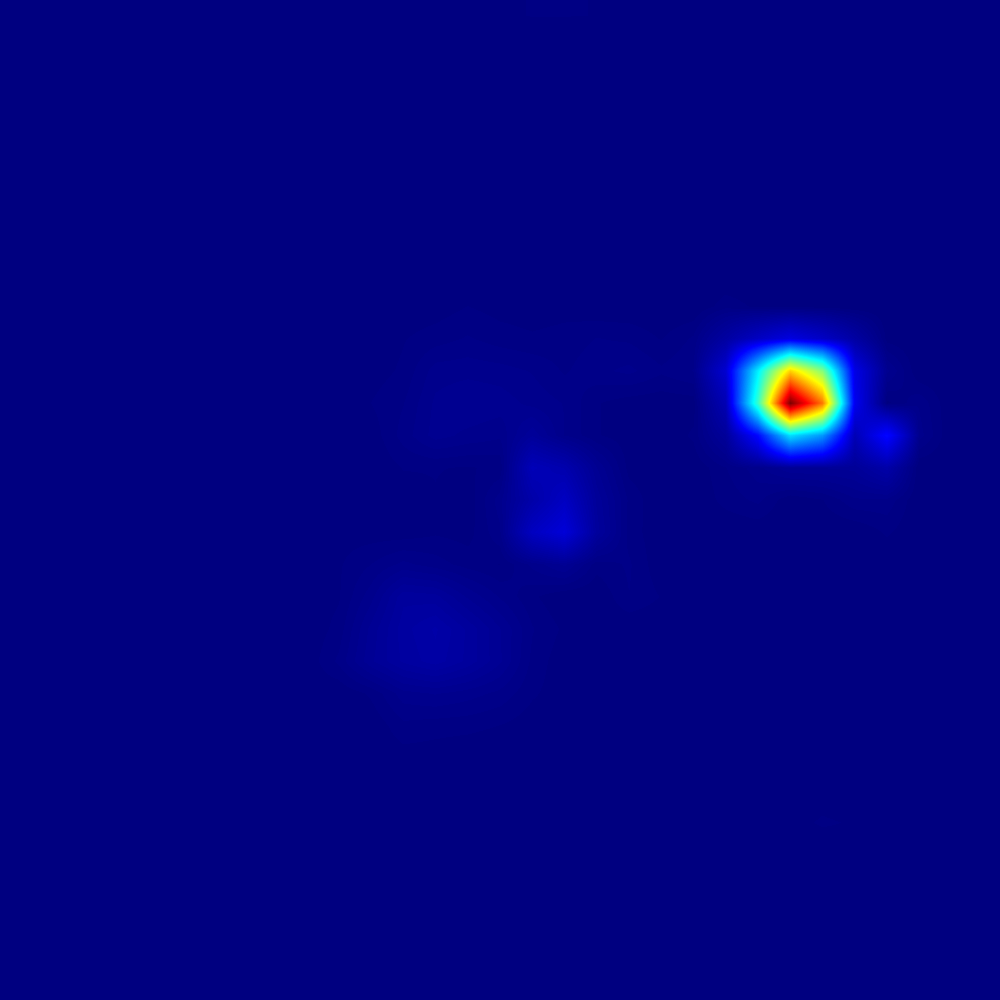

Supplement: Supplementary file 3 — Source Data File [file 41746_2022_681_MOESM3_ESM.zip › ARDA Map/Figure 1c/12.png]

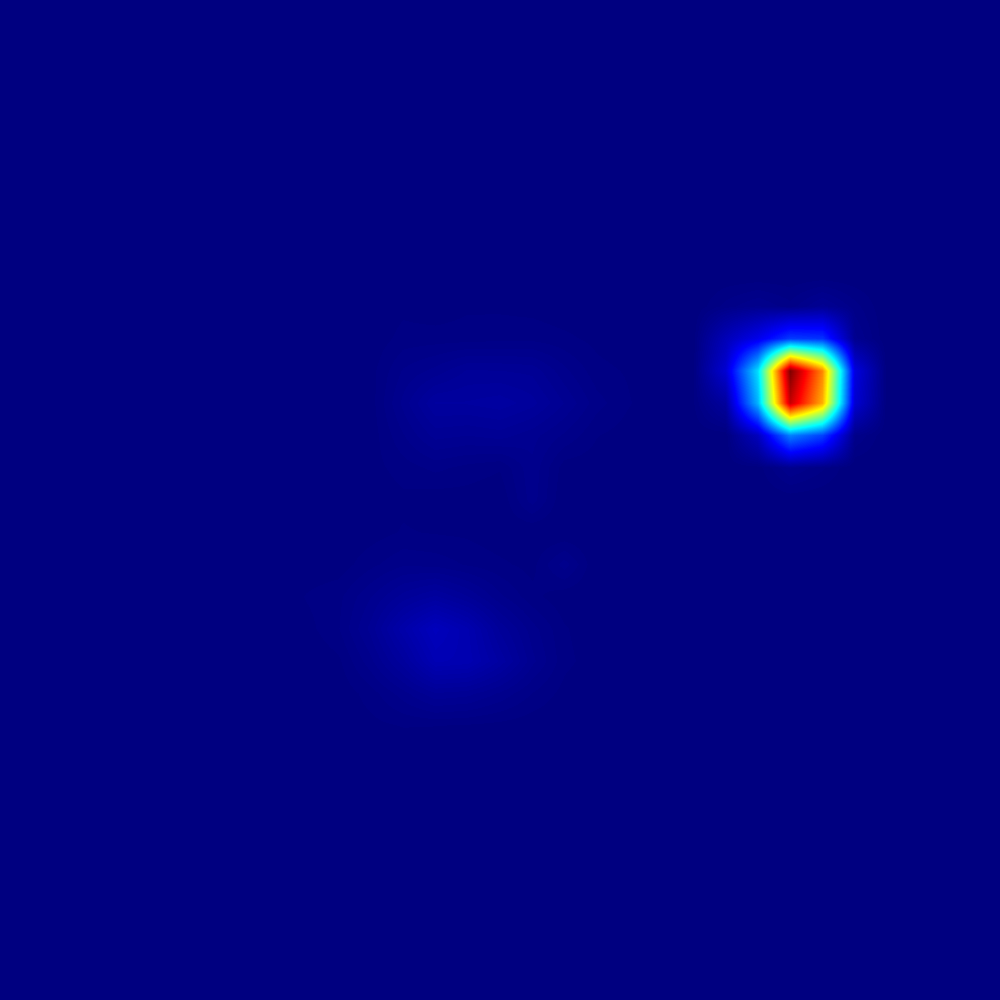

Supplement: Supplementary file 3 — Source Data File [file 41746_2022_681_MOESM3_ESM.zip › ARDA Map/Figure 1c/13.png]

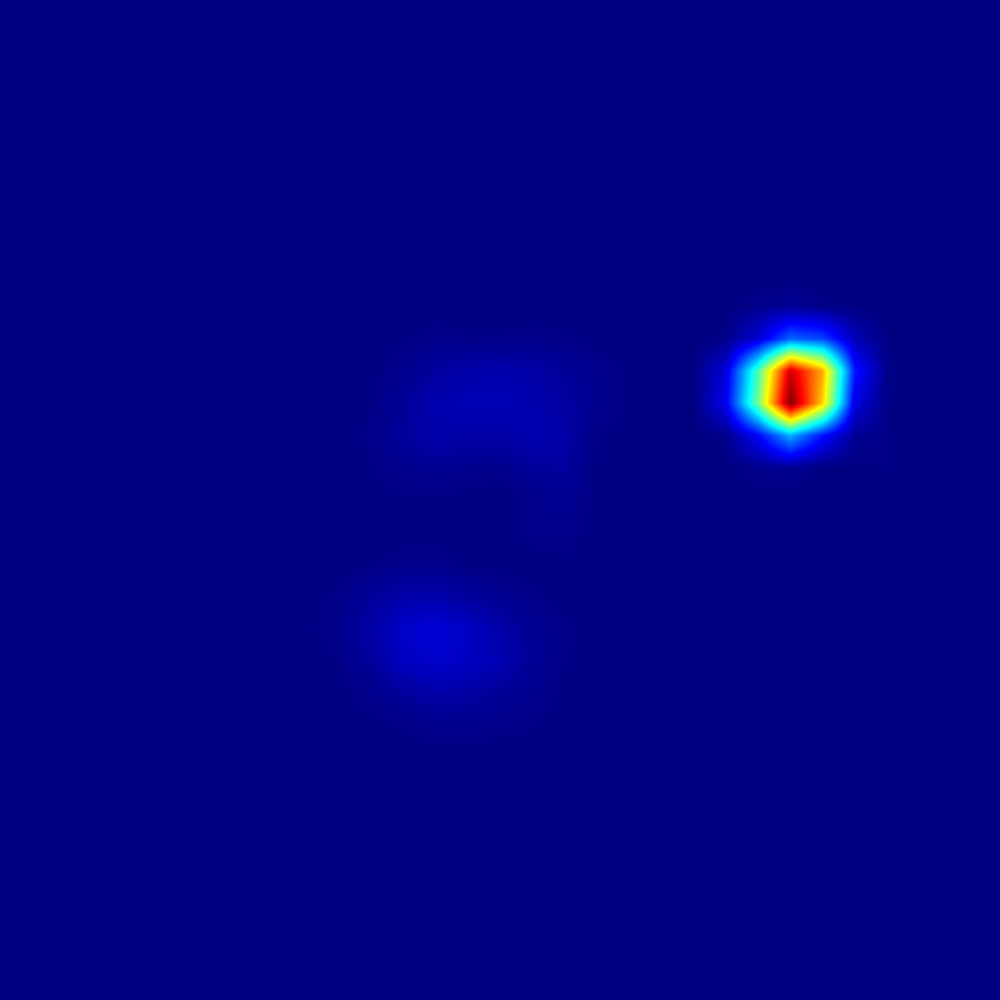

Supplement: Supplementary file 3 — Source Data File [file 41746_2022_681_MOESM3_ESM.zip › ARDA Map/Figure 1c/14.png]

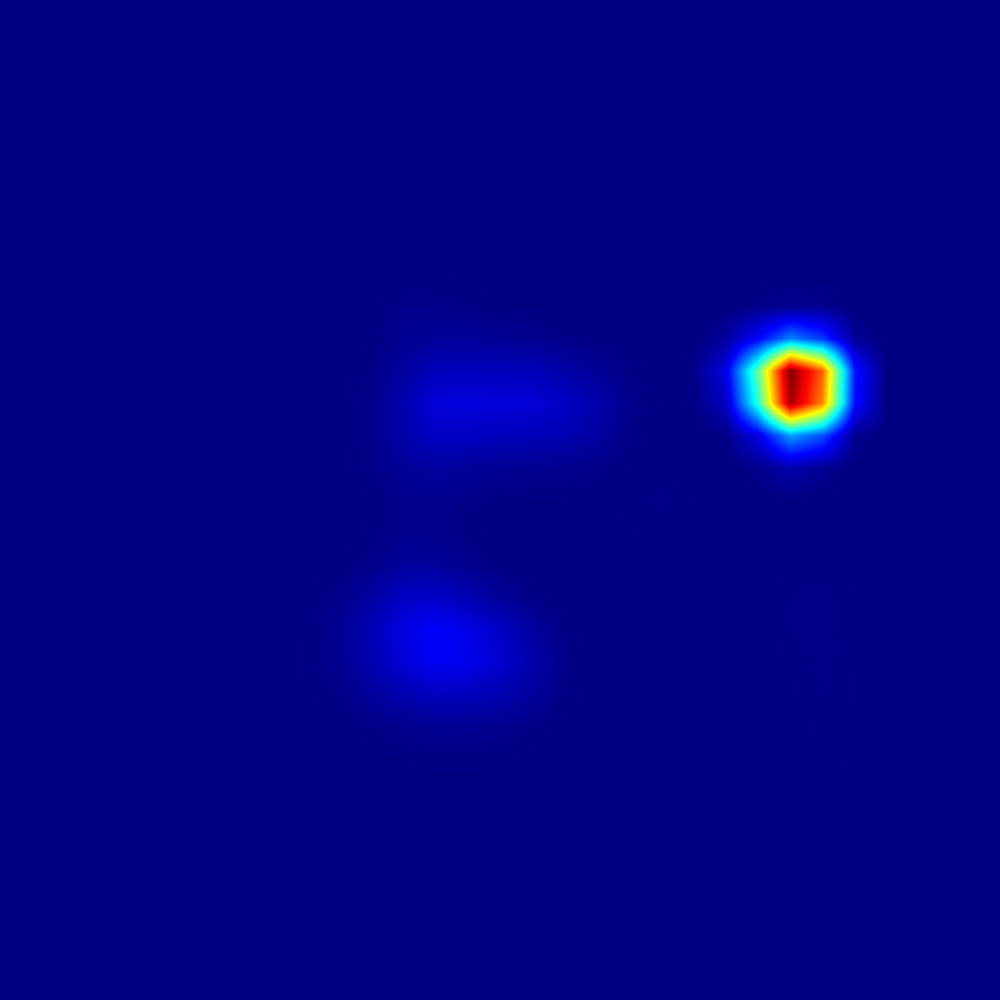

Supplement: Supplementary file 3 — Source Data File [file 41746_2022_681_MOESM3_ESM.zip › ARDA Map/Figure 1c/15.png]

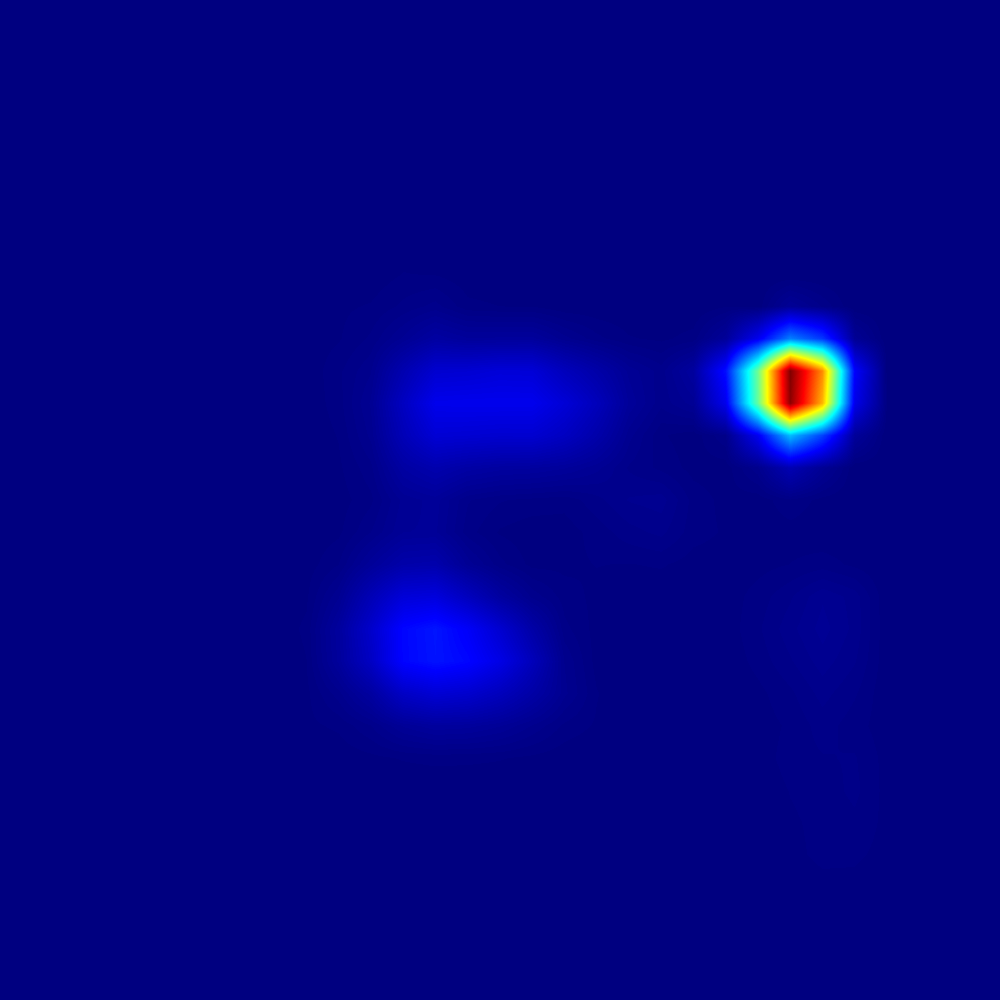

Supplement: Supplementary file 3 — Source Data File [file 41746_2022_681_MOESM3_ESM.zip › ARDA Map/Figure 1c/16.png]

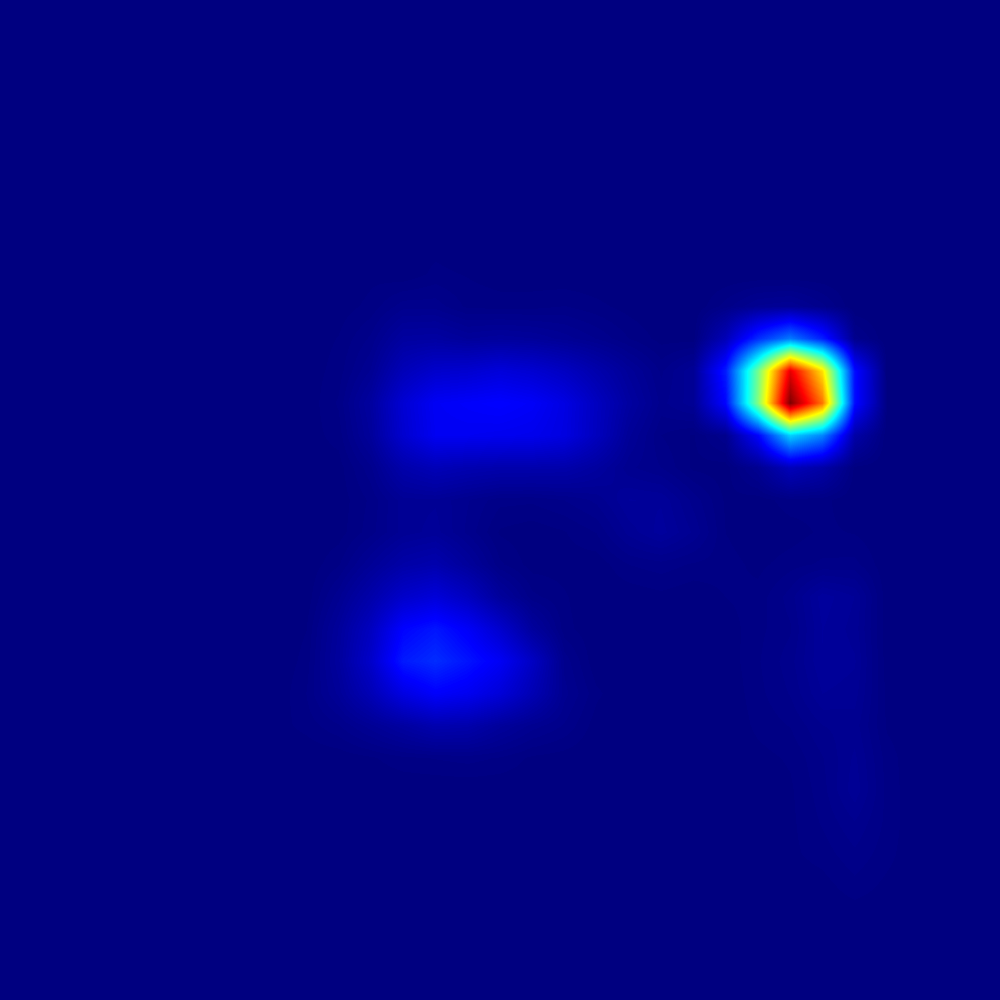

Supplement: Supplementary file 3 — Source Data File [file 41746_2022_681_MOESM3_ESM.zip › ARDA Map/Figure 1c/17.png]

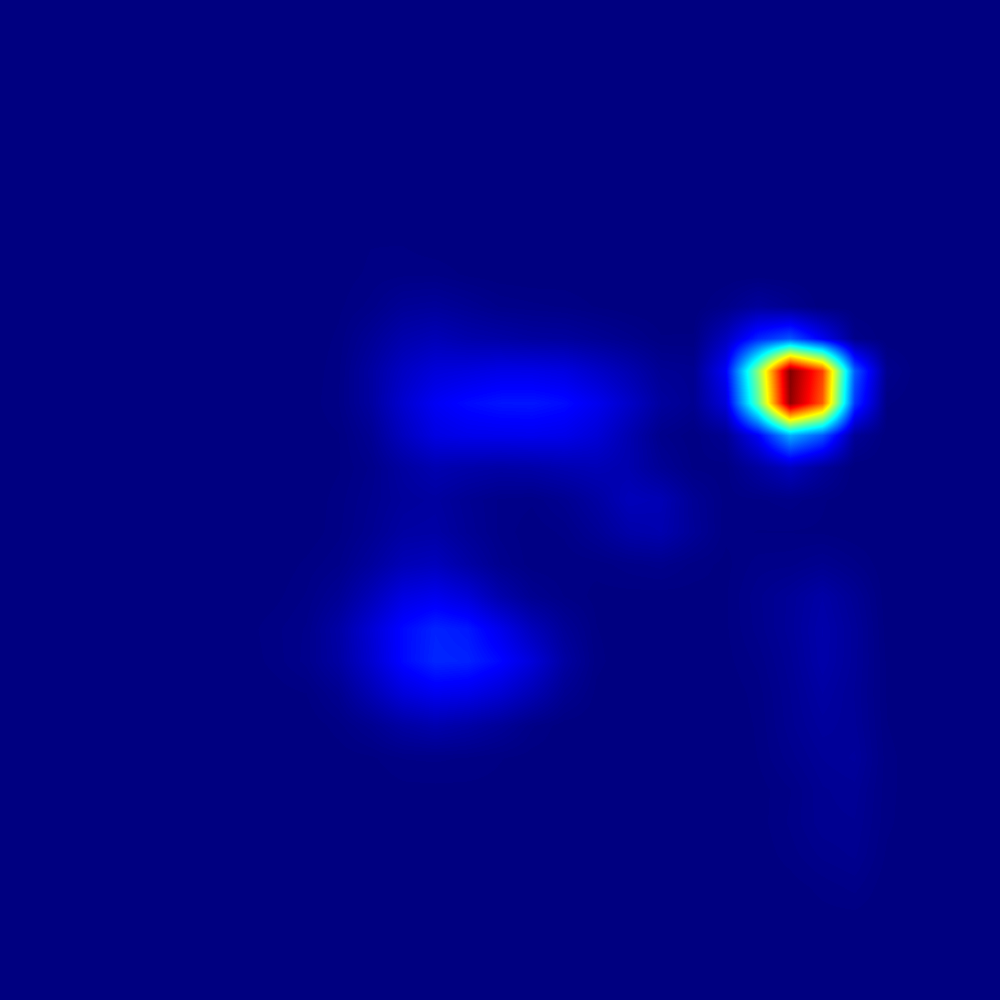

Supplement: Supplementary file 3 — Source Data File [file 41746_2022_681_MOESM3_ESM.zip › ARDA Map/Figure 1c/18.png]

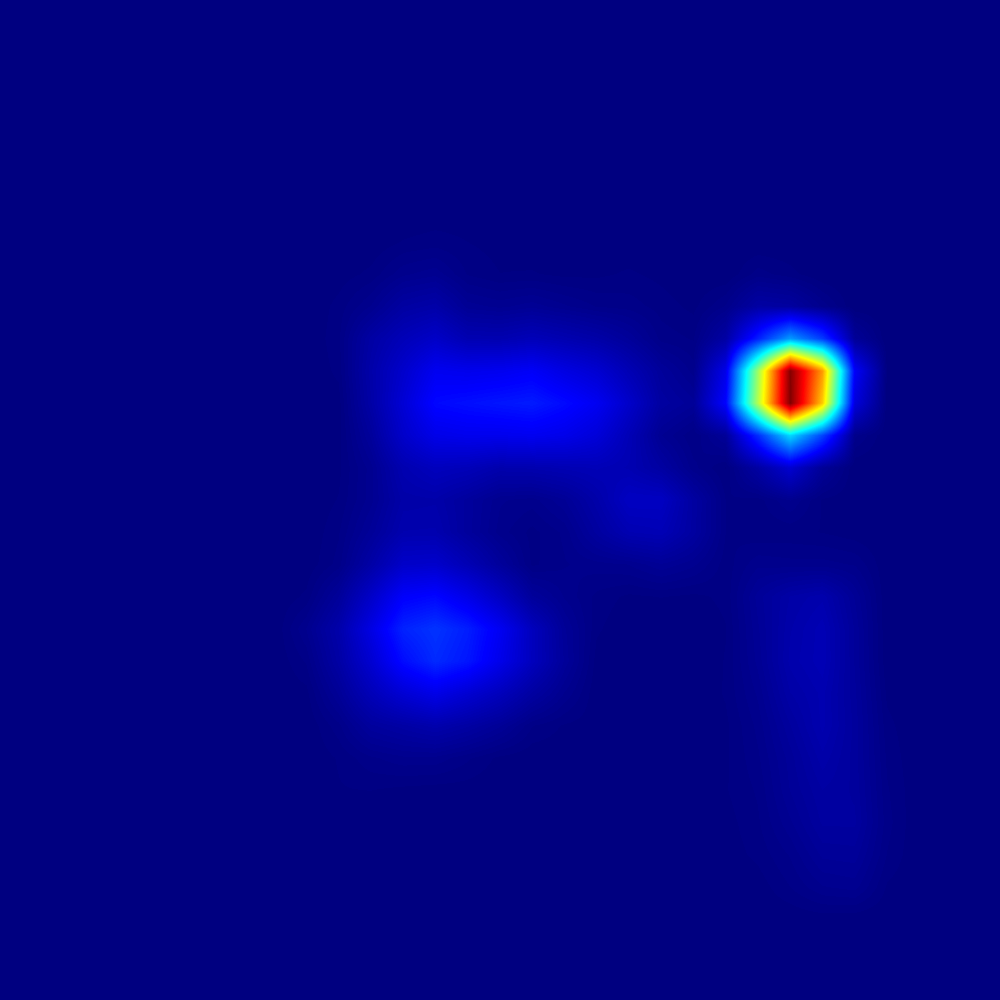

Supplement: Supplementary file 3 — Source Data File [file 41746_2022_681_MOESM3_ESM.zip › ARDA Map/Figure 1c/19.png]

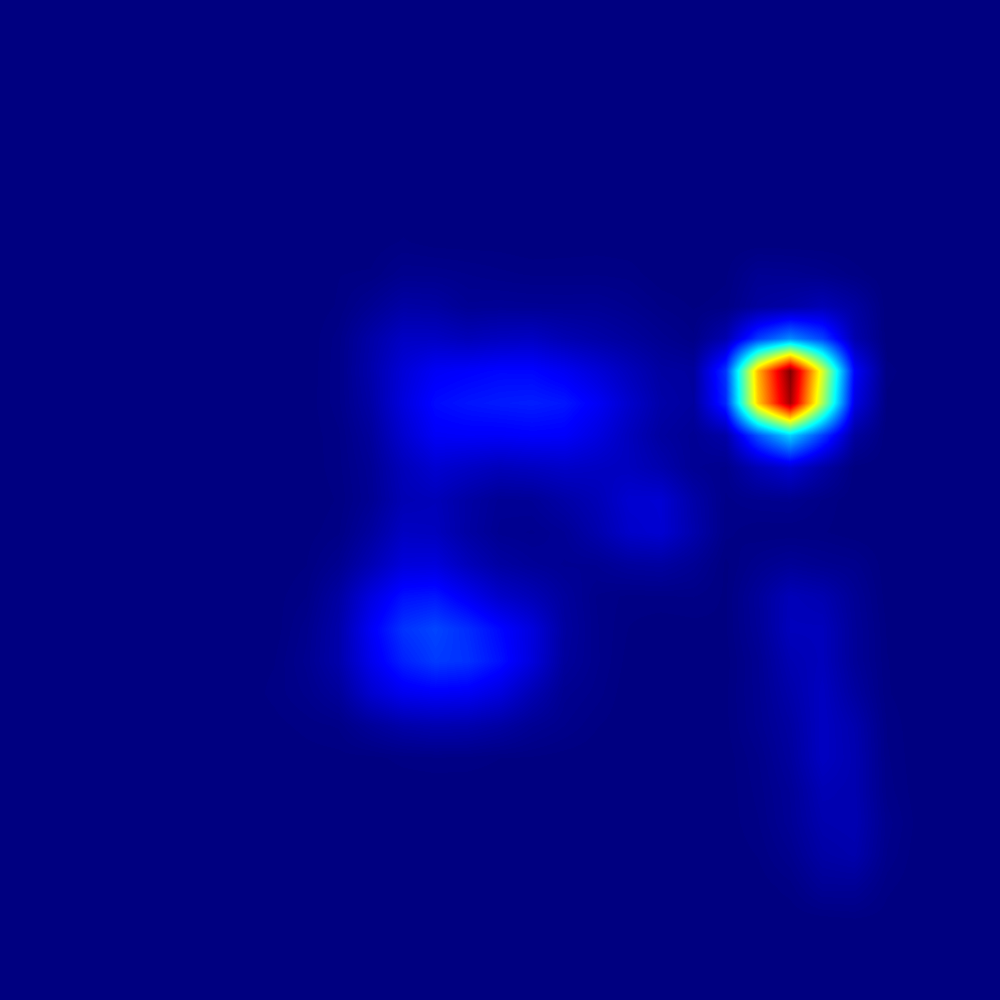

Supplement: Supplementary file 3 — Source Data File [file 41746_2022_681_MOESM3_ESM.zip › ARDA Map/Figure 1c/20.png]

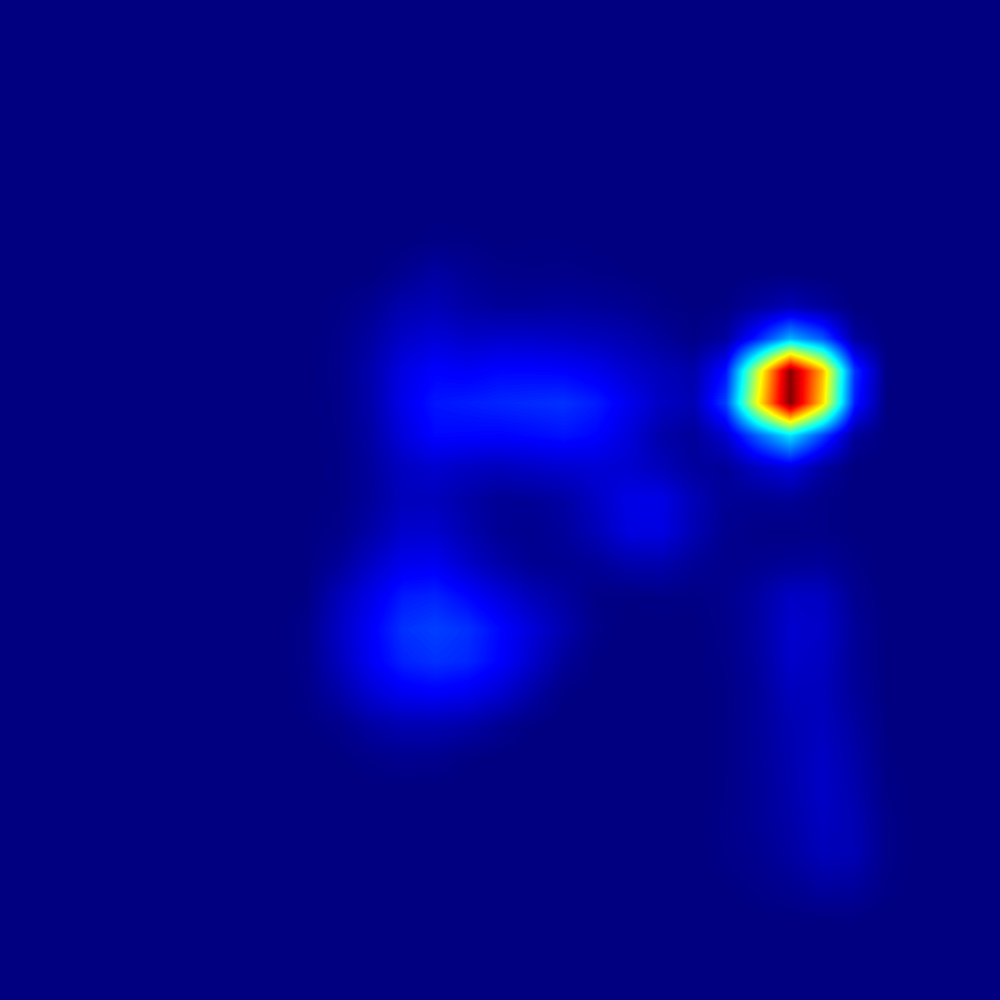

Supplement: Supplementary file 3 — Source Data File [file 41746_2022_681_MOESM3_ESM.zip › ARDA Map/Figure 1c/21.png]

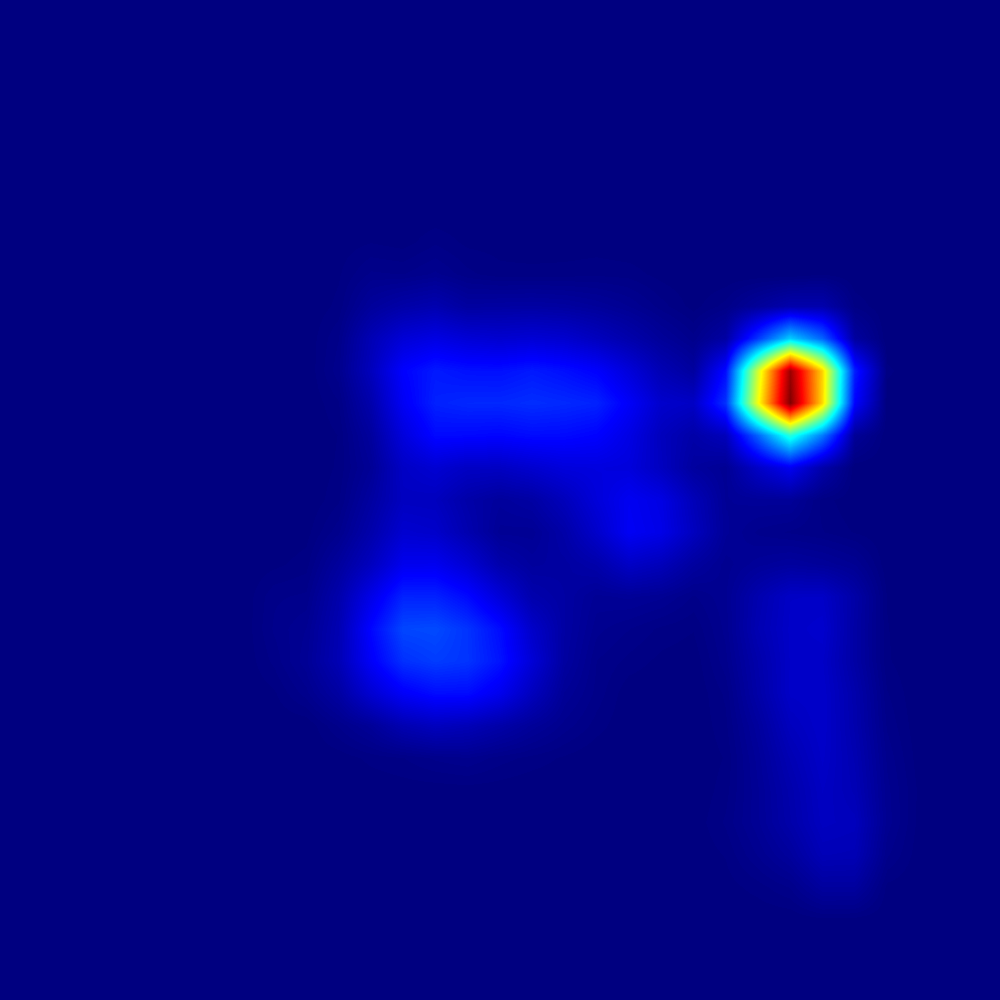

Supplement: Supplementary file 3 — Source Data File [file 41746_2022_681_MOESM3_ESM.zip › ARDA Map/Figure 1c/22.png]

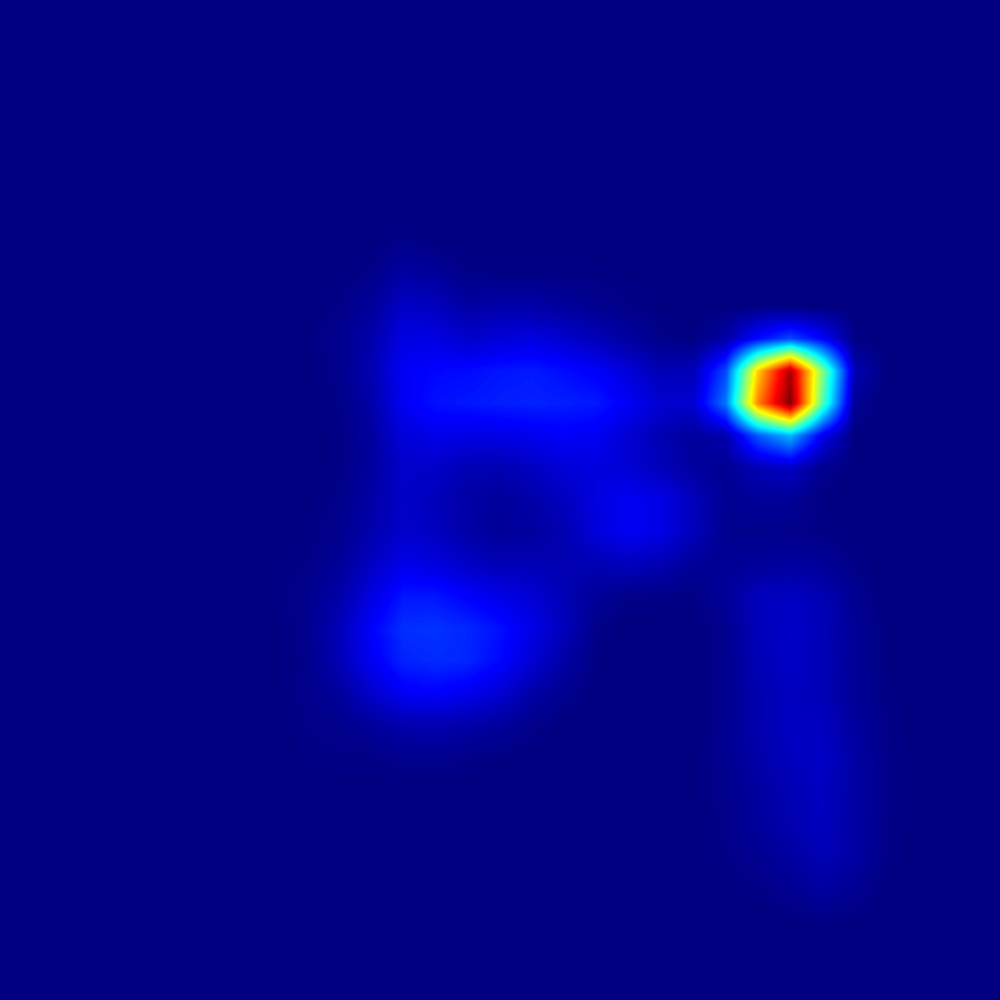

Supplement: Supplementary file 3 — Source Data File [file 41746_2022_681_MOESM3_ESM.zip › ARDA Map/Figure 1c/23.png]

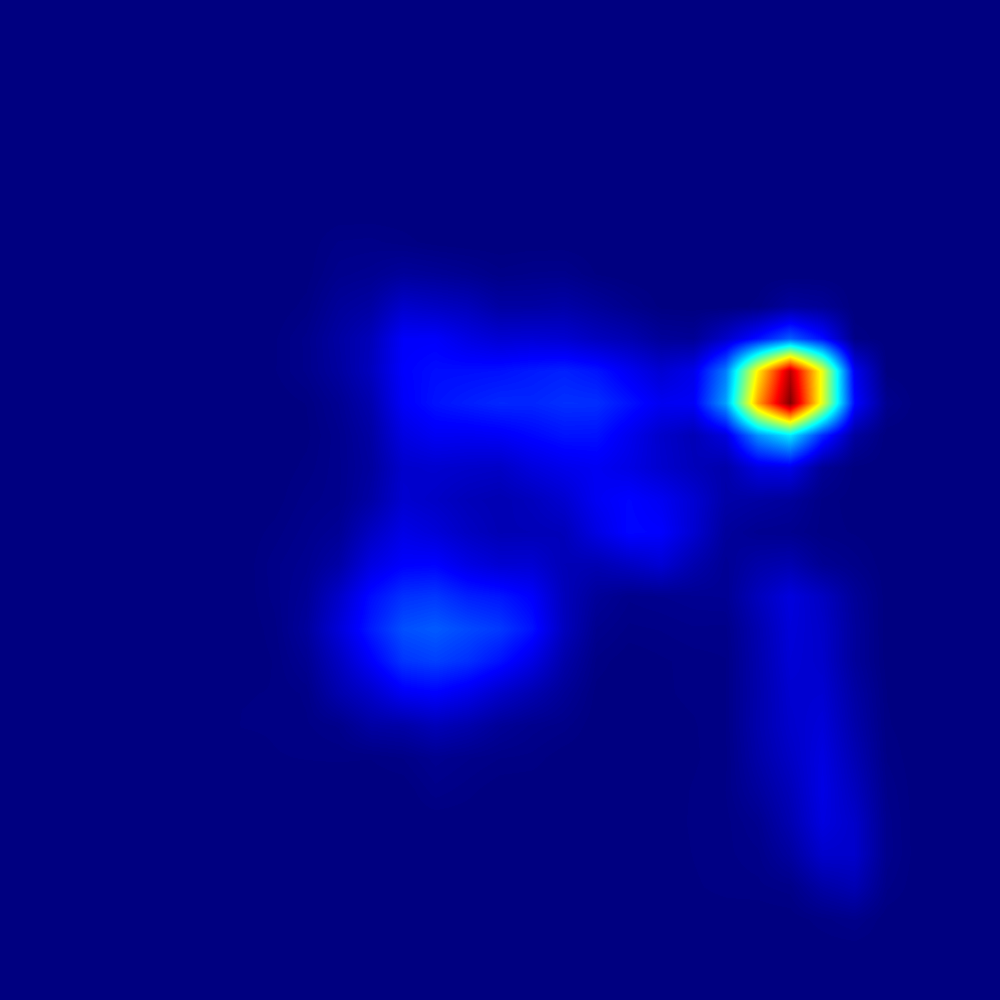

Supplement: Supplementary file 3 — Source Data File [file 41746_2022_681_MOESM3_ESM.zip › ARDA Map/Figure 1c/24.png]

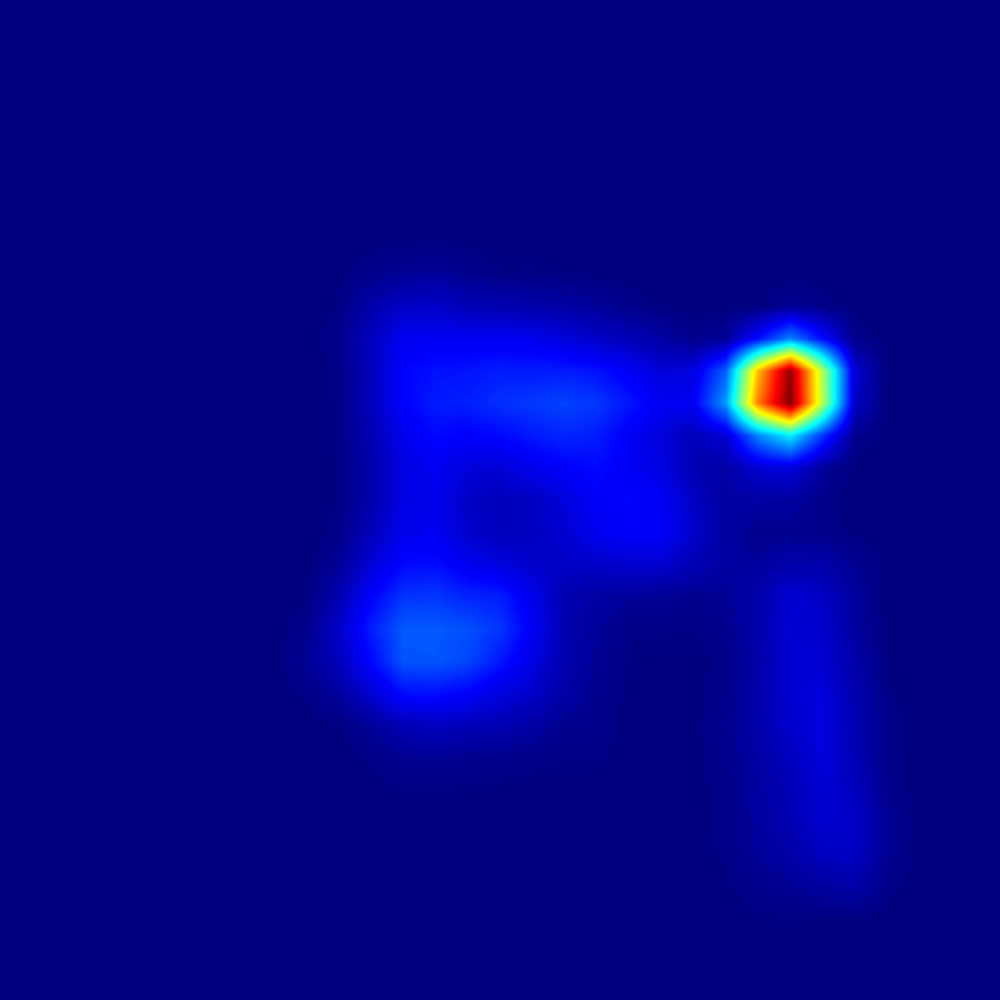

Supplement: Supplementary file 3 — Source Data File [file 41746_2022_681_MOESM3_ESM.zip › ARDA Map/Figure 1c/25.png]

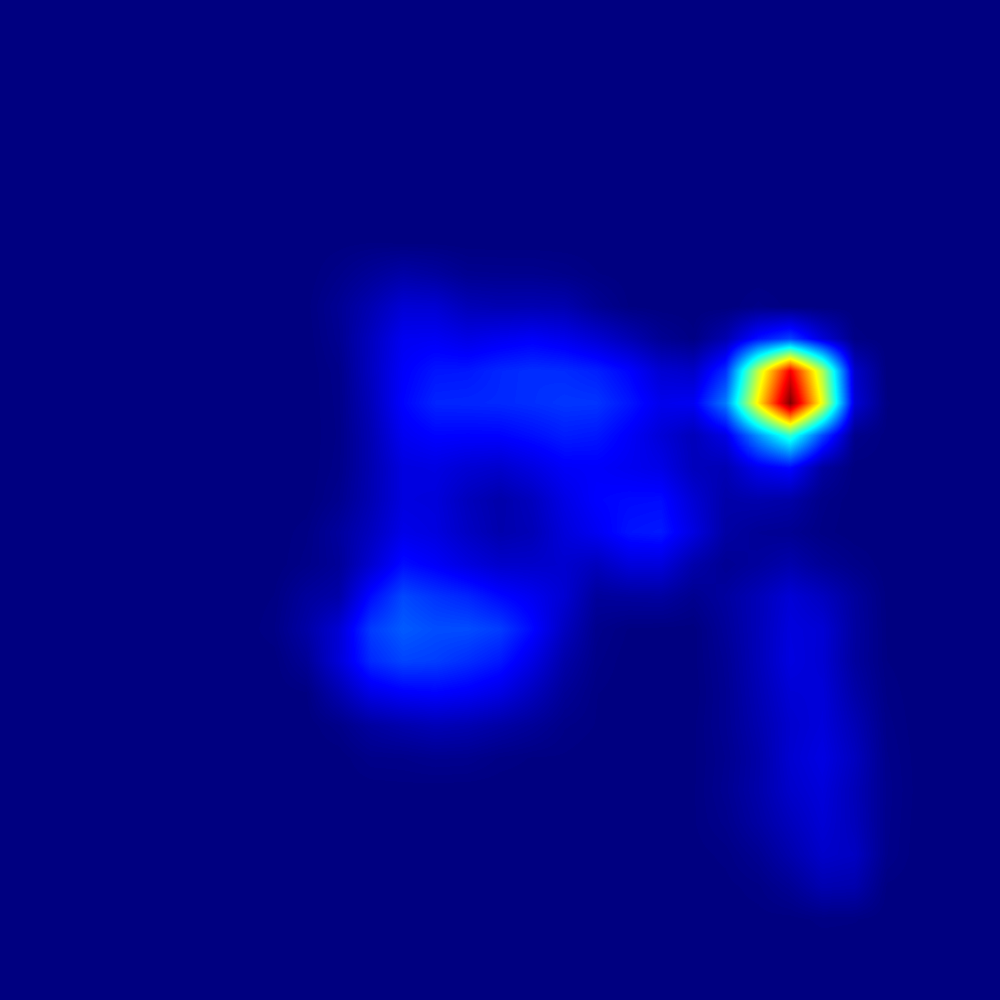

Supplement: Supplementary file 3 — Source Data File [file 41746_2022_681_MOESM3_ESM.zip › ARDA Map/Figure 1c/26.png]

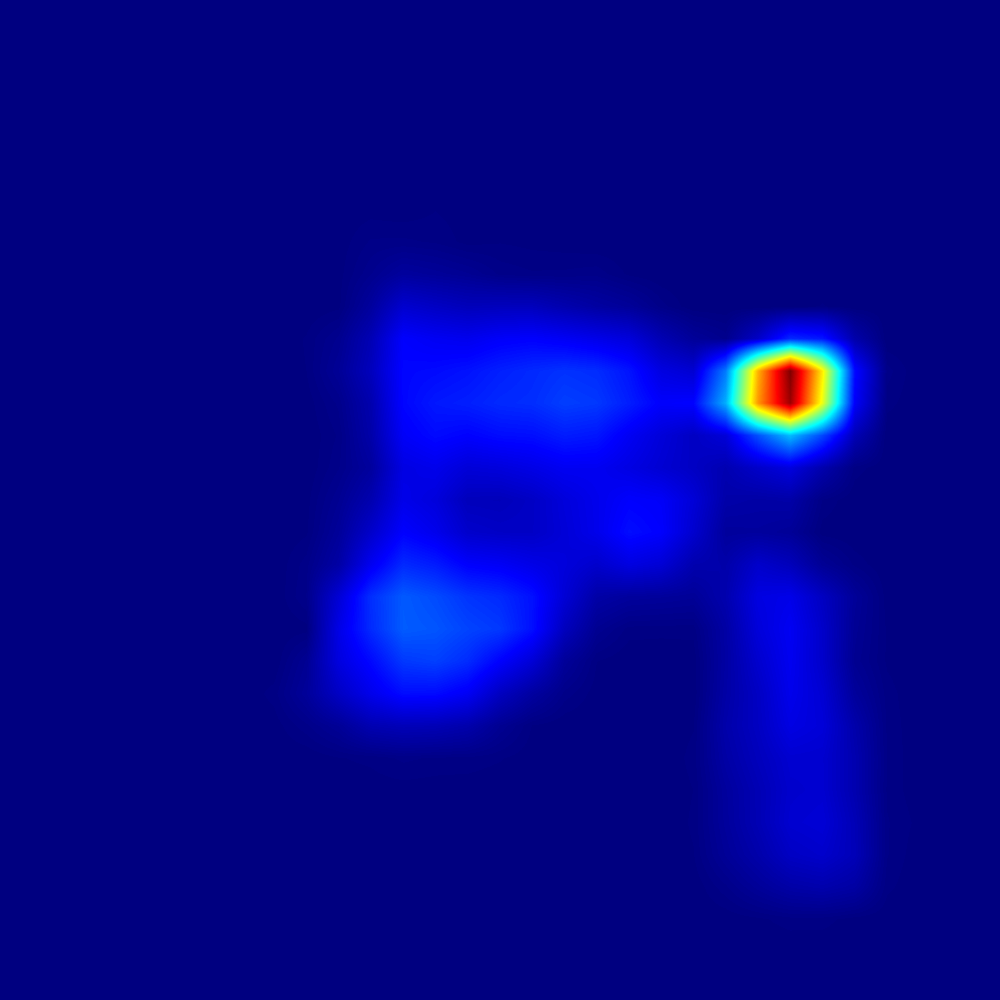

Supplement: Supplementary file 3 — Source Data File [file 41746_2022_681_MOESM3_ESM.zip › ARDA Map/Figure 1c/27.png]

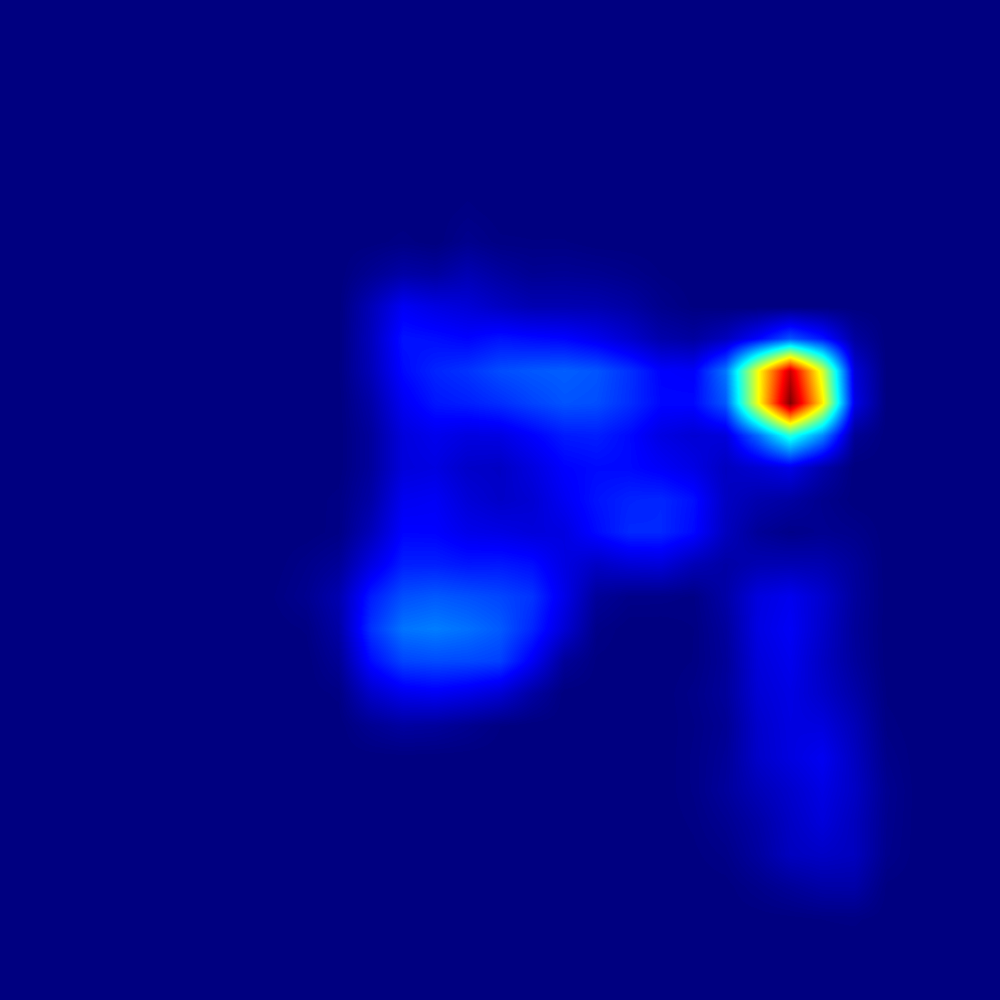

Supplement: Supplementary file 3 — Source Data File [file 41746_2022_681_MOESM3_ESM.zip › ARDA Map/Figure 1c/28.png]

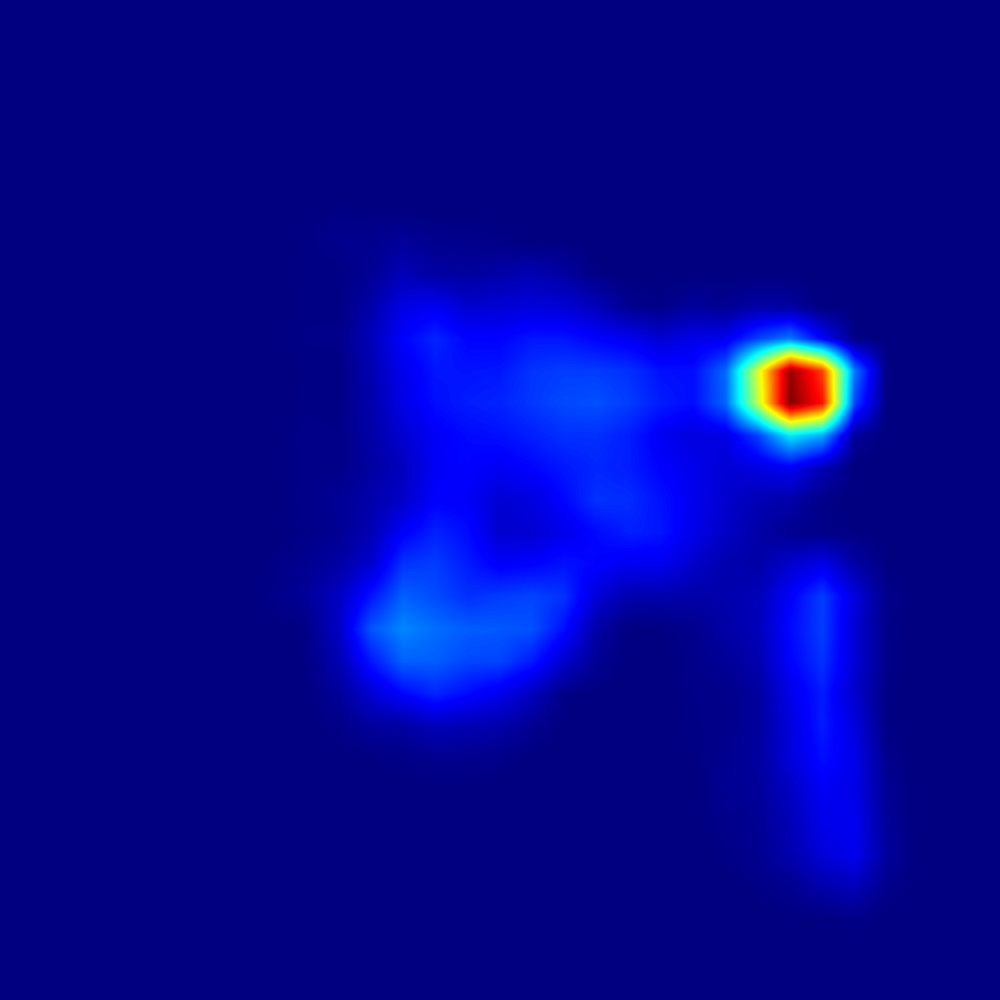

Supplement: Supplementary file 3 — Source Data File [file 41746_2022_681_MOESM3_ESM.zip › ARDA Map/Figure 1c/29.png]

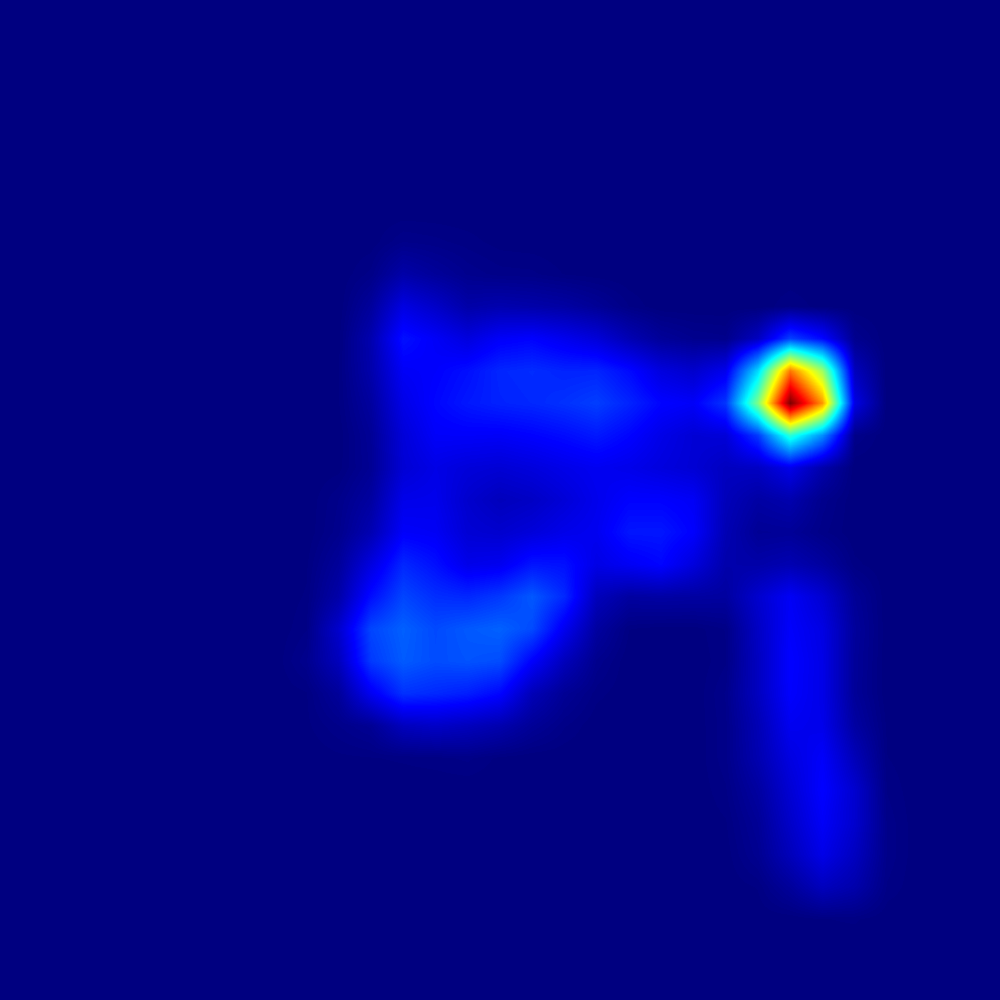

Supplement: Supplementary file 3 — Source Data File [file 41746_2022_681_MOESM3_ESM.zip › ARDA Map/Figure 1c/30.png]

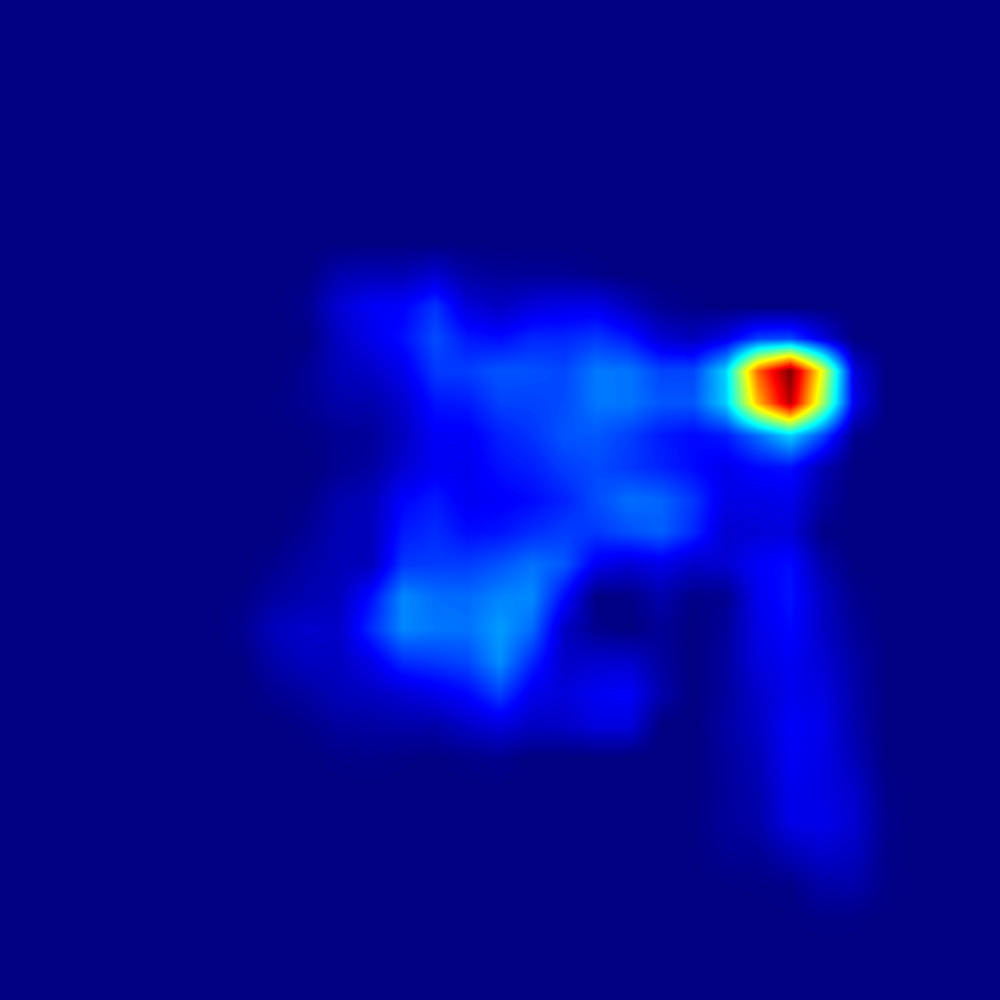

Supplement: Supplementary file 3 — Source Data File [file 41746_2022_681_MOESM3_ESM.zip › ARDA Map/Figure 1c/31.png]

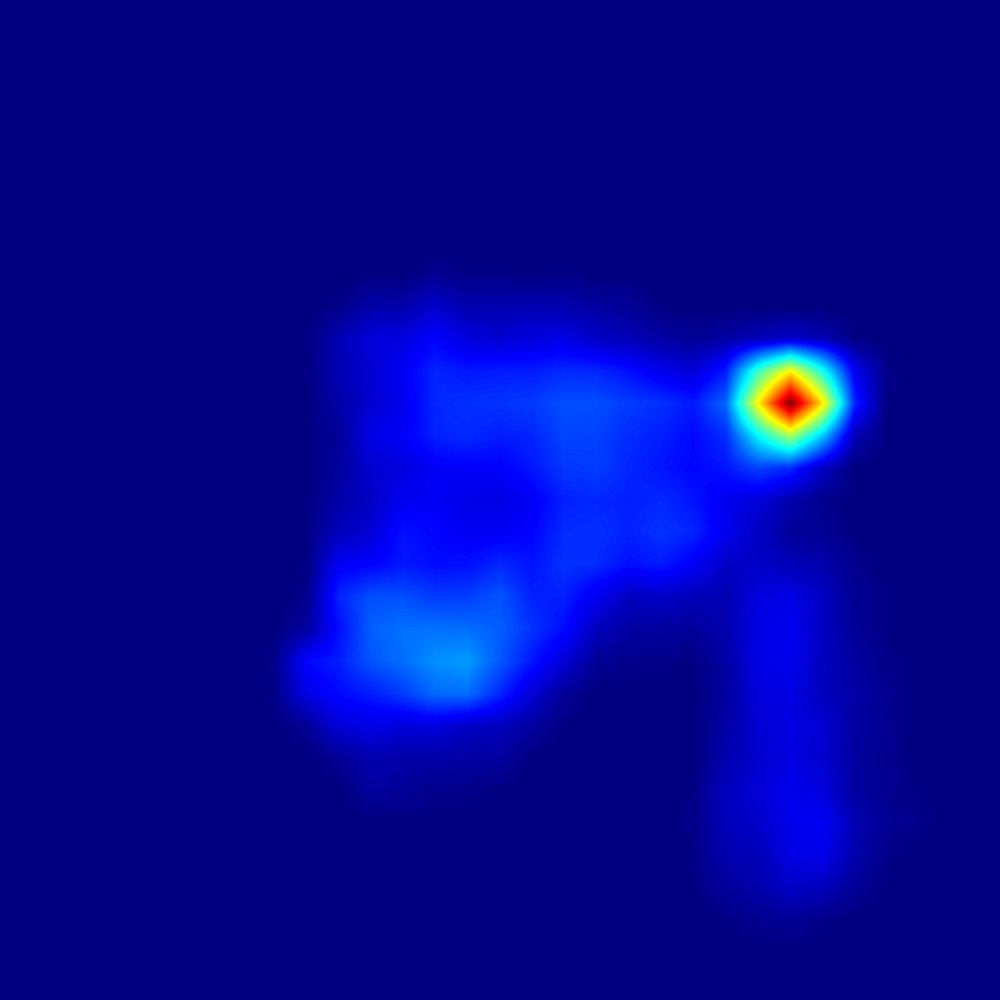

Supplement: Supplementary file 3 — Source Data File [file 41746_2022_681_MOESM3_ESM.zip › ARDA Map/Figure 1c/32.png]

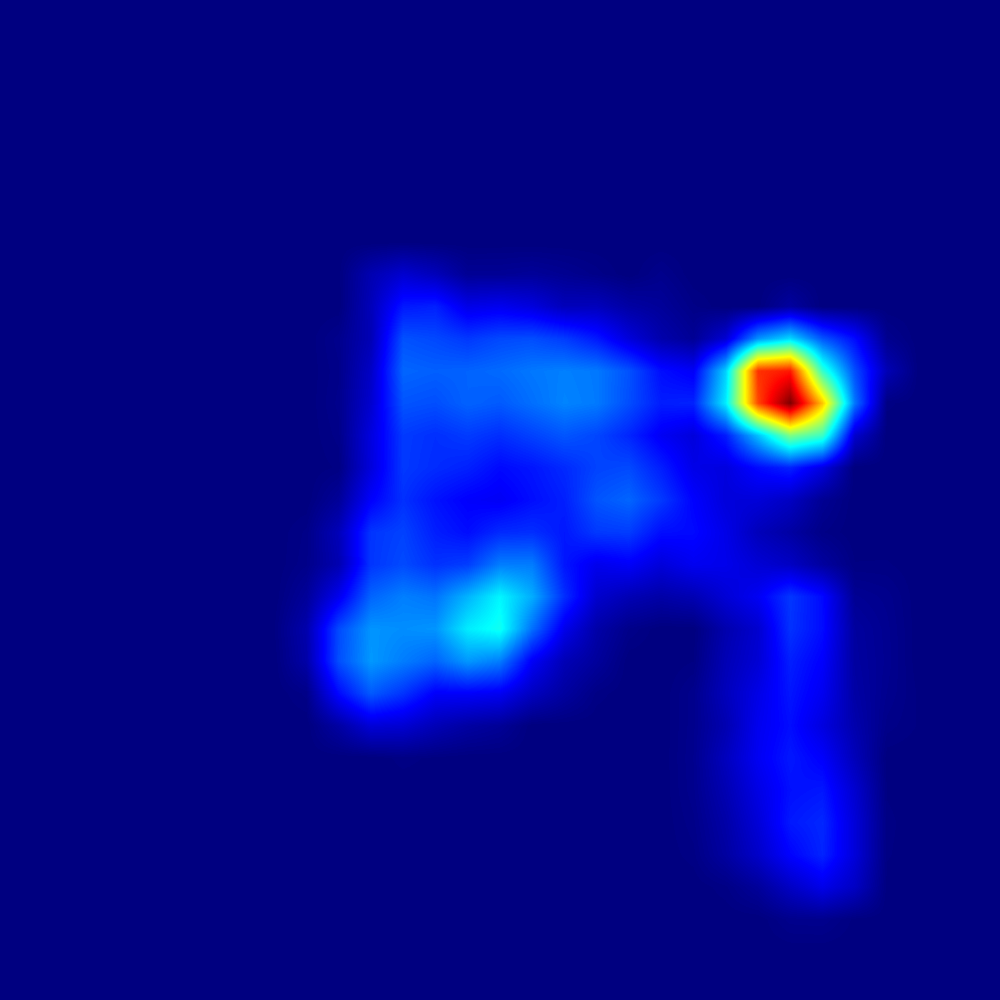

Supplement: Supplementary file 3 — Source Data File [file 41746_2022_681_MOESM3_ESM.zip › ARDA Map/Figure 1c/33.png]

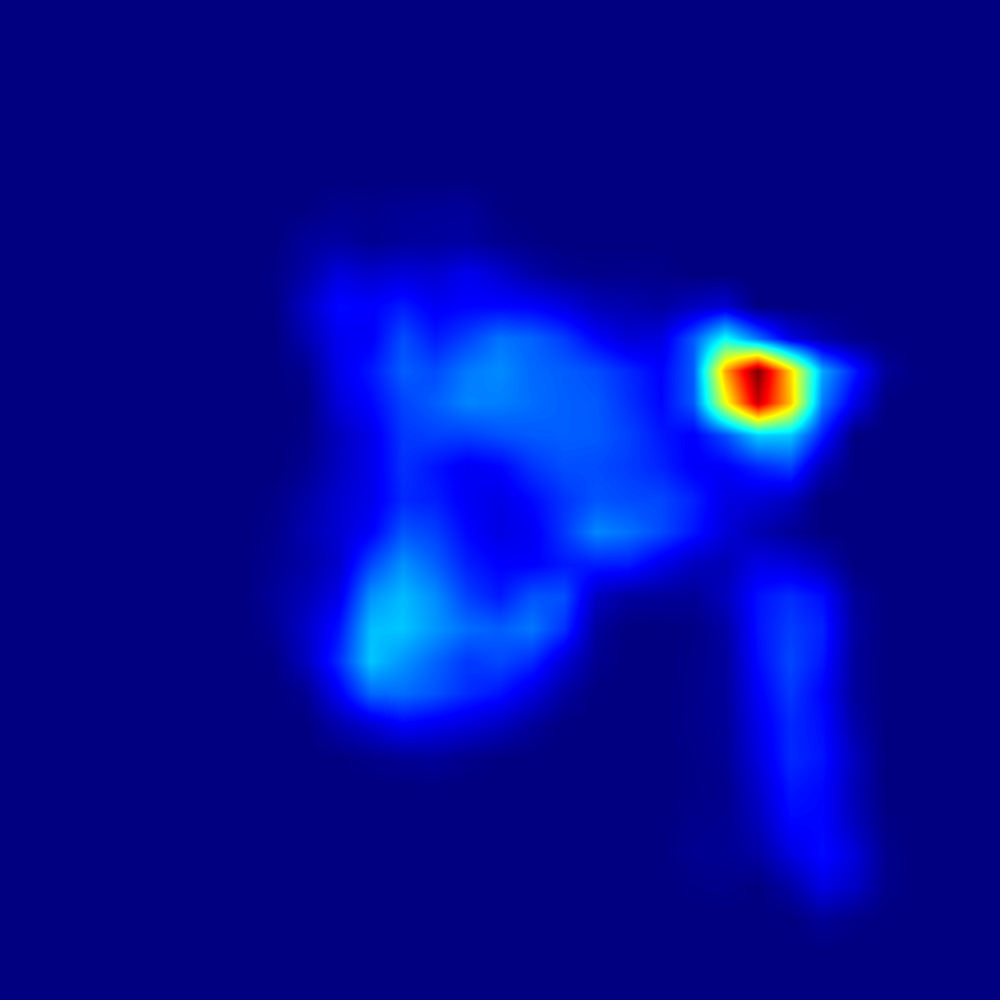

Supplement: Supplementary file 3 — Source Data File [file 41746_2022_681_MOESM3_ESM.zip › ARDA Map/Figure 1c/34.png]

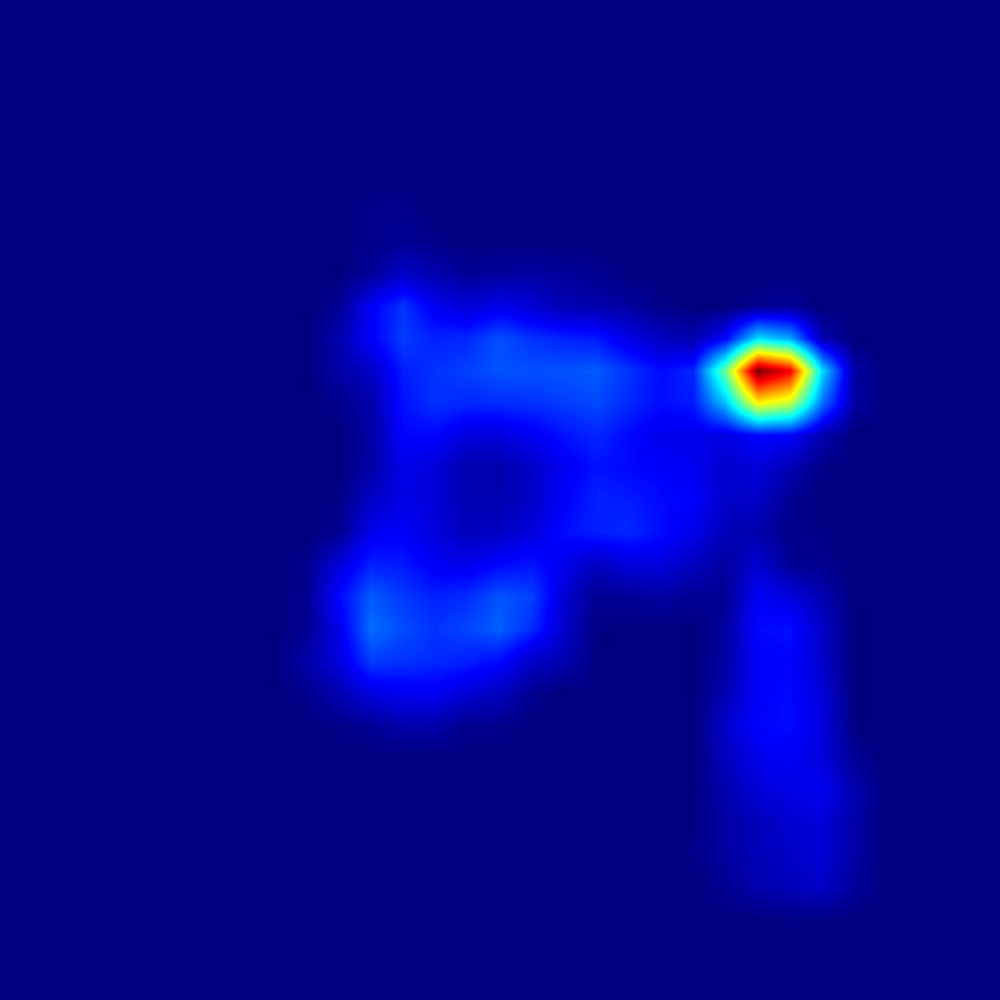

Supplement: Supplementary file 3 — Source Data File [file 41746_2022_681_MOESM3_ESM.zip › ARDA Map/Figure 1c/35.png]

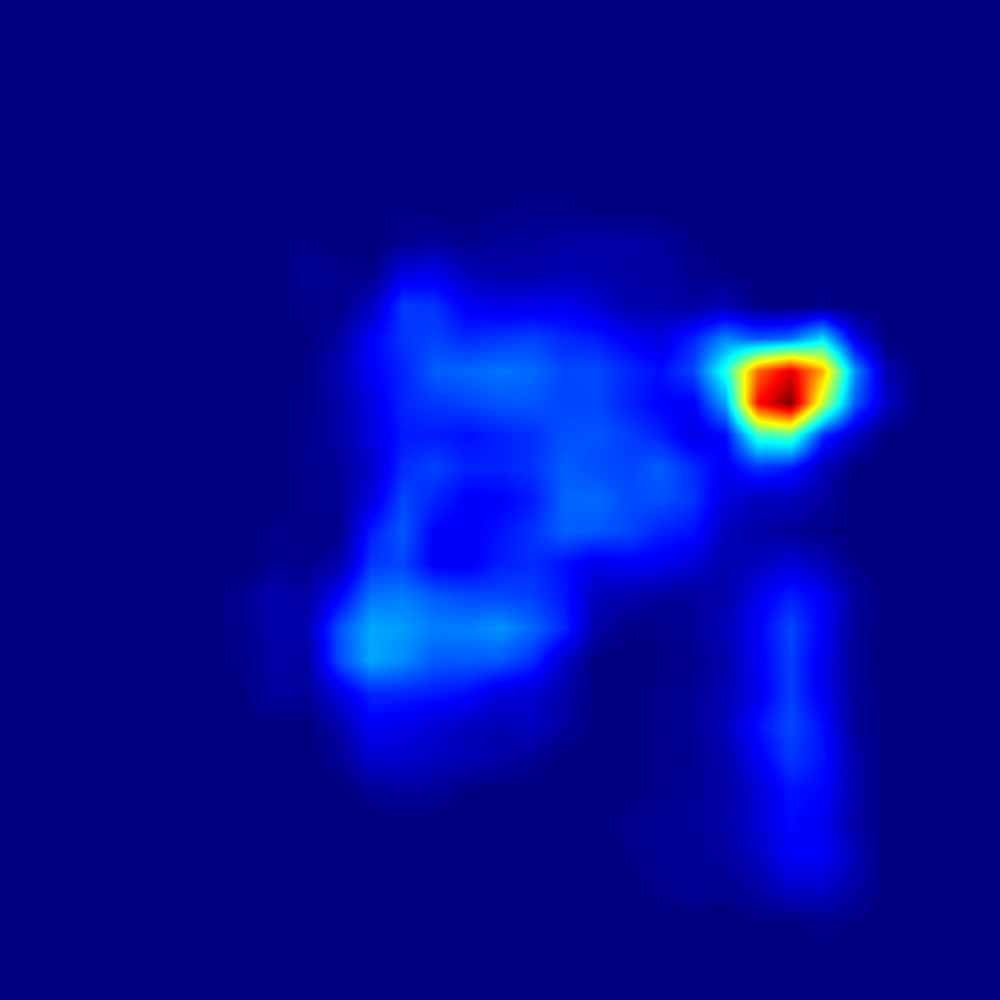

Supplement: Supplementary file 3 — Source Data File [file 41746_2022_681_MOESM3_ESM.zip › ARDA Map/Figure 1c/36.png]

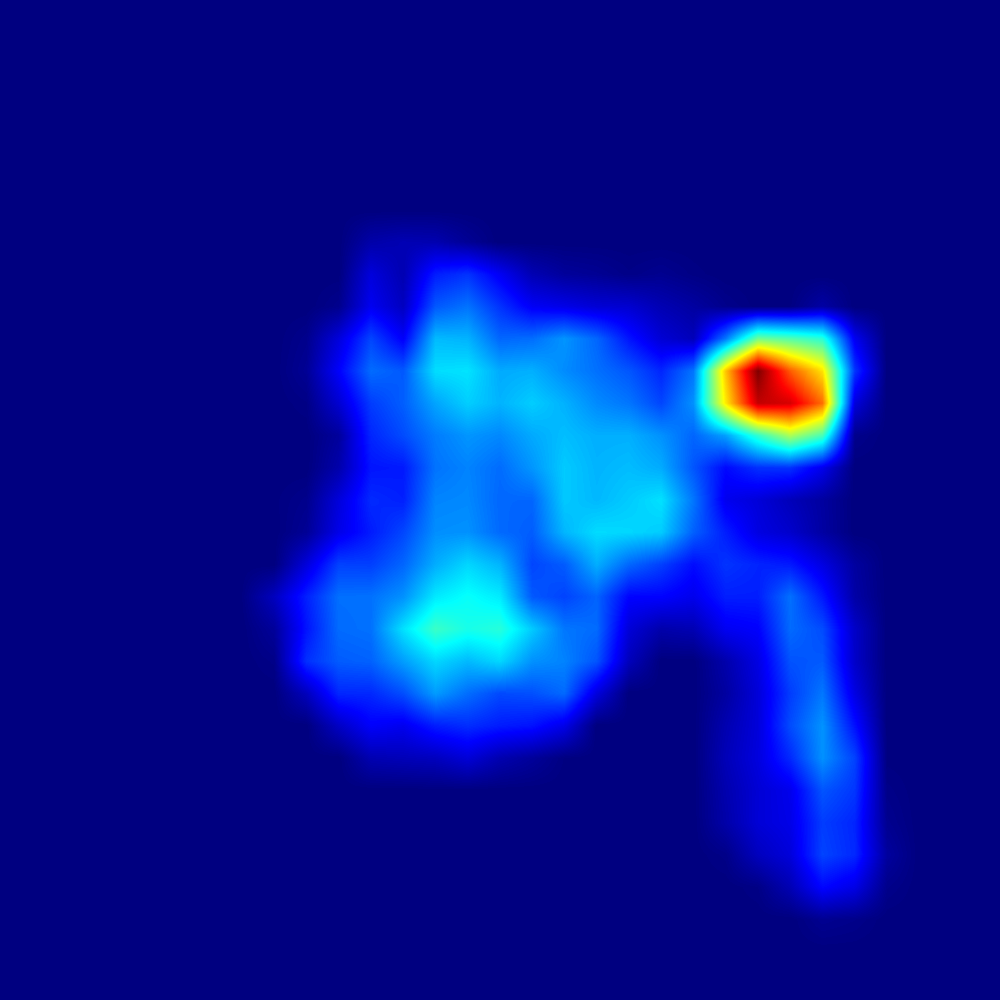

Supplement: Supplementary file 3 — Source Data File [file 41746_2022_681_MOESM3_ESM.zip › ARDA Map/Figure 1c/37.png]

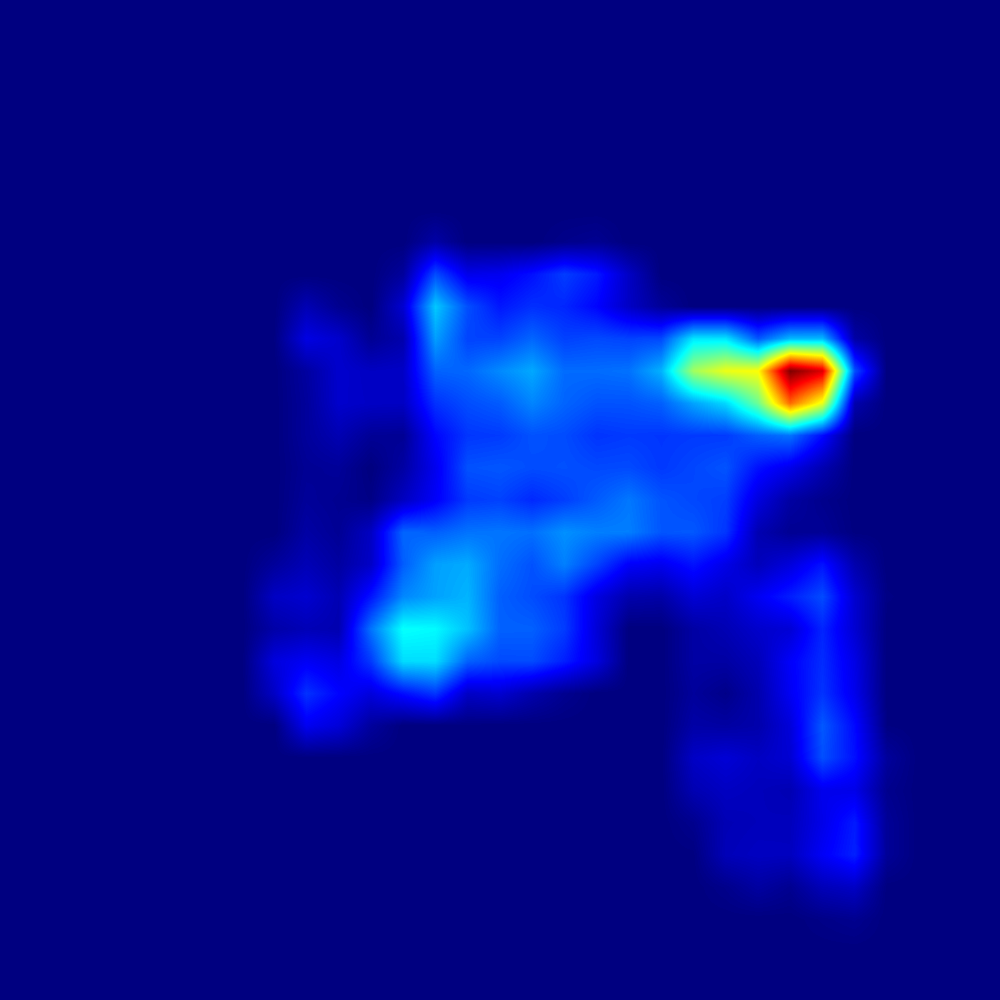

Supplement: Supplementary file 3 — Source Data File [file 41746_2022_681_MOESM3_ESM.zip › ARDA Map/Figure 1c/38.png]

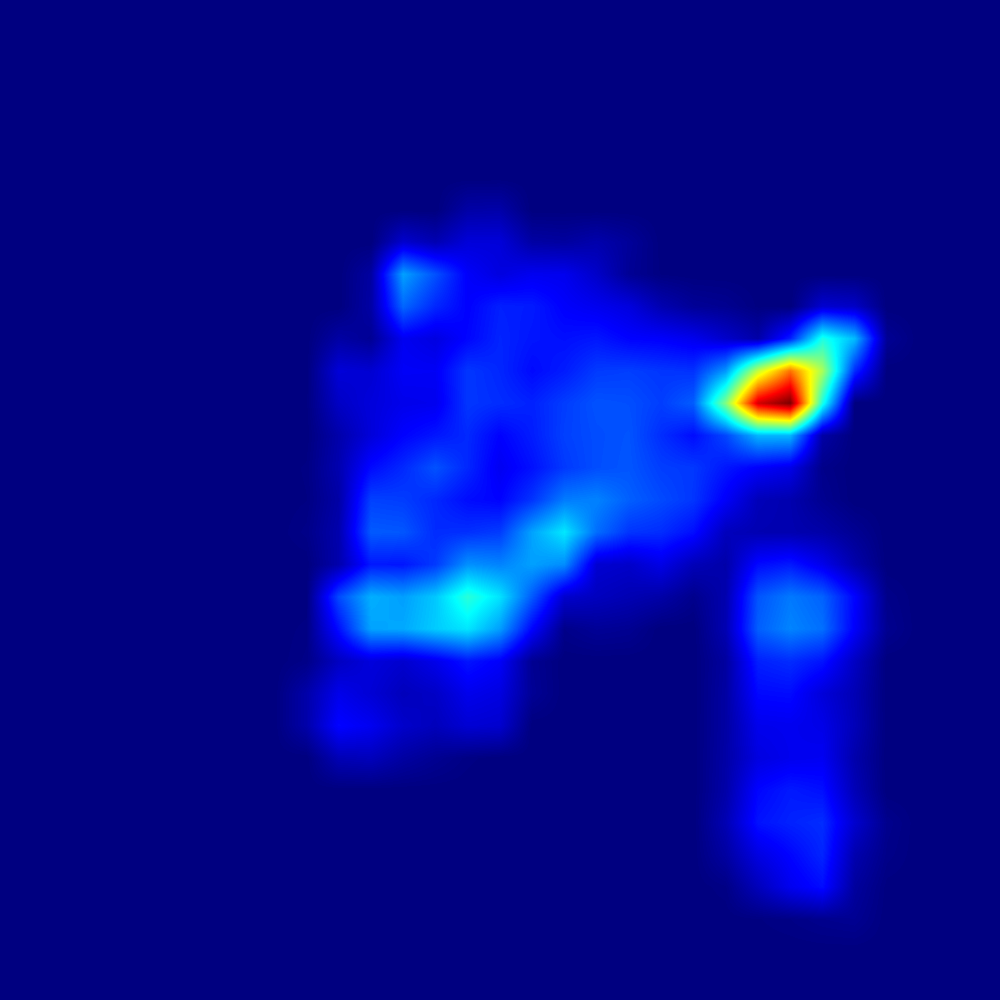

Supplement: Supplementary file 3 — Source Data File [file 41746_2022_681_MOESM3_ESM.zip › ARDA Map/Figure 1c/39.png]

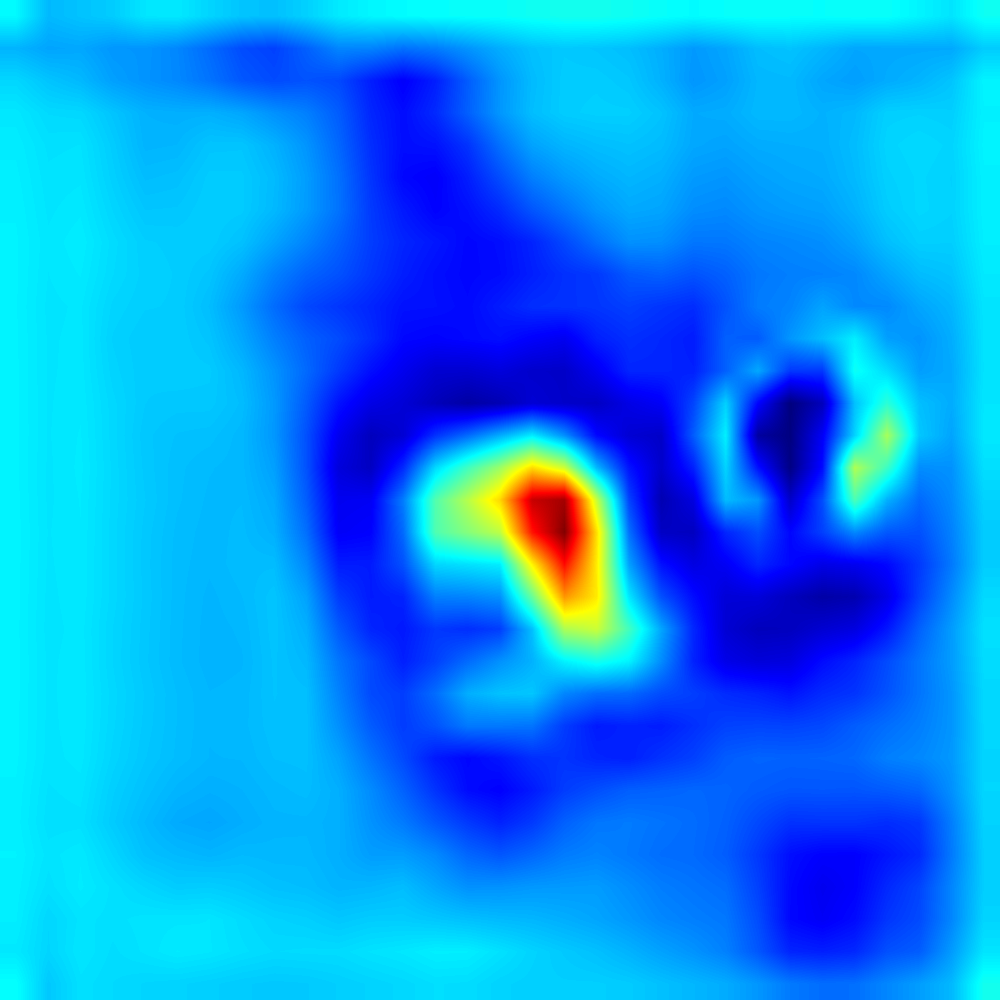

Supplement: Supplementary file 3 — Source Data File [file 41746_2022_681_MOESM3_ESM.zip › ARDA Map/Figure 1c/4.png]

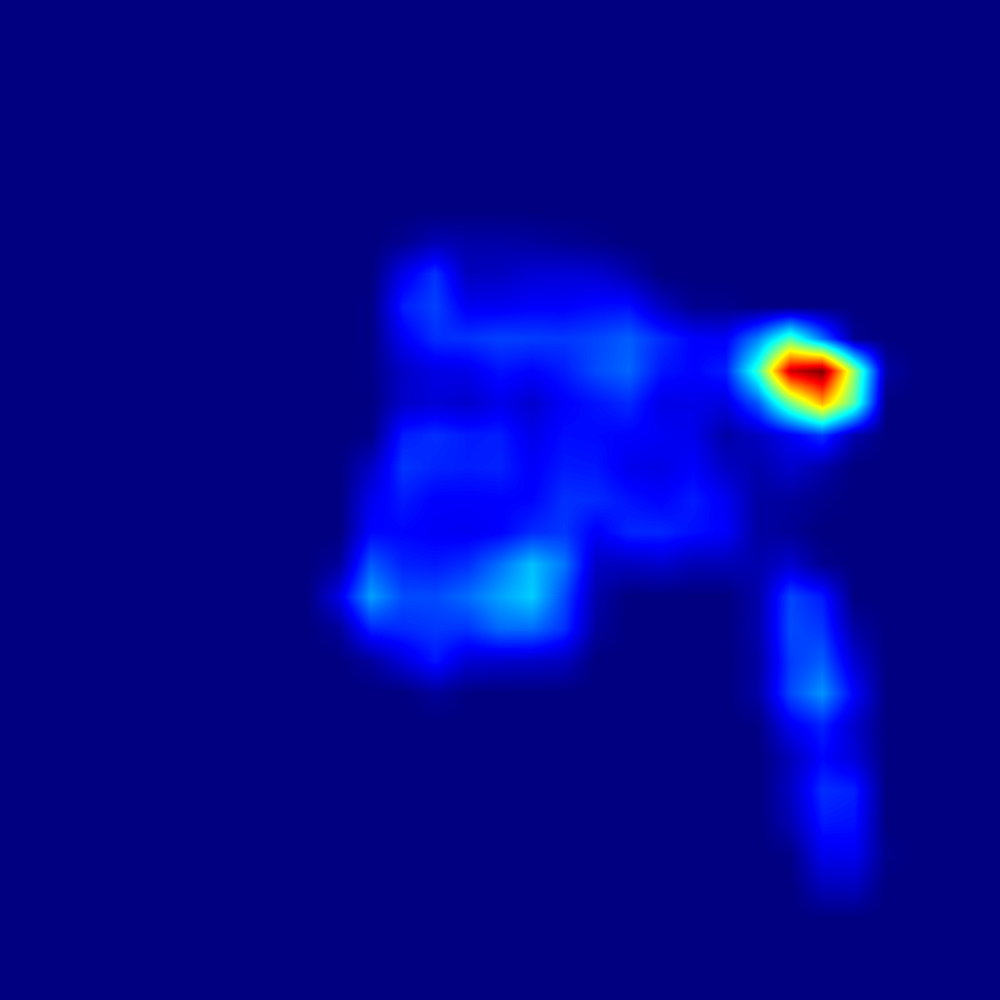

Supplement: Supplementary file 3 — Source Data File [file 41746_2022_681_MOESM3_ESM.zip › ARDA Map/Figure 1c/40.png]

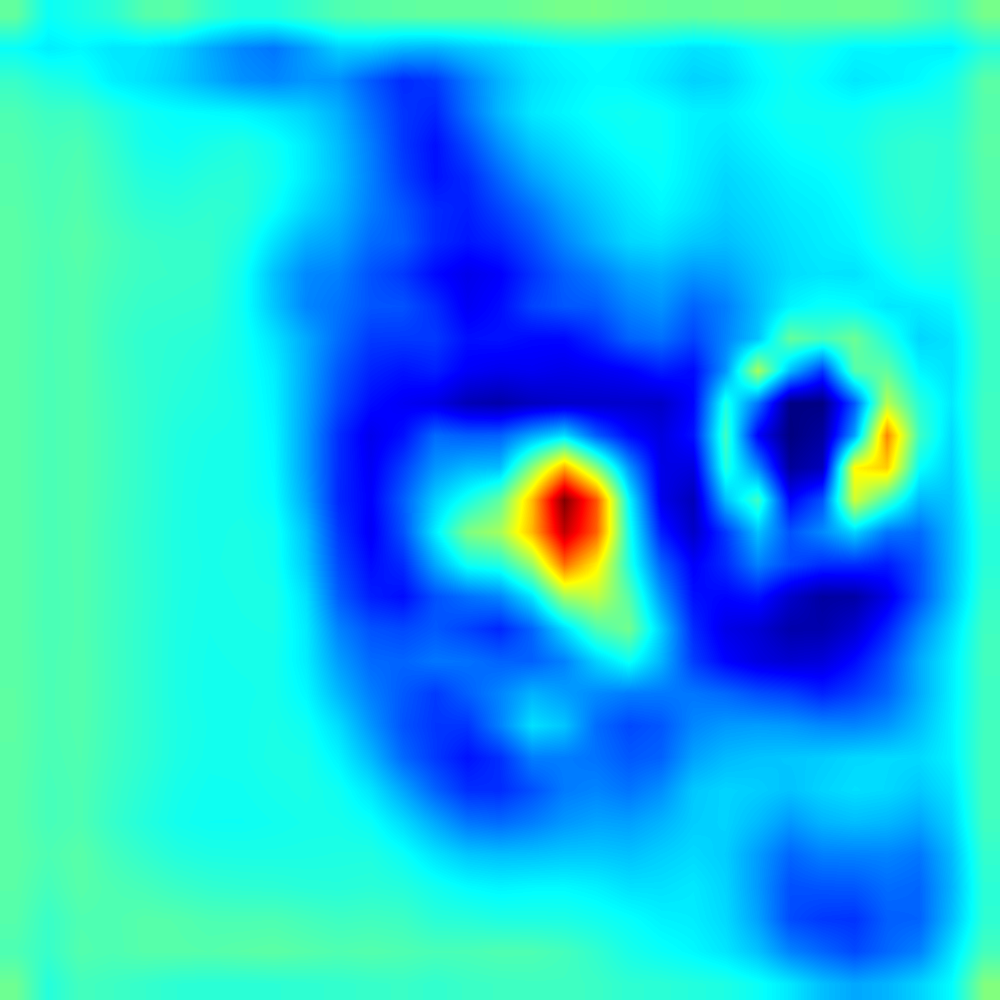

Supplement: Supplementary file 3 — Source Data File [file 41746_2022_681_MOESM3_ESM.zip › ARDA Map/Figure 1c/5.png]

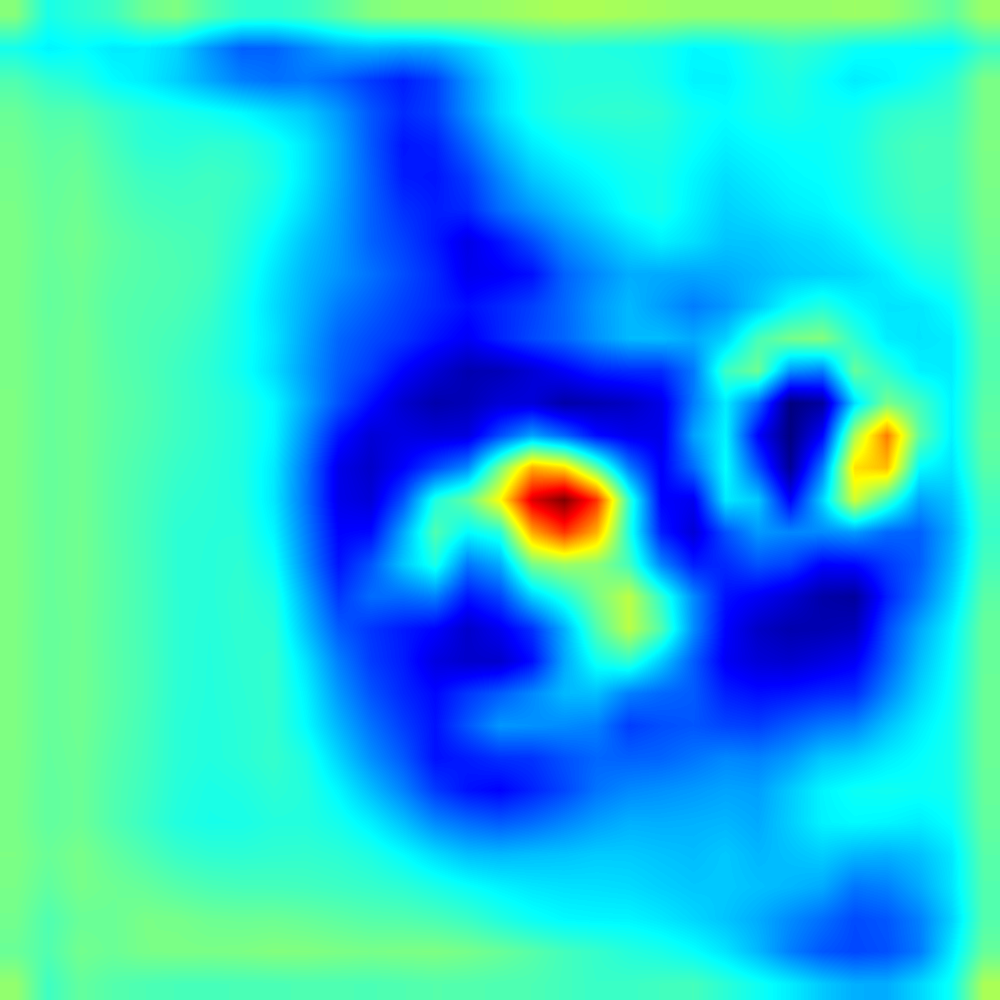

Supplement: Supplementary file 3 — Source Data File [file 41746_2022_681_MOESM3_ESM.zip › ARDA Map/Figure 1c/6.png]

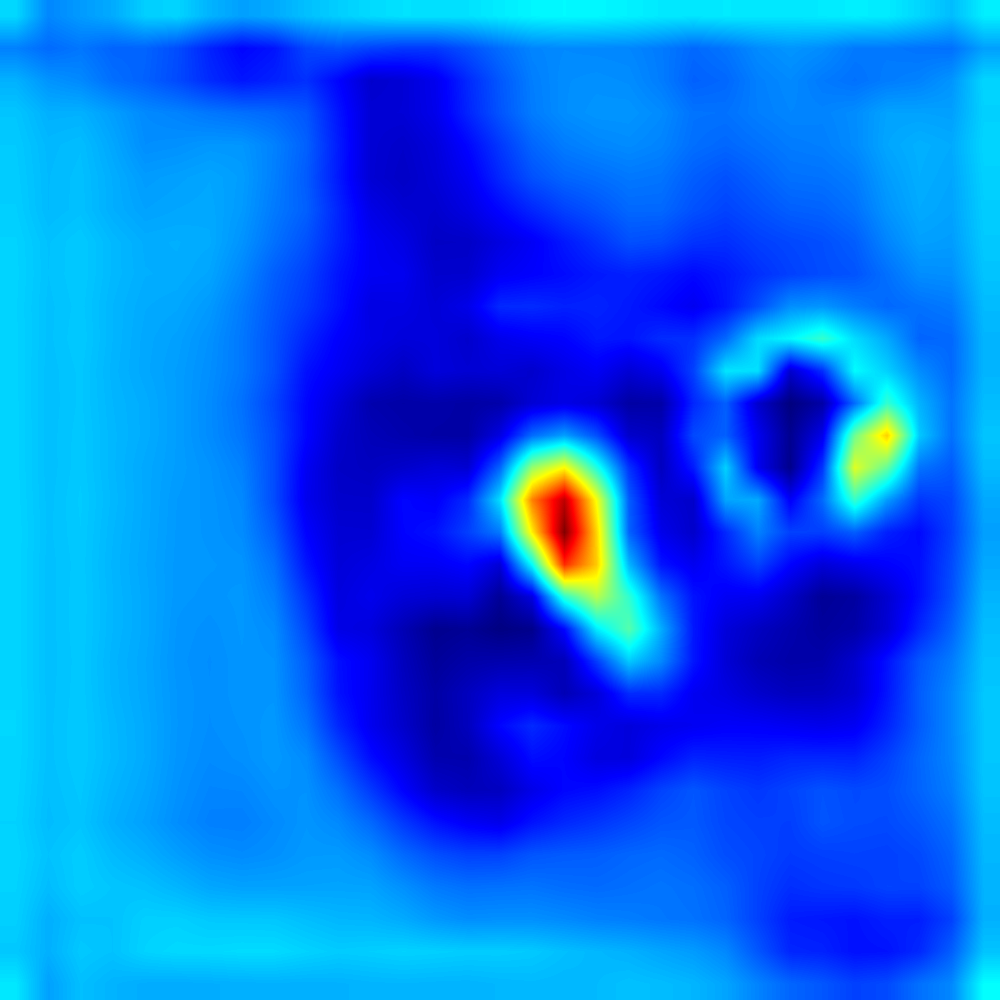

Supplement: Supplementary file 3 — Source Data File [file 41746_2022_681_MOESM3_ESM.zip › ARDA Map/Figure 1c/7.png]

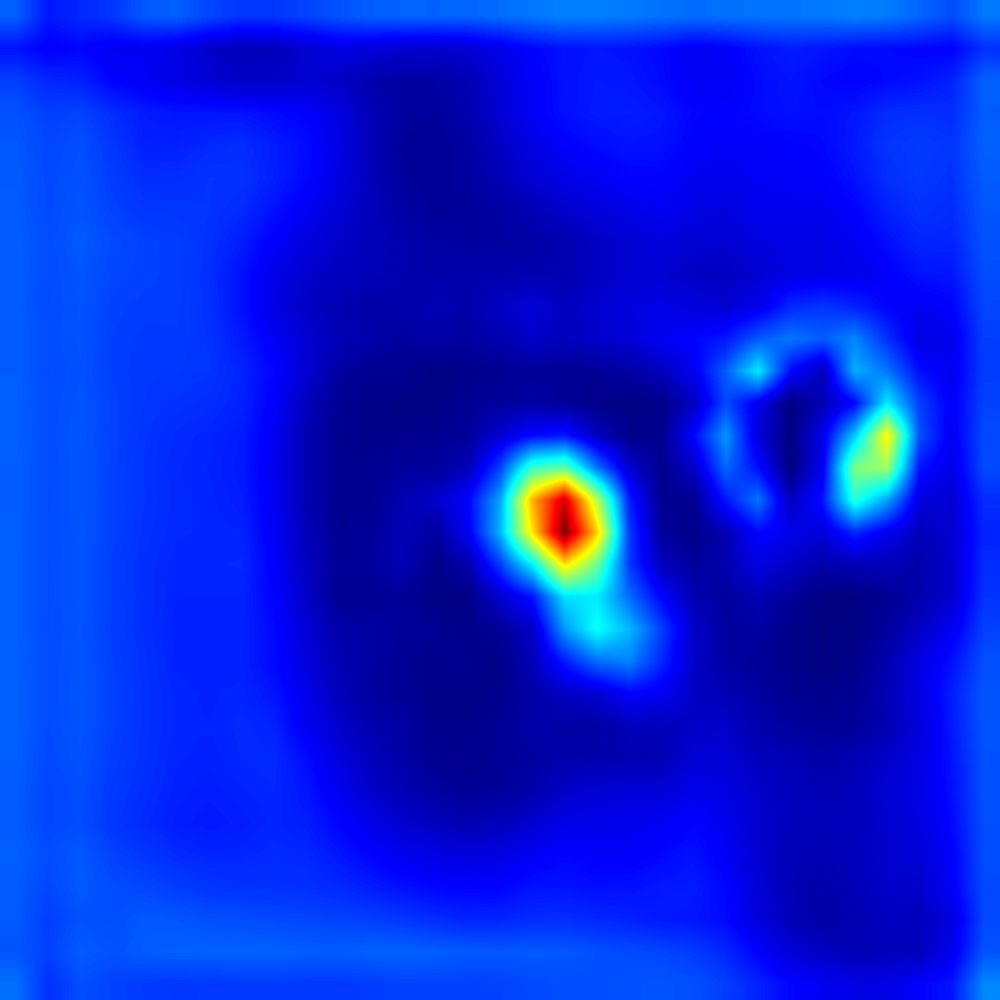

Supplement: Supplementary file 3 — Source Data File [file 41746_2022_681_MOESM3_ESM.zip › ARDA Map/Figure 1c/8.png]

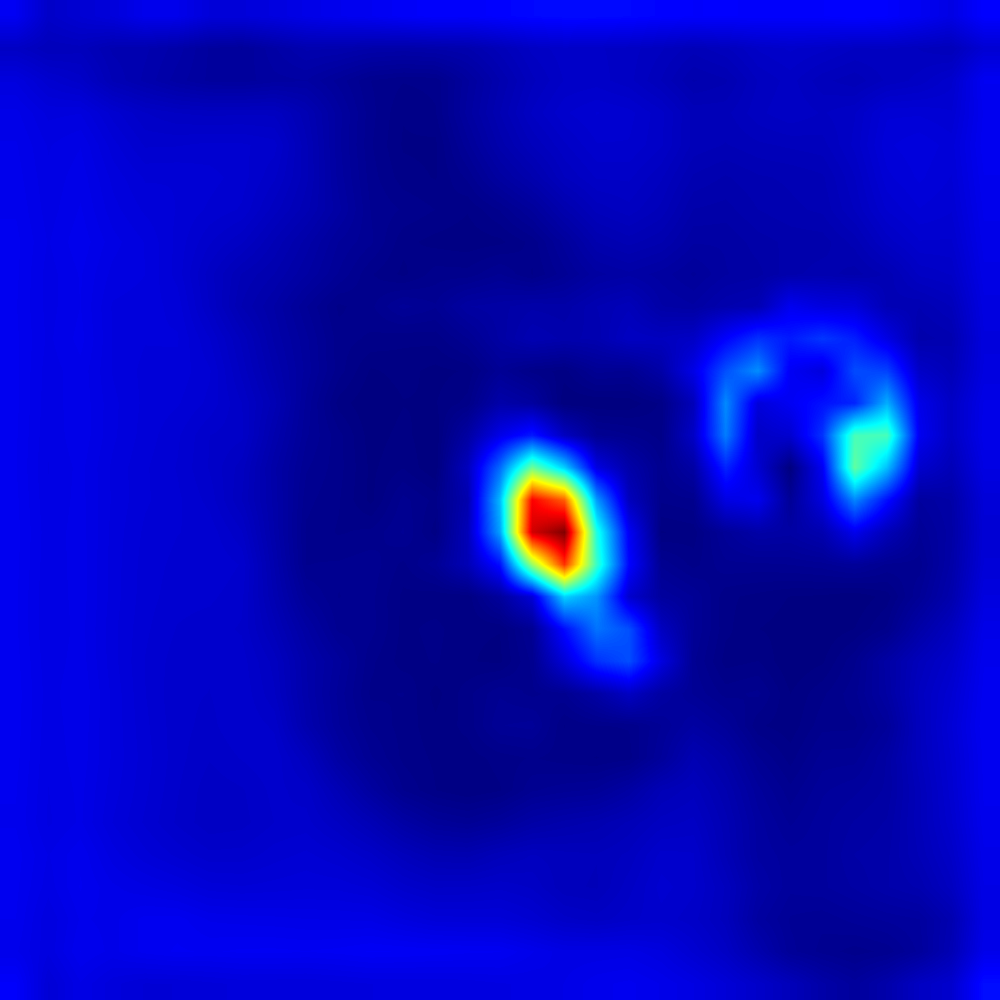

Supplement: Supplementary file 3 — Source Data File [file 41746_2022_681_MOESM3_ESM.zip › ARDA Map/Figure 1c/9.png]

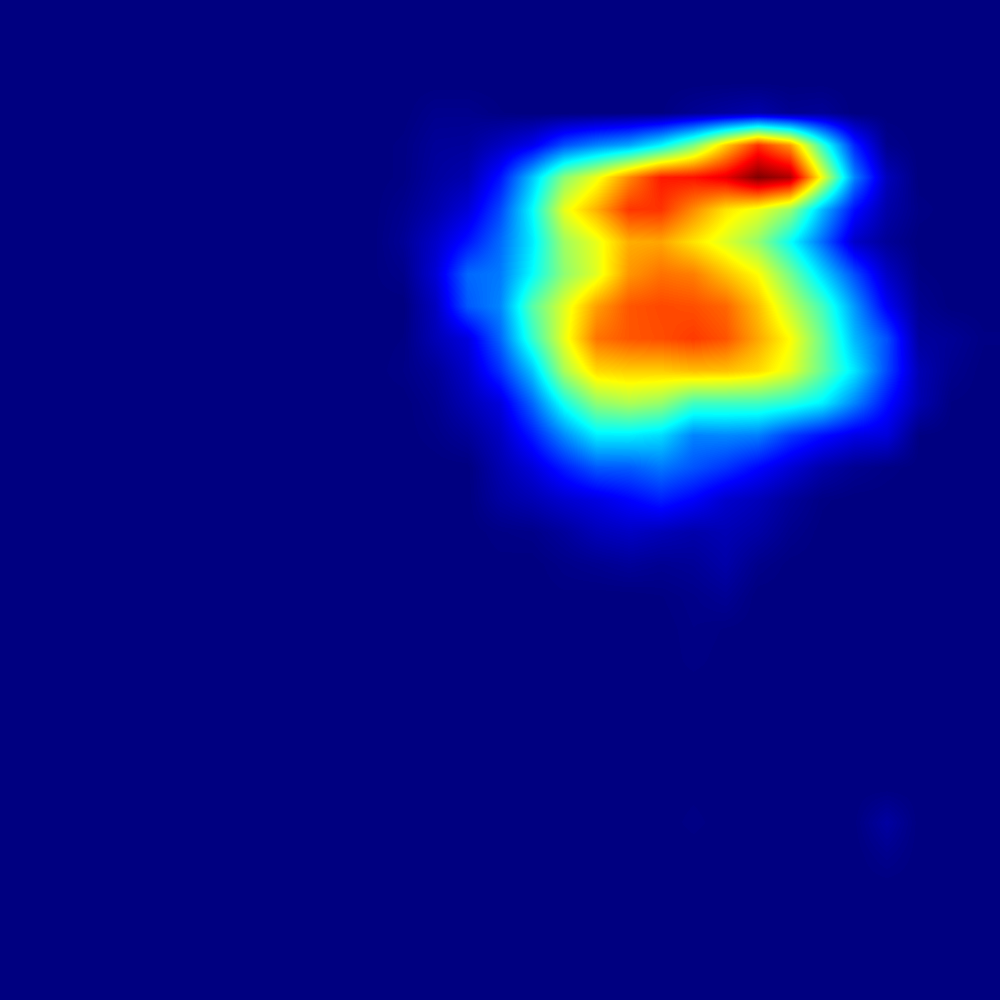

Supplement: Supplementary file 3 — Source Data File [file 41746_2022_681_MOESM3_ESM.zip › ARDA Map/Figure 3/10.png]

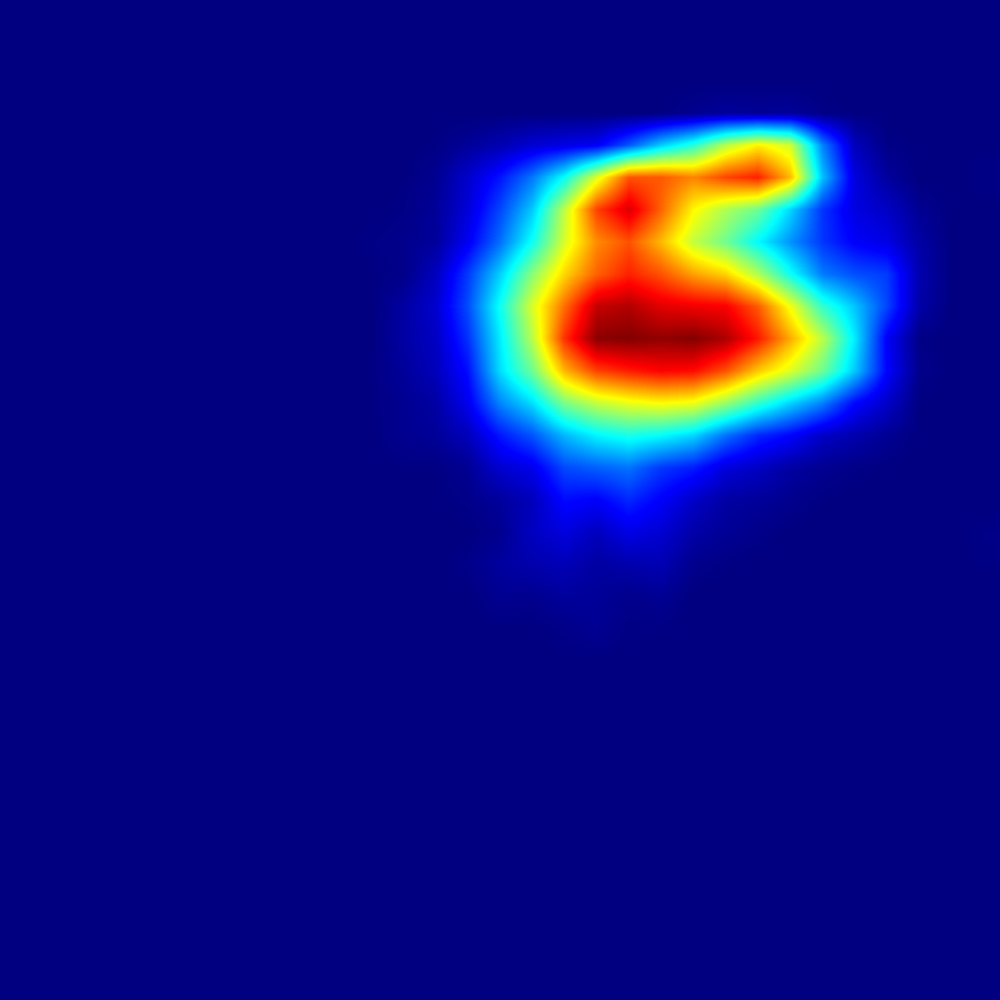

Supplement: Supplementary file 3 — Source Data File [file 41746_2022_681_MOESM3_ESM.zip › ARDA Map/Figure 3/11.png]

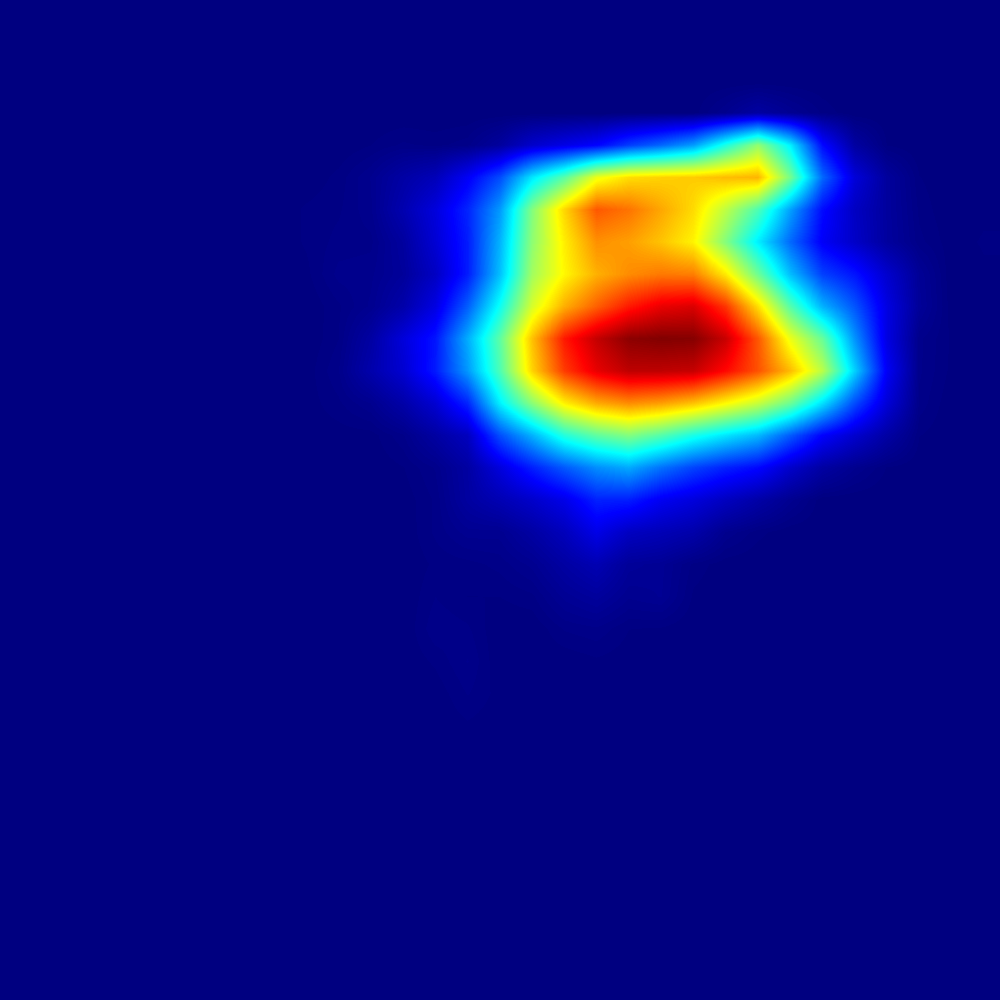

Supplement: Supplementary file 3 — Source Data File [file 41746_2022_681_MOESM3_ESM.zip › ARDA Map/Figure 3/12.png]

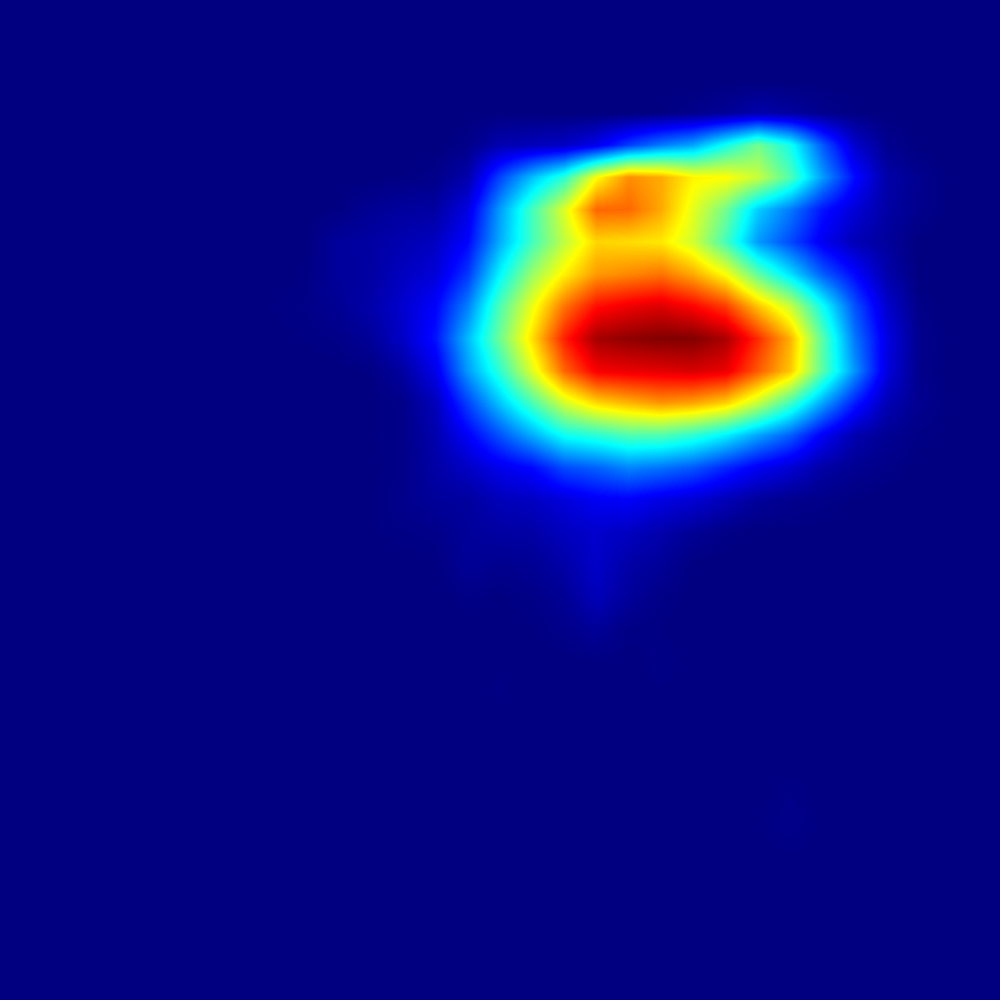

Supplement: Supplementary file 3 — Source Data File [file 41746_2022_681_MOESM3_ESM.zip › ARDA Map/Figure 3/13.png]

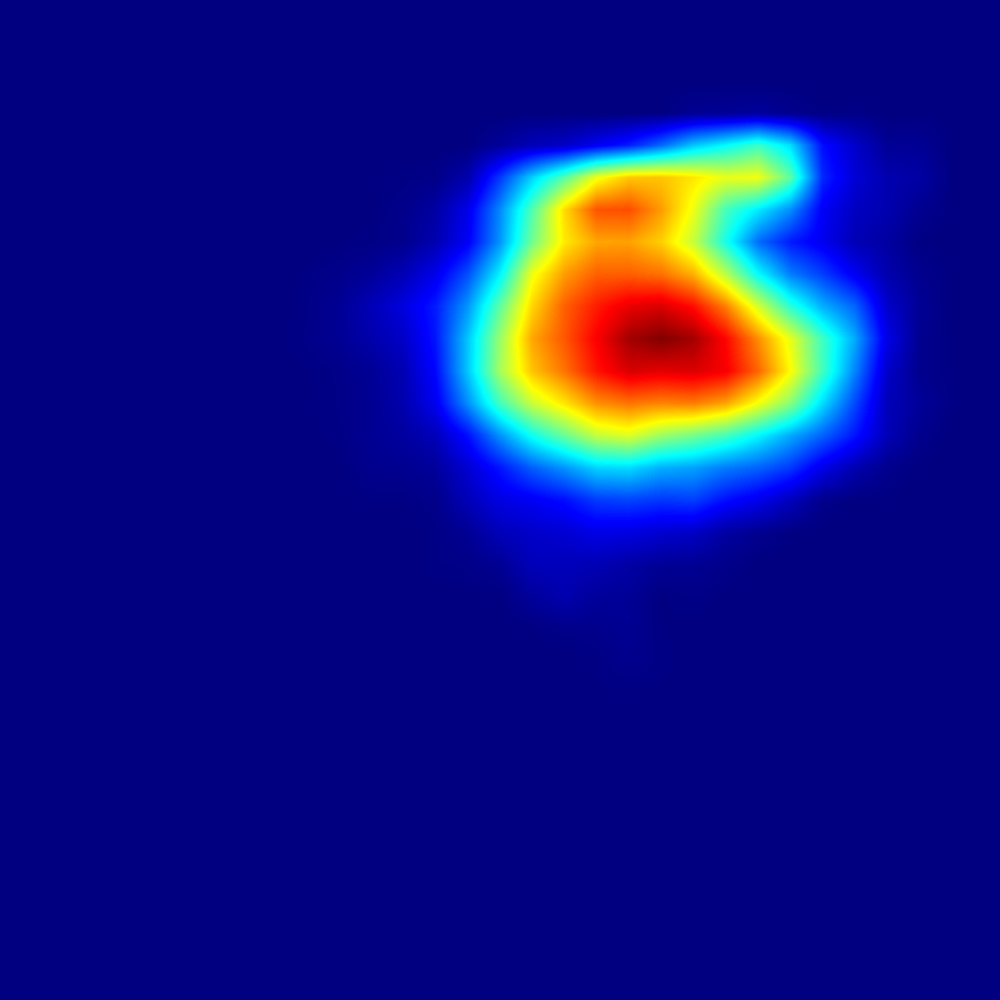

Supplement: Supplementary file 3 — Source Data File [file 41746_2022_681_MOESM3_ESM.zip › ARDA Map/Figure 3/14.png]

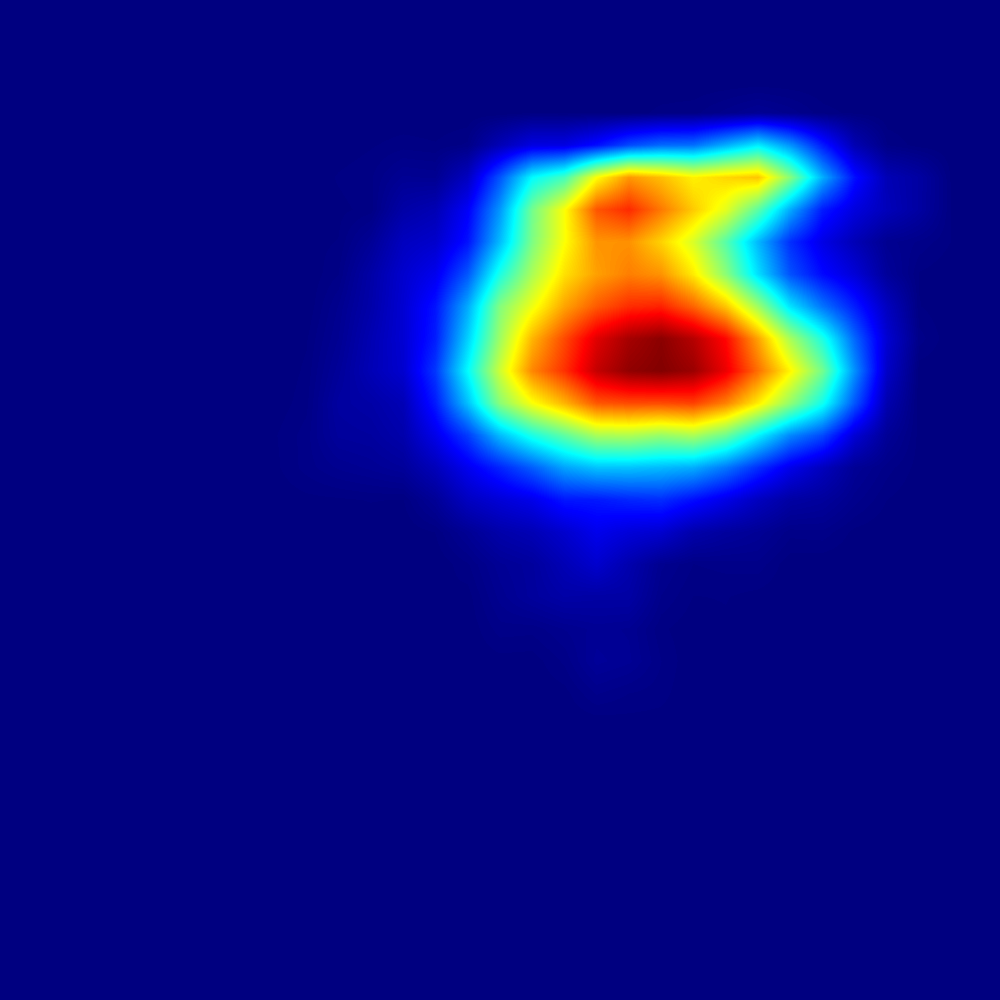

Supplement: Supplementary file 3 — Source Data File [file 41746_2022_681_MOESM3_ESM.zip › ARDA Map/Figure 3/15.png]

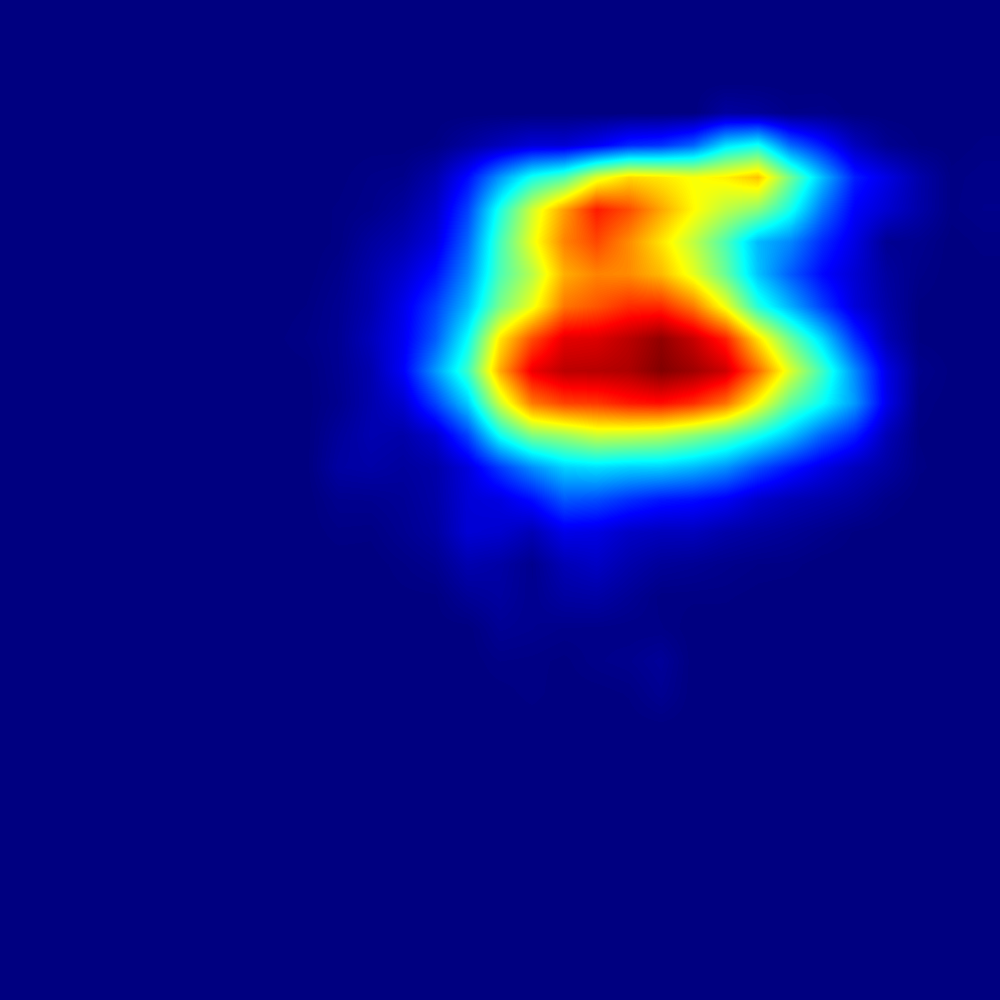

Supplement: Supplementary file 3 — Source Data File [file 41746_2022_681_MOESM3_ESM.zip › ARDA Map/Figure 3/16.png]

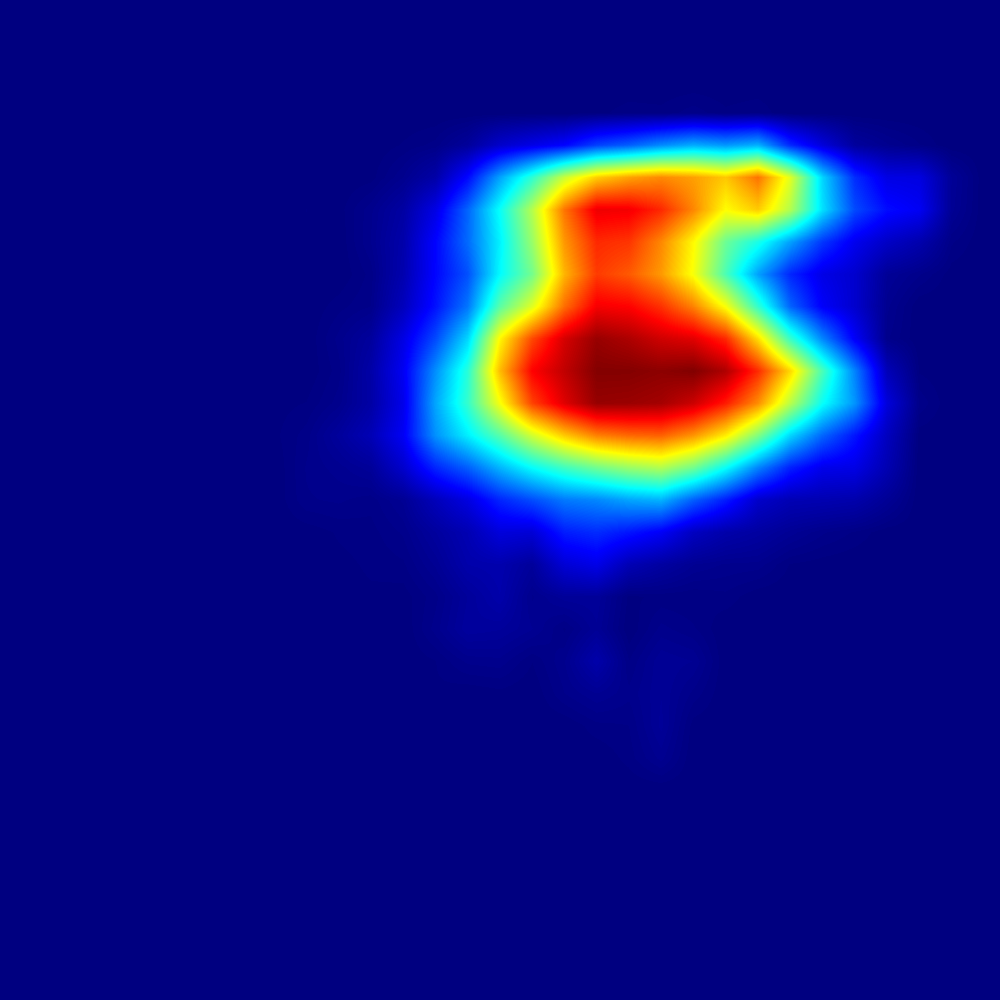

Supplement: Supplementary file 3 — Source Data File [file 41746_2022_681_MOESM3_ESM.zip › ARDA Map/Figure 3/17.png]

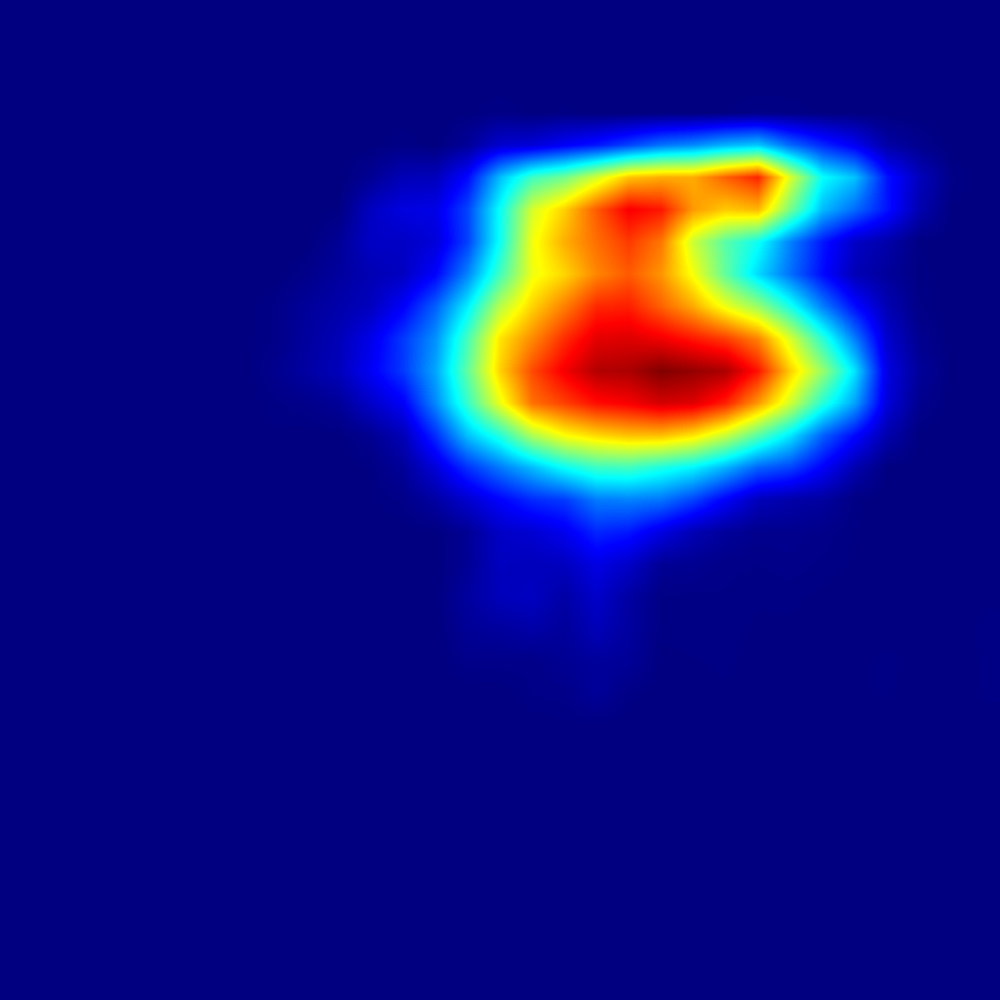

Supplement: Supplementary file 3 — Source Data File [file 41746_2022_681_MOESM3_ESM.zip › ARDA Map/Figure 3/18.png]

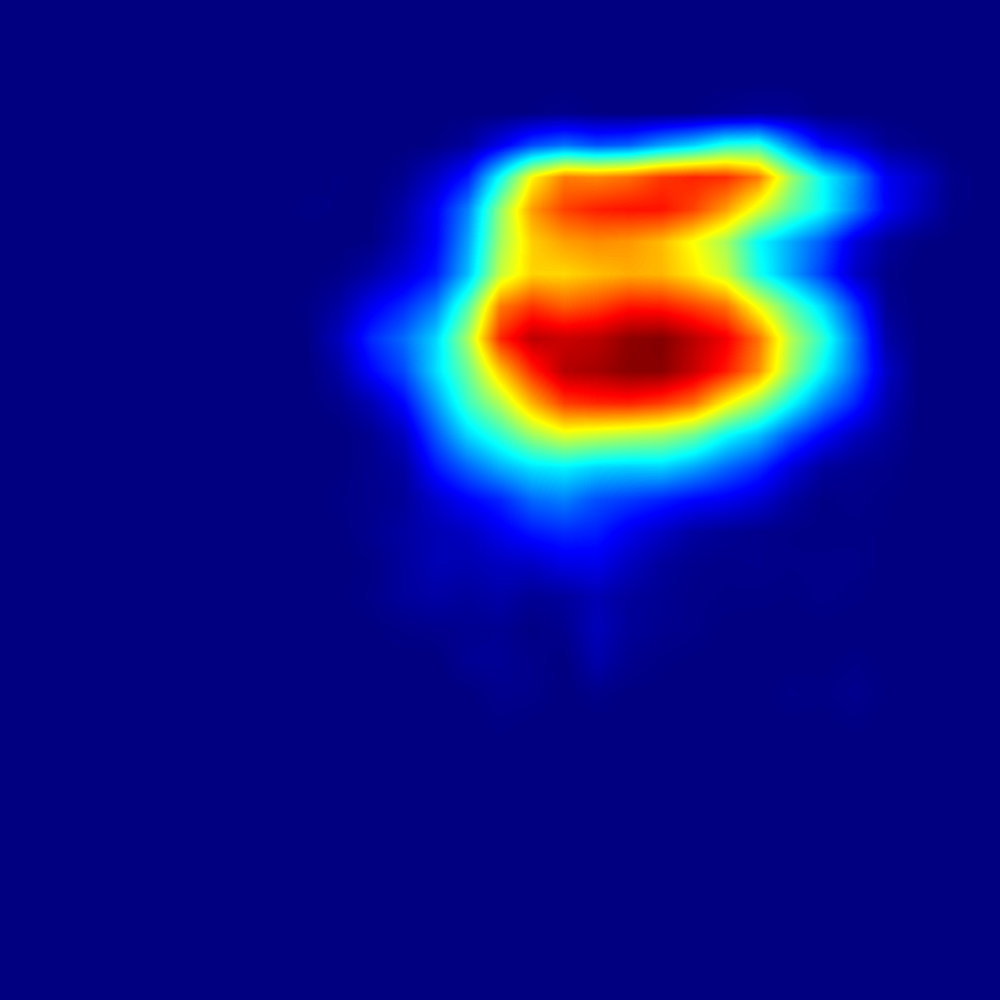

Supplement: Supplementary file 3 — Source Data File [file 41746_2022_681_MOESM3_ESM.zip › ARDA Map/Figure 3/19.png]

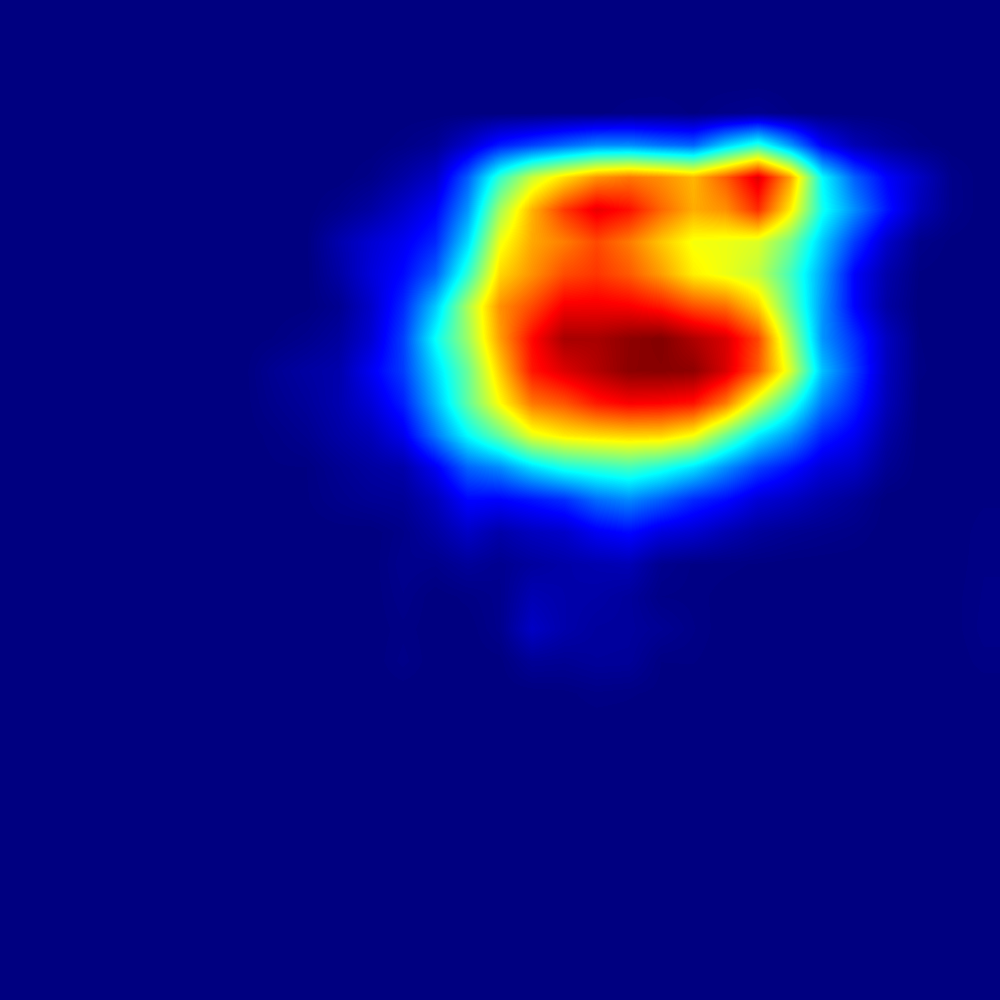

Supplement: Supplementary file 3 — Source Data File [file 41746_2022_681_MOESM3_ESM.zip › ARDA Map/Figure 3/20.png]

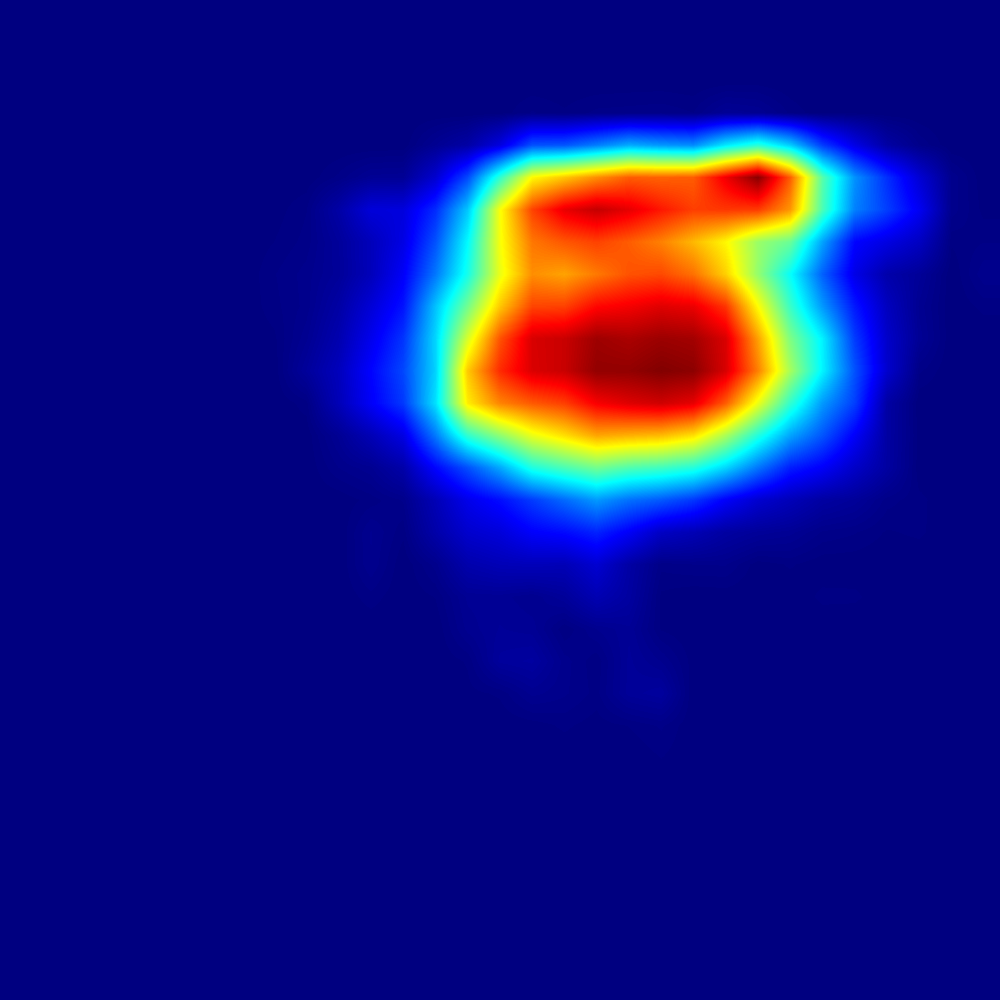

Supplement: Supplementary file 3 — Source Data File [file 41746_2022_681_MOESM3_ESM.zip › ARDA Map/Figure 3/22.png]

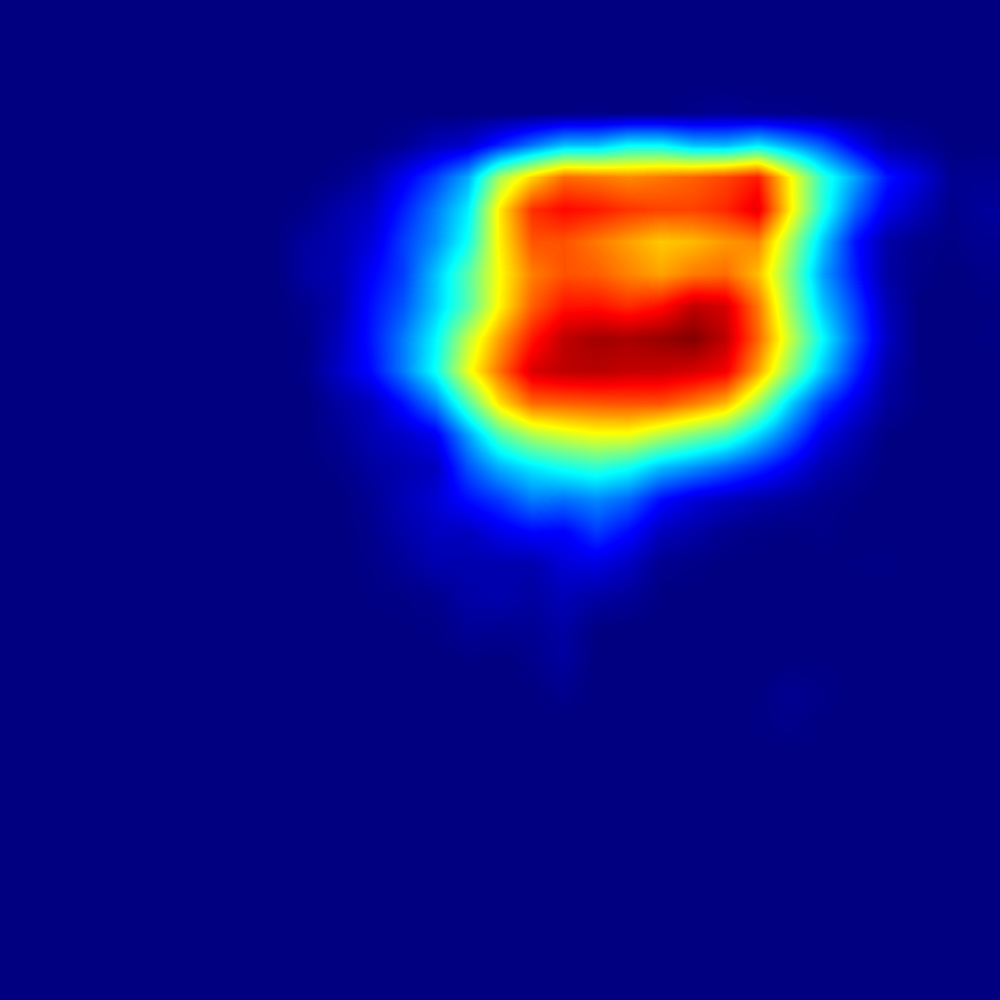

Supplement: Supplementary file 3 — Source Data File [file 41746_2022_681_MOESM3_ESM.zip › ARDA Map/Figure 3/23.png]

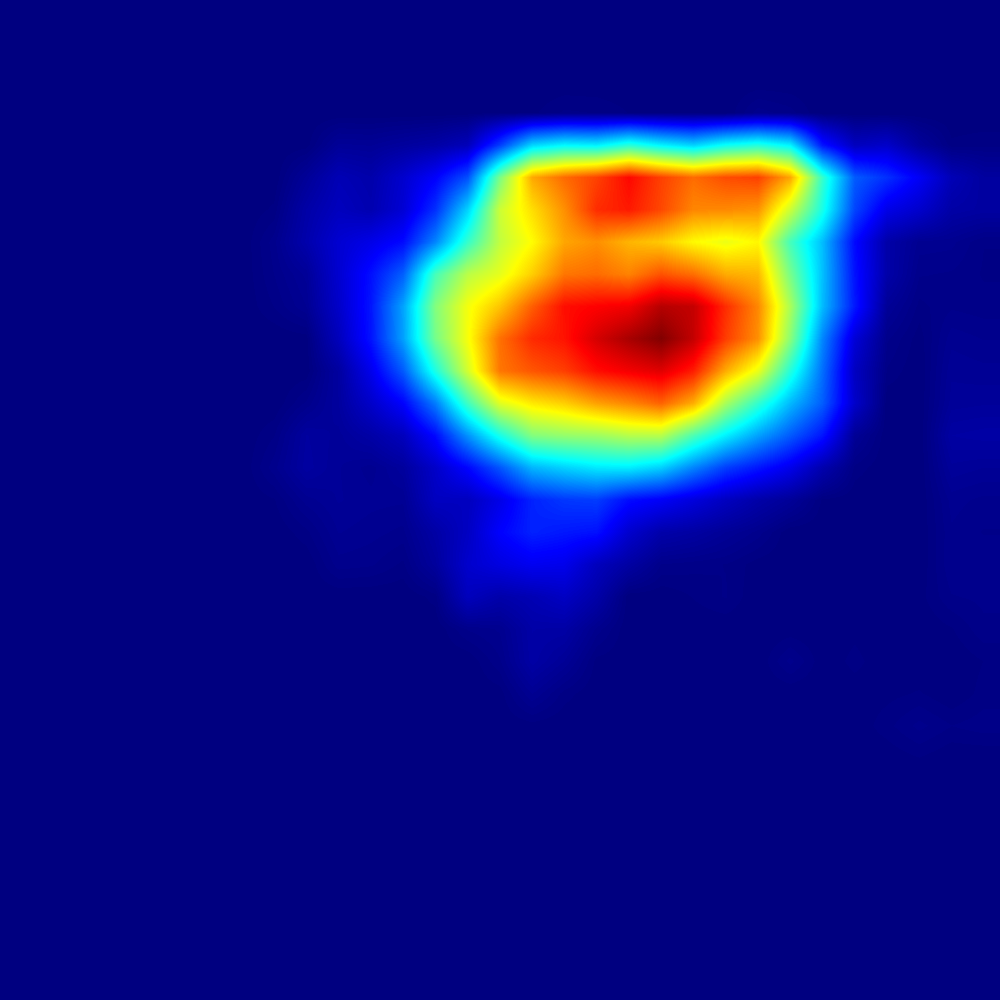

Supplement: Supplementary file 3 — Source Data File [file 41746_2022_681_MOESM3_ESM.zip › ARDA Map/Figure 3/24.png]

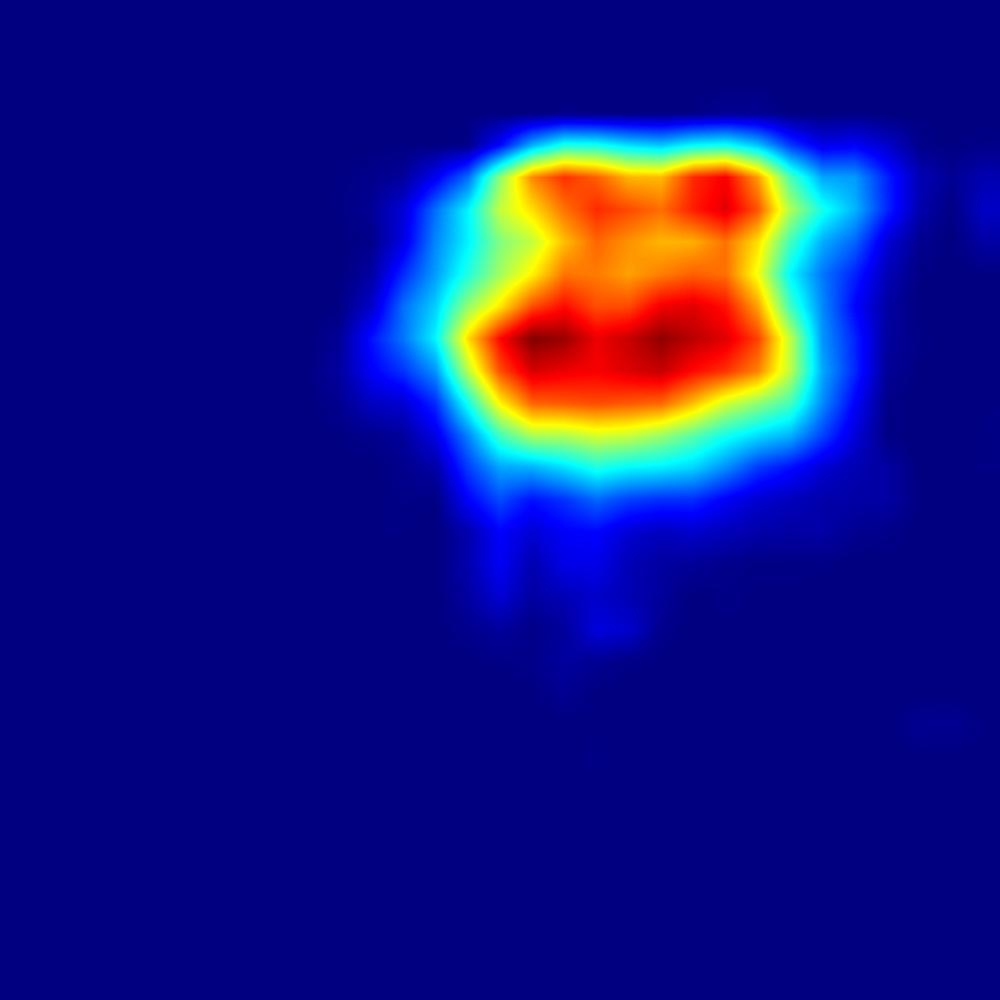

Supplement: Supplementary file 3 — Source Data File [file 41746_2022_681_MOESM3_ESM.zip › ARDA Map/Figure 3/25.png]

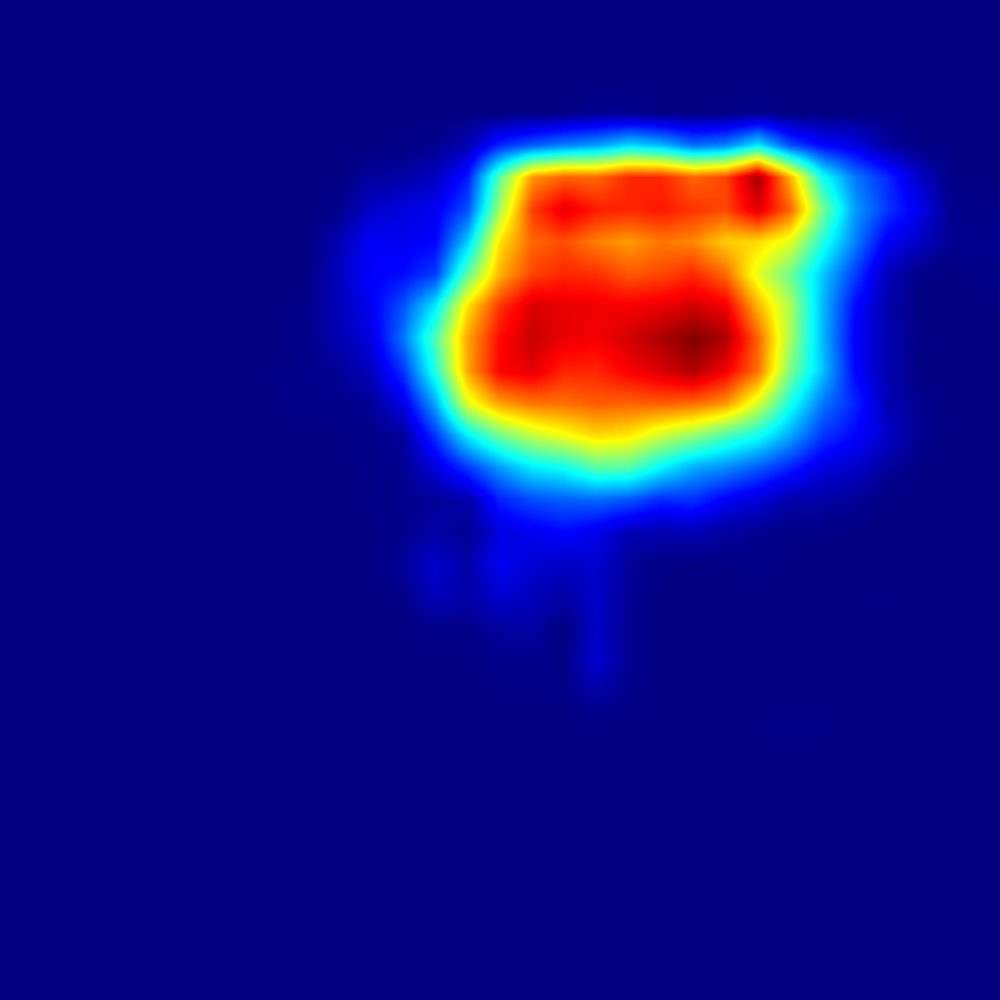

Supplement: Supplementary file 3 — Source Data File [file 41746_2022_681_MOESM3_ESM.zip › ARDA Map/Figure 3/26.png]

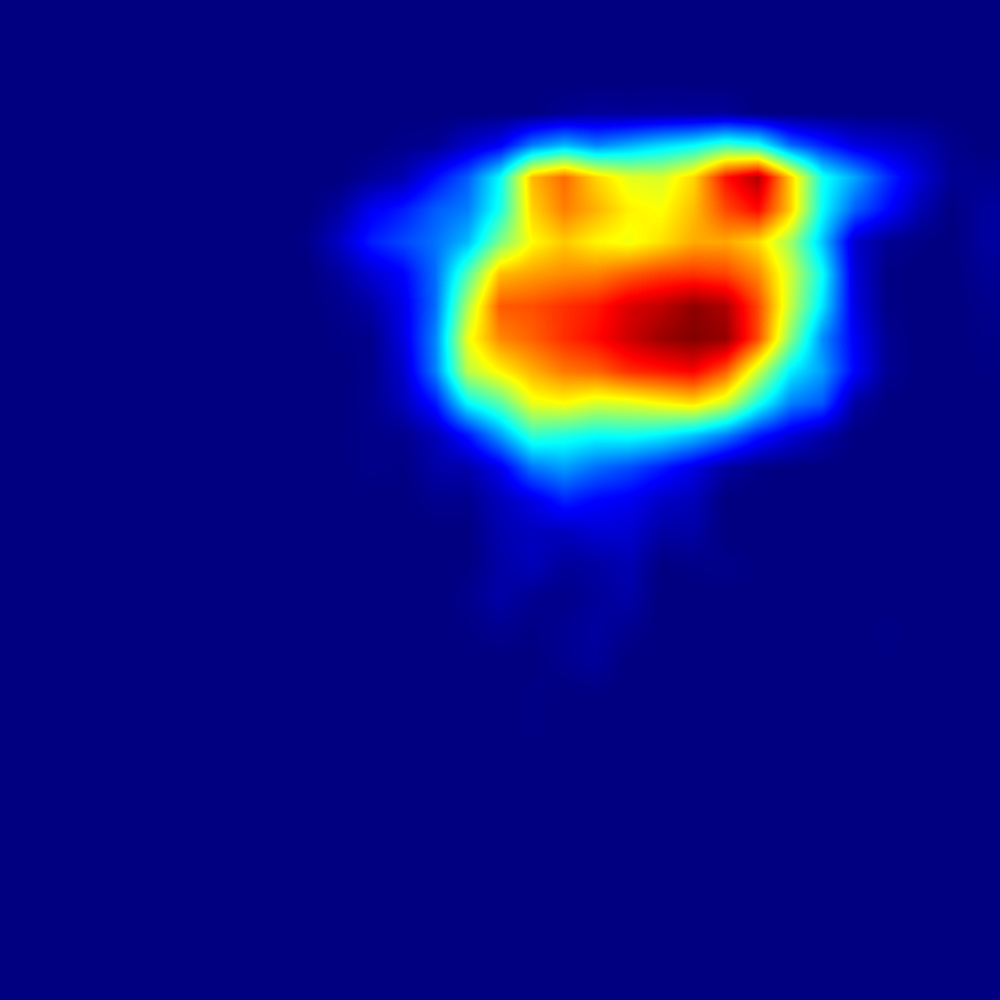

Supplement: Supplementary file 3 — Source Data File [file 41746_2022_681_MOESM3_ESM.zip › ARDA Map/Figure 3/28.png]

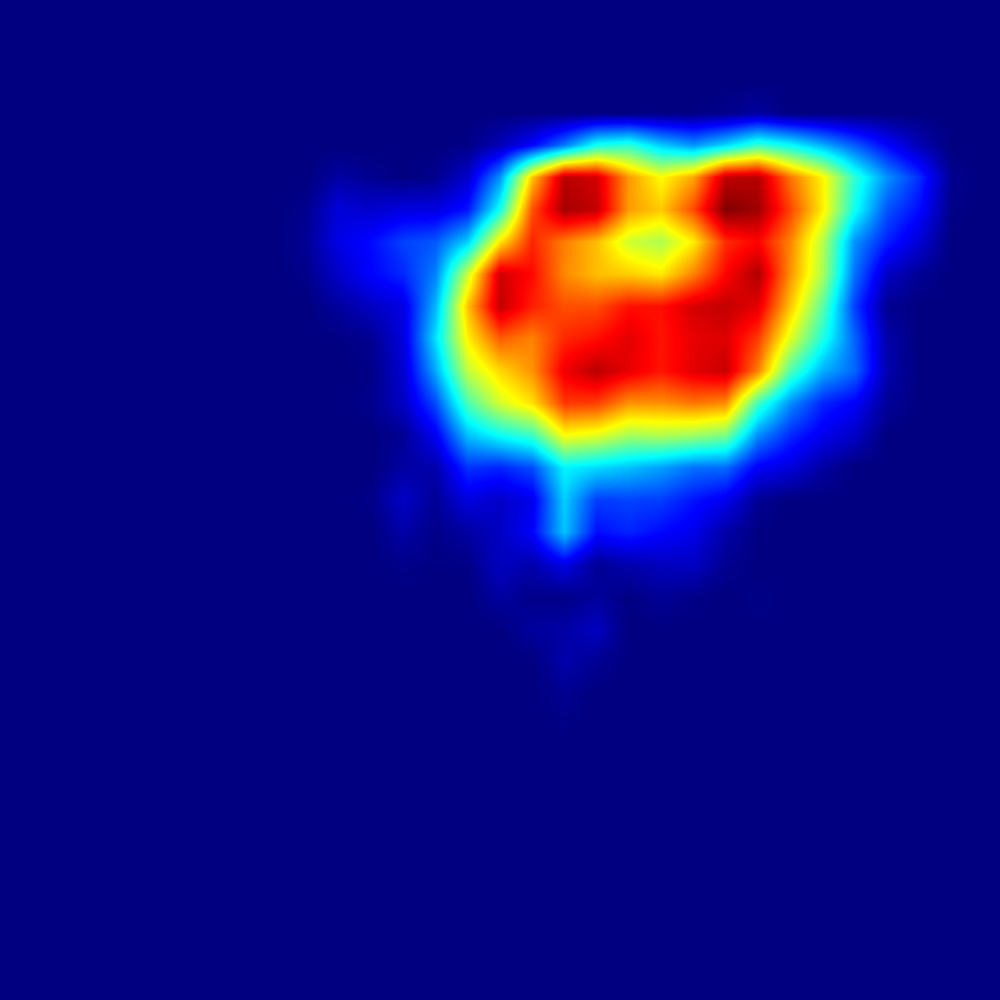

Supplement: Supplementary file 3 — Source Data File [file 41746_2022_681_MOESM3_ESM.zip › ARDA Map/Figure 3/29.png]

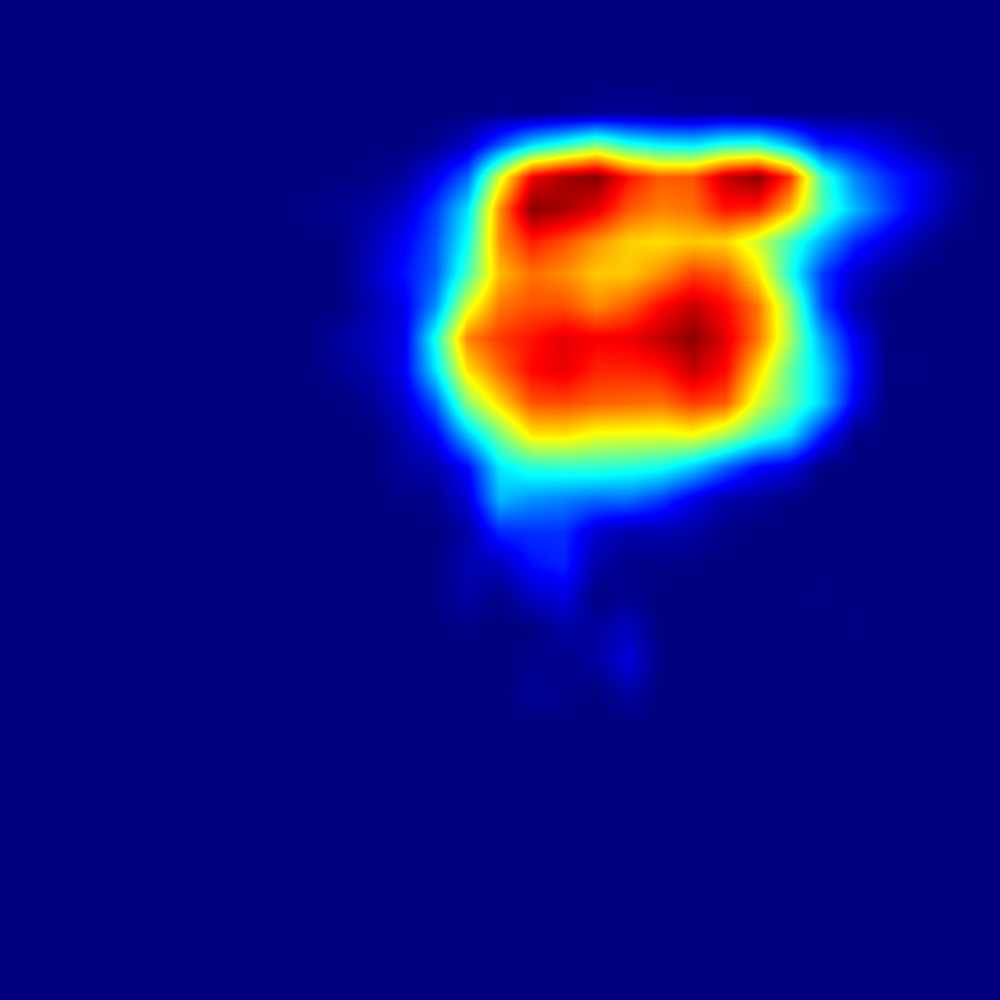

Supplement: Supplementary file 3 — Source Data File [file 41746_2022_681_MOESM3_ESM.zip › ARDA Map/Figure 3/30.png]

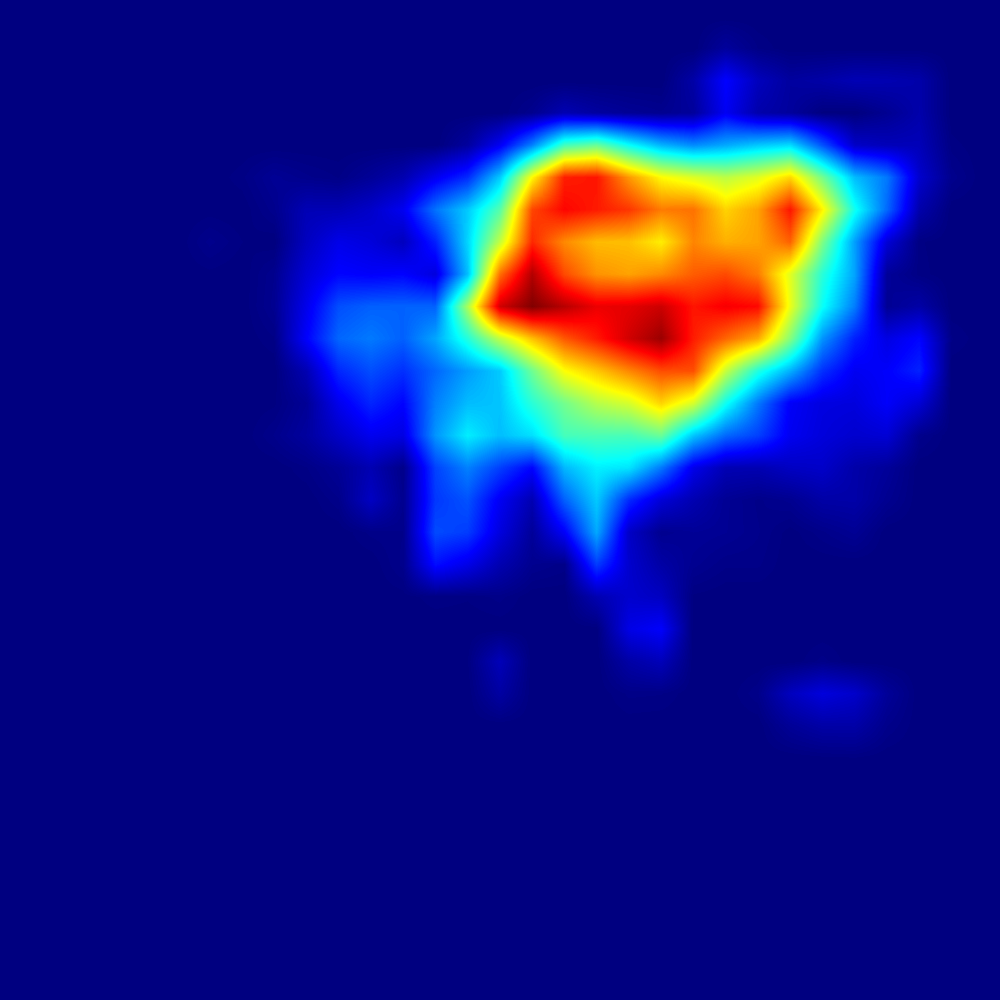

Supplement: Supplementary file 3 — Source Data File [file 41746_2022_681_MOESM3_ESM.zip › ARDA Map/Figure 3/31.png]

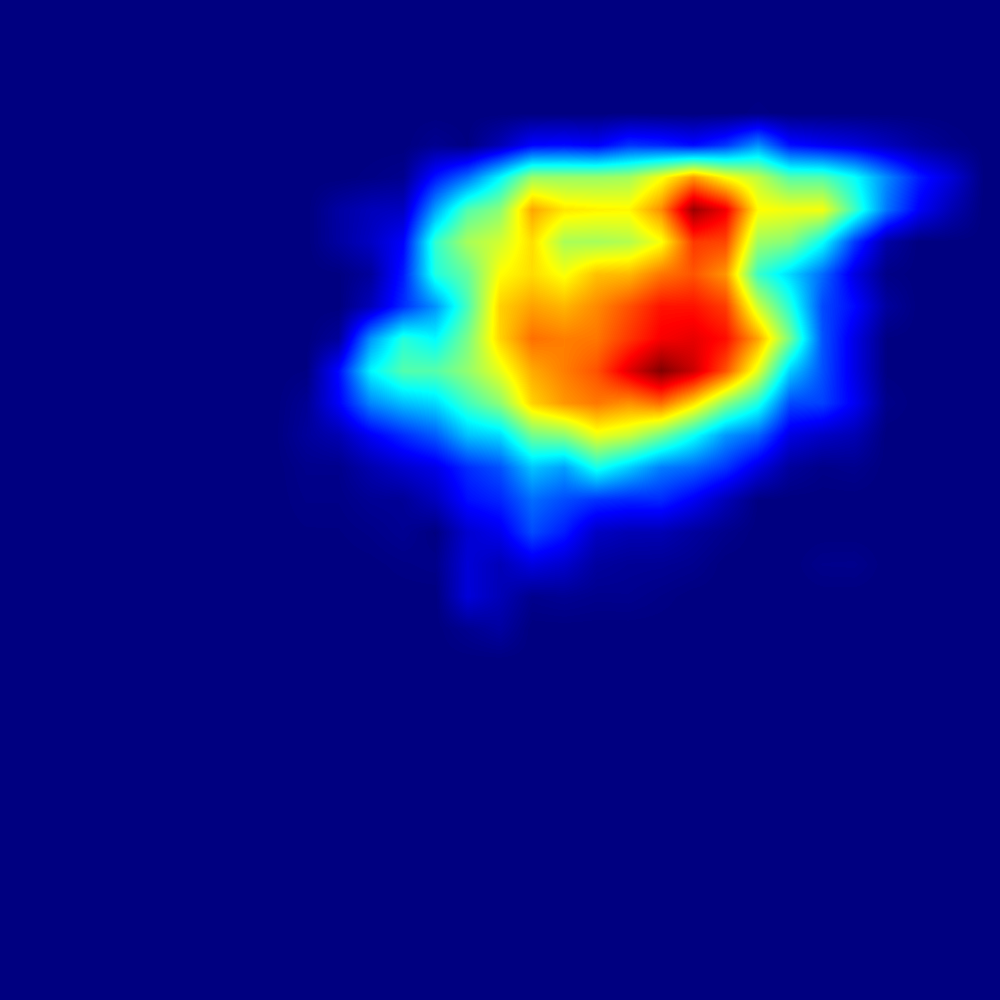

Supplement: Supplementary file 3 — Source Data File [file 41746_2022_681_MOESM3_ESM.zip › ARDA Map/Figure 3/32.png]

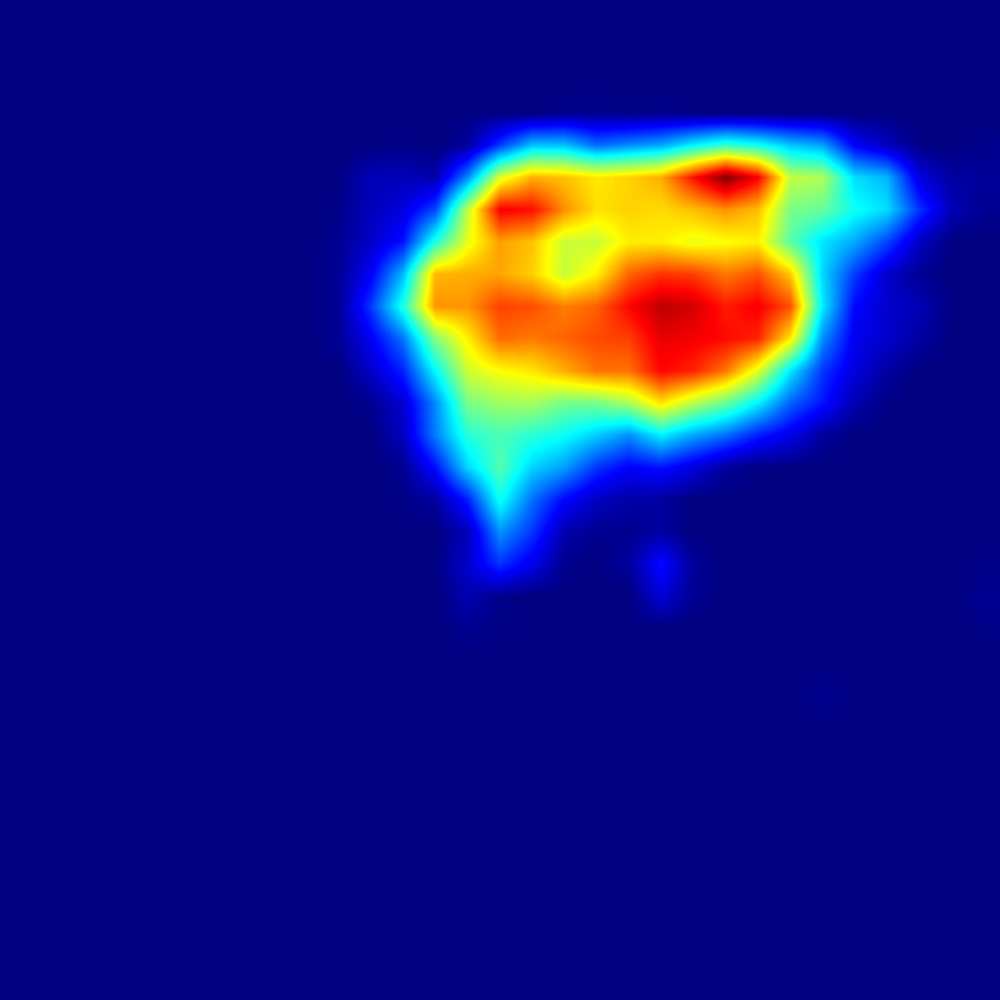

Supplement: Supplementary file 3 — Source Data File [file 41746_2022_681_MOESM3_ESM.zip › ARDA Map/Figure 3/33.png]

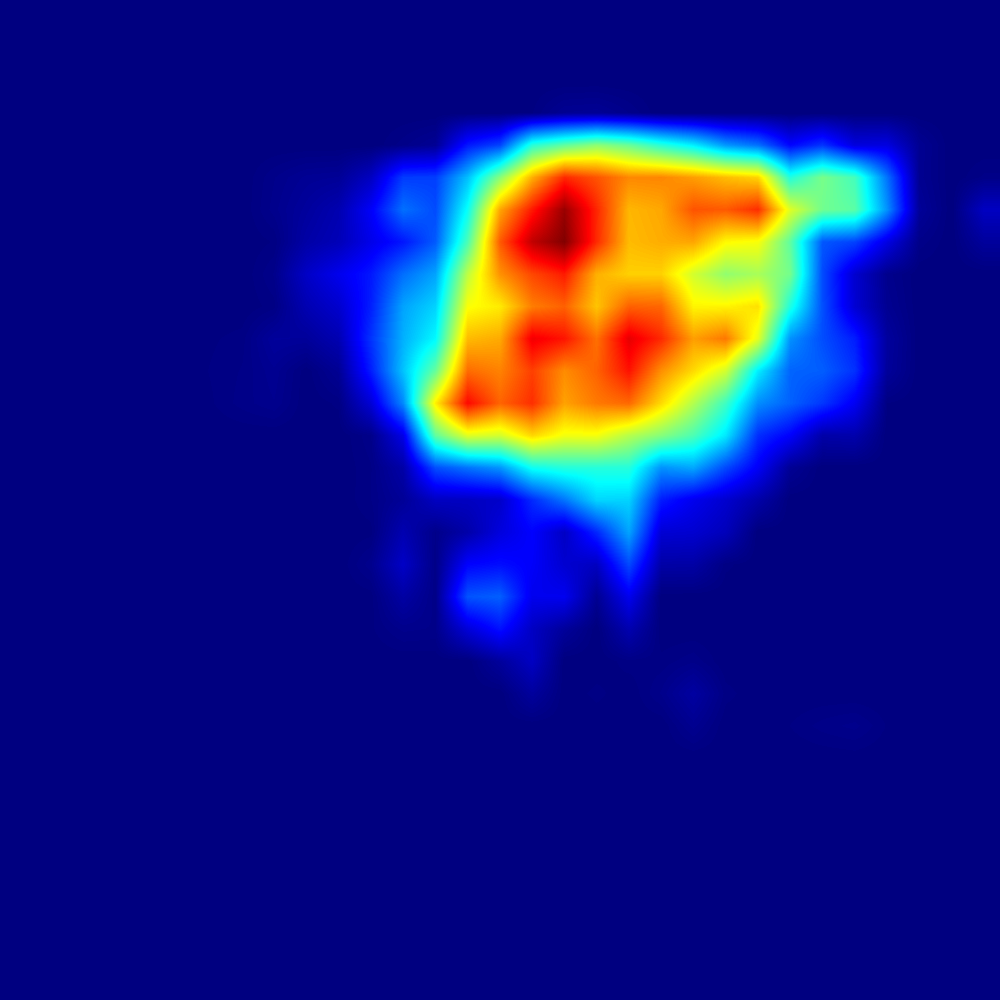

Supplement: Supplementary file 3 — Source Data File [file 41746_2022_681_MOESM3_ESM.zip › ARDA Map/Figure 3/34.png]

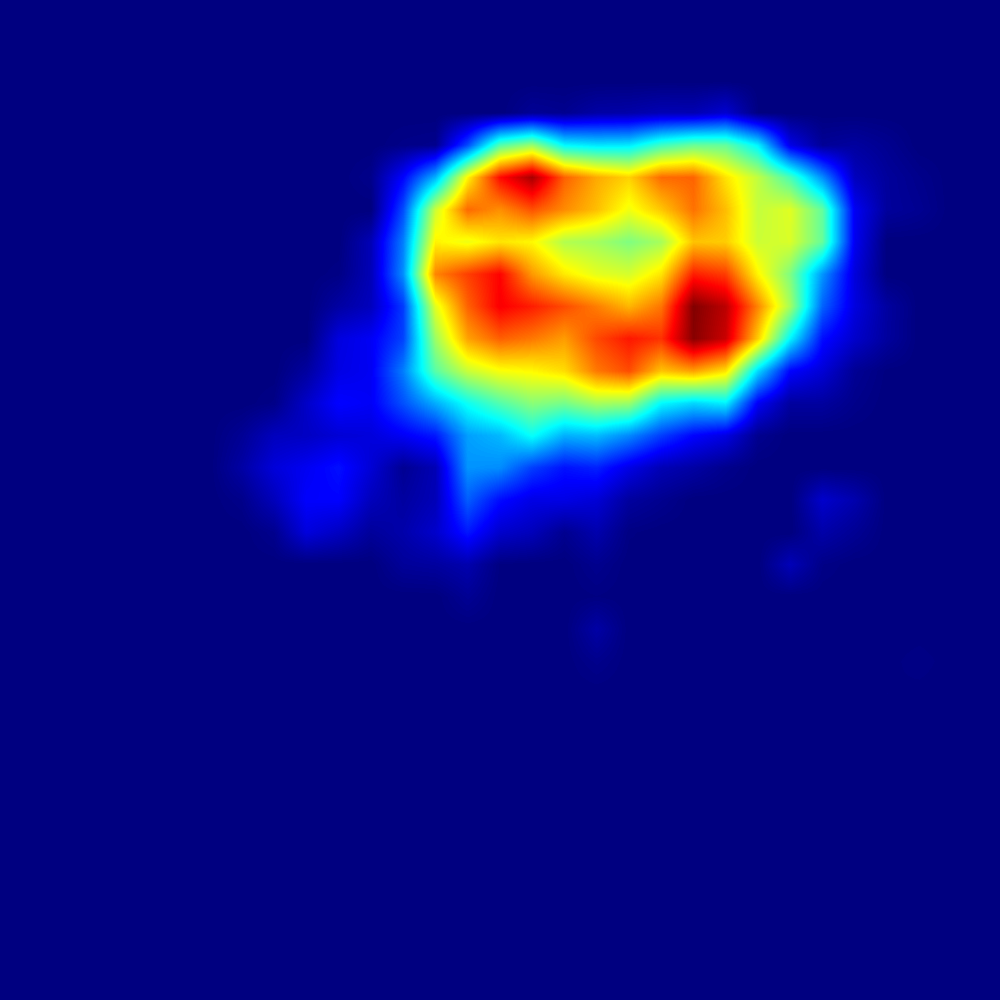

Supplement: Supplementary file 3 — Source Data File [file 41746_2022_681_MOESM3_ESM.zip › ARDA Map/Figure 3/35.png]

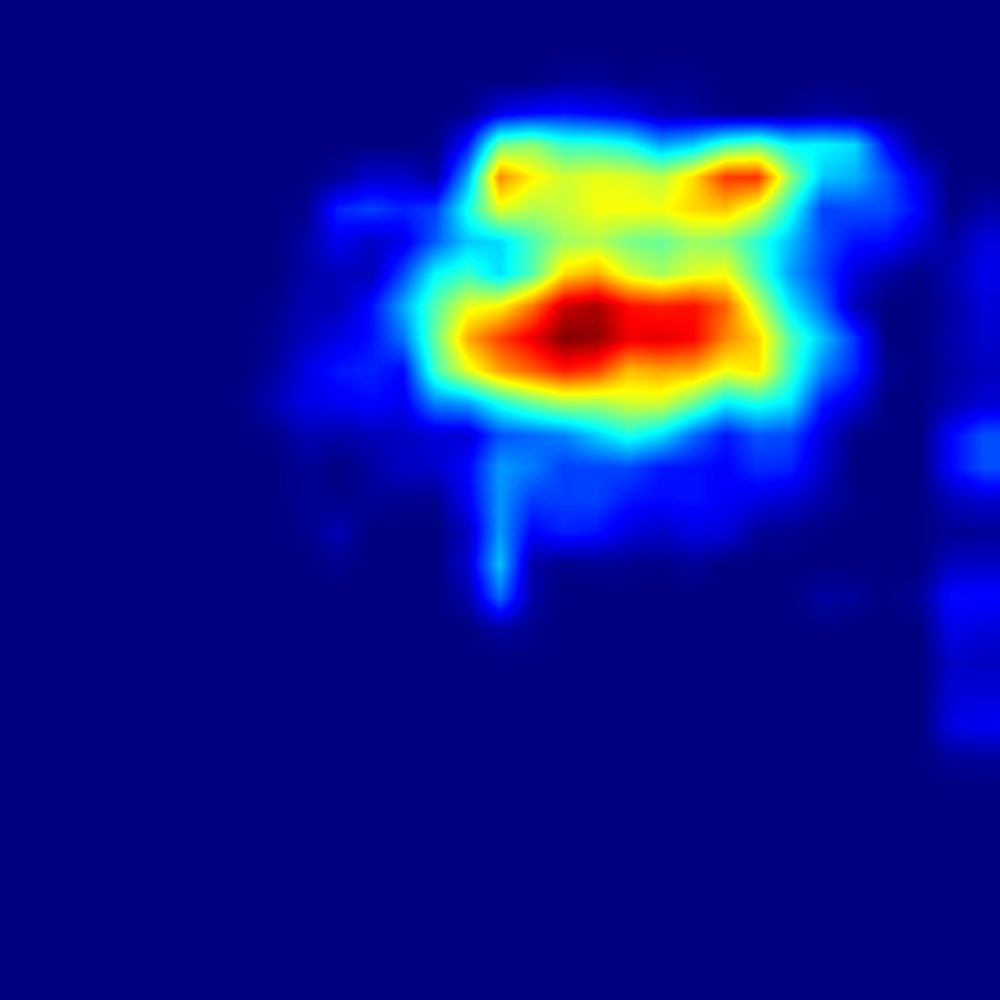

Supplement: Supplementary file 3 — Source Data File [file 41746_2022_681_MOESM3_ESM.zip › ARDA Map/Figure 3/36.png]

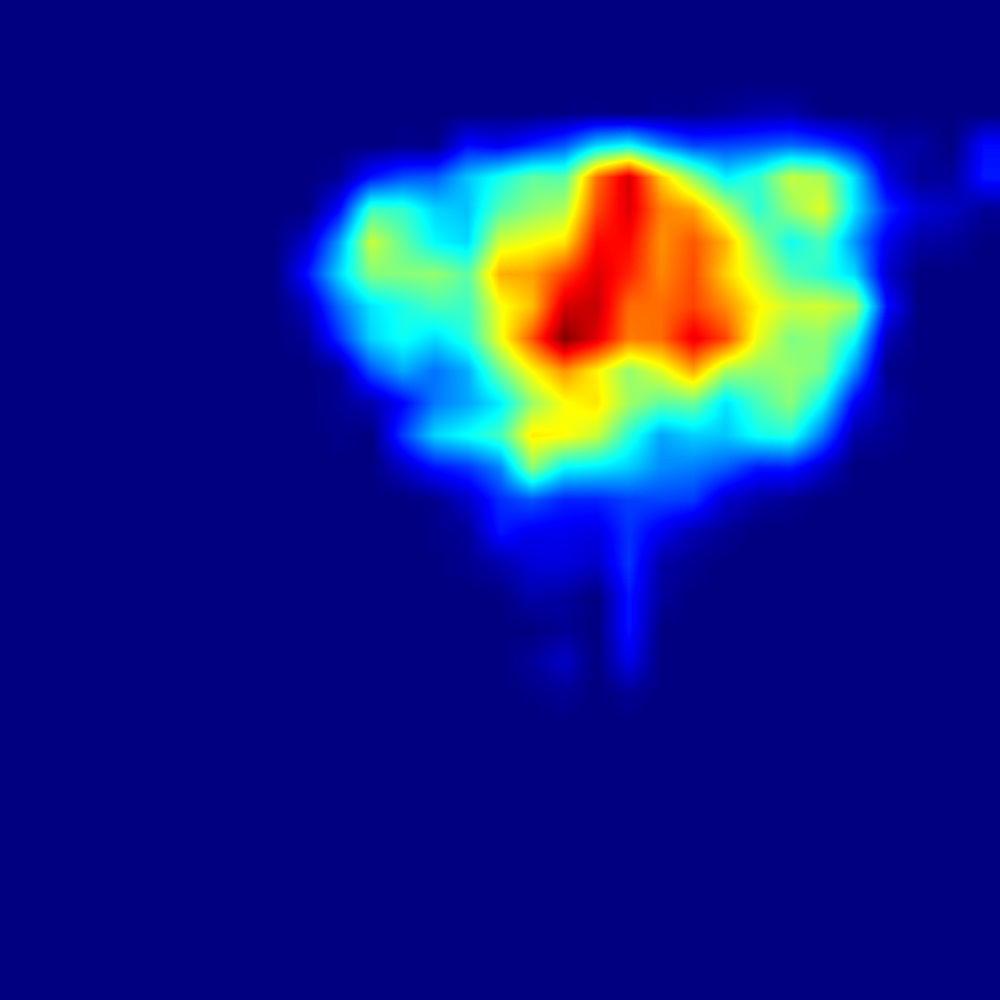

Supplement: Supplementary file 3 — Source Data File [file 41746_2022_681_MOESM3_ESM.zip › ARDA Map/Figure 3/37.png]

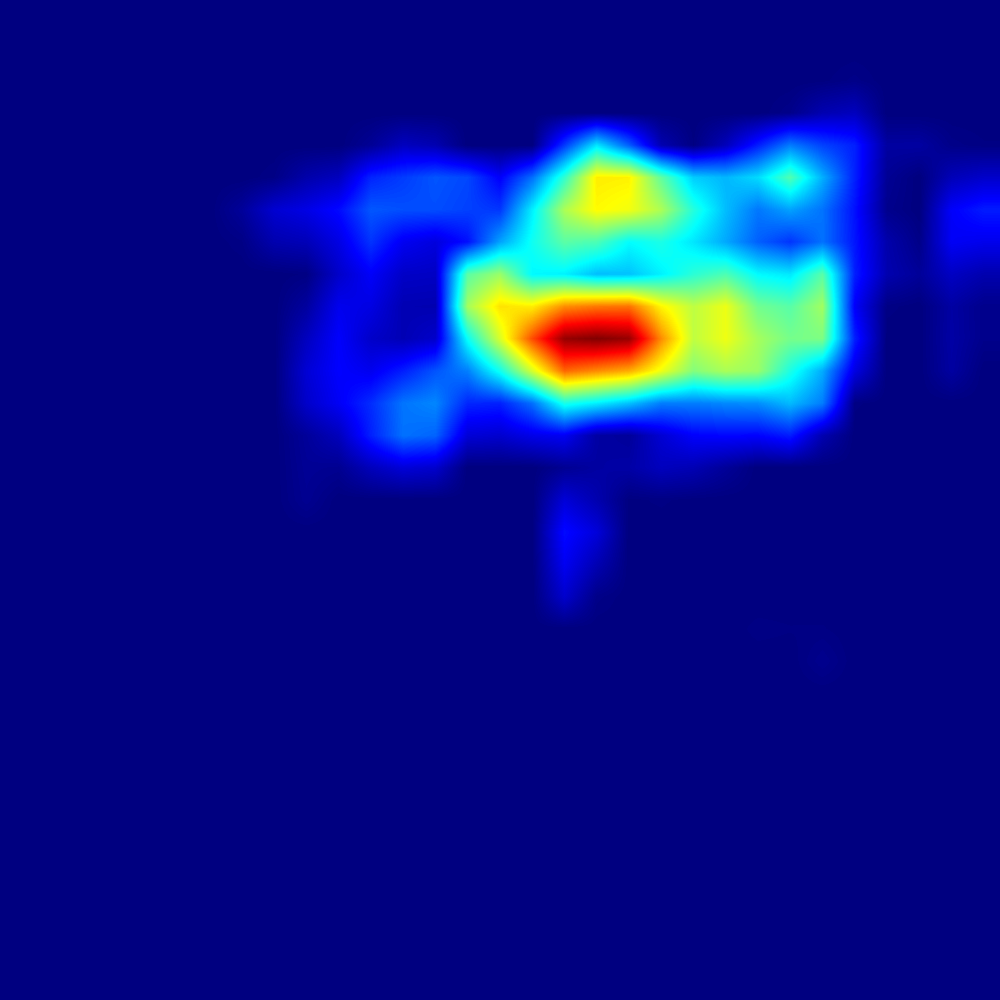

Supplement: Supplementary file 3 — Source Data File [file 41746_2022_681_MOESM3_ESM.zip › ARDA Map/Figure 3/38.png]

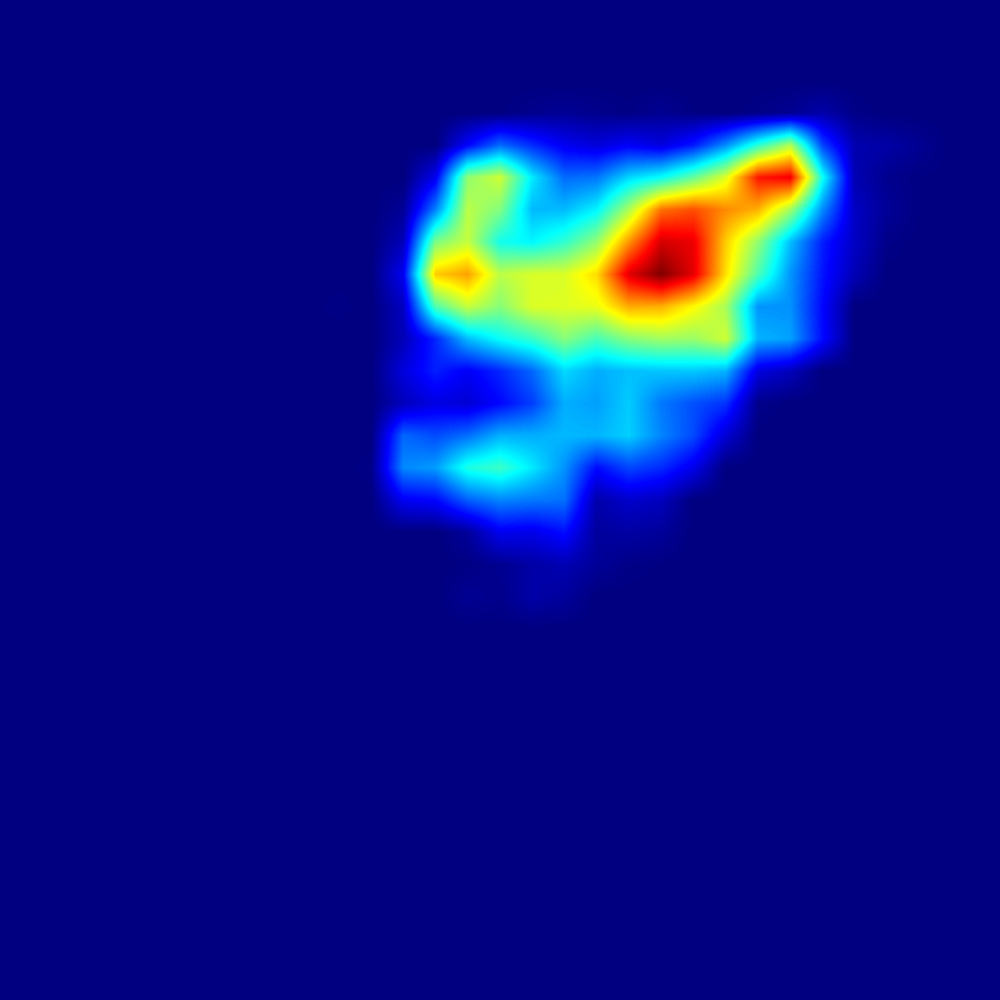

Supplement: Supplementary file 3 — Source Data File [file 41746_2022_681_MOESM3_ESM.zip › ARDA Map/Figure 3/39.png]

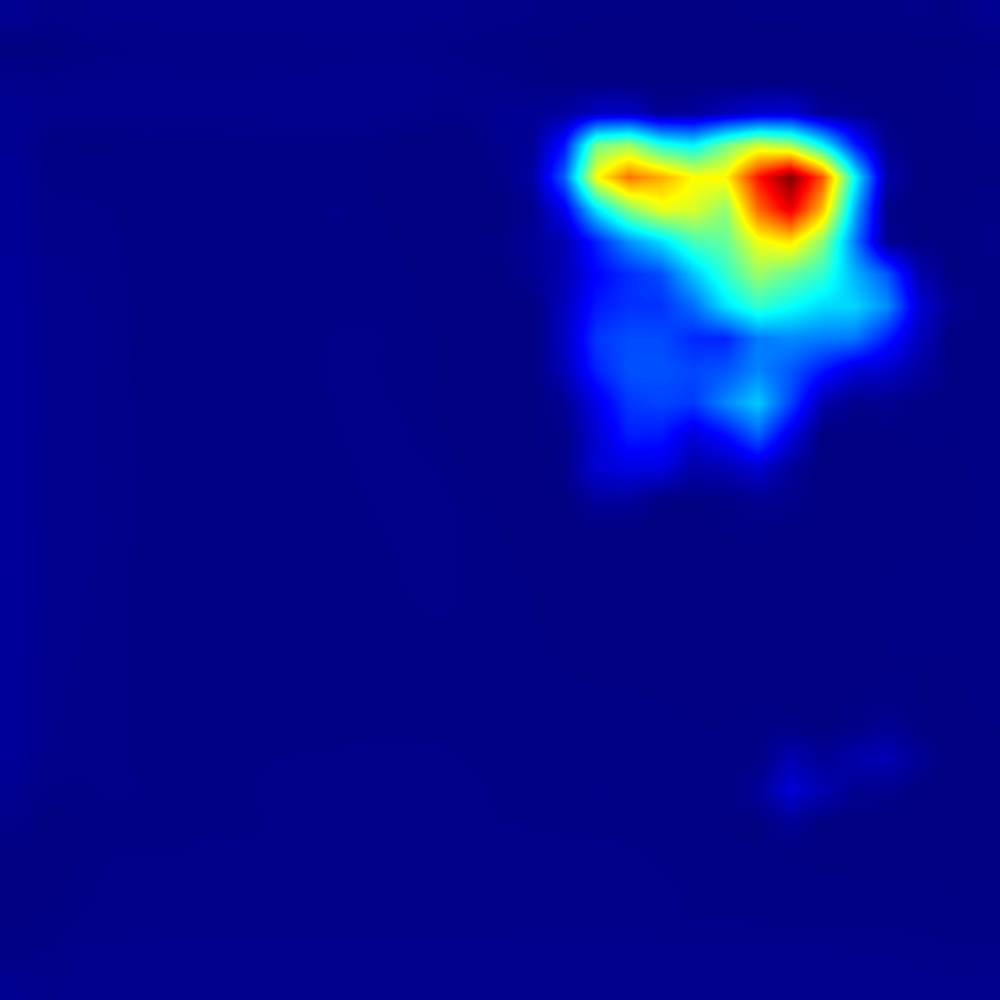

Supplement: Supplementary file 3 — Source Data File [file 41746_2022_681_MOESM3_ESM.zip › ARDA Map/Figure 3/4.png]
